# Characteristics and impact of real-world evidence studies in oncology: comprehensive mapping review of publications evaluating targeted therapies in solid tumours

**DOI:** 10.1016/j.esmorw.2024.100091

**Published:** 2024-12-03

**Authors:** A. Pellat, T. Grinda, P. Cresta Morgado, A. Prelaj, V. Miskovic, A. Valachis, I. Zerdes, D. Martins-Branco, L. Provenzano, A. Spagnoletti, G. Nader-Marta, B.E. Wilson, Y.-H. Yang, G. Pentheroudakis, S. Delaloge, L. Castelo-Branco, M. Koopman

**Affiliations:** 1 Department of Gastroenterology, Endoscopy and Digestive Oncology, Hôpital Cochin APHP, Paris, France; 2 Center for Research in Epidemiology and Statistics (CRESS-U1153), Université Paris Cité and Université Sorbonne Paris Nord, INRAE, Inserm, Hôpital Hôtel-Dieu, Paris, France; 3 Department of Cancer Medicine, Gustave Roussy, Villejuif, France; 4 Prostate Cancer Translational Research Group, Vall d’Hebron Institute of Oncology (VHIO), Barcelona, Spain; 5 Oncology Data Science, Vall d’Hebron Institute of Oncology (VHIO), Barcelona, Spain; 6 Medical Oncology Department, Fondazione IRCCS Istituto Nazionale Tumori, Milan, Italy; 7 Department of Electronics, Information, and Bioengineering, Politecnico di Milano, Milan, Italy; 8 Department of Oncology, Faculty of Medicine and Health, Örebro University Hospital, Örebro University, Örebro, Sweden; 9 Department of Oncology-Pathology, Karolinska Institute, Stockholm, Sweden; 10 Theme Center, Karolinska University Hospital, Karolinska Comprehensive Cancer Center, Stockholm, Sweden; 11 Scientific and Medical Division, European Society for Medical Oncology, Lugano, Switzerland; 12 Academic Trials Promoting Team (ATPT), Institut Jules Bordet, Hôpital Universitaire de Bruxelles (HUB), Université Libre de Bruxelles (ULB), Brussels, Belgium; 13 Department of Oncology, Queen’s University, Kingston, Canada; 14 Division of Cancer Care and Epidemiology, Queen’s Cancer Research Institute, Kingston, Canada; 15 National Institute of Cancer Research, National Health Research Institutes, Tainan, Taiwan; 16 Medical Oncology Division, Oncology Institute of Southern Switzerland, Ente Ospedaliero Cantonale, Bellinzona, Switzerland; 17 Department of Medical Oncology, University Medical Center Utrecht, Utrecht University, Utrecht, The Netherlands

**Keywords:** mapping review, network analysis, oncology, real-world evidence, solid tumours, targeted therapy

## Abstract

**Background:**

A mapping review of real-world evidence (RWE) publications on targeted therapy (TT) for solid tumours was carried out to describe their characteristics, strengths, and limitations.

**Methods:**

RWE publications were identified that: (i) focused on TTs in patients with solid tumours; (ii) included study objectives of effectiveness, predictive or prognostic factors, safety or quality of life; (iii) were published between 1 January 2020 and 22 December 2022. Associations between study variables and journal impact factor (IF) were explored through regression and cluster network analyses.

**Results:**

Of 7775 publications identified, 1251 were eligible for analysis. The number of publications per year increased over time. Most studies were conducted in Asia (50%), Europe (25%), and North America (17%), with only 8% in more than one country. Data sources were mostly health records (55%) and registries (11%). Most studies were retrospective (85%) and only 16% were population based. Gastrointestinal tumours were the most frequently studied (30%), followed by lung (22%) and breast (21%). The median journal IF was 4.4. Involvement of >10 centres and studies originating from Europe were significantly associated with a higher IF (≥7) in multivariable analysis. Network analysis demonstrated strong associations between countries and the number of publications in specific tumour types.

**Conclusions:**

The number of RWE publications on TT for solid tumours is increasing, but studies are heterogeneous, mostly retrospective, and published in low IF journals. International collaboration and promotion of standardised data sources is imperative to enhance the relevance of RWE research to complement clinical guidelines and impact clinical practice.

## Highlights

• This is a mapping review of real-world evidence publications.
• Analysed articles evaluated targeted therapy for solid tumours.
• The characteristics of 1251 publications were evaluated.
• The number of publications per year increased over time.
• Studies were heterogeneous and mostly based on retrospective data.

## Introduction

There has been an exponential increase in the volume of health data collected in the digital medicine era. Registries, electronic health records (EHRs), e-health services, wearable devices, and other technology-driven services, together with increased data storage capacity, have led to the rapid generation and availability of digital real-world data. The analysis of these observational data, collected outside of traditional clinical trials, is known as real-world evidence (RWE).^1^^,^^2^

RWE studies are of growing interest in oncology.^3^, ^4^, ^5^ Treatment strategies in routine clinical practice are mainly driven by guidelines issued by national and international scientific societies, which are based, when possible, on data from randomised controlled trials (RCTs). It is estimated, however, that only ∼5% of patients with cancer are enrolled in clinical trials due to strict study eligibility criteria which do not reflect real-world settings.^6^ For example, elderly patients with comorbidities or impaired performance status are generally underrepresented in RCTs.^7^^,^^8^ High-quality RWE studies are needed to re-evaluate approved treatments in real-world settings, directly compare novel drugs, provide valuable information for future experimental studies, and influence the development, testing, and approval of new treatments.

Recent progress in understanding tumour biology and advances in genomic sequencing have led to the development of several innovative oncology therapies, enabling a more tailored approach to cancer treatment.^9^ In this regard, targeted therapy (TT) has revolutionised the therapeutic landscape for patients with solid tumours, offering a more precise strategy than traditional chemotherapy.^1^^,^^10^, ^11^, ^12^, ^13^, ^14^, ^15^

TT is now widely used across the globe, with 51 different TTs approved by the Food and Drug Administration between 2006 and 2020 for 18 cancer types; this has increased the proportion of patients who can benefit from these therapies from 5% to 13.6%.^9^^,^^16^ TT includes small molecules that target specific biomarkers, such as anaplastic lymphoma kinase (ALK), epidermal growth factor receptor (EGFR) or human epidermal growth factor receptor 2 (HER2), as well as multitargeted inhibitors that act on several biological pathways.^17^^,^^18^ TT also includes monoclonal antibodies that interfere with extramembrane receptors and antibody–drug conjugates (ADCs) that deliver cytotoxic agents specifically to the tumour.

While postmarketing surveillance data and RWE can help us to understand the effectiveness and safety of TT in populations not eligible for RCTs, premarketing RWE can contribute to efficacy evaluation, particularly in rare cancers or in the case of rare molecular alterations amenable to TT, when real-world data may be used in synthetic control arms.^5^^,^^19^ The inappropriate use of RWE, however, can lead to erroneous conclusions or the publication of only positive studies, with a high risk of publication bias.^20^^,^^21^ Finally, the current use and quality of RWE studies assessing TT may differ according to tumour type and study region; therefore, mapping these data can help to improve future research and collaboration.

A comprehensive overview of RWE studies in oncology is needed to describe the strengths and limitations of recent publications, identify the gaps in knowledge and explore potential new approaches to RWE data analysis and interpretation. To address this need, a comprehensive mapping review was carried out to systematically describe the characteristics of RWE studies published in a 3-year period, focusing on the evolving field of TT for the treatment of patients with solid tumours.

## Methodology

### Search strategy

A systematic PubMed search (Medline and PubMed Central) was carried out to identify all RWE studies assessing TTs in solid tumours that were published between 1 January 2020 and 22 December 2022. The journal publication date was used whenever available; otherwise, the ePub date was used. We did not consider filters for exclusion criteria on PubMed (e.g. ‘clinical trials’) to avoid automatic exclusion of some mislabelled cases, opting for a human screening of eligible publications. Full details of the search strategy are available in Table A1 in Supplementary Appendix A, available at https://doi.org/10.1016/j.esmorw.2024.100091.

### Eligibility criteria

All RWE studies that assessed TTs (alone or in combination with other treatment types) in solid tumours were considered. TTs included small molecules, monoclonal antibodies, and ADCs, in line with the European Society for Medical Oncology (ESMO) Handbook of Targeted Therapies and Precision Oncology,^22^ but immunotherapy and endocrine therapy were excluded. Studies evaluating one or more of the following objectives were included: (i) effectiveness, (ii) treatment strategy, (iii) predictive or prognostic factors or biomarkers with effectiveness analysis, (iv) safety, (v) quality of life (QoL), and (vi) patient-reported outcomes (PROs). Clinical trials, preclinical studies, reviews, perspectives, studies without accessible full text available in English, and studies with a patient sample size <100 were excluded. The exclusion criterion of total number of patients was not considered in the case of safety studies reporting adverse events (AEs) as a unit of analysis rather than patients. The full inclusion and exclusion criteria for study selection are available in Table A2 in Supplementary Appendix A, available at https://doi.org/10.1016/j.esmorw.2024.100091.

### Publication screening and selection

Identified publications were exported to Covidence™ software (Covidence systematic review software, Veritas Health Innovation, Melbourne, Australia). Seven authors (APe, TG, APr, AV, IZ, BEW, and DMB) screened all titles and abstracts in parallel and identified publications meeting the prespecified eligibility criteria. Duplicates were deleted by Covidence™ upon verification by the authors. Full publications were then assessed by two authors (APe and TG) in parallel to confirm eligibility for data extraction. Conflicts were resolved through discussion.

### Data extraction

Data from the eligible articles were extracted independently by 12 authors (APe, TG, APr, PCM, AV, IZ, BEW, DMB, GNM, AS, LP, and LCB) using a standardised extraction form created using Covidence™. The form was initially tested on a small number of selected publications, further revised and then accepted in its final form by all authors (Table A3 in Supplementary Appendix A, available at https://doi.org/10.1016/j.esmorw.2024.100091). The assessment was not carried out in duplicate.

### Data synthesis and statistical analysis

Exploratory analyses and descriptive statistics were carried out by three authors (APr, VM and PCM). Continuous variables were summarised using mean and standard deviation or median and interquartile range (IQR) according to the variable distribution. Categorical variables were presented as counts and percentages.

Eligible publications were analysed based on the year of publication and the journal impact factor (IF), which was categorised as <7 or ≥7. We defined IF as the yearly mean number of citations of articles published in the last 2 years in the journal. We chose 2022 as the reference year and the journals’ IF were obtained from Clarivate. The IF cut-off was chosen as it corresponds roughly to the first quartile of the IF value distribution from currently available oncology journals.^23^ A *post hoc* logistic regression analysis was carried out to explore the association between extracted study-related characteristics or variables and IF. We also carried out a *post hoc* analysis assessing the association with IF as a continuous variable and the number of centres (one versus more than one) with the same study-related characteristics. In total, 11 predefined study-related characteristics were analysed: region (Asia, Africa, Latin America and the Caribbean, Europe, Northern America, Oceania or more than one region), tumour type [breast, lung, gastrointestinal (GI) or other], study design (retrospective cohort, prospective cohort or other), population-based study or not, comparative study or not, number of centres involved (1, 2-5, 6-10 or >10), study objectives (effectiveness, treatment strategy, predictive or prognostic, safety, QoL and PROs, or more than one objective), sample size, funding (industry based, no funding, not reported or unclear, or non-industry based), study range (national or international), and type of treatment (TT alone or in combination).

To study the association with journal IF, the 11 study-related variables were evaluated in a univariable model. Variables with a *P* value <0.1 on univariable analysis then underwent multivariable analysis. The effect measurement between explanatory variables and IF was described by the odds ratio and its 95% confidence interval. No imputation was carried out in any step. The significance level was alpha = 0.05 (two-sided) for all tests. No correction for multiple testing was applied. Analyses were carried out using R software, version 4.3.0.^24^

To evaluate countries of origin, each publication was categorised based on the corresponding author’s country of affiliation. GeoPandas library version 0.13.2^25^ was then used for geospatial data manipulation and world map visualisations were generated using Matplotlib.^26^ Publications that lacked geographical information were manually verified and added to the final database. A network analysis was carried out using NetworkX version 3.2.1^27^ to construct and analyse the weighted network graph. Graph nodes were positioned using the Kamada–Kawai path-length cost function.^28^ The Louvain algorithm was applied to identify distinct groups of interconnected nodes and detect underlying community structures within the network.^29^

## Results

### Description of identified publications

**Figure 1:**
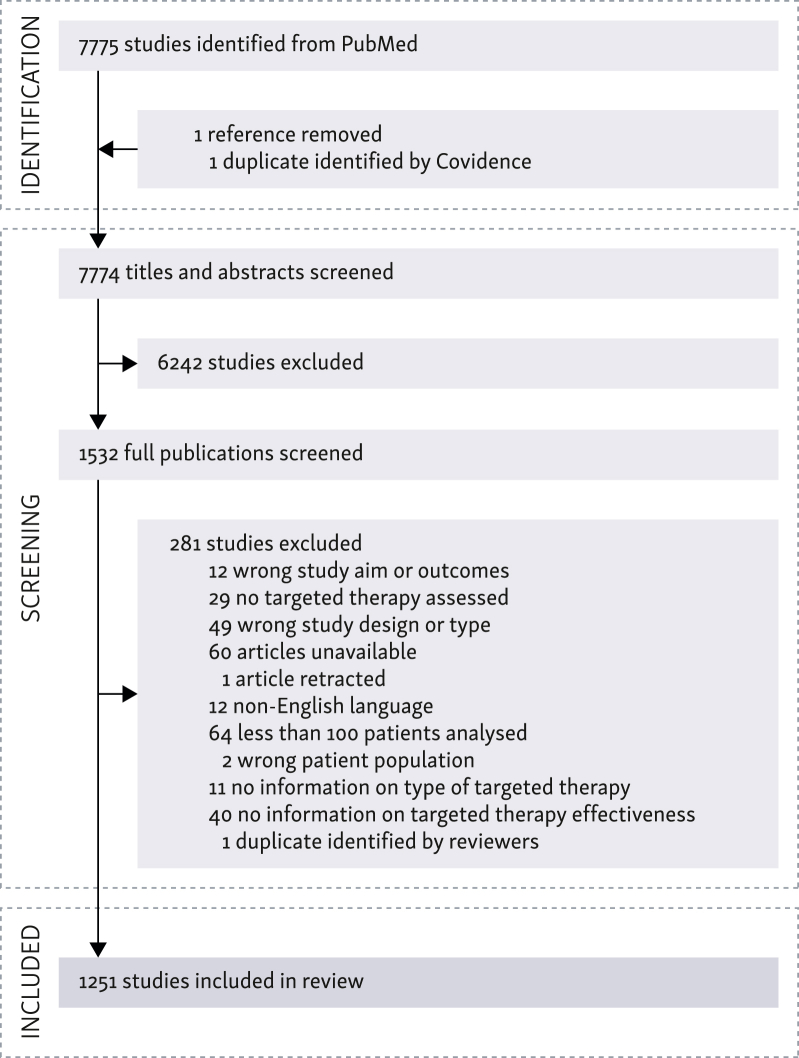
**Flow chart for publication identification and screening.** TT, targeted therapy.

In total, 7775 publications were identified in the initial systematic PubMed search. After title and abstract screening, 6242 publications were excluded because they did not fulfil the eligibility criteria. Most of the excluded articles reported interventional studies or analyses involving <100 patients. After full publication screening, 1251 articles were selected for data extraction (see Figure 1). A full list of the 1251 publications analysed is available in Supplementary Appendix B, available at https://doi.org/10.1016/j.esmorw.2024.100091.

The main characteristics of the extracted studies are shown in Table 1 and Table A4 in Supplementary Appendix A, available at https://doi.org/10.1016/j.esmorw.2024.100091. Almost all studies evaluated adult patients (99%) and the most common tumour types were GI (30%), lung (22%), and breast (21%). Fifty percent of studies were conducted in Asia, 25% in Europe, and 17% in North America. Only 8% and 5% of studies involved patients treated in more than one country and more than one region, respectively (according to World Health Organization classification). Notably, 41% of studies were conducted in a single centre. Small molecules were the most frequently investigated type of TT (62%). While 51% of studies had multiple objectives, 25%, 12%, and 11% only assessed effectiveness, predictive or prognostic factors with effectiveness analysis, and safety, respectively. Among 1082 studies evaluating effectiveness via one of their objectives, the most common endpoints were overall survival in 84% of studies and disease-, recurrence-, or progression-free survival in 65%. The most frequent data sources were health records (55%), different types of registries (11%), and standardised data including case report forms and standardised questionnaires (8%). The majority of studies were retrospective cohort studies (85%) and only 16% were defined or judged as population-based studies. The median sample size of the analysed populations was 256 (IQR 147-620). Most studies were published in a specialist medical journal (86%), while 9% and 5% were published in general medical and non-medical journals, respectively. Seventeen percent of studies were totally or partially funded by industry, while funding information was not reported or was unclear in 26% of the publications.

**Table 1:** Main study characteristics of all publications analysed^a^

Study characteristics	*n* (%)
Tumour type	
Gastrointestinal^b^	377 (30)
Lung	281 (22)
Breast	265 (21)
Genitourinary^c^	111 (9)
Melanoma and other skin cancers	50 (4)
Head and neck	37 (3)
Gynaecological	35 (3)
Other tumours	44 (4)
>1 Tumour type	46 (4)
Not reported	5 (<1)
Region of treatment	
Asia	630 (50)
Europe	310 (25)
North America	210 (17)
Other parts of the world	34 (3)
>1 Region	67 (5)
Type of targeted therapies	
Small molecule	774 (62)
Monoclonal antibody	378 (30)
ADC	10 (1)
>1 Type	89 (7)
Study design	
Retrospective cohort	1060 (85)
Prospective cohort	102 (8)
Other	60 (5)
Not reported	29 (2)
Type of data source	
(Electronic) health records	682 (55)
Registry	141 (11)
Standardised data (CRF or other questionnaires)	106 (8)
Administrative or claims data	60 (5)
Health data aggregator	40 (3)
>1 Source	96 (8)
Other	16 (1)
Not reported	110 (9)
Number of centres	
1	513 (41)
2-5	146 (12)
6-10	67 (5)
>10	237 (19)
Not reported	288 (23)
Comparative study	
Yes	599 (48)
No	652 (52)
Study objective(s)	
Effectiveness	308 (25)
Predictive or prognostic (with effectiveness analysis)	149 (12)
Safety	142 (11)
Treatment strategy	13 (1)
QoL or PROs	7 (<1)
>1 objective	632 (51)

ADC, antibody–drug conjugate; CRF, case report form; PRO patient-reported outcome; QoL, quality of life.

a Expanded study characteristics are described in Table A4 in Supplementary Appendix A, available at https://doi.org/10.1016/j.esmorw.2024.100091.

b Gastrointestinal tumours included rectal, biliary, gastric, oesophageal, pancreatic, colorectal, anal cancer, and hepatocellular carcinoma.

c Genitourinary tumours included prostate, bladder, renal and penile cancer, and testicular seminoma and non-seminoma.

### Geographical mapping

**Figure 2:**
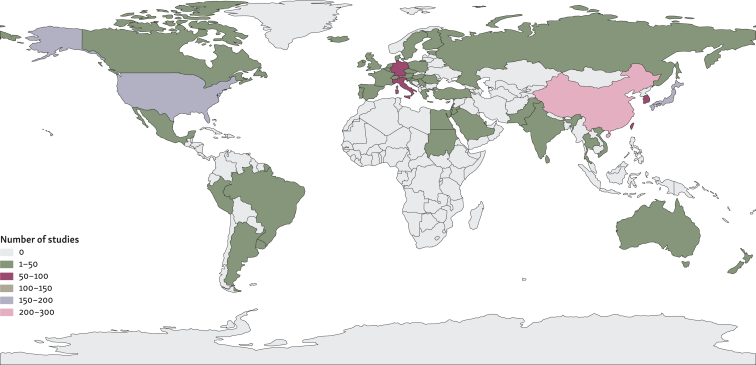
**Number of publications per country.**^**a**^ ^a^The country of origin for each publication was derived from the corresponding author’s affiliation (total of 54 countries).

China was associated with the highest number of publications (*n* = 275). In total, three countries were each associated with >100 publications: China, Japan, and USA) (Figure 2). Notably, while the country of origin of each publication was extracted from the corresponding author’s affiliation, this occasionally differed from the study region. Disparities were observed in 19 studies where the affiliation of the corresponding author did not align with the region in which the research was carried out. In all cases, the region of the corresponding author’s affiliation was used in the analysis.

### Network cluster analysis

**Figure 3:**
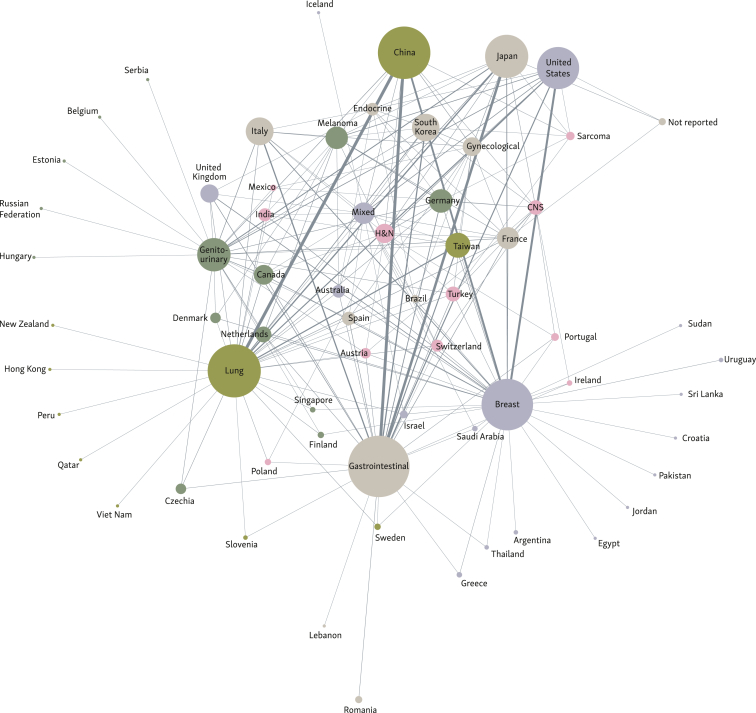
**Network cluster analysis representing tumour types and countries involved in the analysed sample of publications.**^**a**^ CNS, central nervous system; H&N, head and neck; OR, odds ratio. ^a^Graph illustrates relationships between source nodes (representing the country of the publication) and target nodes (representing the studied cancer types). The size of the source and target nodes are proportional to the total number of publications issued from the respective country and focused on a specific tumour type, respectively. Line thickness corresponds to the number of publications on tumour types in the connected countries. Colours represent communities of interconnected nodes.

A pattern emerged regarding the distribution of cancer research studies across countries (Figure 3). The network cluster analysis confirmed that China, Japan, and USA were associated with the highest numbers of publications. It also demonstrated that some countries were more frequently associated with studies on certain tumour types. GI cancer publications (*n* = 377) originated from 27 countries but were mainly associated with Japan and China. Lung cancer publications (*n* = 281) originated from 32 countries, especially China and USA. Breast cancer publications (*n* = 265) were mapped to 24 countries, but were mostly from China and USA. Therefore, China exhibited a prominent focus on all three of the most commonly evaluated types of cancer (GI, lung, and breast). A relatively lower number of publications evaluated genitourinary cancers (*n* = 111), but 24 distinct countries contributed to this research, highlighting diverse global engagement for this tumour type.

### Changes in study characteristics over time (2020-2022)

**Figure 4:**
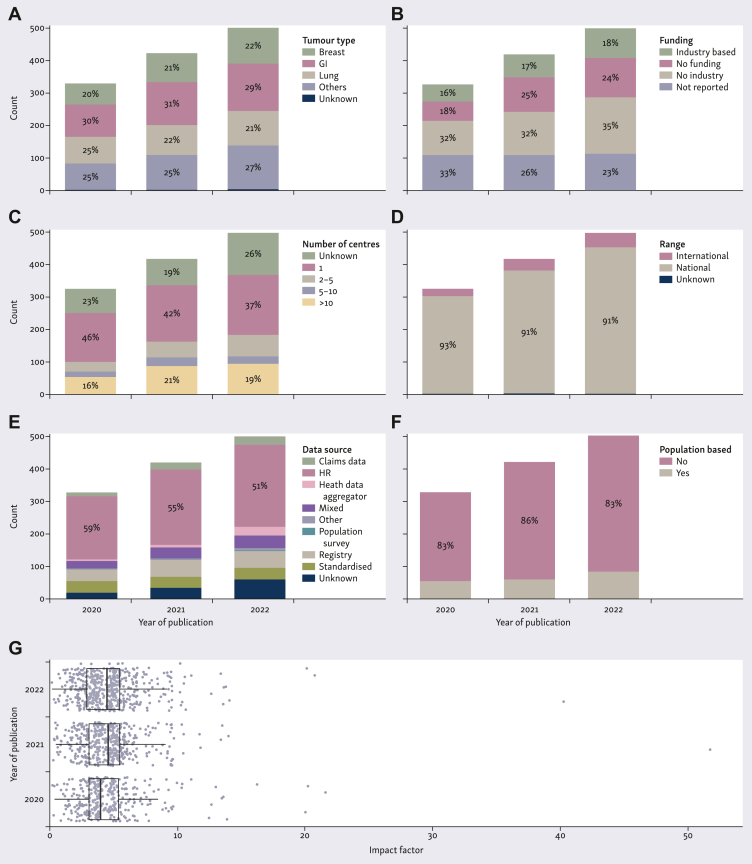
**Changes in main study characteristics over time (2020-2022).** Tumour type (A), funding (B), number of centres involved (C), study range (D), data source (E), population-based studies (F), and IFs of journals publishing the study (G).^a^ GI, gastrointestinal; HR, health record; IF, impact factor; pop survey, population survey. ^a^Percentages are stated for proportions >15%.

The number of eligible publications per year increased over the 3-year review period (*n* = 328 in 2020, *n* = 421 in 2021, and *n* = 502 in 2022) (Figure 4A). Publications on all types of cancers increased numerically, with GI tumours remaining the most represented (Figure 4A). The number of studies assessing small molecules increased numerically with 221, 288, and 349 studies in 2020, 2021, and 2022, respectively. Similarly, there were 123, 156, and 186 studies assessing monoclonal antibodies in 2020, 2021, and 2022, respectively. There was a slight numerical increase in the share of funding attributed partially or totally to industry over time (16%, 17%, and 18% in 2020, 2021, and 2022, respectively), with the high proportion of not reported or unclear funding reducing over time (Figure 4B). Studies remained mainly single centre (46%, 42%, and 37% in 2020, 2021, and 2022, respectively) (Figure 4C) and were mainly national (Figure 4D). Health records remained the primary data source (Figure 4E) and studies were mostly not population based (Figure 4F). The distribution of journal IFs remained numerically unchanged over time (Figure 4G).

### Study-related characteristics associated with impact factor

The median IF across the analysed sample of 1251 publications was 4.4 (IQR 3-5.3). There was an asymmetric distribution of IFs (Figure 4G); while 25% of articles were published in journals with an IF >5.3, only 11% were published in journals with an IF ≥7.

**Figure 5:**
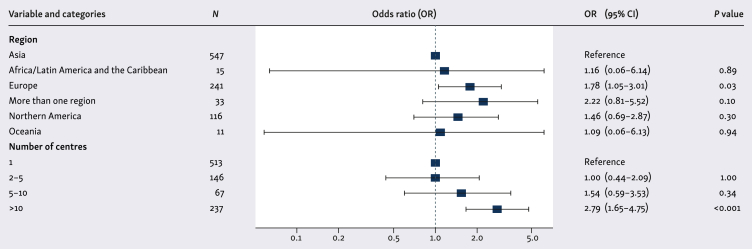
**Multivariable analysis assessing the association of study-related characteristics and journal IF.** CI, confidence interval; IF, impact factor; OR, odds ratio.

Of the 11 predefined variables assessed on univariable analysis (Figure A1 in Supplementary Appendix A, available at https://doi.org/10.1016/j.esmorw.2024.100091), only four study characteristics were significantly associated with publication in a journal with an IF ≥7: number of centres, study region, study range, and population-based study. On multivariable analysis, two variables remained significantly associated with publication in a higher IF journal: study region and number of centres (Figure 5). Studies originating in Europe (compared with Asia) and studies involving >10 centres were more likely to be published in a journal with an IF ≥7. Regarding the *post hoc* analysis assessing the association with IF as a continuous variable, we found the same four characteristics with significant associations.

A *post hoc* logistic regression analysis was also carried out with the same method to explore the association of variables and number of centres (one versus more than one). Here, IF was evaluated as 1 of the 11 variables, replacing number of centres in the previous analysis. In the multivariable analysis, seven variables were significantly associated with the number of centres: region, tumour type, design, population-based, IF, objective, and funding. Prospective studies, carried out in Europe or Oceania, in lung tumours and published in an IF ≥7 were associated with a multicentric setting (data not shown).

## Discussion

This mapping review demonstrated that the number of RWE publications focusing on TT in patients with solid tumours has numerically increased in recent years. Most of the analysed publications reported results from retrospective cohort studies using medical records as a data source, with limited international collaboration and relatively small sample sizes. The median journal IF of the analysed publications was relatively low (IF 4.4), but a study origin of Europe and involvement of >10 centres were independently associated with publication in higher IF journals (IF ≥7).

In terms of geographical distribution, China was associated with the highest number of publications, especially those relating to GI and lung tumours. This may be explained in part by epidemiological factors, as China is one of the most populated countries worldwide, with increasing medical research activity (and thus associated publications) and a high prevalence of cancers potentially treated with TT, such as lung or digestive tract tumours.^30^ The implementation of China’s national health care reform plan may also contribute, as it promotes the digitalisation of health care and hospital management with the use of EHRs, which could facilitate their secondary use for research purposes.^31^^,^^32^ Indeed, a systematic literature review that aimed to identify generic (non-disease-specific) real-world data showed a predominant use of health records data from China.^33^ Interestingly, Italy and South Korea were among the five countries associated with the most publications in the current review, despite each having <60 million inhabitants. This may be partially explained by the existence of national population-based claims data services that can be accessed for population-based research, such as the National Health Insurance Service in South Korea, and national registries, such as the Italian Association of Cancer Registries (AIRTUM) database.^34^^,^^35^ Also, the wide variation in availability and expenditure for most licensed anticancer drugs, including TTs, across different countries could also have influenced the geographical distribution of publications in the studied sample.^36^ Indeed, in lower-middle- and low-income countries, many anticancer drugs, such as innovative TTs, are not available or only available at full cost as an ‘out of pocket’ expense for patients, and accessibility can also be impacted by unreliable supply.^36^ Finally, geographical distribution might have also been influenced by differences in the willingness to publish, with some medical teams in community or academic centres being more motivated to report their experience.

The number of articles published per year increased over the 3-year review period, suggesting a growing interest in RWE over time. There was also a slight increase in funding by industry over time, illustrating industry’s interest in real-world data. Nevertheless, the RWE publications in the analysed sample were heterogeneous in design and setting and mostly relied on retrospective data with relatively small sample sizes and few collaborative centres. These features can be problematic when assessing both effectiveness and safety. Indeed, most of the studies were single centre with limited population-based coverage, which can affect the generalisability of study results. This seems to be a paradox, as one potential advantage of RWE studies is increased external validity when compared with clinical trials, but this is certainly not the case when the RWE studies are monocentric or even national, as this can result in selection bias.

This analysis demonstrated that RWE studies involving >10 centres and those originating from Europe were more likely to be published in a high IF journal. Interestingly, population-based studies, which are often regarded as higher quality RWE, were not a predictor for publication in a high IF journal after adjusting for other variables. It should be noted, however, that the number of population-based studies was small and, as these studies often have longer time gaps between the study date and last date of data coverage, recent population-based TTs studies may not have been captured within the inclusion criteria of this review.

Retrospective data can be less reliable than prospective data when capturing low-grade but potentially clinically-impactful AEs, and may, therefore, give a misleading representation of treatment effects.^37^ RWE studies are generally at risk of bias and are subject to confounding.^38^^,^^39^ The quality and reliability issues associated with RWE have resulted in its current use as supporting (rather than definitive) evidence in the regulatory approval of novel TTs.^5^ A recent study reported that when RWE studies were considered by the European Medicines Agency (EMA) for regulatory clinical efficacy evaluation of TTs, the RWE complemented the regulatory decision but was not conclusive for regulatory authorisation of new TTs indications.^5^ This confirms previous findings indicating that RWE data are currently generally of insufficient quality to inform clinical practice.^15^ Nevertheless, with the growing number of RWE studies in oncology and the development of new statistical methods and frameworks to deal with bias and confounding, such as trial emulation or the use of real-world data in pragmatic trials, the paradigm might already be changing.^40^, ^41^, ^42^

With all of this in mind, the current analysis found that 25% of studies were published in journals with an IF >5.3, with only 11% published in journals with an IF ≥7. IF is an imperfect indicator for publication quality assessment^43^ and should be considered very carefully as a metric of research quality. Although other metrics, such as the number of citations or downloads, could have been considered for analysis, IF was considered the most adequate metric for feasibility reasons (available at the journal level), variability (different number of citations in different sources), and time-depending reasons (citations or downloads are dependent of the date of publication). Also, it can be considered as a holistic measure from the whole scientific society in terms of novelty, innovation, as well as reviews based on journal experts/editors. Therefore, by using IFs, we may investigate the factors associated with general oncological interests in considering RWE. The results presented here may indicate that the currently available RWE has a low impact on clinical practice, or that high IF journals do not consider RWE studies to be sufficiently compelling for publication. We also found that studies carried out in more than one centre were significantly associated with a higher IF (≥7). Finally, no other assessments could be done based on the available data.

This is the first mapping review carried out on RWE publications assessing TT in patients with solid tumours. The review will aid understanding of the current landscape of RWE, with a large number of assessed studies over a 3-year period, resulting in better representation of the recent research in this field. This analysis does, however, have several limitations. First, several authors were involved in the screening and extraction processes, which could have led to heterogenous data extraction; however, a clear extraction template was generated to minimise these discrepancies and the research team met regularly to discuss conflicts and homogenous interpretations. Second, by excluding studies with <100 patients, some important RWE studies in rare diseases may have been excluded, such as those in paediatric cancers or specific subpopulations (e.g. patients with specific comorbidities), for which interventional trials can rarely be carried out. In addition, the analysis focused on TT, so the results cannot be generalised to all types of anticancer treatment. This also implies an underrepresentation of some tumour types for which TT is not the cornerstone of treatment. Moreover, the decision to restrict the inclusion criteria to TT might have led to an underrepresentation of studies from some developing countries, where the incorporation of TT in clinical practice takes longer than in developed regions and is not always possible.^44^ As so few studies examining QoL or PROs were identified, the search strategy may not have adequately captured these endpoints; this could also reflect the lack of implementation of QoL assessment as part of routine clinical care. Such endpoints are difficult to capture, however, and may be more amenable to collection through emerging sources of real-world data such as wearable devices. Further similar research could more specifically map the publications evaluating PROs in patients treated with TT.

This mapping review revealed many remaining challenges regarding RWE generation in precision oncology, including difficulties around the quality measurement of studies, often unclear funding, prominent use of retrospective data sources, lack of collaborative efforts with few international studies and insufficient data sharing, and non-standardised reporting in publications. Therefore, much can still be done to improve the scientific and regulatory impact of RWE studies in clinical oncology.^45^ Collaborative efforts, such as the Data Analysis and Real World Interrogation Network (DARWIN EU) initiative, are crucial for establishing and expanding high-quality and validated data sources for use in medicine regulation. EMA guidance and frameworks can also help with quality assessment of RWE used for regulatory decision-making in the European Union. The development of common data models, such as the Observational Medical Outcomes Partnership (OMOP) models, will help to standardise real-world data and encourage data sharing and analytics compatibility between datasets, as well as multicentric and international research collaboration. The lack of uniform endpoint definitions (e.g. real-world progression-free survival) and standardised disease assessment methods in RWE studies remains to be addressed. Finally, standardised reporting in oncology publications, with the use of modern reporting guidance such as ESMO Guidance for Reporting Oncology real-World evidence (ESMO-GROW), must be encouraged to increase the relevance and future quality of RWE publications and their potential impact on oncology practice.^4^ Further studies shall assess if RWE oncology publications comply with the ESMO-GROW checklist and how the advent of such guidance may improve reporting quality.
