# Supplementary material for: Characteristics and impact of real-world evidence studies in oncology: comprehensive mapping review of publications evaluating targeted therapies in solid tumours
Source: ESMO Real World Data Digit Oncol. 2024 Dec 3;6:100091. doi: 10.1016/j.esmorw.2024.100091 (PMC12836563; doi:10.1016/j.esmorw.2024.100091)
Supplement: Supplementary Appendix A [file mmc1.docx]

Characteristics and impact of real-world evidence studies in oncology: comprehensive mapping review of publications evaluating targeted therapies in solid tumours

APPENDIX A

**Table A1 Full PubMed search strategy for identifying relevant publications**

| **PubMed search terms** |
| --- |
| (((((cancer*[tiab] OR carcinoma*[tiab] OR tumor*[tiab] OR tumour*[tiab] OR neoplasm*[tiab] OR adenocarcinoma*[tiab] OR malignan*[tiab] OR "Neoplasms"[Mesh]) AND ("cohort study"[tiab] OR "cohort analys*"[tiab] OR "observational study"[tiab ] OR "observational analys*"[tiab] OR "retrospective study"[tiab] OR "retrospective analys*"[tiab] OR "Case-control"[tiab] OR "case control"[tiab] OR "Historic study"[tiab] OR "historic analys*"[tiab] OR "cross-section*"[tiab] OR "cross section"[tiab] OR "longitudinal study"[tiab] OR "longitudinal analys*"[tiab] OR "interrupted time series"[tiab] OR "epidemiologic study"[tiab] OR "epidemiologic analys*"[tiab] OR emulat*[tiab] OR "target trial*"[tiab] OR "prevalence study"[tiab] OR "prevalence analys*"[tiab] OR "before after study"[tiab] OR "case-series"[tiab] OR "case series"[tiab] OR database*[tiab] OR registr*[tiab] OR "big data"[tiab] OR "claims data"[tiab] OR record*[tiab] or insurance[tiab] OR "routinely collected"[tiab] OR "real world"[tiab] OR "real-world"[tiab] OR files[tiab] OR administrative[tiab] OR "non interventional study"[tiab] OR "non-interventional study"[tiab] OR "non-interventional"[tiab] OR "non interventional" OR "non randomized"[tiab] OR "non-randomized"[tiab] OR "Routinely Collected Health Data"[Mesh] OR "Observational Studies as Topic"[Mesh] OR "Epidemiologic Studies"[Mesh] OR "Observational Study" [Publication Type]) AND ("targeted therap*"[tiab] OR "Molecular Targeted Therapy"[Mesh] OR Trastuzumab[tiab] OR Gefitinib[tiab] OR Erlotinib[tiab] OR Cetuximab[tiab] OR Bevacizumab[tiab] OR Sorafenib[tiab] OR Sunitinib[tiab] OR Panitumumab[tiab] OR Lapatinib[tiab] OR Temsirolimus[tiab] OR Pazopanib[tiab] OR Everolimus[tiab] OR Icotinib[tiab] OR Crizotinib[tiab] OR Vandetanib[tiab] OR Vemurafenib[tiab] OR Axitinib[tiab] OR Regorafenib[tiab] OR Pertuzumab[tiab] OR "Ado-trastuzumab emtansine"[tiab] OR Cabozantinib[tiab] OR Ponatinib[tiab] OR Afatinib[tiab] OR Trametinib[tiab] OR Dabrafenib[tiab] OR Ceritinib[tiab] OR Apatinib[tiab] OR Olaparib[tiab] OR Idelalisib[tiab] OR Ramucirumab[tiab] OR Alectinib[tiab] OR Cobimetinib[tiab] OR Palbociclib[tiab] OR Osimertinib[tiab] OR Sirolimus[tiab] OR Ixazomib[tiab] OR Lenvatinib[tiab] OR Nintedanib[tiab] OR Necitumumab[tiab] OR Rucaparib[tiab] OR Brigatinib[tiab] OR Tivozanib[tiab] OR Ribociclib[tiab] OR Abemaciclib[tiab] OR Neratinib[tiab] OR Enasidenib[tiab] OR Niraparib[tiab] OR Copanlisib[tiab] OR Anlotinib[tiab] OR Lorlatinib[tiab] OR Fruquintinib[tiab] OR Binimetinib[tiab] OR Encorafenib[tiab] OR Dacomitinib[tiab] OR Ivosidenib[tiab] OR Larotrectinib[tiab] OR Talazoparib[tiab] OR Duvelisib[tiab] OR Entrectinib[tiab] OR Erdafitinib[tiab] OR Alpelisib[tiab] OR "Enfortumab vedotin"[tiab] OR "Trastuzumab deruxtecan"[tiab] OR Pemigatinib[tiab] OR Selumetinib[tiab] OR Capmatinib[tiab] OR Tepotinib[tiab] OR Tucatinib[tiab] OR Almonertinib[tiab] OR Selpercatinib[tiab] OR Pralsetinib[tiab] OR Neratinib[tiab] OR Brigatinib[tiab] OR Olaparib[tiab] OR Rucaparib[tiab] OR Erlotinib[tiab] OR "Belantamab mafodotin"[tiab] OR "Sacituzumab govitecan"[tiab] OR Naxitamab[tiab] OR Margetuximab[tiab] OR Tivozanib[tiab] OR Lorlatinib[tiab] OR Umbralisib[tiab] OR Tepotinib[tiab] OR Crizotinib[tiab] OR Carfilzomib[tiab] OR Asciminib[tiab] OR Abemaciclib[tiab] OR Ruxolitinib[tiab] OR Mobocertinib[tiab] OR Ivosidenib[tiab] OR Lenvatinib[tiab] OR Infigratinib[tiab] OR Sotorasib[tiab] OR Amivantamab[tiab] OR "Tisotumab vedotin"[tiab] OR Alpelisib[tiab] OR Tebentafusp[tiab])) AND (2020/1/1:2022/12/22[pdat])) NOT (animal*[tiab] OR review[ti] OR guideline*[ti] OR "meta analysis"[ti] OR phase[ti])) AND (2020/1/1:2022/12/22[pdat]))/22[pdat])) |

**Table A2 Full eligibility criteria for mapping review**

| **Inclusion criteria** | 1. English-language publications 2. Non-interventional (i.e. observational) studies using real-world data, including routinely collected data 3. Studies in clinical oncology 4. Studies focusing on solid tumours 5. Studies of TTs as part of a pharmacological intervention (alone or in association with any other types of treatments) when assessed for the main objective, even when used outside of approved indications 6. Articles published online over the last 3 years (2020-2022) 7. Studies with a main study objective including one of the following:    1. Effectiveness (cancer outcomes)    2. Biomarker exploration or validation with effectiveness analysis    3. Evaluation of prognostic or predictive factors (as stated by authors, including biomarkers, alone or within a risk score) with effectiveness results    4. Treatment strategies and/or sequence of treatments    5. Safety    6. PROs    7. QoL    8. Multiple of the previously stated objectives |
| --- | --- |
| **Exclusion criteria** | 1. Publications that did not generate primary real-world data or explore existing sources of real-world data 2. Other publication types: guidelines, abstracts, editorials, letters, opinion pieces, reviews, clinical trials (phase I to III), interventional prospective studies, study protocols, *in vitro* and preclinical studies, case reports, case series with <100 patients 3. Studies of haematological malignancies and non-malignant conditions 4. Studies evaluating checkpoint inhibitors or chemotherapy alone or other types of treatments not associated with TT, or studies not evaluating the TT for the primary outcome analysis (if primary outcome is clear) 5. Studies with a main objective related to treatment patterns only with no effectiveness results, economic burden or cost effectiveness, compliance alone or purely descriptive studies 6. Studies with <100 patients overall (if the study assessed patients) |

PRO, patient-reported outcome; QoL, quality of life; TT, targeted therapy.

**Table A3 Standardised extraction form**

| **General information** | |
| --- | --- |
| Title of the publication |  |
| Name of the corresponding author |  |
| Affiliation of the corresponding author |  |
| Email of the corresponding author |  |
| Month, year of publication online on PubMed (MM/YYYY) |  |
| **Journal information** | |
| Name of the journal |  |
| Journal IF (most recent IF available) |  |
| Type of journal | 1. General medical journal (oncology or organ specific)  2. Specialist medical journal  3. Non-medical journal (statistics, epidemiology and others)  4. Other |
| **Study characteristics** | |
| Population involved | 1. Adult  2. Children  3. Both  4. Not reported |
| Region in which the study was conducted ([https://unstats.un.org/unsd/methodology/m49/](about:blank)) | 1. Northern America  2. Latin America and the Caribbean  3. Africa  4. Europe  5. Asia  6. Oceania |
| Tumour type | 1. Breast  2. Lung  3. Genitourinary  4. GI  5. Gynaecological  6. Endocrine and neuroendocrine  7. Head and neck  8. Melanoma and other skin cancers  9. Sarcoma  10. Cancer of unknown primary site  11. Central nervous system  12. Other, specify |
| Type of intervention | 1. TT(s) evaluated alone as an intervention  2. TT(s) evaluated in combination with another anticancer treatment as an intervention  3. TT(s) evaluated in combination with another type of treatment as an intervention (non-anticancer) |
| Type of TT(s) | 1. Small molecules  2. Monoclonal antibodies  3. ADCs |
| Number of TTs involved (either the name of the molecule or the class of TT) | 1. One  2. Two  3. Multiple (>2)  If one or two, please write the full names: |
| Study design as reported by authors^a^ | 1. Prospective cohort study  2. Retrospective cohort study  3. Case-control study  4. Cross-sectional study  5. Phase IV study (including postmarketing surveillance study)  6. Quasi-experimental study (studies with geographical/historical comparator, before-and-after or interrupted time series)  8. Other  9. Not reported  If other, specify: |
| Comparative study (involving the TT(s) as an intervention)? | 1. Yes  2. No  3. Unclear |
| Source(s) of data | 1. Patient or disease registry(ies) (local or national)  2. Administrative or claims data  3. Population health survey  4. (Electronic) health records  5. Standardised data (CRF from a previous or ongoing cohort or PRO questionnaires)  6. Health care data aggregator (process of collecting data from multiple sources and compiling it into a single database)  7. Other  8. Not reported  If other, please specify |
| Study range | 1. National (single country)  2. International (>1 country)  3. Not reported |
| Is the study population based (stated by authors or as judged by reviewers)?^b^ | 1. Yes  2. No |
| Number of collaborative centres (number) | Number:  1. Not reported |
| Study objective(s) | 1. Effectiveness (all that is related to cancer outcomes)  2. Predictive or prognostic studies (as stated by authors, including biomarker and scores) WITH effectiveness analysis  3. Treatment strategies or sequence  4. PROs (including QoL)  5. Safety  6. The aim of the study is unclear but effectiveness results are reported for TT(s)  7. Other  If other, please specify |
| Cancer outcome(s) (if the aim is effectiveness or treatment strategies) | 1. Objective response rate (including disease control rate)  2. Time to treatment failure, time to treatment discontinuation or duration of treatment  3. Progression-free survival or disease-free survival  4. Overall survival  5. Other  If other, specify |
| Whole population analysed in the study | Total number of participants surveyed in the study:  1. The object of interest is not ‘patients’ (e.g. number of AEs) |
| TT(s) population | Number of participants treated with TT(s) (if there is more than one TT used, please give the sum of patients):  1. The object of interest is not ‘patients’ (e.g. number of AEs) |
| Study funding | 1. Academic  2. Governmental  3. Private not for profit (organisations or philanthropies)  4. Industry  5. Mix industry-based (industry and another type)  6. No funding  7. Unclear  8. Not reported |

ADC, antibody–drug conjugate; AE, adverse event; CRF, case report form; GI, gastrointestinal; IF, impact factor; PRO, patient-reported outcome; QoL, quality of life; TT, targeted therapy.

^a^Studies were considered retrospective when both data collection and analysis were retrospective. Similarly, studies were considered prospective when both data collection and analysis were prospective.

^b^Studies that assessed a population of patients who had ≥1 personal or environmental characteristics in common.

**Table A4 Study characteristics of all publications analysed**

| **Study characteristics** | ***n* (%)** |
| --- | --- |
| **Population**  Adults  Children  Both  Not reported | 1243 (99)  1 (<1)  3 (<1)  4 (<1) |
| **Tumour type**  GI^a^  Lung  Breast  Genitourinary^b^  Melanoma and other skin cancers  Head and neck  Gynaecological  Central nervous system  Endocrine and neuroendocrine  Sarcoma  >1 tumour type  Not reported | 377 (30)  281 (22)  265 (21)  111 (9)  50 (4)  37 (3)  35 (3)  21 (2)  16 (1)  7 (1)  46 (4)  5 (<1) |
| **Region of treatment**  Asia  Europe  North America  Oceania  Latin American and the Caribbeans  Africa  >1 region | 630 (50)  310 (25)  210 (17)  16 (1)  16 (1)  2 (<1)  67 (5) |
| **Type of TT**  Small molecule  Monoclonal antibody  ADC  >1 type | 774 (62)  378 (30)  10 (1)  89 (7) |
| **Type of intervention**  TT assessed alone  TT assessed in combination | 575 (46)  676 (54) |
| **Study design**  Retrospective cohort  Prospective cohort  Other (cross-sectional, case-control, postmarketing surveillance study, pharmacovigilance study)  Not reported | 1060 (85)  102 (8)  60 (5)  29 (2) |
| **Type of data source**  (Electronic) health records  Registry  Standardised data (CRF or other questionnaires)  Administrative or claims data  Health data aggregator  Population survey  >1 source  Not reported  Other | 682 (55)  141 (11)  106 (8)  60 (5)  40 (3)  4 (<1)  96 (8)  110 (9)  12 (1) |
| **Number of centres**  1  2-5  6-10  >10  Not reported | 513 (41)  146 (12)  67 (5)  237 (19)  288 (23) |
| **Population-based study**  Yes  No | 199 (16)  1052 (84) |
| **Comparative study**  Yes  No | 599 (48)  652 (52) |
| **Study range**  National  International (>1 country)  Not reported | 1143 (91)  104 (8)  4 (<1) |
| **Study objective(s)**  Effectiveness  Predictive or prognostic (with effectiveness analysis)  Safety  Treatment strategy  QoL and PROs  >1 objective | 308 (25)  149 (12)  142 (11)  13 (1)  7 (1)  632 (51) |
| **Type of funding**  No funding  Industry  Governmental  Private not for profit  Academic  >1 type of funding (industry-based)  >1 type of funding (non-industry based)  Not reported or unclear | 289 (23)  183 (15)  143 (11)  75 (6)  71 (6)  33 (3)  126 (10)  331 (26) |
| **Type of journal**  Specialist medical journal  General medical journal  Non-medical journal | 1077 (86)  107 (9)  67 (5) |
| **Outcomes for effectiveness**  At least one effectiveness outcome measured  OS measured  DFS, PFS or RFS measured | 1082 (86)  911 (84^c^)  705 (65^c^) |

ADC, antibody–drug conjugate; CRF, case report form; DFS, disease-free survival; GI, gastrointestinal; OS, overall survival; PFS, progression-free survival; PRO patient reported outcome; QoL, quality of life; RFS, recurrence-free survival; TT, targeted therapy.

^a^GI tumours included rectal, biliary, gastric, oesophageal, pancreatic, colorectal and anal cancer, and hepatocellular carcinoma.

^b^Genitourinary tumours included prostate, bladder, renal and penile cancer, and testicular seminoma and non-seminoma.

^c^Percentage based on the denominator of 1082 studies with at least one efficacy outcome measured.

**Figure A1 Univariable analysis assessing the association of study characteristics with IF.**


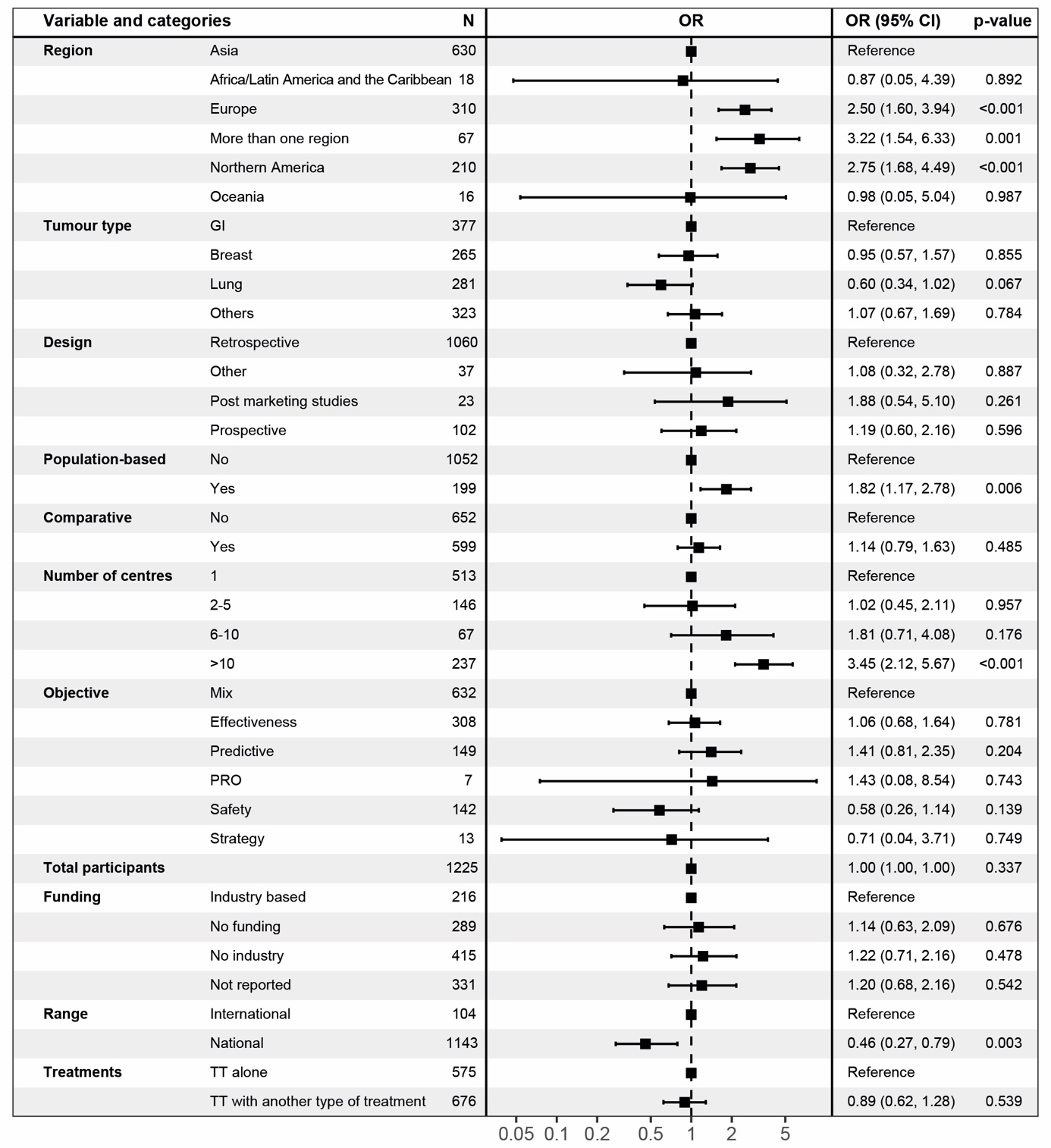


CI, confidence interval; GI, gastrointestinal; IF, impact factor; OR, odds ratio; PRO, patient-reported outcome; TT, targeted therapy.
