# Supplementary material for: Characteristics and impact of real-world evidence studies in oncology: comprehensive mapping review of publications evaluating targeted therapies in solid tumours
Source: ESMO Real World Data Digit Oncol. 2024 Dec 3;6:100091. doi: 10.1016/j.esmorw.2024.100091 (PMC12836563; doi:10.1016/j.esmorw.2024.100091)
Supplement: Supplementary Appendix B [file mmc2.docx]

Characteristics and impact of real-world evidence studies in oncology: comprehensive mapping review of publications evaluating targeted therapies in solid tumours

APPENDIX B – LIST OF INCLUDED PUBLICATIONS

| **Citation** | **Corresponding author** |
| --- | --- |
| Abdel-Qadir H, Bobrowski D, Zhou L, et al. Statin Exposure and Risk of Heart Failure After Anthracycline- or Trastuzumab-Based Chemotherapy for Early Breast Cancer: A Propensity Score‒Matched Cohort Study. *J Am Heart Assoc*. 2021 Jan 19;10(2):e018393. doi: 10.1161/JAHA.119.018393. | Husam Abdel-Qadir |
| Abdelrahim M, Victor D, Esmail A, et al. Transarterial Chemoembolization (TACE) Plus Sorafenib Compared to TACE Alone in Transplant Recipients with Hepatocellular Carcinoma: An Institution Experience. *Cancers (Basel).* 2022 Jan 27;14(3):650. doi: 10.3390/cancers14030650. | Maen Abdelrahim |
| Abdel-Rahman O, Mulder K, Easaw J. Outcomes of Ramucirumab Plus Paclitaxel Among Patients With Previously Treated Metastatic Gastric/Lower Esophageal Cancer: A Real-world Study*. Am J Clin Oncol*. 2021 Apr 1;44(4):158-161. doi: 10.1097/COC.0000000000000799. | Omar Abdel-Rahman |
| Abdel-Razeq H, Sharaf B, AlMasri R, et al. Thromboembolic Events in Patients with HER2-Negative, Hormone Receptor-Positive, Metastatic Breast Cancer Treated with Ribociclib Combined with Letrozole or Fulvestrant: A Real-World Data. *Cancer Manag Res*. 2022 Mar 8;14:1033-1041. doi: 10.2147/CMAR.S353584. | Hikmat Abdel-Razeq |
| Abu Rmilah AA, Lin G, Begna KH, et al. Risk of QTc prolongation among cancer patients treated with tyrosine kinase inhibitors. *Int J Cancer.* 2020 Dec 1;147(11):3160-3167. doi: 10.1002/ijc.33119. | Joerg Herrmann |
| Agema BC, Veerman GDM, Steendam CMJ, et al. Improving the tolerability of osimertinib by identifying its toxic limit. *Ther Adv Med Oncol.* 2022 Jun 3;14:17588359221103212. doi: 10.1177/17588359221103212. | Bram C. Agema |
| Aggarwal H, Punekar RS, Li L, et al. Quality of life analysis of patients treated with cetuximab or cisplatin for locoregionally advanced squamous cell carcinoma of head and neck in the United States. *Health Qual Life Outcomes.* 2020 Jun 22;18(1):195. doi: 10.1186/s12955-020-01424-x. | Himani Aggarwal |
| Aggarwal H, Sheffield KM, Li L, et al. Primary tumor location and survival in colorectal cancer: A retrospective cohort study. *World J Gastrointest Oncol*. 2020 Apr 15;12(4):405-423. doi: 10.4251/wjgo.v12.i4.405. | Himani Aggarwal |
| Aggarwal P, Goepfert RP, Garden AS, et al. Risk and Clinical Risk Factors Associated With Late Lower Cranial Neuropathy in Long-term Oropharyngeal Squamous Cell Carcinoma Survivors. *JAMA Otolaryngol Head Neck Surg.* 2021 May 1;147(5):469-478. doi: 10.1001/jamaoto.2020.5269. | Katherine A. Hutcheson |
| Agrawal C, Goyal P, Agarwal A, et al. Multicentric real world evidence with palbociclib in hormone positive HER2 negative metastatic breast cancer in Indian population. *Sci Rep*. 2021 Aug 10;11(1):16236. doi: 10.1038/s41598-021-95758-1. | Dinesh Chandra Doval |
| Agulnik JS, Kasymjanova G, Pepe C, et al. Real-World Pattern of Treatment and Clinical Outcomes of EGFR-Mutant Non-Small Cell Lung Cancer in a Single Academic Centre in Quebec. *Curr Oncol*. 2021 Dec 7;28(6):5179-5191. doi: 10.3390/curroncol28060434. | Goulnar Kasymjanova |
| Ahmad J, Thurlapati A, Thotamgari S, et al. Anti-cancer Drugs Associated Atrial Fibrillation-An Analysis of Real-World Pharmacovigilance Data. *Front Cardiovasc Med.* 2022 Apr 15;9:739044. doi: 10.3389/fcvm.2022.739044. | Paari Dominic |
| Akilli H, Rahatli S, Aliyeva K, et al. Survival in recurrent ovarian cancer patients before and after the bevacizumab era: an observational single-centre study. *J Obstet Gynaecol*. 2022 Aug;42(6):2230-2234. doi: 10.1080/01443615.2022.2036967. | Huseyin Akilli |
| Akli A, Girard N, Fallet V, et al. Histomolecular Resistance Mechanisms to First-Line Osimertinib in EGFR-Mutated Advanced Non-Small Cell Lung Cancer: A Multicentric Retrospective French Study. *Target Oncol.* 2022 Nov;17(6):675-682. doi: 10.1007/s11523-022-00915-9. | Etienne Giroux-Leprieur |
| Aktepe OH, Guven DC, Sahin TK, et al. The Predictive Value of Red Blood Cell Distribution Width for Survival Outcomes of Metastatic Renal Cell Carcinoma Patients Treated with Targeted Therapy. *Nutr Cancer*. 2021;73(10):1957-1963. doi: 10.1080/01635581.2021.1871925. | Oktay Halit Aktepe |
| Aktürk Esen S, Ergun Y, Erol C, et al. First-line treatment of patients with HER2-positive metastatic gastric and gastroesophageal junction cancer. *Bosn J Basic Med Sci*. 2022 Sep 16;22(5):818-825. doi: 10.17305/bjbms.2021.7069. | Selin Aktürk Esen |
| Alanen V, Iivanainen S, Arffman M, Koivunen JP. Purchase of prophylactic topical corticosteroids is associated with improved survival in NSCLCs treated with EGFR TKI: real-world cohort study. *Acta Oncol*. 2021 Sep;60(9):1100-1105. doi: 10.1080/0284186X.2021.1937309. | Jussi P. Koivunen |
| Alanen V, Iivanainen S, Arffman M, Koivunen JP. Tetracyclines increase the survival of NSCLC patients treated with EGFR TKIs: a retrospective nationwide registry study. *ESMO Open*. 2020 Oct;5(5):e000864. doi: 10.1136/esmoopen-2020-000864. | Jussi Pekka Koivunen |
| Al-Batran SE, Moorahrend E, Maintz C, et al. Clinical Practice Observation of Trastuzumab in Patients with Human Epidermal Growth Receptor 2-Positive Metastatic Adenocarcinoma of the Stomach or Gastroesophageal Junction. *Oncologist.* 2020 Aug;25(8):e1181-e1187. doi: 10.1634/theoncologist.2020-0109. | Salah-Eddin Al-Batran |
| Albiges L, Fléchon A, Chevreau C, et al. Real-world evidence of cabozantinib in patients with metastatic renal cell carcinoma: Results from the CABOREAL Early Access Program. *Eur J Cancer.* 2021 Jan;142:102-111. doi: 10.1016/j.ejca.2020.09.030. | Laurence Albiges |
| Albiges L, Heng DYC, Lee JL, et al. Impact of MET status on treatment outcomes in papillary renal cell carcinoma: A pooled analysis of historical data. *Eur J Cancer*. 2022 Jul;170:158-168. doi: 10.1016/j.ejca.2022.04.021. | Laurence Albiges |
| Aldaak M, Suliman HM, Abd-Elgadir EE, Abdoon IH. Impact of anticancer therapy on the quality of life of Sudanese patients with breast cancer at Khartoum oncology hospital. *BMC Womens Health.* 2022 Nov 14;22(1):448. doi: 10.1186/s12905-022-02041-0. | Iman Hassan Abdoon |
| Alghamdi MA, Amaro CP, Lee-Ying R, et al. Effect of sorafenib starting dose and dose intensity on survival in patients with hepatocellular carcinoma: Results from a Canadian Multicenter Database. *Cancer Med.* 2020 Jul;9(14):4918-4928. doi: 10.1002/cam4.3228. | Vincent C. Tam |
| Alhamar M, Alkamachi B, Mehrotra H, et al. Clinical significance of quantitative categorization of HER2 fluorescent in situ hybridization results in invasive breast cancer patients treated with HER2-targeted agents. *Mod Pathol*. 2021 Apr;34(4):720-734. doi: 10.1038/s41379-020-00728-z. | Dhananjay A. Chitale |
| AlHarbi A, Alshamrani M, Khan M, et al. Real-World Experience of Monitoring Practice of Endocrinopathies Associated with the Use of Novel Targeted Therapies among Patients with Solid Tumors. *Med Sci (Basel).* 2022 Nov 21;10(4):65. doi: 10.3390/medsci10040065. | Atika AlHarbi |
| Alholm Z, Monk BJ, Ting J, et al. Patient characteristics, treatment patterns, and clinical outcomes among patients with previously treated recurrent or metastatic cervical cancer: A community oncology-based analysis. *Gynecol Oncol*. 2021 May;161(2):422-428. doi: 10.1016/j.ygyno.2021.03.002. | Bradley J. Monk |
| Al-Juhaishi T, Deng X, Bandyopadhyay D, Paul A. The Role of Cytoreductive Nephrectomy and Targeted Therapy on Outcomes of Patients With Metastatic Sarcomatoid Renal Cell Carcinoma: A Population-Based Analysis. *Cureus*. 2022 May 27;14(5):e25395. doi: 10.7759/cureus.25395. | Asit Paul |
| Al-Sadawi M, Hussain Y, Copeland-Halperin RS, et al. Racial and Socioeconomic Disparities in Cardiotoxicity Among Women With HER2-Positive Breast Cancer. *Am J Cardiol*. 2021 May 15;147:116-121. doi: 10.1016/j.amjcard.2021.02.013. | Anthony F. Yu |
| Al-Saleh K, Abdel-Warith A, Alghamdi M, et al. Incidence of trastuzumab-induced cardiotoxicity and impact of body mass index in patients with breast cancer: Results from a Saudi tertiary cancer center. *Mol Clin Oncol.* 2022 Apr;16(4):78. doi: 10.3892/mco.2022.2511. | Nashwa Abdel-Aziz |
| Alvarez-Manceñido F, Jimenez-Fonseca P, Carmona-Bayonas A, et al. Is advanced esophageal adenocarcinoma a distinct entity from intestinal subtype gastric cancer? Data from the AGAMENON-SEOM Registry. *Gastric Cancer*. 2021 Jul;24(4):926-936. doi: 10.1007/s10120-021-01169-6. | Felipe Alvarez-Manceñido |
| Amadio G, Marchetti C, Villani ER, et al. ToleRability of BevacizUmab in elderly Ovarian cancer patients (TURBO study): a case-control study of a real-life experience. *J Gynecol Oncol.* 2020 Jan;31(1):e6. doi: 10.3802/jgo.2020.31.e6. | Claudia Marchetti |
| Amanuma M, Nagai H, Igarashi Y. Sorafenib Might Induce Sarcopenia in Patients With Hepatocellular Carcinoma by Inhibiting Carnitine Absorption. *Anticancer Res.* 2020 Jul;40(7):4173-4182. doi: 10.21873/anticanres.14417. | Hidenari Nagai |
| Amaro CP, Batra A, Lupichuk S. First-Line Treatment with a Cyclin-Dependent Kinase 4/6 Inhibitor Plus an Aromatase Inhibitor for Metastatic Breast Cancer in Alberta. *Curr Oncol.* 2021 Jun 18;28(3):2270-2280. doi: 10.3390/curroncol28030209. | Sasha Lupichuk |
| An SJ, Duchesneau ED, Strassle PD, et al. Pathologic complete response and survival after neoadjuvant chemotherapy in cT1-T2/N0 HER2+ breast cancer. *NPJ* *Breast Cancer.* 2022 May 12;8(1):65. doi: 10.1038/s41523-022-00433-x. | Philip M. Spanheimer |
| Ando R, Takahara K, Ito T, et al. Discontinuation of first-line molecular-targeted therapy and prognosis in patients with metastatic renal cell carcinoma: Impact of disease progression vs. adverse events. *Urol Oncol*. 2020 Dec;38(12):937.e19-937.e25. doi: 10.1016/j.urolonc.2020.06.023. | Ryosuke Ando |
| Ando Y, Kawaoka T, Suehiro Y, et al. Analysis of Post-Progression Survival in Patients with Unresectable Hepatocellular Carcinoma Treated with Lenvatinib. *Oncology*. 2020;98(11):787-797. doi: 10.1159/000509387. | Hiroshi Aikata |
| Anoop TM, Joseph PR, Abraham AA, et al. Cardiac toxicity of patients on short course trastuzumab in combination with chemotherapy (FinHer Protocol) in breast cancer. *Breast J.* 2021 Nov;27(11):811-816. doi: 10.1111/tbj.14289. | T. M. Anoop |
| Anoop TM, Joseph PR, Pn M, et al. Cutaneous Toxicities in Breast Cancer Patients Receiving Chemotherapy and Targeted Agents--An Observational Clinical Study. *Clin Breast Cancer.* 2021 Aug;21(4):e434-e447. doi: 10.1016/j.clbc.2021.01.009. | T. M. Anoop |
| Anwaier A, Chen J, Zhou H, et al. Real-world data on the efficacy and safety of pazopanib in IMDC favorable- and intermediate-risk metastatic renal cell carcinoma: a multicenter retrospective cohort study of Chinese patients. *Transl Androl Urol.* 2022 May;11(5):694-709. doi: 10.21037/tau-22-312. | Dingwei Ye |
| Arend RC, O'Malley DM, Banerjee S, et al. Utilization of Poly(ADP-Ribose) Polymerase Inhibitors in Ovarian Cancer: A Retrospective Cohort Study of US Healthcare Claims Data. *Adv Ther*. 2022 Jan;39(1):328-345. doi: 10.1007/s12325-021-01959-5. | Rebecca C. Arend |
| Arigami T, Matsushita D, Okubo K, et al. Changes in Chemotherapeutic Strategies and Their Prognostic Impact in Patients With Advanced Gastric Cancer. *In Vivo.* 2022 Jan-Feb;36(1):409-415. doi: 10.21873/invivo.12718. | Takaaki Arigami |
| Arnold D, Eggers E, Uhlig J, et al. Treatment of Metastatic Colorectal Carcinoma with Bevacizumab in First-Line and beyond First Progression: The KORALLE Non-Interventional Cohort Study. *Oncol Res Treat*. 2022;45(10):576-587. doi: 10.1159/000525031. | Dirk Arnold |
| Atkins MB, Julian C, Secrest MH, et al. Real-world treatment patterns and overall survival in *BRAF*-mutant melanoma patients treated with immunotherapy or targeted therapy. *Future Oncol.* 2022 Jun;18(18):2233-2245. doi: 10.2217/fon-2021-1536. | Michael B. Atkins |
| Atkinson V, Sandhu S, Hospers G, et al. Dabrafenib plus trametinib is effective in the treatment of BRAF V600-mutated metastatic melanoma patients: analysis of patients from the dabrafenib plus trametinib Named Patient Program (DESCRIBE II). *Melanoma Res*. 2020 Jun;30(3):261-267. doi: 10.1097/CMR.0000000000000654. | Victoria Atkinson |
| Auvray M, Tougeron D, Auclin E, et al. Efficacy and Safety of Aflibercept in Combination With Chemotherapy Beyond Second-Line Therapy in Metastatic Colorectal Carcinoma Patients: An AGEO Multicenter Study. *Clin Colorectal Cancer.* 2020 Mar;19(1):39-47.e5. doi: 10.1016/j.clcc.2019.08.003. | Simon Pernot |
| Aye PS, Tin Tin S, McKeage MJ, et al. Development and validation of a predictive model for estimating EGFR mutation probabilities in patients with non-squamous non-small cell lung cancer in New Zealand. *BMC Cancer.* 2020 Jul 14;20(1):658. doi: 10.1186/s12885-020-07162-z. | Phyu Sin Aye |
| Bae SJ, Kim JH, Ahn SG, et al. Real-World Clinical Outcomes of Biosimilar Trastuzumab (CT-P6) in HER2-Positive Early-Stage and Metastatic Breast Cancer. *Front Oncol.* 2021 Jun 4;11:689587. doi: 10.3389/fonc.2021.689587. | Joon Jeong |
| Bahçeci A, Paydaş S, Ak N, et al. Efficacy and Safety of Trastuzumab Emtansine in Her2 Positive Metastatic Breast Cancer: Real-World Experience. *Cancer Invest*. 2021 Jul-Aug;39(6-7):473-481. doi: 10.1080/07357907.2021.1933011. | Aykut Bahçeci |
| Bahl A, Bhatia K, Choudhary P, et al. Palliative chemotherapy with or without cetuximab in recurrent or metastatic squamous cell carcinoma of the head and neck: Indian tertiary care retrospective analysis. *Head Neck.* 2020 May;42(5):955-962. doi: 10.1002/hed.26070. | Ankur Bahl |
| Bai S, Tian T, Pacheco JM, et al. Immune-related adverse event profile of combination treatment of PD-(L)1 checkpoint inhibitors and bevacizumab in non-small cell lung cancer patients: data from the FDA adverse event reporting system. *Transl Lung Cancer Res.* 2021 Jun;10(6):2614-2624. doi: 10.21037/tlcr-21-464. | Pingping Hu |
| Bajpai J, Abraham G, Saklani AP, et al. Demographics, Pattern of Care, and Outcome Analysis of Malignant Melanomas - Experience From a Tertiary Cancer Centre in India. *Front Oncol.* 2021 Sep 8;11:710585. doi: 10.3389/fonc.2021.710585. | Jyoti Bajpai |
| Bakouny Z, El Zarif T, Dudani S, et al. Upfront Cytoreductive Nephrectomy for Metastatic Renal Cell Carcinoma Treated with Immune Checkpoint Inhibitors or Targeted Therapy: An Observational Study from the International Metastatic Renal Cell Carcinoma Database Consortium. *Eur Urol.* 2023 Feb;83(2):145-151. doi: 10.1016/j.eururo.2022.10.004. | Toni K. Choueiri |
| Balawardena J, Skandarajah T, Rathnayake W, Joseph N. Breast Cancer Survival in Sri Lanka. *JCO Glob Oncol.* 2020 Apr;6:589-599. doi: 10.1200/JGO.20.00003. | Nuradh Joseph |
| Baldacci S, Besse B, Avrillon V, et al. Lorlatinib for advanced anaplastic lymphoma kinase-positive non-small cell lung cancer: Results of the IFCT-1803 LORLATU cohort. *Eur J Cancer.* 2022 May;166:51-59. doi: 10.1016/j.ejca.2022.01.018. | Nicolas Girard |
| Bang Y, Yoo C, Lonardi S, et al. Sequential Treatment of Sorafenib-Regorafenib Versus Sorafenib-Physician's Choice: A Propensity Score-Matched Analysis. *Target Oncol.* 2021 May;16(3):401-410. doi: 10.1007/s11523-021-00797-3. | Andrea Casadei-Gardini |
| Bang YH, Hong YS, Lee JS, et al. Effectiveness of Combining Bevacizumab With First-Line Chemotherapy Regimens for Metastatic Colorectal Cancer in Real-World Practice. *Clin Colorectal Cancer.* 2021 Jun;20(2):101-112.e6. doi: 10.1016/j.clcc.2020.10.001. | Tae Won Kim |
| Bang YH, Kim JE, Lee JS, et al. Bevacizumab plus capecitabine as later-line treatment for patients with metastatic colorectal cancer refractory to irinotecan, oxaliplatin, and fluoropyrimidines. *Sci Rep.* 2021 Mar 29;11(1):7118. doi: 10.1038/s41598-021-86482-x. | Yong Sang Hong |
| Bang YH, Lee CK, Yoo C, et al. Real-world efficacy and safety of cabozantinib in Korean patients with advanced hepatocellular carcinoma: a multicenter retrospective analysis. *Ther Adv Med Oncol*. 2022 May 14;14:17588359221097934. doi: 10.1177/17588359221097934. | Baek-Yeol Ryoo |
| Bao J, Wu Z, Zhang C, et al. Efficacy and mechanism of osimertinib combined with bevacizumab in the treatment of postoperative EGFR positive stage II-IIIA lung adenocarcinoma. *Am J Transl Res.* 2022 Jan 15;14(1):633-642. | Jian Bao |
| Barat A, Smeets D, Moran B, et al. Combination of variations in inflammation- and endoplasmic reticulum-associated genes as putative biomarker for bevacizumab response in KRAS wild-type colorectal cancer. *Sci Rep.* 2020 Jun 17;10(1):9778. doi: 10.1038/s41598-020-65869-2. | Ana Barat |
| Barbieri MA, Sorbara EE, Cicala G, et al. Adverse Drug Reactions with HER2-Positive Breast Cancer Treatment: An Analysis from the Italian Pharmacovigilance Database. *Drugs Real World Outcomes.* 2022 Mar;9(1):91-107. doi: 10.1007/s40801-021-00278-z. | Edoardo Spina |
| Barbieri MA, Sorbara EE, Cicala G, et al. Safety profile of tyrosine kinase inhibitors used in non-small-cell lung cancer: An analysis from the Italian pharmacovigilance database. *Front Oncol.* 2022 Nov 23;12:1005626. doi: 10.3389/fonc.2022.1005626. | Maria Antonietta Barbieri |
| Basso U, Facchinetti A, Rossi E, et al. Prognostic Role of Circulating Tumor Cells in Metastatic Renal Cell Carcinoma: A Large, Multicenter, Prospective Trial. *Oncologist.* 2021 Sep;26(9):740-750. doi: 10.1002/onco.13842. | Umberto Basso |
| Bastos AGP, Carvalho B, Silva R, et al. Endoglin (CD105) and proliferation index in recurrent glioblastoma treated with anti-angiogenic therapy. *Front Oncol*. 2022 Sep 6;12:910196. doi: 10.3389/fonc.2022.910196. | Bruno Carvalho |
| Battisti NML, Andres MS, Lee KA, et al. Incidence of cardiotoxicity and validation of the Heart Failure Association-International Cardio-Oncology Society risk stratification tool in patients treated with trastuzumab for HER2-positive early breast cancer. *Breast Cancer Res Treat.* 2021 Jul;188(1):149-163. doi: 10.1007/s10549-021-06192-w. | Alistair Ring |
| Battisti NML, Rogerson F, Lee K, et al. Safety and efficacy of T-DM1 in patients with advanced HER2-positive breast cancer The Royal Marsden experience. *Cancer Treat Res Commun*. 2020;24:100188. doi: 10.1016/j.ctarc.2020.100188. | Alistair Ring |
| Battisti NML, True V, Chaabouni N, et al. Pathological complete response to neoadjuvant systemic therapy in 789 early and locally advanced breast cancer patients: The Royal Marsden experience. *Breast Cancer Res Treat.* 2020 Jan;179(1):101-111. doi: 10.1007/s10549-019-05444-0. | Alistair Ring |
| Beachler DC, de Luise C, Jamal-Allial A, et al. Real-world safety of palbociclib in breast cancer patients in the United States: a new user cohort study. *BMC Cancer*. 2021 Jan 25;21(1):97. doi: 10.1186/s12885-021-07790-z. | Daniel C. Beachler |
| Beca JM, Dai WF, Pataky RE, et al. Real-world Safety of Bevacizumab with First-line Combination Chemotherapy in Patients with Metastatic Colorectal Cancer: Population-based Retrospective Cohort Studies in Three Canadian Provinces. *Clin Oncol (R Coll Radiol).* 2022 Jan;34(1):e7-e17. doi: 10.1016/j.clon.2021.08.009. | J. M. Beca |
| Beige A, Ghiringhelli F, Lecuelle J, et al. Efficacy of Chemotherapy Plus Bevacizumab in Recurrent Glioblastoma Multiform: A Real-life Study. *Anticancer Res.* 2022 Dec;42(12):5847-5858. doi: 10.21873/anticanres.16093. | Alexandre Beige |
| Bekku K, Tsugawa T, Tsuboi K, et al. Molecular-targeted Therapy for Metastatic Renal Cell Carcinoma As First-line Therapy: A Single Institution 13-year Experience. *Acta Med Okayama*. 2022 Aug;76(4):465-472. doi: 10.18926/AMO/63906. | Kensuke Bekku |
| Benekli M, Gumus M, Ozkan M, et al. Tyrosine kinase inhibitors in the treatment of metastatic renal cell cancer patients with early cytokine intolerance: TURCOS, a Turkish national, prospective observational study. *J Oncol Pharm Pract.* 2021 Oct;27(7):1623-1630. doi: 10.1177/1078155220963535. | Mustafa Benekli |
| Beom SH, Bae KB, Zang DY, et al. Real-world experience of safety and effectiveness of regorafenib for treatment of metastatic colorectal cancer, advanced gastrointestinal stromal tumors, and hepatocellular carcinoma: a post-marketing surveillance study in Korea. *J Cancer*. 2022 Sep 21;13(13):3396-3403. doi: 10.7150/jca.74107. | Joong-Bae Ahn |
| Beom SH, Kim JG, Baik SH, et al. Safety and effectiveness of aflibercept in combination with FOLFIRI in Korean patients with metastatic colorectal cancer who received oxaliplatin-containing regimen. *J Cancer Res Clin Oncol.* 2023 Mar;149(3):1131-1143. doi: 10.1007/s00432-022-03946-x. | Jong Gwang Kim |
| Berg T, Jensen MB, Jakobsen EH, et al. Neoadjuvant chemotherapy and HER2 dual blockade including biosimilar trastuzumab (SB3) for HER2-positive early breast cancer: Population based real world data from the Danish Breast Cancer Group (DBCG). *Breast.* 2020 Dec;54:242-247. doi: 10.1016/j.breast.2020.10.014. | Tobias Berg |
| Bergen ES, Binter A, Starzer AM, et al. Favourable outcome of patients with breast cancer brain metastases treated with dual HER2 blockade of trastuzumab and pertuzumab. *Ther Adv Med Oncol.* 2021 Apr 22;13:17588359211009002. doi: 10.1177/17588359211009002. | Anna Sophie Berghoff |
| Berger A, Bernstein K, Alzate JD, et al. Significant survival improvements for patients with melanoma brain metastases: can we reach cure in the current era? *J Neurooncol*. 2022 Jul;158(3):471-480. doi: 10.1007/s11060-022-04036-1. | Assaf Berger |
| Bergqvist M, Christensen HN, Wiklund F, Bergström S. Real world utilization of EGFR TKIs and prognostic factors for survival in NSCLC during 2010-2016 in Sweden: A nationwide observational study. *Int J Cancer*. 2020 May 1;146(9):2510-2517. doi: 10.1002/ijc.32596. | Michael Bergqvist |
| Bernabé-Caro R, Garrido P, García-Campelo R, et al. Alectinib after failure to crizotinib in patients with ALK-positive non-small cell lung cancer: results from the Spanish early access program. *Oncotarget*. 2022 Jun 15;13:812-827. doi: 10.18632/oncotarget.28244. | Reyes Bernabé-Caro |
| Bersanelli M, Iacovelli R, Buti S, et al. Metastatic Renal Cell Carcinoma Rapidly Progressive to Sunitinib: What to Do Next? *Eur Urol Oncol*. 2021 Apr;4(2):274-281. doi: 10.1016/j.euo.2019.06.018. | Melissa Bersanelli |
| Berton D, Floquet A, Lescaut W, et al. Real-World Experience of Bevacizumab as First-Line Treatment for Ovarian Cancer: The GINECO ENCOURAGE Cohort of 468 French Patients. Front Pharmacol. 2021 Sep 20;12:711813. doi: 10.3389/fphar.2021.711813. | Dominique Berton |
| Besiroglu M, Demir T, Shbair ATM, et al. Is the Duration of Temozolomide Predictive for Sequential Bevacizumab Treatment Responses in the Glioblastoma Multiforme Cancer Setting? *J Coll Physicians Surg Pak.* 2021 Aug;31(8):932-936. doi: 10.29271/jcpsp.2021.08.932. | Mehmet Besiroglu |
| Besiroglu M, Shbair AT, Yasin AI, et al. Systemic Inflammatory Markers for Prediction of Bevacizumab Benefit in Glioblastoma Multiforme. *J Coll Physicians Surg Pak.* 2021 Jan;31(1):39-44. doi: 10.29271/jcpsp.2021.01.39. | Tarik Demir |
| Bhargava P, Rathnasamy N, Shenoy R, et al. Clinical Profile and Outcome of Patients With Human Epidermal Growth Factor Receptor 2-Positive Breast Cancer With Brain Metastases: Real-World Experience. *JCO Glob Oncol*. 2022 Sep;8:e2200126. doi: 10.1200/GO.22.00126. | Sudeep Gupta |
| Bhindi B, Graham J, Wells JC, et al. Deferred Cytoreductive Nephrectomy in Patients with Newly Diagnosed Metastatic Renal Cell Carcinoma. *Eur Urol.* 2020 Oct;78(4):615-623. doi: 10.1016/j.eururo.2020.04.038. | Bimal Bhindi |
| Bilger G, Toffart AC, Darrason M, et al. Paclitaxel-bevacizumab combination in advanced non-squamous non-small-cell lung cancer (NSCLC): AVATAX, a retrospective multicentric study. *Ther Adv Med Oncol.* 2022 Jun 6;14:17588359221099399. doi: 10.1177/17588359221099399. | Geoffroy Bilger |
| Bilgin B, Sendur MAN, Yucel S, et al. Real-life comparison of the afatinib and first-generation tyrosine kinase inhibitors in nonsmall cell lung cancer harboring EGFR exon 19 deletion: a Turk Oncology Group (TOG) study. *J Cancer Res Clin Oncol*. 2021 Jul;147(7):2145-2152. doi: 10.1007/s00432-020-03501-6. | Burak Bilgin |
| Bilici A, Uysal M, Menekse S, et al. Real-Life Analysis of Efficacy and Safety of Everolimus Plus Exemestane in Hormone Receptor-Positive, Human Epidermal Growth Factor Receptor-2-Negative Metastatic Breast Cancer Patients: A Turkish Oncology Group (TOG) Study. *Cancer Invest*. 2022 Feb;40(2):199-209. doi: 10.1080/07357907.2021.2017952. | Ahmet Bilici |
| Bini M, Quesada S, Meeus P, et al. Real-World Data on Newly Diagnosed *BRCA*-Mutated High-Grade Epithelial Ovarian Cancers: The French National Multicenter ESME Database. *Cancers (Basel).* 2022 Aug 21;14(16):4040. doi: 10.3390/cancers14164040. | Clémence Romeo |
| Bittoni M, Yang JC, Shih JY, et al. Real-world insights into patients with advanced NSCLC and MET alterations. *Lung Cancer*. 2021 Sep;159:96-106. doi: 10.1016/j.lungcan.2021.06.015. | Marisa Bittoni |
| Björnsson HK, Sverrisdottir A, Björnsson ES. Dili is rare amongst patients without liver metastases receiving cancer treatment in Iceland: a population-based cohort study. *Scand J Gastroenterol.* 2022 Jul;57(7):856-861. doi: 10.1080/00365521.2022.2038260. | Helgi K. Björnsson |
| Blondeaux E, Ferreira AR, Poggio F, et al. Clinical outcomes of patients with breast cancer relapsing after (neo)adjuvant trastuzumab and receiving trastuzumab rechallenge or lapatinib-based therapy: a multicentre retrospective cohort study. *ESMO Open*. 2020 Aug;5(4):e000719. doi: 10.1136/esmoopen-2020-000719. | Lucia Del Mastro |
| Bobrowski D, Suntheralingam S, Calvillo-Argüelles O, et al. The Yield of Routine Cardiac Imaging in Breast Cancer Patients Receiving Trastuzumab-Based Treatment: A Retrospective Cohort Study. *Can J Cardiol.* 2020 Oct;36(10):1658-1666. doi: 10.1016/j.cjca.2019.12.021. | Husam Abdel-Qadir |
| Bochiș OV, Vlad C, Căinap C, et al. Treatment beyond progression in metastatic colorectal cancer: to double or not to double the dose of bevacizumab? *J BUON.* 2020 Mar-Apr;25(2):875-883. | Cătălin Vlad |
| Boegemann M, Goebell PJ, Woike M, et al. Assessment of prognosis by established prognosis scores and physicians' judgement in mRCC patients: an analysis of the STAR-TOR registry. *Transl Androl Urol*. 2021 Oct;10(10):4062-4074. doi: 10.21037/tau-20-938. | Martin Boegemann |
| Boegemann M, Schlack K, Rink M, et al. Effect of comorbidities/comedications on sunitinib outcomes for metastatic renal cell carcinoma: the STAR-TOR registry. *Future Oncol*. 2020 Dec;16(35):2939-2948. doi: 10.2217/fon-2020-0548. | Martin Boegemann |
| Boekel NB, Duane FK, Jacobse JN, et al. Heart failure after treatment for breast cancer. *Eur J Heart Fail. 2020* Feb;22(2):366-374. doi: 10.1002/ejhf.1620. | Flora E. van Leeuwen |
| Boer FL, Ten Eikelder MLG, van Geloven N, et al. Evaluation of treatment, prognostic factors, and survival in 198 vulvar melanoma patients: Implications for clinical practice. *Gynecol Oncol.* 2021 Apr;161(1):202-210. doi: 10.1016/j.ygyno.2021.01.018. | Florine L. Boer |
| Bolzacchini E, Pinotti G, Bertù L, et al. On-target Toxicities Predictive of Survival in Metastatic Renal Cell Carcinoma (mRCC) Treated With Sunitinib: A Multicenter Retrospective Study. *Clin Genitourin Cancer*. 2020 Apr;18(2):e145-e156. doi: 10.1016/j.clgc.2019.10.003. | Elena Bolzacchini |
| Bon G, Pizzuti L, Laquintana V, et al. Loss of HER2 and decreased T-DM1 efficacy in HER2 positive advanced breast cancer treated with dual HER2 blockade: the SePHER Study. *J Exp Clin Cancer Res.* 2020 Dec 10;39(1):279. doi: 10.1186/s13046-020-01797-3. | Giulia Bon |
| Bonafede MM, Korytowsky B, Singh P, et al. Treatment Patterns and Economic Burden by Lines of Therapy Among Patients with Advanced Hepatocellular Carcinoma Treated with Systemic Cancer Therapy. *J Gastrointest Cancer*. 2020 Mar;51(1):217-226. doi: 10.1007/s12029-019-00230-z. | Machaon M. Bonafede |
| Bonanno L, Pavan A, Ferro A, et al. Clinical Impact of Plasma and Tissue Next-Generation Sequencing in Advanced Non-Small Cell Lung Cancer: A Real-World Experience. *Oncologist.* 2020 Dec;25(12):e1996-e2005. doi: 10.1634/theoncologist.2020-0148. | Laura Bonanno |
| Boosman RJ, Jebbink M, Veldhuis WB, et al. Exposure-Response Analysis of Osimertinib in EGFR Mutation Positive Non-Small Cell Lung Cancer Patients in a Real-Life Setting. *Pharm Res.* 2022 Oct;39(10):2507-2514. doi: 10.1007/s11095-022-03355-2. | René J. Boosman |
| Botticelli A, Pomati G, Cirillo A, et al. Weekly chemotherapy as first line treatment in frail head and neck cancer patients in the immunotherapy era. *J Transl Med.* 2021 Jul 12;19(1):303. doi: 10.1186/s12967-021-02975-3. | Alessio Cirillo |
| Bouwer NI, Steenbruggen TG, van Rosmalen J, et al. Cardiotoxicity during long-term trastuzumab use in patients with HER2-positive metastatic breast cancer: who needs cardiac monitoring? *Breast Cancer Res Treat.* 2021 Apr;186(3):851-862. doi: 10.1007/s10549-020-06039-w. | N. I. Bouwer |
| Brat K, Bratova M, Skrickova J, et al. Real-life effectiveness of first-line anticancer treatments in stage IIIB/IV NSCLC patients: Data from the Czech TULUNG Registry. *Thorac Cancer.* 2020 Nov;11(11):3346-3356. doi: 10.1111/1759-7714.13679. | Monika Bratova |
| Britschgi C, Addeo A, Rechsteiner M, et al. Real-World Treatment Patterns and Survival Outcome in Advanced Anaplastic Lymphoma Kinase (ALK) Rearranged Non-Small-Cell Lung Cancer Patients. *Front Oncol*. 2020 Aug 21;10:1299. doi: 10.3389/fonc.2020.01299. | Alessandra Curioni-Fontecedro |
| Brose MS, Smit JWA, Lin CC, et al. Multikinase Inhibitors for the Treatment of Asymptomatic Radioactive Iodine-Refractory Differentiated Thyroid Cancer: Global Noninterventional Study (RIFTOS MKI). *Thyroid*. 2022 Sep;32(9):1059-1068. doi: 10.1089/thy.2022.0061. | Marcia S. Brose |
| Brückl WM, Reck M, Griesinger F, et al. Afatinib as first-line treatment in patients with EGFR-mutated non-small cell lung cancer in routine clinical practice. *Ther Adv Med Oncol*. 2021 May 6;13:17588359211012361. doi: 10.1177/17588359211012361. | Wolfgang M. Brückl |
| Brueckl WM, Reck M, Schäfer H, et al. Older patients with EGFR mutation-positive non-small cell lung cancer treated with afatinib in clinical practice: A subset analysis of the non-interventional GIDEON study. *J Geriatr Oncol.* 2023 Jan;14(1):101394. doi: 10.1016/j.jgo.2022.10.009. | Wolfgang M. Brueckl |
| Brufsky A, Liu X, Li B, et al. Real-World Effectiveness of Palbociclib Plus Letrozole vs Letrozole Alone for Metastatic Breast Cancer With Lung or Liver Metastases: Flatiron Database Analysis. *Front Oncol.* 2022 Jul 4;12:865292. doi: 10.3389/fonc.2022.865292. | Adam Brufsky |
| Brufsky A, Liu X, Li B, et al. Real-World Tumor Response of Palbociclib Plus Letrozole Versus Letrozole for Metastatic Breast Cancer in US Clinical Practice. *Target Oncol.* 2021 Sep;16(5):601-611. doi: 10.1007/s11523-021-00826-1. | Adam Brufsky |
| Brzozowska M, Wierzba W, Szafraniec-Buryło S, et al. Overall survival of patients with EGFR mutation-positive non-small-cell lung cancer treated with erlotinib, gefitinib or afatinib under drug programmes in Poland - real-world data. *Arch Med Sci*. 2019 Jan 22;17(6):1618-1627. doi: 10.5114/aoms.2018.80362. | Gabriela Majkut |
| Buchler T, Kiss I, Hornova J, et al. Sequential Treatment with Bevacizumab and Aflibercept for Metastatic Colorectal Cancer in Real-World Clinical Practice. *Target Oncol.* 2020 Apr;15(2):193-201. doi: 10.1007/s11523-020-00705-1. | Tomas Buchler |
| Burgio V, Iavarone M, Di Costanzo GG, et al. Real-Life Clinical Data of Lenvatinib versus Sorafenib for Unresectable Hepatocellular Carcinoma in Italy. *Cancer Manag Res*. 2021 Dec 24;13:9379-9389. doi: 10.2147/CMAR.S330195. | Andrea Casadei-Gardini |
| Buti S, Tommasi C, Scartabellati G, et al. The impact of proton-pump inhibitors administered with tyrosine kinase inhibitors in patients with metastatic renal cell carcinoma. *Anticancer Drugs*. 2023 Jan 1;34(1):178-186. doi: 10.1097/CAD.0000000000001356. | Chiara Tommasi |
| Cacho-Díaz B, Cuapantécatl LD, Garcilazo-Reyes YJ, et al. Targeted Therapies and Utility of the Lung-molGPA in Non-Small-Cell Lung Cancer Patients with Brain Metastases. *Oncology.* 2022;100(10):542-554. doi: 10.1159/000525082. | Oscar Arrieta |
| Căinap C, Bochiş OV, Vlad C, et al. Doubling the Dose of Bevacizumab Beyond Progression in Metastatic Colorectal Cancer-the Experience of a Tertiary Cancer Center. *Front Pharmacol.* 2021 Mar 11;12:487316. doi: 10.3389/fphar.2021.487316. | Ovidiu Crişan |
| Cainap C, Ungur RA, Bochis OV, et al. Partnering bevacizumab with irinotecan as first line-therapy of metastatic colorectal cancer improves progression free survival-A retrospective analysis. *PLoS One*. 2021 Apr 28;16(4):e0248922. doi: 10.1371/journal.pone.0248922. | Rodica Ana Ungur |
| Cakan B, Acikgoz O, Bilici A, et al. Prognostic significance of primary tumor localization in patients with metastatic colorectal cancer: Is it beneficial to select targeted treatment? Real-life experience from Turkey. *J BUON*. 2021 Sep-Oct;26(5):1908-1917. | Ahmet Bilici |
| Camejo N, Castillo C, Alonso R, et al. Effectiveness of Trastuzumab for Human Epidermal Growth Factor Receptor 2-Positive Breast Cancer in a Real-Life Setting: One Decade of Experience Under National Treatment Coverage Regulations. *JCO Glob Oncol.* 2020 Feb;6:217-223. doi: 10.1200/JGO.19.00299. | Natalia Camejo |
| Cancel M, Fromont G, Blonz C, et al. Everolimus or sunitinib as first-line treatment of metastatic papillary renal cell carcinoma: A retrospective study of the GETUG group (Groupe d'Etude des Tumeurs Uro-Génitales). *Eur J Cancer.* 2021 Oct 4;158:1-11. doi: 10.1016/j.ejca.2021.08.046. | Claude Linassier |
| Cantini L, Pistelli M, Merloni F, et al. Body Mass Index and Hormone Receptor Status Influence Recurrence Risk in HER2-Positive Early Breast Cancer Patients. Clin Breast Cancer. 2020 Feb;20(1):e89-e98. doi: 10.1016/j.clbc.2019.06.008. | Rossana Berardi |
| Cao C, Shou J, Shi H, et al. Novel cut-off values of time from diagnosis to systematic therapy predict the overall survival and the efficacy of targeted therapy in renal cell carcinoma: A long-term, follow-up, retrospective study. *Int J Urol*. 2022 Mar;29(3):212-220. doi: 10.1111/iju.14751. | Jianzhong Shou |
| Cao L, Shenk R, Stabellini N, et al. Adjuvant trastuzumab with or without chemotherapy in stage 1 pT1N0 HER2+ breast cancer: a National Cancer Database analysis. *Breast Cancer Res Treat.* 2022 Jan;191(1):169-176. doi: 10.1007/s10549-021-06411-4. | Alberto J. Montero |
| Cao L, Towe CW, Shenk R, et al. A comparison of local therapy alone with local plus systemic therapy for stage I pT1aN0M0 HER2+ breast cancer: A National Cancer Database analysis. *Cancer.* 2022 Jul 1;128(13):2433-2440. doi: 10.1002/cncr.34200. | Alberto J. Montero |
| Cao X, Tang D, Ratto B, et al. Real-world Clinical Outcomes of Pazopanib Immediately After Discontinuation of Immunotherapy for Advanced Renal Cell Carcinoma. *Clin Genitourin Cancer.* 2020 Feb;18(1):e37-e45. doi: 10.1016/j.clgc.2019.10.010. | Xiting Cao |
| Caputo F, Dadduzio V, Tovoli F, et al. The role of PNI to predict survival in advanced hepatocellular carcinoma treated with Sorafenib. *PLoS One*. 2020 May 7;15(5):e0232449. doi: 10.1371/journal.pone.0232449. | Andrea Casadei-Gardini |
| Carausu M, Carton M, Diéras V, et al. Association of Endocrine Therapy for HR+/ERBB2+ Metastatic Breast Cancer With Survival Outcomes. *JAMA Netw Open.* 2022 Dec 1;5(12):e2247154. doi: 10.1001/jamanetworkopen.2022.47154. | Luc Cabel |
| Carballo-Folgoso L, Álvarez-Velasco R, Lorca R, et al. Evaluation of cardiovascular events in patients with hepatocellular carcinoma treated with sorafenib in the clinical practice. The CARDIO-SOR study. *Liver Int.* 2021 Sep;41(9):2200-2211. doi: 10.1111/liv.14941. | Rebeca Lorca |
| Carbasse C, Leenhardt F, Jacot W, et al. Oral targeted therapy dose adaptation in older patients with cancer: A real-life French cohort. *Br J Clin Pharmacol*. 2022 Jul;88(7):3370-3377. doi: 10.1111/bcp.15285. | Clément Carbasse |
| Cardoso Borges F, Alves da Costa F, Ramos A, et al. Real-world effectiveness of palbociclib plus fulvestrant in advanced breast cancer: Results from a population-based cohort study. *Breast.* 2022 Apr;62:135-143. doi: 10.1016/j.breast.2022.02.005. | Fábio Cardoso Borges |
| Carlino F, Diana A, Ventriglia A, et al. HER2-Low Status Does Not Affect Survival Outcomes of Patients with Metastatic Breast Cancer (MBC) Undergoing First-Line Treatment with Endocrine Therapy plus Palbociclib: Results of a Multicenter, Retrospective Cohort Study. *Cancers (Basel).* 2022 Oct 11;14(20):4981. doi: 10.3390/cancers14204981. | Francesca Carlino |
| Carvalho B, Lopes RG, Linhares P, Costa A, Caeiro C, Fernandes AC, Tavares N, Osório L, Vaz R. Hypertension and proteinuria as clinical biomarkers of response to bevacizumab in glioblastoma patients. *J Neurooncol.* 2020 Mar;147(1):109-116. doi: 10.1007/s11060-020-03404-z. | Bruno Carvalho |
| Casadei-Gardini A, Dadduzio V, Rovesti G, et al. Utility of neutrophil-to-lymphocyte ratio to identify long-term survivors among HCC patients treated with sorafenib. *Medicine (Baltimore).* 2020 May 29;99(22):e19958. doi: 10.1097/MD.0000000000019958. | Andrea Casadei-Gardini |
| Casadei-Gardini A, Marisi G, Dadduzio V, et al. Association of NOS3 and ANGPT2 Gene Polymorphisms with Survival in Patients with Hepatocellular Carcinoma Receiving Sorafenib: Results of the Multicenter Prospective INNOVATE Study. *Clin Cancer Res*. 2020 Sep 1;26(17):4485-4493. doi: 10.1158/1078-0432.CCR-19-3897. | Giorgia Marisi |
| Casadei-Gardini A, Rimassa L, Rimini M, et al. Regorafenib versus cabozantinb as second-line treatment after sorafenib for unresectable hepatocellular carcinoma: matching-adjusted indirect comparison analysis. *J Cancer Res Clin Oncol*. 2021 Dec;147(12):3665-3671. doi: 10.1007/s00432-021-03602-w. | Andrea Casadei-Gardini |
| Casadei-Gardini A, Rimini M, Tada T, et al. Atezolizumab plus bevacizumab versus lenvatinib for unresectable hepatocellular carcinoma: a large real-life worldwide population. *Eur J Cancer.* 2023 Feb;180:9-20. doi: 10.1016/j.ejca.2022.11.017. | Andrea Casadei-Gardini |
| Casadei-Gardini A, Rovesti G, Dadduzio V, et al. Impact of Aspirin on clinical outcome in advanced HCC patients receiving sorafenib and regorafenib. *HPB (Oxford).* 2021 Jun;23(6):915-920. doi: 10.1016/j.hpb.2020.09.024. | Andrea Casadei-Gardini |
| Castillejo Becerra CM, Smith WM, Dalvin LA. Ophthalmic adverse effects of BRAF inhibitors. *Eur J Ophthalmol*. 2022 Oct 11:11206721221132872. doi: 10.1177/11206721221132872. | Lauren A. Dalvin |
| Castillo C, Camejo N, Etcheverria C, et al. Trastuzumab-induced cardiotoxicity in early breast cancer over a 10-year period in Uruguay. *Medicine (Baltimore).* 2022 Jul 29;101(30):e29927. doi: 10.1097/MD.0000000000029927. | Cecilia Castillo |
| Catalano V, Bergamo F, Cremolini C, et al. Clinical impact of first-line bevacizumab plus chemotherapy in metastatic colorectal cancer of mucinous histology: a multicenter, retrospective analysis on 685 patients. *J Cancer Res Clin Oncol.* 2020 Feb;146(2):493-501. doi: 10.1007/s00432-019-03077-w. | Vincenzo Catalano |
| Cecere SC, Giannone G, Salutari V, et al. Olaparib as maintenance therapy in patients with BRCA 1-2 mutated recurrent platinum sensitive ovarian cancer: Real world data and post progression outcome. *Gynecol Oncol*. 2020 Jan;156(1):38-44. doi: 10.1016/j.ygyno.2019.10.023. | Sandro Pignata |
| Celik A, Berg T, Nielsen LB, et al. First-Line Treatment of HER2-Positive Metastatic Breast Cancer With Dual Blockade Including Biosimilar Trastuzumab (SB3): Population-Based Real-World Data From the DBCG. *Breast Cancer (Auckl).* 2022 Mar 24;16:11782234221086992. doi: 10.1177/11782234221086992. | Alan Celik |
| Cha C, Ahn SG, Kim D, et al. Axillary response according to neoadjuvant single or dual human epidermal growth factor receptor 2 (HER2) blockade in clinically node-positive, HER2-positive breast cancer. *Int J Cancer*. 2021 Oct 15;149(8):1585-1592. doi: 10.1002/ijc.33726. | Joon Jeong |
| Chainitikun S, Long JP, Rodriguez-Bautista R, et al. The efficacy of first-line chemotherapy in endocrine-resistant hormone receptor-positive (HR+), human epidermal growth factor receptor 2-negative (HER2-) metastatic breast cancer. *Breast Cancer Res Treat.* 2020 Oct;183(3):729-739. doi: 10.1007/s10549-020-05837-6. | Naoto T. Ueno |
| Chakiryan NH, Acevedo AM, Garzotto MA, et al. Survival outcomes and practice trends for off-label use of adjuvant targeted therapy in high-risk locoregional renal cell carcinoma. *Urol Oncol*. 2020 Jun;38(6):604.e1-604.e7. doi: 10.1016/j.urolonc.2020.02.028. | Nicholas H. Chakiryan |
| Chakiryan NH, Jiang DD, Gillis KA, et al. Real-World Survival Outcomes Associated With First-Line Immunotherapy, Targeted Therapy, and Combination Therapy for Metastatic Clear Cell Renal Cell Carcinoma. *JAMA Netw Open*. 2021 May 3;4(5):e2111329. doi: 10.1001/jamanetworkopen.2021.11329. | Nicholas H. Chakiryan |
| Chakravarthy VB, Schachner B, Amin AG, et al. The Impact of Targetable Mutations on Clinical Outcomes of Metastatic Epidural Spinal Cord Compression in Patients With Non-Small-Cell Lung Cancer Treated With Hybrid Therapy (Surgery Followed by Stereotactic Body Radiation Therapy). *Neurosurgery.* 2023 Mar 1;92(3):557-564. doi: 10.1227/neu.0000000000002247. | Ori Barzilai |
| Chan OSH, Lee VHF, Nyaw SF, et al. Can Quantitative Measures of T790M Allelic Fraction Predict Survival Outcomes in Patients Receiving Osimertinib? Observations From an Early Access Programme. *Clin Oncol (R Coll Radiol).* 2021 Jul;33(7):e305-e314. doi: 10.1016/j.clon.2021.01.011. | O. S. H. Chan |
| Chang JW, Huang CY, Fang YF, et al. Epidermal growth factor receptor tyrosine kinase inhibitors for non-small cell lung cancer harboring uncommon EGFR mutations: Real-world data from Taiwan. *Thorac Cancer.* 2023 Jan;14(1):12-23. doi: 10.1111/1759-7714.14537. | Chiao-En Wu |
| Chang JW, Huang CY, Fang YF, et al. Risk Stratification Using a Novel Nomogram for 2190 EGFR-Mutant NSCLC Patients Receiving the First or Second Generation EGFR-TKI. *Cancers (Basel).* 2022 Feb 15;14(4):977. doi: 10.3390/cancers14040977. | Chiao-En Wu |
| Chang M, Dalvin LA, Mazloumi M, et al. Prophylactic Intravitreal Bevacizumab After Plaque Radiotherapy for Uveal Melanoma: Analysis of Visual Acuity, Tumor Response, and Radiation Complications in 1131 Eyes Based on Patient Age. *Asia Pac J Ophthalmol (Phila).* 2020 Jan-Feb;9(1):29-38. doi: 10.1097/APO.0000000000000271. | Carol L. Shields |
| Chang N, Duan J, Wang L, et al. Patients with advanced non-small cell lung cancer with EGFR mutations in addition to complex mutations treated with osimertinib have a poor clinical outcome: A real-world data analysis. *Oncol Lett.* 2020 Sep;20(3):2266-2272. doi: 10.3892/ol.2020.11801. | Zhefeng Liu |
| Chang Q, Qiang H, Qian J, et al. Epidermal Growth Factor Receptor Mutation Status and Response to Tyrosine Kinase Inhibitors in Advanced Chinese Female Lung Squamous Cell Carcinoma: A Retrospective Study. *Front Oncol.* 2021 Apr 2;11:652560. doi: 10.3389/fonc.2021.652560. | Tianqing Chu |
| Chang WI, Kim BH, Kim YJ, et al. Role of radiotherapy in Barcelona Clinic Liver Cancer stage C hepatocellular carcinoma treated with sorafenib. *J Gastroenterol Hepatol.* 2022 Feb;37(2):387-394. doi: 10.1111/jgh.15722. | Byoung Hyuck Kim |
| Chang WT, Chen PW, Lin HW, et al. Risks of trastuzumab-related cardiotoxicity in breast cancer patients in Taiwan. *ESC Heart Fail.* 2021 Dec;8(6):5149-5158. doi: 10.1002/ehf2.13591. | Yi-Heng Li |
| Chapin WJ, Hwang WT, Karasic TB, et al. Comparison of nivolumab and sorafenib for first systemic therapy in patients with hepatocellular carcinoma and Child-Pugh B cirrhosis. *Cancer Med.* 2023 Jan;12(1):189-199. doi: 10.1002/cam4.4906. | William J. Chapin |
| Chau I, Fakih M, García-Alfonso P, et al. Safety and Effectiveness of Aflibercept + Fluorouracil, Leucovorin, and Irinotecan (FOLFIRI) for the Treatment of Patients with Metastatic Colorectal Cancer (mCRC) in Current Clinical Practice: OZONE Study. *Cancers (Basel).* 2020 Mar 11;12(3):657. doi: 10.3390/cancers12030657. | Ian Chau |
| Chawla S, Hill A, Fearfield L, et al. Cutaneous toxicities occurring during palbociclib (CDK4/6 inhibitor) and endocrine therapy in patients with advanced breast cancer: a single-centre experience. *Breast Cancer Res Treat.* 2021 Jul;188(2):535-545. doi: 10.1007/s10549-021-06169-9. | Kara Heelan |
| Che YQ, Zhang Y, Ou KP, et al. Depth of Response and Early Tumor Shrinkage for Predicting Clinical Outcomes in HER2-Positive Metastatic Breast Cancer Treated with Trastuzumab. *Cancer Manag Res.* 2020 Sep 16;12:8527-8534. doi: 10.2147/CMAR.S269067. | Yang Luo |
| Chen B, Lei J, Zhao H, et al. Efficacy and Safety of TKI Plus PD-1 Inhibitors in Elderly uHCC Patients: A Retrospective Study. *J Hepatocell Carcinoma*. 2022 Nov 8;9:1171-1185. doi: 10.2147/JHC.S387254. | Jiamin Cheng |
| Chen B, Zheng D, Yu W, et al. Cetuximab versus bevacizumab maintenance following prior 8-cycle modified FOLFOXIRI plus cetuximab in Asian postmenopausal women with treatment-naive KRAS and BRAF wild-type metastatic colorectal cancer. *J Int Med Res.* 2020 Sep;48(9):300060520930440. doi: 10.1177/0300060520930440. | Jintao Zhuang |
| Chen C, Wu Y, Liu BL, et al. Whole-Brain Radiotherapy Can Improve the Survival of Patients with Multiple Brain Metastases from Non-Small Cell Lung Cancer Treated by Epidermal Growth Factor Receptor-Tyrosine Kinase Inhibitors. *Cancer Manag Res.* 2020 Nov 6;12:11333-11340. doi: 10.2147/CMAR.S279096. | Ju Ying Zhou |
| Chen CH, Chang JW, Chang CF, et al. Real-world Afatinib Outcomes in Advanced Non-small Cell Lung Cancer Harboring *EGFR* Mutations. *Anticancer Res*. 2022 Apr;42(4):2145-2157. doi: 10.21873/anticanres.15697. | Chiao-En Wu |
| Chen CT, Hsu CH, Cheng AL, Shao YY. Expanding Sorafenib Treatment for Hepatocellular Carcinoma Beyond Barcelona Clinic Liver Cancer Stage C Patients: A National Study. *Anticancer Res.* 2022 Sep;42(9):4461-4470. doi: 10.21873/anticanres.15946. | Yu-Yun Shao |
| Chen DY, Liu JR, Tseng CN, et al. Major Adverse Cardiovascular Events in Patients With Renal Cell Carcinoma Treated With Targeted Therapies. *JACC CardioOncol*. 2022 Jun 21;4(2):223-234. doi: 10.1016/j.jaccao.2022.05.002. | Wen-Kuan Huang |
| Chen H, Xue L, Liu L, Li P. Efficacy of apatinib combined with tegafur gimeracil and oteracil potassium in the second-line treatment of advanced gastric cancer. *J BUON*. 2021 May-Jun;26(3):917-923. | Pengfei Li |
| Chen HJ, Tu CY, Huang KY, et al. Early serum tumor marker levels after fourteen days of tyrosine kinase inhibitor targeted therapy predicts outcomes in patients with advanced lung adenocarcinoma. *PLoS One*. 2020 Dec 11;15(12):e0240736. doi: 10.1371/journal.pone.0240736. | Hung-Jen Chen |
| Chen HL, Chen Q, Deng YC. Pathologic complete response to neoadjuvant anti-HER2 therapy is associated with HER2 immunohistochemistry score in HER2-positive early breast cancer. *Medicine (Baltimore).* 2021 Nov 5;100(44):e27632. doi: 10.1097/MD.0000000000027632. | Yong-Chuan Deng |
| Chen J, Xiong P, Nie M, et al. The combination treatment strategy of lenvatinib for hepatocellular carcinoma: a real-world study. *J Cancer Res Clin Oncol.* 2023 Jun;149(6):2491-2500. doi: 10.1007/s00432-022-04082-2. | Li Xu |
| Chen K, Wei W, Liu L, et al. Lenvatinib with or without immune checkpoint inhibitors for patients with unresectable hepatocellular carcinoma in real-world clinical practice. *Cancer Immunol Immunother.* 2022 May;71(5):1063-1074. doi: 10.1007/s00262-021-03060-w. | Jian-Hong Zhong |
| Chen L, Ke Z, Xiong F, et al. Platelet-to-lymphocyte ratio predicts therapy outcomes of transarterial chemoembolization plus apatinib in the treatment of advanced hepatocellular carcinoma. *Anticancer Drugs.* 2020 Oct;31(9):966-972. doi: 10.1097/CAD.0000000000000913. | Chuansheng Zheng |
| Chen PY, Wang CC, Hsu CN, Chen CY. Association of EGFR Tyrosine Kinase Inhibitor Treatment With Progression-Free Survival Among Taiwanese Patients With Advanced Lung Adenocarcinoma and EGFR Mutation. *Front Pharmacol*. 2021 Aug 9;12:720687. doi: 10.3389/fphar.2021.720687. | Chung-Yu Chen |
| Chen Q, Li Y, Zhang W, et al. Safety and efficacy of ICI plus anlotinib vs. anlotinib alone as third-line treatment in extensive-stage small cell lung cancer: a retrospective study. *J Cancer Res Clin Oncol*. 2022 Feb;148(2):401-408. doi: 10.1007/s00432-021-03858-2. | Qisen Guo |
| Chen Q, Ouyang D, Anwar M, et al. Effectiveness and Safety of Pyrotinib, and Association of Biomarker With Progression-Free Survival in Patients With HER2-Positive Metastatic Breast Cancer: A Real-World, Multicentre Analysis. *Front Oncol.* 2020 May 25;10:811. doi: 10.3389/fonc.2020.00811. | Wenjun Yi |
| Chen S, Wu Z, Shi F, et al. Lenvatinib plus TACE with or without pembrolizumab for the treatment of initially unresectable hepatocellular carcinoma harbouring PD-L1 expression: a retrospective study. *J Cancer Res Clin Oncol*. 2022 Aug;148(8):2115-2125. doi: 10.1007/s00432-021-03767-4. | Bo Xu |
| Chen S, Xu B, Wu Z, et al. Pembrolizumab plus lenvatinib with or without hepatic arterial infusion chemotherapy in selected populations of patients with treatment-naive unresectable hepatocellular carcinoma exhibiting PD-L1 staining: a multicenter retrospective study. *BMC Cancer*. 2021 Oct 19;21(1):1126. doi: 10.1186/s12885-021-08858-6. | Wenbo Guo |
| Chen SC, Huang YH, Chen MH, et al. Anti-PD-1 combined sorafenib versus anti-PD-1 alone in the treatment of advanced hepatocellular cell carcinoma: a propensity score-matching study. *BMC Cancer*. 2022 Jan 11;22(1):55. doi: 10.1186/s12885-022-09173-4. | Yee Chao |
| Chen T, Song C, Liang G, et al. Neutrophil-to-Lymphocyte Ratio, Platelet-to-Lymphocyte Ratio, and Their Variations as a Basis for a Prediction Model in Advanced NSCLC Patients Receiving Anlotinib. *Dis Markers*. 2022 Mar 20;2022:5879137. doi: 10.1155/2022/5879137. | Mengqiu Tang |
| Chen TH, Pan YY, Lee TL, et al. Treatment outcomes of cetuximab-containing regimen in locoregional recurrent and distant metastatic head and neck squamous cell carcinoma. *BMC Cancer.* 2022 Dec 20;22(1):1336. doi: 10.1186/s12885-022-10440-7. | Peter Mu-Hsin Chang |
| Chen X, Nie J, Dai L, et al. Comparison of endostatin combined with PT-DC versus bevacizumab combined with PT-DC in the first-line treatment of advanced lung adenocarcinoma: a retrospective propensity score-matched cohort study. *Ann Palliat Med.* 2021 Jul;10(7):7847-7856. doi: 10.21037/apm-21-1401. | Jian Fang |
| Chen X, Wang J, Fan Y, et al. Primary Trastuzumab Resistance After (Neo)adjuvant Trastuzumab-containing Treatment for Patients With HER2-positive Breast Cancer in Real-world Practice. *Clin Breast Cancer.* 2021 Jun;21(3):191-198. doi: 10.1016/j.clbc.2020.09.003. | Binghe Xu |
| Chen X, Wei L, Chi L, et al. Adverse events of alpelisib: A postmarketing study of the World Health Organization pharmacovigilance database. *Br J Clin Pharmacol*. 2022 May;88(5):2180-2189. doi: 10.1111/bcp.15143. | Xiaofei Ye |
| Chen Y, Cai C, Li Y. The impact of baseline brain metastases on clinical benefits and progression patterns after first-line crizotinib in anaplastic lymphoma kinase-rearranged non-small cell lung cancer. *Medicine (Baltimore).* 2021 Feb 26;100(8):e24784. doi: 10.1097/MD.0000000000024784. | Yuqi Chen |
| Chen Y, Katayose T, Nagaoka S, et al. A post-marketing observational study of ramucirumab in patients with gastric cancer in Japan. *Gastric Cancer*. 2021 Nov;24(6):1320-1329. doi: 10.1007/s10120-021-01199-0. | Yucherng Chen |
| Chen Y, Noma S, Taguchi Y, et al. Characteristics of interstitial lung disease in patients from post-marketing data on metastatic breast cancer patients who received abemaciclib in Japan. *Breast Cancer*. 2021 May;28(3):710-719. doi: 10.1007/s12282-020-01207-8. | Yucherng Chen |
| Chen Y, Wang S, Zhang B, et al. Clinical Factors Affecting the Response to Osimertinib in Non-Small Cell Lung Cancer Patients with An Acquired Epidermal Growth Factor Receptor T790M Mutation: A Long-Term Survival Analysis. *Target Oncol.* 2020 Jun;15(3):337-345. doi: 10.1007/s11523-020-00724-y. | Baohui Han |
| Chen YC, Tsai MJ, Lee MH, et al. Lower starting dose of afatinib for the treatment of metastatic lung adenocarcinoma harboring exon 21 and exon 19 mutations. *BMC Cancer*. 2021 May 3;21(1):495. doi: 10.1186/s12885-021-08235-3. | Chih-Jen Yang |
| Chen YH, Su YC, Hsu CW, et al. Mortality of patients with metastatic colorectal cancer who received elective or emergent operation after exposure to bevacizumab: A nationwide database study. *Eur J Surg Oncol.* 2023 Feb;49(2):445-451. doi: 10.1016/j.ejso.2022.09.018. | Chih-Chien Wu |
| Chen YL, Huang AP, Wang CC, et al. Peri-radiosurgical administration of bevacizumab improves radiographic response to single and fractionated stereotactic radiosurgery for large brain metastasis. *J Neurooncol.* 2021 Jul;153(3):455-465. doi: 10.1007/s11060-021-03782-y. | Feng-Ming Hsu |
| Chen YY, Chang SC, Chang CY, et al. Real-world effectiveness of second-line Afatinib versus chemotherapy for the treatment of advanced lung squamous cell carcinoma in immunotherapy-naïve patients. *BMC Cancer.* 2021 Nov 15;21(1):1225. doi: 10.1186/s12885-021-08920-3. | Chung-Yu Chen |
| Chen Z, Ouyang Q, Wang Y, et al. Real-World First-Line Treatment Patterns and Outcomes in Hormone Receptor-Positive Advanced Breast Cancer Patients: A Multicenter, Retrospective Study in China. *Front Oncol.* 2022 Mar 3;12:829693. doi: 10.3389/fonc.2022.829693. | Xiaojia Wang |
| Cheng HF, Tsai YF, Huang CC, et al. Clinical outcomes and metastatic behavior between de novo versus recurrent HER2-positive metastatic breast cancer: A 17-year single-institution cohort study at Taipei Veterans General Hospital. *J Chin Med Assoc.* 2022 Jan 1;85(1):88-94. doi: 10.1097/JCMA.0000000000000622. | Ling-Ming Tseng |
| Cheng J, Qiu M, Zhang Y, et al. Enhanced Rim on MDCT of Colorectal Liver Metastases: Assessment of Ability to Predict Progression-Free Survival and Response to Bevacizumab-Based Chemotherapy. *AJR Am J Roentgenol*. 2020 Dec;215(6):1377-1383. doi: 10.2214/AJR.19.22280. | Yi Wang |
| Cheng JD, Chai LX, Zhao ZP, et al. Efficacy and Safety of Anlotinib for Patients with Advanced NSCLC Who Progressed After Standard Regimens and the Preliminary Analysis of an Efficacy Predictor. *Cancer Manag Res.* 2020 Jul 12;12:5641-5650. doi: 10.2147/CMAR.S253366. | Shuo Li |
| Cheng W, Kletas V, Kollmannsberger C, de Lemos M. Survival outcomes associated with different sunitinib dosing regimens in metastatic renal cell carcinoma. *J Oncol Pharm Pract*. 2020 Jan;26(1):67-73. doi: 10.1177/1078155219837333. | Victoria Kletas |
| Cheng WC, Hsia TC, Tu CY, Chen HJ. The Impact of Acquired EGFR T790M Mutation and *EGFR* Circulating Cell-Free DNA on Survival in Patients with Lung Adenocarcinoma Following EGFR-TKI Therapy. *Onco Targets Ther*. 2021 Jan 5;13:13425-13435. doi: 10.2147/OTT.S279540. | Hung-Jen Chen |
| Cheng WC, Shen YC, Chien CR, et al. The optimal therapy strategy for epidermal growth factor receptor-mutated non-small cell lung cancer patients with brain metastasis: A real-world study from Taiwan. *Thorac Cancer.* 2022 May;13(10):1505-1512. doi: 10.1111/1759-7714.14423. | Hung-Jen Chen |
| Cheng Y, Xiang H, Xin L, et al. Neoadjuvant therapy for early human epidermal growth factor receptor 2 positive breast cancer in China: A multicenter real-world study (CSBrS-015). *Chin Med J (Engl).* 2022 Oct 5;135(19):2311-2318. doi: 10.1097/CM9.0000000000002197. | Yinhua Liu |
| Cheon J, Yoo C, Hong JY, et al. Efficacy and safety of atezolizumab plus bevacizumab in Korean patients with advanced hepatocellular carcinoma. *Liver Int.* 2022 Mar;42(3):674-681. doi: 10.1111/liv.15102. | Ho Yeong Lim |
| Chevalier T, Daste A, Saada-Bouzid E, et al. Cetuximab combined with paclitaxel or paclitaxel alone for patients with recurrent or metastatic head and neck squamous cell carcinoma progressing after EXTREME. *Cancer Med.* 2021 Jun;10(12):3952-3963. doi: 10.1002/cam4.3953. | Thomas Chevalier |
| Chiang CL, Huang HC, Shen CI, et al. Post-Progression Survival in Secondary EGFR T790M-Mutated Non-Small-Cell Lung Cancer Patients With and Without Osimertinib After Failure of a Previous EGFR TKI. *Target Oncol.* 2020 Aug;15(4):503-512. doi: 10.1007/s11523-020-00737-7. | Chao-Hua Chiu |
| Chiang TY, Hsu HC, Jane SW, Chen SC. EGFRI-associated health-related quality of life by severity of skin toxicity in metastatic colorectal cancer patients receiving epidermal growth factor receptor inhibitor target therapy. *Support Care Cancer.* 2020 Oct;28(10):4771-4779. doi: 10.1007/s00520-020-05321-3. | Shu-Ching Chen |
| Chida K, Kotani D, Moriwaki T, et al. Survival Benefit of Crossover Administration of Regorafenib and Trifluridine/Tipiracil Hydrochloride for Patients With Metastatic Colorectal Cancer: Exploratory Analysis of a Japanese Society for Cancer of the Colon and Rectum Multicenter Observational Study (REGOTAS). *Front Oncol.* 2021 Mar 8;11:576036. doi: 10.3389/fonc.2021.576036. | Daisuke Kotani |
| Chida K, Kotani D, Nakamura Y, et al. Efficacy and safety of trifluridine/tipiracil plus bevacizumab and trifluridine/tipiracil or regorafenib monotherapy for chemorefractory metastatic colorectal cancer: a retrospective study. *Ther Adv Med Oncol*. 2021 Apr 20;13:17588359211009143. doi: 10.1177/17588359211009143. | Daisuke Kotani |
| Chiu TH, Tung PH, Huang CH, et al. The different overall survival between single-agent EGFR-TKI treatment and with bevacizumab in non-small cell lung cancer patients with brain metastasis. *Sci Rep*. 2022 Mar 15;12(1):4398. doi: 10.1038/s41598-022-08449-w. | Chih-Hsi Scott Kuo |
| Cho A, Kranawetter B, Untersteiner H, et al. Neutrophil-to-Lymphocyte Ratio Is Superior to Other Leukocyte-Based Ratios as a Prognostic Predictor in Non-Small Cell Lung Cancer Patients with Radiosurgically Treated Brain Metastases Under Immunotherapy or Targeted Therapy. *World Neurosurg.* 2021 Jul;151:e324-e331. doi: 10.1016/j.wneu.2021.04.033. | Josa M. Frischer |
| Cho BC, Kim DW, Park K, et al. Real-world use of osimertinib in non-small cell lung cancer: ASTRIS study Korean subgroup analysis. *Curr Med Res Opin*. 2020 Mar;36(3):477-482. doi: 10.1080/03007995.2019.1676708. | Sang-We Kim |
| Cho YY, Yu SJ, Lee HW, et al. Clinical Characteristics of Long-Term Survivors After Sorafenib Treatment for Unresectable Hepatocellular Carcinoma: A Korean National Multicenter Retrospective Cohort Study. *J Hepatocell Carcinoma.* 2021 Jun 18;8:613-623. doi: 10.2147/JHC.S304439. | Yoon Jun Kim |
| Choi HJ, Lee YY, Choi CH, et al. Triplet chemotherapy vs doublet chemotherapy plus bevacizumab in metastatic, recurrent, and persistent cervical cancer. *Curr Probl Cancer*. 2020 Oct;44(5):100557. doi: 10.1016/j.currproblcancer.2020.100557. | Byoung-Gie Kim |
| Choi MG, Choi CM, Lee DH, et al. Different prognostic implications of hepatic metastasis according to front-line treatment in non-small cell lung cancer: a real-world retrospective study. *Transl Lung Cancer Res.* 2021 Jun;10(6):2551-2561. doi: 10.21037/tlcr-21-206. | Jae Cheol Lee |
| Choi NR, Kim JY, Hong JH, et al. Comparison of the outcomes between sorafenib and lenvatinib as the first-line systemic treatment for HBV-associated hepatocellular carcinoma: a propensity score matching analysis. *BMC Gastroenterol*. 2022 Mar 25;22(1):135. doi: 10.1186/s12876-022-02210-3. | Yoon Jun Kim |
| Choi SJ, Lee SS, Jung KH, et al. Noncirrhotic Portal Hypertension after Trastuzumab Emtansine in HER2-positive Breast Cancer as Determined by Deep Learning-measured Spleen Volume at CT. *Radiology.* 2022 Dec;305(3):606-613. doi: 10.1148/radiol.220536. | Seung Soo Lee |
| Choi SY, Ha MS, Lee JW, et al. Shifting role of cytoreductive nephrectomy according to type of systemic therapy: A nationwide cohort study. *Asian J Surg*. 2023 Jan;46(1):328-336. doi: 10.1016/j.asjsur.2022.04.008. | Se Young Choi |
| Choi WM, Choi J, Lee D, et al. Regorafenib Versus Nivolumab After Sorafenib Failure: Real-World Data in Patients With Hepatocellular Carcinoma. *Hepatol Commun.* 2020 Jun 16;4(7):1073-1086. doi: 10.1002/hep4.1523. | Kang Mo Kim |
| Chon YE, Cheon J, Kim H, et al. Predictive biomarkers of survival in patients with advanced hepatocellular carcinoma receiving atezolizumab plus bevacizumab treatment. *Cancer Med*. 2023 Feb;12(3):2731-2738. doi: 10.1002/cam4.5161. | Beom Kyung Kim |
| Chong LC, Hardingham JE, Townsend AR, et al. Rechallenge with Anti-EGFR Therapy in Metastatic Colorectal Cancer (mCRC): Results from South Australia mCRC Registry. *Target Oncol*. 2020 Dec;15(6):751-757. doi: 10.1007/s11523-020-00760-8. | Timothy J. Price |
| Choong GM, Liddell S, Ferre RAL, et al. Clinical management of metastatic hormone receptor-positive, HER2-negative breast cancer (MBC) after CDK 4/6 inhibitors: a retrospective single-institution study. *Breast Cancer Res Treat*. 2022 Nov;196(1):229-237. doi: 10.1007/s10549-022-06713-1. | Karthik V. Giridhar |
| Chou HH, Fereday S, DeFazio A, et al. Contrasting clinical characteristics and treatment patterns in women with newly diagnosed advanced-stage epithelial ovarian cancer in Australia, South Korea and Taiwan. *J Gynecol Oncol.* 2023 Jan;34(1):e3. doi: 10.3802/jgo.2023.34.e3. | Byoung-Gie Kim |
| Christensen T, Berg T, Nielsen LB, et al. Dual HER2 blockade in the first-line treatment of metastatic breast cancer - A retrospective population-based observational study in Danish patients. *Breast.* 2020 Jun;51:34-39. doi: 10.1016/j.breast.2020.03.002. | Thomas Christensen |
| Chu G, Liu X, Yu W, et al. Cisplatin plus paclitaxel chemotherapy with or without bevacizumab in postmenopausal women with previously untreated advanced cervical cancer: a retrospective study. *BMC Cancer*. 2021 Feb 6;21(1):133. doi: 10.1186/s12885-021-07869-7. | Lingyun Dong |
| Chu HH, Kim JH, Shim JH, et al. Chemoembolization Plus Radiotherapy Versus Chemoembolization Plus Sorafenib for the Treatment of Hepatocellular Carcinoma Invading the Portal Vein: A Propensity Score Matching Analysis. *Cancers (Basel).* 2020 Apr 29;12(5):1116. doi: 10.3390/cancers12051116. | Jin Hyoung Kim |
| Chu SS, Kuo YH, Liu WS, et al. Effect of radiotherapy on survival in advanced hepatocellular carcinoma patients treated with sorafenib: a nationwide cancer-registry-based study. *Sci Rep.* 2021 Jan 15;11(1):1614. doi: 10.1038/s41598-021-81176-w. | Hung-Chang Wu |
| Chung SW, Park MK, Cho YY, et al. Effectiveness of Transarterial Chemoembolization-First Treatment for Advanced Hepatocellular Carcinoma: A Propensity Score Matching Analysis. *J Hepatocell Carcinoma.* 2021 Jun 15;8:587-598. doi: 10.2147/JHC.S294440. | Jeong-Hoon Lee |
| Cohen Aubart F, Lhote R, Amoura A, et al. Drug-induced sarcoidosis: an overview of the WHO pharmacovigilance database. *J Intern Med.* 2020 Sep;288(3):356-362. doi: 10.1111/joim.12991. | Fleur Cohen Aubart |
| Colomba E, Alves Costa Silva C, Le Teuff G, et al. Weight and skeletal muscle loss with cabozantinib in metastatic renal cell carcinoma. *J Cachexia Sarcopenia Muscle.* 2022 Oct;13(5):2405-2416. doi: 10.1002/jcsm.13021. | Laurence Albiges |
| Coltelli L, Allegrini G, Orlandi P, et al. A pharmacogenetic interaction analysis of bevacizumab with paclitaxel in advanced breast cancer patients. *NPJ Breast Cancer.* 2022 Mar 21;8(1):33. doi: 10.1038/s41523-022-00400-6. | Guido Bocci |
| Copeland-Halperin RS, Al-Sadawi M, Patil S, et al. Early Trastuzumab Interruption and Recurrence-Free Survival in ERBB2-Positive Breast Cancer. *JAMA Oncol.* 2020 Dec 1;6(12):1971-1972. doi: 10.1001/jamaoncol.2020.4749. | Anthony F. Yu |
| Costello BA, Bhavsar NA, Zakharia Y, et al. A Prospective Multicenter Evaluation of Initial Treatment Choice in Metastatic Renal Cell Carcinoma Prior to the Immunotherapy Era: The MaRCC Registry Experience. *Clin Genitourin Cancer*. 2022 Feb;20(1):1-10. doi: 10.1016/j.clgc.2021.07.002. | Brian A. Costello |
| Cotes Sanchís A, Gallego J, Hernandez R, et al. Second-line treatment in advanced gastric cancer: Data from the Spanish AGAMENON registry. *PLoS One.* 2020 Jul 31;15(7):e0235848. doi: 10.1371/journal.pone.0235848. | Javier Gallego |
| Courtney D, Davey MG, Moloney BM, et al. Breast cancer recurrence: factors impacting occurrence and survival. *Ir J Med Sci*. 2022 Dec;191(6):2501-2510. doi: 10.1007/s11845-022-02926-x. | Matthew G. Davey |
| Coutzac C, Trouilloud I, Artru P, et al. Sequential Treatment With Trifluridine/Tipiracil and Regorafenib in Refractory Metastatic Colorectal Cancer Patients: An AGEO Prospective "Real-World Study". *Clin Colorectal Cancer*. 2022 Jun;21(2):132-140. doi: 10.1016/j.clcc.2021.12.003. | Julien Taieb |
| Cowey CL, Boyd M, Aguilar KM, et al. An observational study of drug utilization and associated outcomes among adult patients diagnosed with BRAF-mutant advanced melanoma treated with first-line anti-PD-1 monotherapies or BRAF/MEK inhibitors in a community-based oncology setting. *Cancer Med.* 2020 Nov;9(21):7863-7878. doi: 10.1002/cam4.3312. | Charles L. Cowey |
| Cui Q, Mao Y, Wu D, et al. Apatinib combined with PD-1 antibody for third-line or later treatment of advanced gastric cancer. *Front Oncol.* 2022 Oct 28;12:952494. doi: 10.3389/fonc.2022.952494. | Huaimin Liu |
| Cullom ME, Amin AL, Balanoff CR, et al. Evaluation of breast surgical oncology complications after single agent versus dual agent HER2 targeted neoadjuvant chemotherapy. *Am J Surg*. 2020 Nov;220(5):1225-1229. doi: 10.1016/j.amjsurg.2020.06.053. | Kelsey E. Larson |
| Cuyun Carter G, Sheffield KM, Gossai A, et al. Real-world treatment patterns and outcomes of abemaciclib for the treatment of HR+, HER2- metastatic breast cancer. *Curr Med Res Opin.* 2021 Jul;37(7):1179-1187. doi: 10.1080/03007995.2021.1923468. | Gebra Cuyun Carter |
| Dackus GMHE, Jóźwiak K, van der Wall E, et al. Concurrent versus sequential use of trastuzumab and chemotherapy in early HER2+ breast cancer. *Breast Cancer Res Treat.* 2021 Feb;185(3):817-830. doi: 10.1007/s10549-020-05978-8. | Sabine C. Linn |
| Dahlan YM, Shirah BH, Alghamdi AS, et al. Clinical Outcomes of Radiological Treatment Modalities of Hepatocellular Carcinoma: A Single-Center Experience from Saudi Arabia. *Gulf J Oncolog*. 2022 May;1(39):56-62. | Yaser M. Dahlan |
| Dai L, Wang W, Li W, et al. Effect of Gefitinib Combined with Chemotherapy in Patients with Advanced NSCLC: A Retrospective Cohort Study. *Int J Gen Med.* 2022 Jan 15;15:637-644. doi: 10.2147/IJGM.S342917. | Kaixin Qu |
| Dai WF, Beca JM, Nagamuthu C, et al. Comparative Effectiveness and Safety of Pertuzumab and Trastuzumab Plus Chemotherapy vs Trastuzumab Plus Chemotherapy for Treatment of Metastatic Breast Cancer. *JAMA Netw Open*. 2022 Feb 1;5(2):e2145460. doi: 10.1001/jamanetworkopen.2021.45460. | Kelvin K. W. Chan |
| Dai Y, Sun L, Zhuang L, et al. Efficacy and safety of low-dose apatinib plus S-1 versus regorafenib and fruquintinib for refractory metastatic colorectal cancer: a retrospective cohort study. *J Gastrointest Oncol*. 2022 Apr;13(2):722-731. doi: 10.21037/jgo-22-285. | Hong Qiu. |
| Dal Maso A, Lorenzi M, Ferro A, et al. Real-world data on treatment outcomes in EGFR-mutant non-small-cell lung cancer patients receiving osimertinib in second or further lines. *Future Oncol*. 2021 Jul;17(19):2513-2527. doi: 10.2217/fon-2021-0356. | Giulia Pasello |
| Dal Maso A, Lorenzi M, Roca E, et al. Clinical Features and Progression Pattern of Acquired T790M-positive Compared With T790M-negative EGFR Mutant Non-small-cell Lung Cancer: Catching Tumor and Clinical Heterogeneity Over Time Through Liquid Biopsy. *Clin Lung Cancer.* 2020 Jan;21(1):1-14.e3. doi: 10.1016/j.cllc.2019.07.009. | Giulia Pasello |
| D'Alessio A, Fulgenzi CAM, Nishida N, et al. Preliminary evidence of safety and tolerability of atezolizumab plus bevacizumab in patients with hepatocellular carcinoma and Child-Pugh A and B cirrhosis: A real-world study. *Hepatology*. 2022 Oct;76(4):1000-1012. doi: 10.1002/hep.32468. | David J. Pinato |
| Daniels B, Kiely BE, Tang M, et al. Trastuzumab emtansine for HER2-positive metastatic breast cancer: Outcomes from a whole-of-population Australian cohort. *Breast.* 2021 Aug;58:106-112. doi: 10.1016/j.breast.2021.05.001. | Benjamin Daniels |
| de Castro T, Jochheim LS, Bathon M, et al. Atezolizumab and bevacizumab in patients with advanced hepatocellular carcinoma with impaired liver function and prior systemic therapy: a real-world experience. *Ther Adv Med Oncol.* 2022 Feb 26;14:17588359221080298. doi: 10.1177/17588359221080298. | Arndt Vogel |
| De Sanctis R, Giordano L, D'Antonio F, et al. Clinical predictors of cardiac toxicity in HER2-positive early breast cancer patients treated with adjuvant s.c. versus i.v. trastuzumab. *Breast.* 2021 Jun;57:80-85. doi: 10.1016/j.breast.2021.03.004. | Rita De Sanctis |
| DeBusk K, Ike C, Lindegger N, et al. Real-world outcomes among patients with HER2+ metastatic breast cancer with brain metastases. *J Manag Care Spec Pharm*. 2022 Jun;28(6):657-666. doi: 10.18553/jmcp.2022.28.6.657. | Kendra DeBusk |
| Degeling K, Wong HL, Koffijberg H, et al. Simulating Progression-Free and Overall Survival for First-Line Doublet Chemotherapy With or Without Bevacizumab in Metastatic Colorectal Cancer Patients Based on Real-World Registry Data. *Pharmacoeconomics.* 2020 Nov;38(11):1263-1275. doi: 10.1007/s40273-020-00951-1. | Koen Degeling |
| Del Re M, Crucitta S, Omarini C, et al. Concomitant administration of proton pump inhibitors does not significantly affect clinical outcomes in metastatic breast cancer patients treated with ribociclib. *Breast.* 2022 Dec;66:157-161. doi: 10.1016/j.breast.2022.10.005. | Stefano Fogli |
| Del Re M, Omarini C, Diodati L, et al. Drug-drug interactions between palbociclib and proton pump inhibitors may significantly affect clinical outcome of metastatic breast cancer patients. *ESMO Open.* 2021 Oct;6(5):100231. doi: 10.1016/j.esmoop.2021.100231. | Romano Danesi |
| DeMichele A, Cristofanilli M, Brufsky A, et al. Comparative effectiveness of first-line palbociclib plus letrozole versus letrozole alone for HR+/HER2- metastatic breast cancer in US real-world clinical practice. *Breast Cancer Res*. 2021 Mar 24;23(1):37. doi: 10.1186/s13058-021-01409-8. | Angela DeMichele |
| Demircan NC, Alan Ö, Başoğlu Tüylü T, et al. Impact of the Charlson Comorbidity Index on dose-limiting toxicity and survival in locally advanced and metastatic renal cell carcinoma patients treated with first-line sunitinib or pazopanib. *J Oncol Pharm Pract*. 2020 Jul;26(5):1147-1155. doi: 10.1177/1078155219890032. | Nazım C. Demircan |
| Denduluri N, Espirito JL, Hackshaw MD, et al. Retrospective Observational Study of Outcomes in HER2-Positive Metastatic Breast Cancer (mBC) Patients Treated with Ado-Trastuzumab Emtansine (T-DM1) and Subsequent Treatments After T-DM1 in the United States. *Drugs Real World Outcomes*. 2023 Jun;10(2):177-186. doi: 10.1007/s40801-022-00340-4. | Neelima Denduluri |
| Dennison T, Heiling H, Deal A, et al. Tolerability of palbociclib in younger and older patients with advanced breast cancer. *J Oncol Pharm Pract*. 2023 Jan;29(1):96-104. doi: 10.1177/10781552211053639. | Aimee Faso |
| Dercle L, Lu L, Schwartz LH, et al. Radiomics Response Signature for Identification of Metastatic Colorectal Cancer Sensitive to Therapies Targeting EGFR Pathway. *J Natl Cancer Inst.* 2020 Sep 1;112(9):902-912. doi: 10.1093/jnci/djaa017. | Laurent Dercle |
| Descourt R, Pérol M, Rousseau-Bussac G, et al. Brigatinib for Pretreated, ALK-Positive, Advanced Non-Small-Cell Lung Cancers: Long-Term Follow-Up and Focus on Post-Brigatinib Lorlatinib Efficacy in the Multicenter, Real-World BrigALK2 Study. *Cancers (Basel).* 2022 Mar 30;14(7):1751. doi: 10.3390/cancers14071751. | Renaud Descourt |
| Deutsch TM, Riethdorf S, Fremd C, et al. HER2-targeted therapy influences CTC status in metastatic breast cancer. *Breast Cancer Res Treat*. 2020 Jul;182(1):127-136. doi: 10.1007/s10549-020-05687-2. | Markus Wallwiener |
| Di Filippo Y, Dalle S, Mortier L, et al. Relevance of body mass index as a predictor of systemic therapy outcomes in metastatic melanoma: analysis of the MelBase French cohort data☆. *Ann Oncol.* 2021 Apr;32(4):542-551. doi: 10.1016/j.annonc.2020.12.012. | Henri Montaudié |
| Dibba P, Ludwig E, Calo D, et al. Bevacizumab does not increase risk of perforation in patients undergoing percutaneous endoscopic gastrostomy or jejunostomy placement. *Surg Endosc*. 2021 Jun;35(6):2976-2980. doi: 10.1007/s00464-020-07738-7. | Robin Mendelsohn |
| Dijksterhuis WPM, Verhoeven RHA, Meijer SL, et al. Increased assessment of HER2 in metastatic gastroesophageal cancer patients: a nationwide population-based cohort study. *Gastric Cancer.* 2020 Jul;23(4):579-590. doi: 10.1007/s10120-020-01039-7. | Hanneke W. M. van Laarhoven |
| Dimitriou F, Urner-Bloch U, Eggenschwiler C, et al. The association between immune checkpoint or BRAF/MEK inhibitor therapy and uveitis in patients with advanced cutaneous melanoma. *Eur J Cancer.* 2021 Feb;144:215-223. doi: 10.1016/j.ejca.2020.11.027. | Reinhard Dummer |
| Ding H, Wen W, Ding Q, Zhao X. Diagnostic Valuation of Serum miR-184 and miR-191 in Patients With Non-Small-Cell Lung Cancer. *Cancer Control.* 2020 Jan-Dec;27(1):1073274820964783. doi: 10.1177/1073274820964783. | Xin Zhao |
| Ding N, Huang J, Li N, et al. Roles of neutrophil/lymphocyte ratio in prognosis and in differentiation of potential beneficiaries in HER2-positive breast cancer with trastuzumab therapy. *BMC Cancer.* 2020 Mar 19;20(1):235. doi: 10.1186/s12885-020-06750-3. | Zhi Xiao |
| Ding PN, Roberts TL, Chua W, et al. Plasma pre-treatment T790M relative allelic frequency in patients with advanced EGFR-mutated non-small cell lung cancer predicts treatment response to subsequent-line osimertinib. *Transl Lung Cancer Res.* 2021 Apr;10(4):1623-1634. doi: 10.21037/tlcr-20-1125. | Pei Ni Ding |
| Doehn C, Bögemann M, Grünwald V, et al. The Non-Interventional PAZOREAL Study to Assess the Effectiveness and Safety of Pazopanib in a Real-Life Setting: Reflecting a Changing mRCC Treatment Landscape. *Cancers (Basel).* 2022 Nov 8;14(22):5486. doi: 10.3390/cancers14225486. | Christian Doehn |
| Dolladille C, Launoy G, Bouvier V, et al. Association Between Use of Anticancer Drugs and Cardiovascular Disease-Related Hospitalization in Metastatic Colorectal Cancer: Insights From a Population-Based Study, the Anticancer Vigilance of Cardiac Events Study. *Am J Epidemiol*. 2021 Feb 1;190(3):376-385. doi: 10.1093/aje/kwaa203. | Charles Dolladille |
| Donskov F, Pinto CA, Predoiu R, et al. Molecular analysis and favorable clinical outcomes in real-world patients with metastatic renal cell carcinoma. *Acta Oncol.* 2022 Oct;61(10):1268-1277. doi: 10.1080/0284186X.2022.2119100. | Frede Donskov |
| Du R, Wang X, Zhou H, et al. The health-related quality of life of lung cancer patients with EGFR-TKI-related skin adverse drug reactions and its relationship with coping style and self-management. *Support Care Cancer.* 2022 Dec;30(12):9889-9899. doi: 10.1007/s00520-022-07451-2. | Changying Chen |
| Du R, Yang H, Zhou H, et al. The relationship between medication literacy and skin adverse reactions in non-small-cell lung cancer patients undergoing targeted EGFR-TKI therapy. *BMC Cancer.* 2022 May 3;22(1):491. doi: 10.1186/s12885-022-09599-w. | Changying Chen |
| Du Y, Cao Q, Jiang C, et al. Effectiveness and safety of low-dose apatinib in advanced gastric cancer: A real-world study. *Cancer Med*. 2020 Jul;9(14):5008-5014. doi: 10.1002/cam4.3105. | Guoping Sun |
| Duan F, Song C, Ma Y, et al. Establishment of Prognostic Nomograms for Predicting the Survival of HR-Positive, HER2-Negative Metastatic Breast Cancer Patients Treated with Everolimus. *Drug Des Devel Ther.* 2021 Aug 10;15:3463-3473. doi: 10.2147/DDDT.S314723. | Wen Xia |
| Duco MR, Murdock JL, Reeves DJ. Vascular endothelial growth factor inhibitor induced hypertension: Retrospective analysis of the impact of blood pressure elevations on outcomes. *J Oncol Pharm Pract*. 2022 Mar;28(2):265-273. doi: 10.1177/1078155220985915. | David J. Reeves |
| Dupuis HGA, Chebbi A, Surlemont L, et al. Efficacy and safety of anti-vascular endothelial growth factor therapies in older patients for first line treatment of metastatic renal cell carcinoma. *Transl Androl Urol.* 2021 Jun;10(6):2418-2426. doi: 10.21037/tau-20-1481. | Hugo Georges Arthur Dupuis |
| Eggers H, Häbel L, Ganser A, et al. Anti-EGFR-Based Therapy in Recurrent or Metastatic HNSCC - What Difference Does it Make? *Cancer Invest.* 2023 Jan;41(1):93-100. doi: 10.1080/07357907.2022.2134414. | Philipp Ivanyi |
| Eggers H, Schünemann C, Grünwald V, et al. Improving survival in metastatic renal cell carcinoma (mRCC) patients: do elderly patients benefit from expanded targeted therapeutic options? *World J Urol.* 2022 Oct;40(10):2489-2497. doi: 10.1007/s00345-022-04110-3. | Philipp Ivanyi |
| Ehrenstein V, Huang K, Kahlert J, et al. Outcomes in patients with lung cancer treated with crizotinib and erlotinib in routine clinical practice: A post-authorization safety cohort study conducted in Europe and in the United States. *Pharmacoepidemiol Drug Saf*. 2021 Jun;30(6):758-769. doi: 10.1002/pds.5193. | Vera Ehrenstein |
| Eisele A, Seystahl K, Rushing EJ, et al. Venous thromboembolic events in glioblastoma patients: An epidemiological study. *Eur J Neurol.* 2022 Aug;29(8):2386-2397. doi: 10.1111/ene.15404. | Dorothee Gramatzki |
| El Badri S, Tahir B, Balachandran K, et al. Palbociclib in combination with aromatase inhibitors in patients ≥ 75 years with oestrogen receptor-positive, human epidermal growth factor receptor 2 negative advanced breast cancer: A real-world multicentre UK study. *Breast.* 2021 Dec;60:199-205. doi: 10.1016/j.breast.2021.10.010. | Salma El Badri |
| Ellebaek E, Svane IM, Schmidt H, et al. The Danish metastatic melanoma database (DAMMED): A nation-wide platform for quality assurance and research in real-world data on medical therapy in Danish melanoma patients. *Cancer Epidemiol.* 2021 Aug;73:101943. doi: 10.1016/j.canep.2021.101943. | Eva Ellebaek |
| Ellegård S, Engvall K, Asowed M, et al. Long-term follow-up of early stage HER2-positive breast cancer patients treated with trastuzumab: A population-based real world multicenter cohort study. *Front Oncol.* 2022 Aug 2;12:861324. doi: 10.3389/fonc.2022.861324. | Sander Ellegård |
| Ellis-Caleo T, Neal JW. The role of ramucirumab with docetaxel in epidermal growth factor receptor mutant and wild-type non-small cell lung cancer. *J Thorac Dis.* 2021 Aug;13(8):4864-4871. doi: 10.21037/jtd-21-557. | Joel W. Neal |
| Emile G, Penager S, Levy C, et al. Baseline lymphopenia as prognostic factor in patients with metastatic breast cancer treated with palbociclib. *Oncol Lett.* 2022 Jan;23(1):25. doi: 10.3892/ol.2021.13143. | George Emile |
| Erickson AW, Habbous S, Wright F, et al. Assessing the Association of Targeted Therapy and Intracranial Metastatic Disease. *JAMA Oncol.* 2021 Aug 1;7(8):1220-1224. doi: 10.1001/jamaoncol.2021.1600. | Sunit Das |
| Erman M, Biswas B, Danchaivijitr P, et al. Prospective observational study on Pazopanib in patients treated for advanced or metastatic renal cell carcinoma in countries in Asia Pacific, North Africa, and Middle East regions: PARACHUTE study. *BMC Cancer*. 2021 Sep 14;21(1):1021. doi: 10.1186/s12885-021-08738-z. | Ravindran Kanesvaran |
| Erol C, Sendur Mehmet AN, Bilgetekin I, et al. Efficacy and safety of folfiri plus aflibercept in second-line treatment of metastatic colorectal cancer: Real-life data from Turkish oncology group. *J Cancer Res Ther.* 2022 Dec;18(12 Suppl 2):S347-S353. doi: 10.4103/jcrt.jcrt_1104_21. | Cihan Erol |
| Escudier B, de Zélicourt M, Bourouina R, et al. Management and Health Resource Use of Patients With Metastatic Renal Cell Carcinoma treated With Systemic Therapy Over 2014-2017 in France: A National Real-World Study. *Clin Genitourin Cancer.* 2022 Dec;20(6):533-542. doi: 10.1016/j.clgc.2022.07.010. | Antoine Thiery-Vuillemin |
| Eser K, Önder AH, Sezer E, et al. Proton pump inhibitors may reduce the efficacy of ribociclib and palbociclib in metastatic breast cancer patients based on an observational study. *BMC Cancer*. 2022 May 7;22(1):516. doi: 10.1186/s12885-022-09624-y. | Kadir Eser |
| Ethier JL, Desautels D, Robinson A, et al. Practice Patterns and Outcomes of Novel Targeted Agents for the Treatment of ERBB2-Positive Metastatic Breast Cancer. *JAMA Oncol.* 2021 Sep 1;7(9):e212140. doi: 10.1001/jamaoncol.2021.2140. | Josee-Lyne Ethier |
| Famularo S, Donadon M, Cipriani F, et al. Hepatectomy Versus Sorafenib in Advanced Nonmetastatic Hepatocellular Carcinoma: A Real-life Multicentric Weighted Comparison. *Ann Surg.* 2022 Apr 1;275(4):743-752. doi: 10.1097/SLA.0000000000005373. | Guido Torzilli |
| Fan W, Zhu B, Yue S, et al. Idarubicin-Loaded DEB-TACE plus Lenvatinib versus Lenvatinib for patients with advanced hepatocellular carcinoma: A propensity score-matching analysis. *Cancer Med.* 2023 Jan;12(1):61-72. doi: 10.1002/cam4.4937. | Jiaping Li |
| Fang Y, Wang Z, Wu J, et al. Factors Influencing Adjuvant Chemotherapy and Trastuzumab Choice in Older Human Epidermal Growth Factor Receptor 2-positive Breast Cancer Patients. *J Cancer.* 2020 Feb 14;11(9):2602-2609. doi: 10.7150/jca.39509. | Kunwei Shen |
| Farrukh N, Bano R, Naqvi SRQ, Latif H. Assessment of Pathological Complete Response in Patients with Breast Cancer Receiving Neoadjuvant Systemic Therapy*. J Coll Physicians Surg Pak.* 2022 Jun;32(6):746-750. doi: 10.29271/jcpsp.2022.06.746. | Nida Farrukh |
| Feinberg B, Halmos B, Gucalp R, et al. Making the case for EGFR TKI sequencing in EGFR mutation-positive NSCLC: a GioTag study US patient analysis. *Future Oncol*. 2020 Aug;16(22):1585-1595. doi: 10.2217/fon-2020-0188. | Bruce Feinberg |
| Feng Y, Qin Z, Yang Z. Deceleration capacity of heart rate predicts trastuzumab-related cardiotoxicity in patients with HER2-positive breast cancer: A prospective observational study. *J Clin Pharm Ther*. 2021 Feb;46(1):93-98. doi: 10.1111/jcpt.13258. | Zhijun Yang |
| Ferraro E, Singh J, Patil S, et al. Incidence of brain metastases in patients with early HER2-positive breast cancer receiving neoadjuvant chemotherapy with trastuzumab and pertuzumab. *NPJ Breast Cancer*. 2022 Mar 22;8(1):37. doi: 10.1038/s41523-022-00380-7. | Chau T. Dang |
| Fiala O, Finek J, Poprach A, et al. Outcomes According to MSKCC Risk Score with Focus on the Intermediate-Risk Group in Metastatic Renal Cell Carcinoma Patients Treated with First-Line Sunitinib: A Retrospective Analysis of 2390 Patients. Cancers (Basel). 2020 Mar 27;12(4):808. doi: 10.3390/cancers12040808. | Ondrej Fiala |
| Fiala O, Ostašov P, Rozsypalová A, et al. Impact of Concomitant Cardiovascular Medication on Survival of Metastatic Renal Cell Carcinoma Patients Treated with Sunitinib or Pazopanib in the First Line. *Target Oncol.* 2021 Sep;16(5):643-652. doi: 10.1007/s11523-021-00829-y. | Ondřej Fiala |
| Fiala O, Ostašov P, Rozsypalová A, et al. Metformin Use and the Outcome of Metastatic Renal Cell Carcinoma Treated with Sunitinib or Pazopanib. *Cancer Manag Res.* 2021 May 21;13:4077-4086. doi: 10.2147/CMAR.S305321. | Ondřej Fiala |
| Fluhrer H, Hutterer GC, Golbeck S, et al. Improved overall survival of metastatic renal cell carcinoma patients in the era of modern tyrosine kinase inhibitors and immune checkpoint inhibitors: results from a real-life, population-based Austrian study comprising three decades of follow-up. *Ther Adv Med Oncol.* 2022 Nov 19;14:17588359221134065. doi: 10.1177/17588359221134065. | Martin Pichler |
| Forschner A, Loquai C, Meiss F, et al. Is there an overtreatment of melanoma patients at the end of their life? Results of a multicenter study on 193 melanoma patients. *J Dtsch Dermatol Ges.* 2021 Sep;19(9):1297-1305. doi: 10.1111/ddg.14501. | Andrea Forschner |
| Franchi M, Garau D, Kirchmayer U, et al. Effectiveness and Costs Associated to Adding Cetuximab or Bevacizumab to Chemotherapy as Initial Treatment in Metastatic Colorectal Cancer: Results from the Observational FABIO Project. *Cancers (Basel).* 2020 Mar 31;12(4):839. doi: 10.3390/cancers12040839. | Matteo Franchi |
| Franchi M, Trama A, Merlo I, et al. Cardiovascular Risk After Adjuvant Trastuzumab in Early Breast Cancer: An Italian Population-Based Cohort Study. *Oncologist.* 2020 Oct;25(10):e1492-e1499. doi: 10.1634/theoncologist.2020-0216. | Matteo Franchi |
| Franchi M, Tritto R, Torroni L, et al. Effectiveness and Healthcare Cost of Adding Trastuzumab to Standard Chemotherapy for First-Line Treatment of Metastatic Gastric Cancer: A Population-Based Cohort Study. *Cancers (Basel).* 2020 Jun 25;12(6):1691. doi: 10.3390/cancers12061691. | Matteo Franchi |
| François E, Mineur L, Deplanque G, et al. Efficacy and Safety of Bevacizumab Combined With First-Line Chemotherapy in Elderly (≥75 Years) Patients With Metastatic Colorectal Cancer: A Real-World Study. *Clin Colorectal Cancer*. 2020 Sep;19(3):e100-e109. doi: 10.1016/j.clcc.2020.02.009. | Eric François |
| Franklin C, Mohr P, Bluhm L, et al. Impact of radiotherapy and sequencing of systemic therapy on survival outcomes in melanoma patients with previously untreated brain metastasis: a multicenter DeCOG study on 450 patients from the prospective skin cancer registry ADOREG. *J Immunother Cancer.* 2022 Jun;10(6):e004509. doi: 10.1136/jitc-2022-004509. | Cindy Franklin |
| Fu Z, Li X, Zhong J, et al. Lenvatinib in combination with transarterial chemoembolization for treatment of unresectable hepatocellular carcinoma (uHCC): a retrospective controlled study. *Hepatol Int.* 2021 Jun;15(3):663-675. doi: 10.1007/s12072-021-10184-9. | Zengqiang Qu |
| Fujisawa Y, Ito T, Kato H, et al. Outcome of combination therapy using BRAF and MEK inhibitors among Asian patients with advanced melanoma: An analysis of 112 cases. *Eur J Cancer.* 2021 Mar;145:210-220. doi: 10.1016/j.ejca.2020.12.021. | Yasuhiro Fujisawa |
| Fujita H, Murakami T, Tomoike F, et al. Ceritinib-associated hyperglycemia in the Japanese Adverse Drug Event Report Database. *J Diabetes Investig*. 2020 May;11(3):726-730. doi: 10.1111/jdi.13168. | Nobuya Inagaki |
| Fujita M, Abe K, Kuroda H, et al. Influence of skeletal muscle volume loss during lenvatinib treatment on prognosis in unresectable hepatocellular carcinoma: a multicenter study in Tohoku, Japan. *Sci Rep*. 2022 Apr 20;12(1):6479. doi: 10.1038/s41598-022-10514-3. | Masashi Fujita |
| Fujita N, Fujita K, Kim SJ, et al. Response-Guided Omission of Anthracycline in Patients with HER2-Positive Early Breast Cancer Treated with Neoadjuvant Taxane and Trastuzumab: 5-Year Follow-Up of Prognostic Study Using Propensity Score Matching. *Oncology.* 2022;100(5):257-266. doi: 10.1159/000522384. | Noriko Fujita |
| Fukushima T, Morimoto M, Ueno M, et al. Comparative study between sorafenib and lenvatinib as the first-line therapy in the sequential treatment of unresectable hepatocellular carcinoma in a real-world setting. *JGH Open*. 2021 Dec 17;6(1):29-35. doi: 10.1002/jgh3.12691. | Manabu Morimoto |
| Fulgenzi CAM, Cheon J, D'Alessio A, et al. Reproducible safety and efficacy of atezolizumab plus bevacizumab for HCC in clinical practice: Results of the AB-real study. *Eur J Cancer*. 2022 Nov;175:204-213. doi: 10.1016/j.ejca.2022.08.024. | David James Pinato |
| Funakoshi Y, Takigawa K, Hata N, et al. Changes in the Relapse Pattern and Prognosis of Glioblastoma After Approval of First-Line Bevacizumab: A Single-Center Retrospective Study. *World Neurosurg.* 2022 Mar;159:e479-e487. doi: 10.1016/j.wneu.2021.12.075. | Nobuhiro Hata |
| Funasaka C, Naito Y, Kusuhara S, et al. Clinical features of CDK4/6 inhibitor-related interstitial lung disease in patients with breast cancer: a case series study. *Jpn J Clin Oncol*. 2023 Jan 28;53(2):105-114. doi: 10.1093/jjco/hyac168. | Toru Mukohara |
| Fung AS, Afzal AR, Banerjee R, et al. A real-world comparison of cisplatin vs cetuximab used concurrently with radiation in the treatment of locally advanced oropharyngeal carcinoma. *Head Neck*. 2021 Jan;43(1):153-163. doi: 10.1002/hed.26469. | Desiree Hao |
| Fung AS, Tam VC, Meyers DE, et al. Second-line treatment of hepatocellular carcinoma after sorafenib: Characterizing treatments used over the past 10 years and real-world eligibility for cabozantinib, regorafenib, and ramucirumab. *Cancer Med*. 2020 Jul;9(13):4640-4647. doi: 10.1002/cam4.3116. | Richard Lee-Ying |
| Gadducci A, Cosio S, Lissoni AA, et al. Impact of Bevacizumab-containing Primary Treatment on Outcome of Recurrent Ovarian Cancer: An Italian Study. *Anticancer Res.* 2020 Mar;40(3):1543-1550. doi: 10.21873/anticanres.14100. | Angiolo Gadducci |
| Gaitonde P, Chirikov V, Kelkar S, Liljas B. Considerations for the Utility of Real-World Evidence Beyond Trial Data in Advanced NSCLC: The Case of Frontline Tyrosine Kinase Inhibitors. *Cancer Manag Res*. 2022 Dec 7;14:3421-3435. doi: 10.2147/CMAR.S380857. | Priyanka Gaitonde |
| Gallardo-Rincón D, Montes-Servín E, Alamilla-García G, et al. Clinical Benefits of Olaparib in Mexican Ovarian Cancer Patients With Founder Mutation *BRCA1*-Del ex9-12. *Front Genet.* 2022 Jun 6;13:863956. doi: 10.3389/fgene.2022.863956. | Dolores Gallardo-Rincón |
| Galvez-Nino M, Ruiz R, Roque K, et al. Real-world outcomes of anti-EGFR therapy in advanced non-small cell lung cancer EGFR mutated in Peru. *Thorac Cancer.* 2023 Jan;14(1):61-67. doi: 10.1111/1759-7714.14714. | Marco Galvez-Nino |
| Gan CL, Dudani S, Wells JC, et al. Cabozantinib real-world effectiveness in the first-through fourth-line settings for the treatment of metastatic renal cell carcinoma: Results from the International Metastatic Renal Cell Carcinoma Database Consortium. *Cancer Med*. 2021 Feb;10(4):1212-1221. doi: 10.1002/cam4.3717. | Daniel Y. C. Heng |
| Ganguly S, Mukherjee N, Mandal S, et al. Efficacy of cyclin-dependent kinase 4/6 inhibitors in patients with metastatic hormone positive breast cancer: a single institutional study from India. *Ecancermedicalscience.* 2022 Sep 26;16:1450. doi: 10.3332/ecancer.2022.1450. | Sandip Ganguly |
| Ganti AK, Lin CW, Yang E, et al. Real-world adherence and persistence with anaplastic lymphoma kinase inhibitors in non-small cell lung cancer. *J Manag Care Spec Pharm*. 2022 Mar;28(3):305-314. doi: 10.18553/jmcp.2021.21310. | Chia-Wei Lin |
| Gao C, Wang F, Suki D, et al. Effects of systemic therapy and local therapy on outcomes of 873 breast cancer patients with metastatic breast cancer to brain: MD Anderson Cancer Center experience. *Int J Cancer*. 2021 Feb 15;148(4):961-970. doi: 10.1002/ijc.33243. | Nuhad K. Ibrahim |
| Gao SL, Wang DY, Wang X, et al. Prognostic factors and adjuvant systemic therapy for patients with HER2-positive T1N0 breast cancer: evidence from a real-world study with long-term follow-up. *Breast Cancer Res Treat.* 2023 Feb;197(3):569-582. doi: 10.1007/s10549-022-06762-6. | Peng Yuan |
| Garg A, Batra U, Choudhary P, et al. Clinical predictors of response to EGFR-tyrosine kinase inhibitors in EGFR-mutated non-small cell lung cancer: A real-world multicentric cohort analysis from India. *Curr Probl Cancer*. 2020 Jun;44(3):100570. doi: 10.1016/j.currproblcancer.2020.100570. | Anant Mohan |
| Garg A, Iyer H, Jindal V, et al. Prognostic factors for treatment response and survival outcomes after first-line management of Stage 4 non-small cell lung cancer: A real-world Indian perspective. *Lung India.* 2022 Mar-Apr;39(2):102-109. doi: 10.4103/lungindia.lungindia_408_21. | Anant Mohan |
| Géczi L, Bodoky G, Rokszin G, et al. Survival Benefits of Second-line Axitinib Versus Everolimus After First Line Sunitinib Treatment in Metastatic Renal Cell Carcinoma. *Pathol Oncol Res*. 2020 Oct;26(4):2201-2207. doi: 10.1007/s12253-020-00809-z. | Lajos Géczi |
| Gemma A, Kusumoto M, Sakai F, et al. Real-World Evaluation of Factors for Interstitial Lung Disease Incidence and Radiologic Characteristics in Patients With EGFR T790M-positive NSCLC Treated With Osimertinib in Japan. *J Thorac Oncol.* 2020 Dec;15(12):1893-1906. doi: 10.1016/j.jtho.2020.08.025. | Akihiko Gemma |
| Gen S, Tanaka I, Morise M, et al. Clinical efficacy of osimertinib in EGFR-mutant non-small cell lung cancer with distant metastasis. *BMC Cancer*. 2022 Jun 14;22(1):654. doi: 10.1186/s12885-022-09741-8. | Ichidai Tanaka |
| Geng N, Ding CM, Liu ZK, et al. Influence of VEGFR2 gene polymorphism on the clinical outcomes of apatinib for patients with chemotherapy-refractory extensive-stage SCLC: a real-world retrospective study. *Int J Clin Oncol.* 2021 Apr;26(4):670-683. doi: 10.1007/s10147-020-01849-w. | Wen-Xia Hu |
| Gervaso L, Montero AJ, Jia X, Khorana AA. Venous thromboembolism in breast cancer patients receiving cyclin-dependent kinase inhibitors. *J Thromb Haemost*. 2020 Jan;18(1):162-168. doi: 10.1111/jth.14630. | Alok A. Khorana |
| Ghosh J, Joy Phillip DS, Ghosh J, et al. Survival outcomes with 12 weeks of adjuvant or neoadjuvant trastuzumab in breast cancer. *Indian J Cancer.* 2022 Jul-Sep;59(3):387-393. doi: 10.4103/ijc.IJC_850_19. | Jaya Ghosh |
| Giampieri R, Lupi A, Ziranu P, et al. Retrospective Comparative Analysis of KRAS G12C vs. Other KRAS Mutations in mCRC Patients Treated With First-Line Chemotherapy Doublet + Bevacizumab. *Front Oncol.* 2021 Sep 30;11:736104. doi: 10.3389/fonc.2021.736104. | Riccardo Giampieri |
| Gijtenbeek RGP, Damhuis RAM, Groen HJM, et al. Nationwide Real-world Cohort Study of First-line Tyrosine Kinase Inhibitor Treatment in Epidermal Growth Factor Receptor-mutated Non-small-cell Lung Cancer. *Clin Lung Cancer.* 2020 Nov;21(6):e647-e653. doi: 10.1016/j.cllc.2020.05.019. | Rolof G. P. Gijtenbeek |
| Gilbert A, Williams C, Azuero A, et al. Utilizing Data Visualization to Identify Survival and Treatment Differences Between Women With De Novo and Recurrent Metastatic Breast Cancer. *Clin Breast Cancer.* 2021 Aug;21(4):292-301. doi: 10.1016/j.clbc.2020.11.009. | Gabrielle Rocque |
| Girard N, Perol M, Simon G, et al. Treatment strategies for unresectable locally advanced non-small cell lung cancer in the real-life ESME cohort. *Lung Cancer.* 2021 Dec;162:119-127. doi: 10.1016/j.lungcan.2021.10.017. | Nicolas Girard |
| Girod M, Dalle S, Mortier L, et al. Non-V600E/K BRAF Mutations in Metastatic Melanoma: Molecular Description, Frequency, and Effectiveness of Targeted Therapy in a Large National Cohort. *JCO Precis Oncol.* 2022 Nov;6:e2200075. doi: 10.1200/PO.22.00075. | Olivier Dereure |
| Gleeson JP, Keane F, Keegan NM, et al. Similar overall survival with reduced vs. standard dose bevacizumab monotherapy in progressive glioblastoma. *Cancer Med.* 2020 Jan;9(2):469-475. doi: 10.1002/cam4.2616. | Patrick G. Morris |
| Goel S, Negassa A, Acuna-Villaorduna A. Comparative Effectiveness of Biologic Agents Among Black and White Medicare Patients in the US With Metastatic Colorectal Cancer. *JAMA Netw Open*. 2021 Dec 1;4(12):e2136378. doi: 10.1001/jamanetworkopen.2021.36378. | Sanjay Goel |
| Goh MJ, Kang W, Jeong WK, et al. Prognostic significance of cachexia index in patients with advanced hepatocellular carcinoma treated with systemic chemotherapy. *Sci Rep.* 2022 May 10;12(1):7647. doi: 10.1038/s41598-022-11736-1. | Wonseok Kang |
| Goh MJ, Oh JH, Park Y, et al. Efficacy and Safety of Lenvatinib Therapy for Unresectable Hepatocellular Carcinoma in a Real-World Practice in Korea. *Liver Cancer.* 2021 Feb;10(1):52-62. doi: 10.1159/000512239. | Wonseok Kang |
| Göksu SS, Tatlı AM, Geredeli Ç, et al. Cetuximab-induced rash is associated with overall survival in patients with recurrent/metastatic squamous cell carcinoma of head and neck. *Cancer Chemother Pharmacol*. 2021 Nov;88(5):805-812. doi: 10.1007/s00280-021-04328-9. | Sema Sezgin Göksu |
| Goldman A, Bomze D, Dankner R, et al. Cardiovascular Toxicities of Antiangiogenic Tyrosine Kinase Inhibitors: A Retrospective, Pharmacovigilance Study. *Target Oncol*. 2021 Jul;16(4):471-483. doi: 10.1007/s11523-021-00817-2. | Elad Maor |
| Gong IY, Yan AT, Earle CC, et al. Comparison of outcomes in a population-based cohort of metastatic breast cancer patients receiving anti-HER2 therapy with clinical trial outcomes. *Breast Cancer Res Treat*. 2020 May;181(1):155-165. doi: 10.1007/s10549-020-05614-5. | Kelvin K. W. Chan |
| González-Santiago S, Saura C, Ciruelos E, et al. Real-world effectiveness of dual HER2 blockade with pertuzumab and trastuzumab for neoadjuvant treatment of HER2-positive early breast cancer (The NEOPETRA Study). *Breast Cancer Res Treat.* 2020 Nov;184(2):469-479. doi: 10.1007/s10549-020-05866-1. | Santiago González-Santiago |
| Gopal V, Dubashi B, Kayal S, et al. Challenges in the Management of Lung Cancer: Real-World Experience from a Tertiary Center in South India. *South Asian J Cancer.* 2021 Dec 20;10(3):175-182. doi: 10.1055/s-0041-1733312. | Prasanth Ganesan |
| Graham J, Shah AY, Wells JC, et al. Outcomes of Patients with Metastatic Renal Cell Carcinoma Treated with Targeted Therapy After Immuno-oncology Checkpoint Inhibitors*. Eur Urol Oncol.* 2021 Feb;4(1):102-111. doi: 10.1016/j.euo.2019.11.001. | Daniel Y. C. Heng |
| Graham J, Wells JC, Dudani S, et al. Outcomes of patients with advanced non-clear cell renal cell carcinoma treated with first-line immune checkpoint inhibitor therapy. *Eur J Cancer.* 2022 Aug;171:124-132. doi: 10.1016/j.ejca.2022.05.002. | Jeffrey Graham |
| Grinda T, Antoine A, Jacot W, et al. Evolution of overall survival and receipt of new therapies by subtype among 20 446 metastatic breast cancer patients in the 2008-2017 ESME cohort. *ESMO Open*. 2021 Jun;6(3):100114. doi: 10.1016/j.esmoop.2021.100114. | Suzette Delaloge |
| Groenland SL, Geel DR, Janssen JM, et al. Exposure-Response Analyses of Anaplastic Lymphoma Kinase Inhibitors Crizotinib and Alectinib in Non-Small Cell Lung Cancer Patients. *Clin Pharmacol Ther*. 2021 Feb;109(2):394-402. doi: 10.1002/cpt.1989. | Stefanie L. Groenland |
| Grohé C, Wehler T, Dechow T, et al. Nintedanib plus docetaxel after progression on first-line immunochemotherapy in patients with lung adenocarcinoma: Cohort C of the non-interventional study, VARGADO. *Transl Lung Cancer Res*. 2022 Oct;11(10):2010-2021. doi: 10.21037/tlcr-21-1018. | Christian Grohé |
| Grünwald V, Chirovsky D, Cheung WY, et al. Global treatment patterns and outcomes among patients with recurrent and/or metastatic head and neck squamous cell carcinoma: Results of the GLANCE H&N study. *Oral Oncol.* 2020 Mar;102:104526. doi: 10.1016/j.oraloncology.2019.104526. | Viktor Grünwald |
| Gu T, Jiang A, Zhou C, et al. Adverse reactions associated with immune checkpoint inhibitors and bevacizumab: A pharmacovigilance analysis. *Int J Cancer*. 2023 Feb 1;152(3):480-495. doi: 10.1002/ijc.34332. | Jian Zhang |
| Guha A, Jain P, Fradley MG, et al. Cardiovascular adverse events associated with BRAF versus BRAF/MEK inhibitor: Cross-sectional and longitudinal analysis using two large national registries. *Cancer Med*. 2021 Jun;10(12):3862-3872. doi: 10.1002/cam4.3938. | Avirup Guha |
| Gullo G, Rubatto M, Fava P, et al. Cutaneous side effects and types of dermatological reactions in metastatic melanoma patients treated by immunotherapies or targeted therapies: A retrospective single center study. *Dermatol Ther.* 2022 Jun;35(6):e15492. doi: 10.1111/dth.15492. | Marco Rubatto |
| Guo DZ, Cheng JW, Yan JY, et al. Efficacy and safety of lenvatinib for preventing tumor recurrence after liver transplantation in hepatocellular carcinoma beyond the Milan criteria. *Ann Transl Med*. 2022 Oct;10(20):1091. doi: 10.21037/atm-22-1353. | Xin-Rong Yang |
| Guo LW, Li XG, Yang YS, et al. Large-scale genomic sequencing reveals adaptive opportunity of targeting mutated-PI3Kα in early and advanced HER2-positive breast cancer. *Clin Transl Med*. 2021 Nov;11(11):e589. doi: 10.1002/ctm2.589. | Xin Hu |
| Guo M, Shu Y, Chen G, et al. A real-world pharmacovigilance study of FDA adverse event reporting system (FAERS) events for niraparib. *Sci Rep*. 2022 Nov 29;12(1):20601. doi: 10.1038/s41598-022-23726-4. | Feie Li |
| Guo P, Pi X, Gao F, et al. Transarterial chemoembolization plus lenvatinib with or without programmed death-1 inhibitors for patients with unresectable hepatocellular carcinoma: A propensity score matching study. *Front Oncol.* 2022 Oct 20;12:945915. doi: 10.3389/fonc.2022.945915. | Wendong Cao |
| Gürbüz M, Akkuş E, Sakin A, et al. Combination of trastuzumab and taxane-containing intensified chemotherapy in first-line treatment of HER2-positive advanced gastric cancer. *Tumori.* 2021 Oct;107(5):416-423. doi: 10.1177/0300891620969823. | Mustafa Gürbüz |
| Ha MJ, Singareeka Raghavendra A, Kettner NM, et al. Palbociclib plus endocrine therapy significantly enhances overall survival of HR+/HER2- metastatic breast cancer patients compared to endocrine therapy alone in the second-line setting: A large institutional study. *Int J Cancer*. 2022 Jun 15;150(12):2025-2037. doi: 10.1002/ijc.33959. | Khandan Keyomarsi |
| Haist M, Stege H, Ebner R, et al. The Role of Treatment Sequencing with Immune-Checkpoint Inhibitors and BRAF/MEK Inhibitors for Response and Survival of Patients with BRAFV600-Mutant Metastatic Melanoma-A Retrospective, Real-World Cohort Study. *Cancers (Basel).* 2022 Apr 21;14(9):2082. doi: 10.3390/cancers14092082. | Maximilian Haist |
| Hajiev S, Allara E, Motedayеn Aval L, et al. Impact of age on sorafenib outcomes in hepatocellular carcinoma: an international cohort study. *Br J Cancer.* 2021 Jan;124(2):407-413. doi: 10.1038/s41416-020-01116-9. | Rohini Sharma |
| Han F, Tian H, Jin B, Chen G. Influence of GSTP1 Polymorphism on the Clinical Outcomes of Patients With Advanced NSCLC Receiving First-Line Bevacizumab-Based Regimen: A Real-World Retrospective Study. *Clin Med Insights Oncol.* 2021 Dec 13;15:11795549211059146. doi: 10.1177/11795549211059146. | Gang Chen |
| Han HS, Kim BJ, Jee HJ, et al. Ramucirumab plus paclitaxel as second-line treatment in patients with advanced gastric or gastroesophageal junction adenocarcinoma: a nationwide real-world outcomes in Korea study (KCSG-ST19-16). *Ther Adv Med Oncol.* 2021 Sep 18;13:17588359211042812. doi: 10.1177/17588359211042812. | Dae Young Zang |
| Han JM, Han HW, Yee J, et al. Factors affecting high-grade hepatotoxicity of tyrosine kinase inhibitors in cancer patients: a multi-center observational study. *Eur J Clin Pharmacol*. 2020 Aug;76(8):1183-1191. doi: 10.1007/s00228-020-02897-x. | Hye Sun Gwak |
| Han S, Choi HJ, Beom SH, et al. Treatment efficacy by hepatic arterial infusion chemotherapy vs. sorafenib after liver-directed concurrent chemoradiotherapy for advanced hepatocellular carcinoma. *J Cancer Res Clin Oncol.* 2021 Oct;147(10):3123-3133. doi: 10.1007/s00432-021-03632-4. | Beom Kyung Kim |
| Hanzel J, Kosir Bozic T, Stabuc B, Jansa R. Sorafenib for the treatment of hepatocellular carcinoma: a single-centre real-world study. *Radiol Oncol*. 2020 May 28;54(2):233-236. doi: 10.2478/raon-2020-0027. | Rado Jansa |
| Hao C, Bai X, Zhang J, et al. Real-world data for the renal safety of abemaciclib combined with bisphosphonate in HR+/HER2- advanced breast cancer. *Thorac Cancer.* 2023 Jan;14(1):68-72. doi: 10.1111/1759-7714.14715. | Zhongsheng Tong |
| Hassing CMS, Mejdahl MK, Lænkholm AV, et al. Benefit of adjuvant chemotherapy and trastuzumab in patients with HER2-positive, node-negative breast tumors ≤ 10 mm: a nationwide study. *Breast Cancer Res Treat.* 2022 Nov;196(1):197-206. doi: 10.1007/s10549-022-06724-y. | Christina M. S. Hassing |
| Hata N, Mizoguchi M, Kuga D, et al. First-line bevacizumab contributes to survival improvement in glioblastoma patients complementary to temozolomide. *J Neurooncol.* 2020 Feb;146(3):451-458. doi: 10.1007/s11060-019-03339-0. | Nobuhiro Hata |
| Hatanaka T, Hiraoka A, Tada T, et al. Association of early bevacizumab interruption with efficacy of atezolizumab plus bevacizumab for advanced hepatocellular carcinoma: A landmark analysis. *Hepatol Res.* 2022 May;52(5):462-470. doi: 10.1111/hepr.13748. | Takeshi Hatanaka |
| Hatanaka T, Kakizaki S, Hiraoka A, et al. Comparative efficacy and safety of atezolizumab and bevacizumab between hepatocellular carcinoma patients with viral and non-viral infection: A Japanese multicenter observational study. *Cancer Med.* 2023 Mar;12(5):5293-5303. doi: 10.1002/cam4.5337. | Takeshi Hatanaka |
| Hatanaka T, Kakizaki S, Hiraoka A, et al. Prognostic impact of C-reactive protein and alpha-fetoprotein in immunotherapy score in hepatocellular carcinoma patients treated with atezolizumab plus bevacizumab: a multicenter retrospective study. *Hepatol Int.* 2022 Oct;16(5):1150-1160. doi: 10.1007/s12072-022-10358-z. | Takeshi Hatanaka |
| Hatanaka T, Kakizaki S, Nagashima T, et al. A change in the timing for starting systemic therapies for hepatocellular carcinoma: the comparison of sorafenib and lenvatinib as the first-line treatment. *Acta Gastroenterol Belg*. 2021 Jan-Mar;84(1):65-72. doi: 10.51821/84.1.109. | Satoru Kakizaki |
| Hatanaka T, Kakizaki S, Nagashima T, et al. Lenvatinib for Hepatocellular Carcinoma Patients with Nonviral Infection Who Were Unlikely to Respond to Immunotherapy: A Retrospective, Comparative Study. *Oncology.* 2021;99(10):641-651. doi: 10.1159/000517494. | Takeshi Hatanaka |
| Hatanaka T, Kakizaki S, Nagashima T, et al. Liver Function Changes in Patients with Hepatocellular Carcinoma Treated with Lenvatinib: Predictive Factors of Progression to Child-Pugh Class B, the Formation of Ascites and the Candidates for the Post-Progression Treatment. *Cancers (Basel).* 2020 Oct 10;12(10):2906. doi: 10.3390/cancers12102906. | Takeshi Hatanaka |
| Hatanaka T, Naganuma A, Hiraoka A, et al. The hepatocellular carcinoma modified Gustave Roussy Immune score (HCC-GRIm score) as a novel prognostic score for patients treated with atezolizumab and bevacizumab: A multicenter retrospective analysis. *Cancer Med.* 2023 Feb;12(4):4259-4269. doi: 10.1002/cam4.5294. | Takeshi Hatanaka |
| Hatori M, Kawakami K, Wakatsuki T, et al. Association Between Regorafenib Dose and Efficacy Against Metastatic Colorectal Cancer in a Real-World Setting. *Dose Response.* 2021 Oct 12;19(4):15593258211047658. doi: 10.1177/15593258211047658. | Masahiro Hatori |
| Hawkins R, Fife K, Hurst M, et al. Treatment patterns and health outcomes in metastatic renal cell carcinoma patients treated with targeted systemic therapies in the UK. *BMC Cancer*. 2020 Jul 17;20(1):670. doi: 10.1186/s12885-020-07154-z. | Robert Hawkins |
| Hayes AR, Mak IYF, Evans N, et al. Understanding the Treatment Algorithm of Patients with Metastatic Pancreatic Neuroendocrine Neoplasms: A Single-Institution Retrospective Analysis Comparing Outcomes of Chemotherapy, Molecular Targeted Therapy, and Peptide Receptor Radionuclide Therapy in 255 Patients. *Neuroendocrinology.* 2021;111(9):863-875. doi: 10.1159/000511662. | Aimee R. Hayes |
| He MK, Liang RB, Zhao Y, et al. Lenvatinib, toripalimab, plus hepatic arterial infusion chemotherapy versus lenvatinib alone for advanced hepatocellular carcinoma. *Ther Adv Med Oncol*. 2021 Mar 25;13:17588359211002720. doi: 10.1177/17588359211002720. | Ming Shi |
| He Q, Chen J, Zhou K, et al. Effect of Additional Trastuzumab in Neoadjuvant and Adjuvant Treatment for Patients with Resectable HER2-Positive Gastric Cancer. *Ann Surg Oncol.* 2021 Aug;28(8):4413-4422. doi: 10.1245/s10434-020-09405-6. | Jiafu Ji |
| He Q, Liu J, Cai X, et al. Comparison of first-generation EGFR-TKIs (gefitinib, erlotinib, and icotinib) as adjuvant therapy in resected NSCLC patients with sensitive EGFR mutations. *Transl Lung Cancer Res*. 2021 Nov;10(11):4120-4129. doi: 10.21037/tlcr-21-649. | Wenhua Liang |
| He X, Dai X, Ji J, et al. Nine-Year Median Follow-up of Cardiotoxicity and Efficacy of Trastuzumab Concurrently With Anthracycline-Based and Anthracycline-Free Neoadjuvant Chemotherapy in HER2-Positive Breast Cancer Patients. *Clin Breast Cancer.* 2022 Jan;22(1):e80-e90. doi: 10.1016/j.clbc.2021.05.008. | Sai-Ching Jim Yeung |
| He X, Liu J, Xiao L, et al. Cisplatin-based chemotherapy with or without bevacizumab for Chinese postmenopausal women with advanced cervical cancer: a retrospective observational study. *BMC Cancer.* 2020 May 5;20(1):381. doi: 10.1186/s12885-020-06854-w. | Xiaoli He |
| Hecht M, Hahn D, Wolber P, et al. A Prospective Real-World Multi-Center Study to Evaluate Progression-Free and Overall Survival of Radiotherapy with Cetuximab and Platinum-Based Chemotherapy with Cetuximab in Locally Recurrent Head and Neck Cancer. *Cancers (Basel).* 2021 Jul 8;13(14):3413. doi: 10.3390/cancers13143413. | Markus Hecht |
| Hecht M, Hahn D, Wolber P, et al. Treatment response lowers tumor symptom burden in recurrent and/or metastatic head and neck cancer. *BMC Cancer.* 2020 Sep 29;20(1):933. doi: 10.1186/s12885-020-07440-w. | Markus Hecht |
| Heilig CE, Laßmann A, Mughal SS, et al. Gene expression-based prediction of pazopanib efficacy in sarcoma. *Eur J Cancer.* 2022 Sep;172:107-118. doi: 10.1016/j.ejca.2022.05.025. | Stefan Fröhling |
| Hellmund P, Schmitt J, Roessler M, et al. Targeted and Checkpoint Inhibitor Therapy of Metastatic Malignant Melanoma in Germany, 2000-2016. *Cancers (Basel).* 2020 Aug 20;12(9):2354. doi: 10.3390/cancers12092354. | Olaf Schoffer |
| Hellyer JA, White MN, Gardner RM, et al. Impact of Tumor Suppressor Gene Co-Mutations on Differential Response to EGFR TKI Therapy in EGFR L858R and Exon 19 Deletion Lung Cancer. *Clin Lung Cancer*. 2022 May;23(3):264-272. doi: 10.1016/j.cllc.2021.09.004. | Heather A. Wakelee |
| Henriksen JN, Bøttger P, Hermansen CK, et al. Pazopanib-Induced Liver Toxicity in Patients With Metastatic Renal Cell Carcinoma: Effect of UGT1A1 Polymorphism on Pazopanib Dose Reduction, Safety, and Patient Outcomes. *Clin Genitourin Cancer.* 2020 Feb;18(1):62-68.e2. doi: 10.1016/j.clgc.2019.09.013. | Frede Donskov |
| Henriksson M, Björnsson B, Sternby Eilard M, et al. Treatment patterns and survival in patients with hepatocellular carcinoma in the Swedish national registry SweLiv. *BJS Open.* 2020 Feb;4(1):109-117. doi: 10.1002/bjs5.50226. | Martin Henriksson |
| Hermansen CK, Donskov F. Outcomes based on age in patients with metastatic renal cell carcinoma treated with first line targeted therapy or checkpoint immunotherapy: Older patients more prone to toxicity. *J Geriatr Oncol.* 2021 Jun;12(5):827-833. doi: 10.1016/j.jgo.2020.12.008. | Frede Donskov |
| Hester A, Gaß P, Fasching PA, et al. Trastuzumab Biosimilars in the Therapy of Breast Cancer - "Real World" Experiences from four Bavarian University Breast Centres. *Geburtshilfe Frauenheilkd*. 2020 Sep;80(9):924-931. doi: 10.1055/a-1226-6666. | Anna Hester |
| Hiraoka A, Kumada T, Kariyama K, et al. Clinical importance of muscle volume in lenvatinib treatment for hepatocellular carcinoma: Analysis adjusted with inverse probability weighting. *J Gastroenterol Hepatol.* 2021 Jul;36(7):1812-1819. doi: 10.1111/jgh.15336. | Atsushi Hiraoka |
| Hiraoka A, Kumada T, Tada T, et al. Atezolizumab plus bevacizumab treatment for unresectable hepatocellular carcinoma: Early clinical experience. *Cancer Rep (Hoboken).* 2022 Feb;5(2):e1464. doi: 10.1002/cnr2.1464. | Atsushi Hiraoka |
| Hiraoka A, Kumada T, Tada T, et al. Clinical Predictor of Urinary Protein as Adverse Event Associated with Atezolizumab plus Bevacizumab Treatment for Unresectable Hepatocellular Carcinoma. *Oncology.* 2022;100(12):645-654. doi: 10.1159/000526521. | Atsushi Hiraoka |
| Hiraoka A, Kumada T, Tada T, et al. Efficacy of lenvatinib for unresectable hepatocellular carcinoma based on background liver disease etiology: multi-center retrospective study. *Sci Rep.* 2021 Aug 17;11(1):16663. doi: 10.1038/s41598-021-96089-x. | Atsushi Hiraoka |
| Hiraoka A, Tanizawa Y, Huang YJ, et al. Association of Albumin-Bilirubin Grade and Sequential Treatment with Standard Systemic Therapies for Advanced Hepatocellular Carcinoma: A Retrospective Cohort Study Using a Japanese Administrative Database. *Drugs Real World Outcomes*. 2021 Sep;8(3):301-314. doi: 10.1007/s40801-021-00245-8. | Yoshinori Tanizawa |
| Hizal M, Bilgin B, Paksoy N, et al. The percentage of ALK-positive cells and the efficacy of first-line alectinib in advanced non-small cell lung cancer: is it a novel factor for stratification? (Turkish Oncology Group Study). *J Cancer Res Clin Oncol.* 2023 Jul;149(8):4141-4148. doi: 10.1007/s00432-022-04252-2. | Mutlu Hizal |
| Hizal M, Bilgin B, Paksoy N, et al. The real-life efficacy and safety of osimertinib in pretreated advanced non-small cell lung cancer patients with T790M mutation: a Turkish Oncology Group Study. *J Cancer Res Clin Oncol*. 2022 Jun;148(6):1501-1508. doi: 10.1007/s00432-021-03748-7. | Mutlu Hizal |
| Ho CC, Wen PC, Yu WC, et al. Pre-existing chronic kidney disease and hypertension increased the risk of cardiotoxicity among colorectal cancer patients treated with anticancer drugs. *J Chin Med Assoc.* 2021 Sep 1;84(9):877-884. doi: 10.1097/JCMA.0000000000000590. | Chen-Chang Yang |
| Ho CM, Lee CH, Lee MC, et al. Survival After Treatable Hepatocellular Carcinoma Recurrence in Liver Recipients: A Nationwide Cohort Analysis. *Front Oncol.* 2021 Jan 28;10:616094. doi: 10.3389/fonc.2020.616094. | Jann-Yuan Wang |
| Hoeh B, Schmucker P, Klümper N, et al. Comparison of First-Line Anti-PD-1-Based Combination Therapies in Metastatic Renal-Cell Carcinoma: Real-World Experiences from a Retrospective, Multi-Institutional Cohort. *Urol Int.* 2022;106(11):1150-1157. doi: 10.1159/000521661. | Benedikt Hoeh |
| Hofheinz RD, Anchisi S, Grünberger B, et al. Real-World Evaluation of Quality of Life, Effectiveness, and Safety of Aflibercept Plus FOLFIRI in Patients with Metastatic Colorectal Cancer: The Prospective QoLiTrap Study. *Cancers (Basel).* 2022 Jul 20;14(14):3522. doi: 10.3390/cancers14143522. | Ralf-Dieter Hofheinz |
| Hong L, Lewis WE, Nilsson M, et al. Limited Benefit from the Addition of Immunotherapy to Chemotherapy in TKI-Refractory EGFR-Mutant Lung Adenocarcinoma. *Cancers (Basel).* 2022 Jul 17;14(14):3473. doi: 10.3390/cancers14143473. | Xiuning Le |
| Hong S, Daniels B, van Leeuwen MT, et al. Incidence and risk factors of hypertension therapy in Australian cancer patients treated with vascular signalling pathway inhibitors. *Discov Oncol.* 2022 Jan 20;13(1):6. doi: 10.1007/s12672-022-00468-3. | Soojung Hong |
| Hooks M, Sandhu G, Maganti T, et al. Incidental coronary calcium in cancer patients treated with anthracycline and/or trastuzumab. *Eur J Prev Cardiol.* 2022 Dec 7;29(17):2200-2210. doi: 10.1093/eurjpc/zwac185. | Prabhjot S. Nijjar |
| Horimoto Y, Ishizuka Y, Ueki Y, et al. Comparison of tumors with HER2 overexpression versus HER2 amplification in HER2-positive breast cancer patients. *BMC Cancer.* 2022 Mar 5;22(1):242. doi: 10.1186/s12885-022-09351-4. | Yoshiya Horimoto |
| Horita N, Miyagi E, Mizushima T, et al. Severe anaphylaxis caused by intravenous anti-cancer drugs. *Cancer Med.* 2021 Oct;10(20):7174-7183. doi: 10.1002/cam4.4252. | Nobuyuki Horita |
| Howell J, Samani A, Mannan B, et al. Impact of NAFLD on clinical outcomes in hepatocellular carcinoma treated with sorafenib: an international cohort study. *Therap Adv Gastroenterol.* 2022 Sep 30;15:17562848221100106. doi: 10.1177/17562848221100106. | Rohini Sharma |
| Howell SJ, Coe F, Wang X, et al. Carboplatin dose capping affects pCR rate in HER2-positive breast cancer patients treated with neoadjuvant Docetaxel, Carboplatin, Trastuzumab, Pertuzumab (TCHP). *Breast Cancer Res Treat*. 2020 Nov;184(2):481-489. doi: 10.1007/s10549-020-05868-z. | Maria Ekholm |
| Hsieh MC, Rau KM, Lin SE, et al. An Observational Study of Trifluridine/Tipiracil-Containing Regimen Versus Regorafenib-Containing Regimen in Patients With Metastatic Colorectal Cancer. *Front Oncol.* 2022 May 19;12:867546. doi: 10.3389/fonc.2022.867546. | Hsin-Pao Chen |
| Hsieh MH, Kao TY, Hsieh TH, et al. Prognostic roles of diabetes mellitus and hypertension in advanced hepatocellular carcinoma treated with sorafenib. *PLoS One*. 2020 Dec 31;15(12):e0244293. doi: 10.1371/journal.pone.0244293. | Jung-Ta Kao |
| Hsieh YY, Fang WT, Lo YW, et al. Comparing the effectiveness of different EGFR-TKIs in patients with EGFR mutant non-small-cell lung cancer: A retrospective cohort study in Taiwan. *Int J Cancer*. 2020 Aug 15;147(4):1107-1116. doi: 10.1002/ijc.32841. | Li-Nien Chien |
| Hsu HC, Huang KC, Chen WS, et al. Preference criteria for regorafenib in treating refractory metastatic colorectal cancer are the small tumor burden, slow growth and poor/scanty spread. *Sci Rep*. 2021 Jul 28;11(1):15370. doi: 10.1038/s41598-021-94968-x. | Hao-Wei Teng |
| Hsu HT, Yu CC, Lee YH, et al. Association between dermatologic adverse events and quality of life in lung cancer patients treated with epidermal growth factor receptor-tyrosine kinase inhibitors. *Support Care Cancer*. 2022 Nov;30(11):9211-9219. doi: 10.1007/s00520-022-07347-1. | Chia-Yu Chu |
| Hsu JC, Wei CF, Yang SC, et al. Lung cancer survival and mortality in Taiwan following the initial launch of targeted therapies: an interrupted time series study. *BMJ Open*. 2020 May 10;10(5):e033427. doi: 10.1136/bmjopen-2019-033427. | Jason C. Hsu |
| Hsu PC, Chang JW, Chang CF, et al. Sequential treatment in advanced non-small cell lung cancer harboring EGFR mutations. *Ther Adv Respir Dis.* 2022 Jan-Dec;16:17534666221132731. doi: 10.1177/17534666221132731. | Chiao-En Wu |
| Hu J, Chen Y, Zhu X, et al. Surgical choice of non-small cell lung cancer with unexpected pleural dissemination intraoperatively. *BMC Cancer.* 2021 Apr 22;21(1):445. doi: 10.1186/s12885-021-08180-1. | Peng Zhang |
| Hu W, Li B, Geng N, et al. Association Between PDL1 Genetic Variation and Efficacy of Apatinib Monotherapy in Patients with Previously Treated Advanced NSCLC: A Real-World Retrospective Study. *Int J Gen Med.* 2021 Jun 21;14:2703-2714. doi: 10.2147/IJGM.S303717. | Cuimin Ding |
| Huang AC, Huang CH, Ju JS, et al. First- or second-generation epidermal growth factor receptor tyrosine kinase inhibitors in a large, real-world cohort of patients with non-small cell lung cancer. *Ther Adv Med Oncol*. 2021 Jul 31;13:17588359211035710. doi: 10.1177/17588359211035710. | Chih-Hsi Scott Kuo |
| Huang C, Gu X, Zeng X, et al. Cetuximab versus bevacizumab following prior FOLFOXIRI and bevacizumab in postmenopausal women with advanced KRAS and BRAF wild-type colorectal cancer: a retrospective study. *BMC Cancer*. 2021 Jan 7;21(1):30. doi: 10.1186/s12885-020-07770-9. | Meiji Chen |
| Huang CH, Ju JS, Chiu TH, et al. Afatinib treatment in a large real-world cohort of nonsmall cell lung cancer patients with common and uncommon epidermal growth factor receptor mutation. *Int J Cancer.* 2022 Feb 15;150(4):626-635. doi: 10.1002/ijc.33821. | Chih-Hsi Scott Kuo |
| Huang J, Guo Y, Huang W, et al. Regorafenib Combined with PD-1 Blockade Immunotherapy versus Regorafenib as Second-Line Treatment for Advanced Hepatocellular Carcinoma: A Multicenter Retrospective Study. *J Hepatocell Carcinoma.* 2022 Mar 10;9:157-170. doi: 10.2147/JHC.S353956. | Kangshun Zhu |
| Huang J, Wang Y, Zhang C, et al. Surgical Management and Oncologic Outcomes for Local Retroperitoneal Recurrence of Renal Cell Carcinoma After Radical Nephrectomy. *Clin Genitourin Cancer.* 2023 Apr;21(2):315.e1-315.e7. doi: 10.1016/j.clgc.2022.10.010. | Zhisong He |
| Huang LY, Chang HP, Chang RY, et al. First-line treatment and overall survival in EGFR mutation-positive advanced non-small cell lung cancer: a national cohort study. *Eur Rev Med Pharmacol Sci.* 2022 Oct;26(20):7632-7640. doi: 10.26355/eurrev_202210_30039. | Po-Chang Lee |
| Huang MY, Hsieh KP, Huang RY, et al. Comparing survival and subsequent treatment of first-line tyrosine kinase inhibitors in patients of advanced lung adenocarcinoma with epidermal growth factor receptor mutation. *J Formos Med Assoc*. 2022 Jan;121(1 Pt 1):170-180. doi: 10.1016/j.jfma.2021.02.012. | Yi-Hsin Yang |
| Huang SC, Lin CC, Teng HW, et al. A Long-Term and Large-Scale Real-World Study in Taiwan: Efficacy of Target Therapy in Stage IV Colorectal Cancer. *Front Oncol.* 2022 Mar 17;12:808808. doi: 10.3389/fonc.2022.808808. | Jeng-Kai Jiang |
| Huang W, Zhang H, Tian Y, et al. Efficacy and safety analysis of bevacizumab combined with capecitabine in the maintenance treatment of RAS-mutant metastatic colorectal cancer. *J Clin Pharm Ther*. 2022 Apr;47(4):531-538. doi: 10.1111/jcpt.13576. | Xia Yuan |
| Huang YH, Hsu KH, Tseng JS, et al. The Difference in Clinical Outcomes Between Osimertinib and Afatinib for First-Line Treatment in Patients with Advanced and Recurrent EGFR-Mutant Non-Small Cell Lung Cancer in Taiwan. *Target Oncol*. 2022 May;17(3):295-306. doi: 10.1007/s11523-022-00878-x. | Gee-Chen Chang |
| Huang YH, Tseng JS, Hsu KH, et al. The impact of different first-line EGFR-TKIs on the clinical outcome of sequential osimertinib treatment in advanced NSCLC with secondary T790M. *Sci Rep*. 2021 Jun 8;11(1):12084. doi: 10.1038/s41598-021-91657-7. | Gee-Chen Chang |
| Huang Z, Zhou C, Xiong Y, et al. PD-1 inhibitor versus bevacizumab in combination with platinum-based chemotherapy for first-line treatment of advanced lung adenocarcinoma: A retrospective-real world study. *Front Oncol.* 2022 Nov 9;12:909721. doi: 10.3389/fonc.2022.909721. | Zhan Wang |
| Hue-Fontaine L, Lemelin A, Forestier J, et al. Metformin and everolimus in neuroendocrine tumours: A synergic effect? *Clin Res Hepatol Gastroenterol.* 2020 Nov;44(6):954-960. doi: 10.1016/j.clinre.2020.02.011. | Thomas Walter |
| Huemer F, Weiss L, Regitnig P, et al. Local and Central Evaluation of HER2 Positivity and Clinical Outcome in Advanced Gastric and Gastroesophageal Cancer-Results from the AGMT GASTRIC-5 Registry. *J Clin Med*. 2020 Mar 29;9(4):935. doi: 10.3390/jcm9040935. | Ewald Wöll |
| Hung CC, Tsai IC, Hsu CY, Lin HC. Clinical Outcomes of Neoadjuvant Therapy in Human Epidermal Growth Factor Receptor 2 Breast Cancer Patients: A Single-Center Retrospective Study. *J Clin Med*. 2022 Mar 5;11(5):1434. doi: 10.3390/jcm11051434. | Hsin-Chen Lin |
| Huntoon K, Damante M, Wang J, et al. Survival benefit with resection of brain metastases from renal cell carcinoma in the setting of molecular targeted therapy and/or immune therapy. *Curr Probl Cancer*. 2022 Apr;46(2):100805. doi: 10.1016/j.currproblcancer.2021.100805. | Kristin Huntoon |
| Hwang WY, Chang SJ, Kim HS, et al. Gastrointestinal/ genitourinary perforation and fistula formation with or without bevacizumab in patients with previously irradiated recurrent cervical cancer: a Korean multicenter retrospective study of the Gynecologic Oncology Research Investigators Collaboration (GORILLA) group (GORILLA-1001). *BMC Cancer*. 2022 Jun 2;22(1):603. doi: 10.1186/s12885-022-09695-x. | Dong Hoon Suh |
| Iavarone M, Invernizzi F, Ivanics T, et al. Regorafenib Efficacy After Sorafenib in Patients With Recurrent Hepatocellular Carcinoma After Liver Transplantation: A Retrospective Study. *Liver Transpl*. 2021 Dec;27(12):1767-1778. doi: 10.1002/lt.26264. | Massimo Iavarone |
| Ibragimova KIE, Geurts SME, Croes S, et al. Survival before and after the introduction of pertuzumab and T-DM1 in HER2-positive advanced breast cancer, a study of the SONABRE Registry. *Breast Cancer Res Treat*. 2021 Jul;188(2):571-581. doi: 10.1007/s10549-021-06178-8. | Vivianne C G Tjan-Heijnen |
| Ibrahim AM, Le May M, Bossé D, et al. Imaging Intensity and Survival Outcomes in High-Risk Resected Melanoma Treated by Systemic Therapy at Recurrence. *Ann Surg Oncol.* 2020 Oct;27(10):3683-3691. doi: 10.1245/s10434-020-08407-8. | Michael Ong |
| Ielasi L, Tovoli F, Tonnini M, et al. Beneficial Prognostic Effects of Aspirin in Patients Receiving Sorafenib for Hepatocellular Carcinoma: A Tale of Multiple Confounders. *Cancers (Basel).* 2021 Dec 20;13(24):6376. doi: 10.3390/cancers13246376. | Francesco Tovoli |
| Imamura F, Kimura M, Yano Y, et al. Real-world osimertinib for EGFR mutation-positive non-small-cell lung cancer with acquired T790M mutation. *Future Oncol.* 2020 Jul;16(21):1537-1547. doi: 10.2217/fon-2020-0203. | Fumio Imamura |
| Inanc M, Sirakaya HA, Karaman H, Bozkurt O. The prognostic importance of VEGF-A, PDGF-BB and c-MET in patients with metastatic colorectal cancer. *J Oncol Pharm Pract.* 2020 Dec;26(8):1878-1885. doi: 10.1177/1078155220904151. | Oktay Bozkurt |
| Ishihara H, Fukuda H, Takagi T, et al. Efficacy of nivolumab versus molecular-targeted therapy as second-line therapy for metastatic renal cell carcinoma: Real-world data from two Japanese institutions. *Int J Urol.* 2021 Jan;28(1):99-106. doi: 10.1111/iju.14412. | Tsunenori Kondo |
| Ishihara H, Nemoto Y, Nakamura K, et al. Changes in Real-World Outcomes in Patients with Metastatic Renal Cell Carcinoma from the Molecular-Targeted Therapy Era to the Immune Checkpoint Inhibitor Era. *Target Oncol.* 2022 May;17(3):307-319. doi: 10.1007/s11523-022-00879-w. | Hiroki Ishihara |
| Ishihara H, Tachibana H, Fukuda H, et al. Prognostic Impact of Trial-Eligibility Criteria in Patients with Metastatic Renal Cell Carcinoma. *Urol Int.* 2022;106(4):368-375. doi: 10.1159/000518162. | Hiroki Ishihara |
| Ishihara H, Takagi T, Kondo T, et al. Assessing improvements in metastatic renal cell carcinoma systemic treatments from the pre-cytokine to the immune checkpoint inhibitor eras: a retrospective analysis of real-world data. *Jpn J Clin Oncol.* 2021 Apr 30;51(5):793-801. doi: 10.1093/jjco/hyaa232. | Tsunenori Kondo |
| Ishihara H, Takagi T, Kondo T, et al. Prognostic impact of metastasectomy in renal cell carcinoma in the postcytokine therapy era. *Urol Oncol*. 2021 Jan;39(1):77.e17-77.e25. doi: 10.1016/j.urolonc.2020.08.011. | Toshio Takagi |
| Ishihara H, Takagi T, Kondo T, et al. Prognostic impact of systemic therapy change in metastatic renal cell carcinoma treated with cytoreductive nephrectomy. *Jpn J Clin Oncol.* 2021 Feb 8;51(2):296-304. doi: 10.1093/jjco/hyaa171. | Tsunenori Kondo |
| Ishikawa M, Iwasa S, Nagashima K, et al. Retrospective comparison of nab-paclitaxel plus ramucirumab and paclitaxel plus ramucirumab as second-line treatment for advanced gastric cancer focusing on peritoneal metastasis. *Invest New Drugs.* 2020 Apr;38(2):533-540. doi: 10.1007/s10637-019-00822-3. | Masashi Ishikawa |
| Ismail RK, van Breeschoten J, Wouters MWJM, et al. Palbociclib dose reductions and the effect on clinical outcomes in patients with advanced breast cancer. *Breast.* 2021 Dec;60:263-271. doi: 10.1016/j.breast.2021.11.013. | R. K. Ismail |
| Ito K, Morise M, Wakuda K, et al. A multicenter cohort study of osimertinib compared with afatinib as first-line treatment for EGFR-mutated non-small-cell lung cancer from practical dataset: CJLSG1903. *ESMO Open*. 2021 Jun;6(3):100115. doi: 10.1016/j.esmoop.2021.100115. | M. Morise |
| Ito K, Murotani K, Kubo A, et al. Propensity score analysis of overall survival between first- and second-generation EGFR-TKIs using real-world data. *Cancer Sci.* 2020 Oct;111(10):3705-3713. doi: 10.1111/cas.14560. | Tatsuya Yoshida |
| Ito K, Yamanaka T, Hayashi H, et al. Sequential therapy of crizotinib followed by alectinib for non-small cell lung cancer harbouring anaplastic lymphoma kinase rearrangement (WJOG9516L): A multicenter retrospective cohort study. *Eur J Cancer.* 2021 Mar;145:183-193. doi: 10.1016/j.ejca.2020.12.026. | Nobuyuki Yamamoto |
| Iwamoto H, Niizeki T, Nagamatsu H, et al. The Clinical Impact of Hepatic Arterial Infusion Chemotherapy New-FP for Hepatocellular Carcinoma with Preserved Liver Function. *Cancers (Basel).* 2022 Oct 5;14(19):4873. doi: 10.3390/cancers14194873. | Hideki Iwamoto |
| Iwasaki H, Toda S, Murayama D, et al. Relationship between adverse events associated with lenvatinib treatment for thyroid cancer and patient prognosis. *Mol Clin Oncol.* 2021 Feb;14(2):28. doi: 10.3892/mco.2020.2190. | Hiroyuki Iwasaki |
| Jacobse JN, Schaapveld M, Boekel NB, et al. Risk of heart failure after systemic treatment for early breast cancer: results of a cohort study. *Breast Cancer Res Treat*. 2021 Jan;185(1):205-214. doi: 10.1007/s10549-020-05930-w. | Flora E. van Leeuwen |
| Jácome AA, Kee B, Fogelman D, et al. FOLFOXIRI Versus Doublet Regimens in Right-Sided Metastatic Colorectal Cancer: Focus on Subsequent Therapies and Impact on Overall Survival. *Clin Colorectal Cancer.* 2020 Dec;19(4):248-255.e6. doi: 10.1016/j.clcc.2020.05.010. | Cathy Eng |
| Jacquin JP, Uwer L, Savignoni A, et al. Safety profile of subcutaneous trastuzumab in patients with HER2-positive early breast cancer: The French HERmione non-interventional prospective study. *Breast*. 2020 Feb;49:1-7. doi: 10.1016/j.breast.2019.10.002. | Yazid Belkacemi |
| Jahanzeb M, Lin HM, Pan X, et al. Real-World Treatment Patterns and Progression-Free Survival Associated with Anaplastic Lymphoma Kinase (ALK) Tyrosine Kinase Inhibitor Therapies for ALK+ Non-Small Cell Lung Cancer. *Oncologist.* 2020 Oct;25(10):867-877. doi: 10.1634/theoncologist.2020-0011. | Mohammad Jahanzeb |
| Jahanzeb M, Lin HM, Wu Y, et al. Real-World Efficacy and Tolerability of Brigatinib in Patients with Non-Small Cell Lung Cancer with Prior ALK-TKIs in the United States. *Oncologist.* 2022 Sep 2;27(9):790-798. doi: 10.1093/oncolo/oyac116. | Mohammad Jahanzeb |
| Janisch F, Hillemacher T, Fuehner C, et al. The impact of cytoreductive nephrectomy on survival outcomes in patients treated with tyrosine kinase inhibitors for metastatic renal cell carcinoma in a real-world cohort. *Urol Oncol*. 2020 Sep;38(9):739.e9-739.e15. doi: 10.1016/j.urolonc.2020.04.033. | Michael Rink |
| Janisch F, Kienapfel C, Fühner C, et al. Treatment and Outcome of Metastatic Renal Cell Carcinoma With Sarcomatoid Differentiation: A Single-Center, Real-World Analysis of Retrospective Data. *Front Surg.* 2021 Nov 18;8:763271. doi: 10.3389/fsurg.2021.763271. | Michael Rink |
| Jansen L, Boakye D, Alwers E, et al. Uptake Rates of Novel Therapies and Survival Among Privately Insured Versus Publicly Insured Patients With Colorectal Cancer in Germany. *J Natl Compr Canc Netw*. 2021 Feb 12;19(4):411-420. doi: 10.6004/jnccn.2020.7636. | Lina Jansen |
| Jefremow A, Wiesmueller M, Rouse RA, et al. Beyond the border: the use of lenvatinib in advanced hepatocellular carcinoma after different treatment lines: a retrospective analysis. *J Physiol Pharmacol*. 2020 Oct;71(5). doi: 10.26402/jpp.2020.5.11. | Andre Jefremow |
| Jemielita T, Widman L, Fox C, et al. Replication of Oncology Randomized Trial Results using Swedish Registry Real World-Data: A Feasibility Study. *Clin Pharmacol Ther.* 2021 Dec;110(6):1613-1621. doi: 10.1002/cpt.2424. | Thomas Jemielita |
| Jeong ISD, Mo H, Nguyen A, et al. Primary chemoradiation with cisplatin versus cetuximab for locally advanced head and neck cancer: a retrospective cohort study. *Exp Hematol Oncol*. 2020 Aug 5;9:19. doi: 10.1186/s40164-020-00175-1. | Huynh Cao |
| Ji L, Cheng L, Zhu X, et al. Risk and prognostic factors of breast cancer with liver metastases. *BMC Cancer.* 2021 Mar 6;21(1):238. doi: 10.1186/s12885-021-07968-5. | Zhonghua Wang |
| Jia J, Guo H, Zhao H, et al. Oncogenic gene transcripts detection by FISH on liquid-based cytology slides of 338 advanced lung cancer patients. *J Am Soc Cytopathol.* 2021 May-Jun;10(3):270-277. doi: 10.1016/j.jasc.2020.12.002. | ZhiHui Zhang, |
| Jiang N, Chen J, Jin Y. Two therapeutic regimens for advanced gastric carcinoma: DOS chemotherapy alone vs. trastuzumab combined with DOS. *J BUON.* 2021 May-Jun;26(3):904-910. | Yuan Jin |
| Jiang W, Shi H, Zhang L, et al. Responses to Targeted Therapy among Organs Affected by Metastasis in Patients with Renal Cell Carcinoma are Organ-Specific. *Urol J.* 2020 Oct 21;18(5):512-518. doi: 10.22037/uj.v16i7.6129. | Hongzhe Shi |
| Jiang Y, Chen W, Yu W, et al. Survival analysis of afatinib versus erlotinib for individuals with advanced del19 lung adenocarcinoma with asymptomatic brain metastasis after pemetrexed-cisplatin chemotherapy: a retrospective study. *J Int Med Res*. 2020 Aug;48(8):300060520937093. doi: 10.1177/0300060520937093. | Meiji Chen |
| Jimenez-Fonseca P, Carmona-Bayonas A, Martinez-Torron A, et al. External validity of clinical trials with diverse trastuzumab-based chemotherapy regimens in advanced gastroesophageal adenocarcinoma: data from the AGAMENON-SEOM registry. *Ther Adv Med Oncol.* 2021 Jun 17;13:17588359211019672. doi: 10.1177/17588359211019672. | Alberto Carmona-Bayonas |
| Jin Y, Li J, Shen L, et al. A multi-center effectiveness comparison study of fruquintinib with constructed external control cohort of other targeted kinase inhibitors using real-world data in third-line treatment of metastatic colorectal cancer. *Front Oncol.* 2022 Nov 24;12:1044328. doi: 10.3389/fonc.2022.1044328. | Shukui Qin |
| Jochems A, Bastiaannet E, Aarts MJB, et al. Outcomes for systemic therapy in older patients with metastatic melanoma: Results from the Dutch Melanoma Treatment Registry. *J Geriatr Oncol.* 2021 Sep;12(7):1031-1038. doi: 10.1016/j.jgo.2021.04.006. | Anouk Jochems |
| Joel A, Georgy JT, Thumaty DB, et al. Neoadjuvant chemotherapy with biosimilar trastuzumab in human epidermal growth factor receptor 2 overexpressed non-metastatic breast cancer: patterns of use and clinical outcomes in India. *Ecancermedicalscience.* 2021 Mar 19;15:1207. doi: 10.3332/ecancer.2021.1207. | Ashish Singh |
| Ju S, Zhou C, Yang C, et al. Apatinib Plus Camrelizumab With/Without Chemoembolization for Hepatocellular Carcinoma: A Real-World Experience of a Single Center. *Front Oncol.* 2022 Jan 31;11:835889. doi: 10.3389/fonc.2021.835889. | Bin Xiong |
| Jung HA, Woo SY, Lee SH, et al. The different central nervous system efficacy among gefitinib, erlotinib and afatinib in patients with epidermal growth factor receptor mutation-positive non-small cell lung cancer. *Transl Lung Cancer Res*. 2020 Oct;9(5):1749-1758. doi: 10.21037/tlcr-20-379. | Jong-Mu Sun |
| Jung J, Kim HY, Kim DG, et al. Sequential Treatment with an Immune Checkpoint Inhibitor Followed by a Small-Molecule Targeted Agent Increases Drug-Induced Pneumonitis. *Cancer Res Treat.* 2021 Jan;53(1):77-86. doi: 10.4143/crt.2020.543. | Youngjoo Lee |
| Jürgens H, Ojamaa K, Pokker H, et al. Changes in therapy and survival of metastatic renal cell carcinoma in Estonia. *BMC Cancer*. 2020 Mar 12;20(1):201. doi: 10.1186/s12885-020-6685-y. | Hannes Jürgens |
| Kähler KC, Gutzmer R, Meier F, et al. Early Exanthema Upon Vemurafenib Plus Cobimetinib Is Associated With a Favorable Treatment Outcome in Metastatic Melanoma: A Retrospective Multicenter DeCOG Study. *Front Oncol.* 2021 May 24;11:672172. doi: 10.3389/fonc.2021.672172. | Katharina C. Kähler |
| Kakuta T, Yabusaki H, Bamba T, et al. Efficacy and safety of ramucirumab plus paclitaxel therapy for advanced gastric cancer patients treated previously with docetaxel-containing chemotherapy. *Int J Clin Oncol.* 2021 Apr;26(4):684-693. doi: 10.1007/s10147-020-01845-0. | Tomoyuki Kakuta |
| Kalantzis I, Nonni A, Pavlakis K, et al. Clinicopathological differences and correlations between right and left colon cancer. *World J Clin Cases*. 2020 Apr 26;8(8):1424-1443. doi: 10.12998/wjcc.v8.i8.1424. | Ioannis Kalantzis |
| Kalchiem-Dekel O, Falcon CJ, Bestvina CM, et al. Brief Report: Chylothorax and Chylous Ascites During RET Tyrosine Kinase Inhibitor Therapy. *J Thorac Oncol*. 2022 Sep;17(9):1130-1136. doi: 10.1016/j.jtho.2022.06.008. | Jessica J. Lin |
| Kamiya-Matsuoka C, Hamza MA, de Groot JF. Impact of adverse events of bevacizumab on survival outcomes of patients with recurrent glioblastoma. *J Clin Neurosci*. 2020 Apr;74:36-40. doi: 10.1016/j.jocn.2020.01.066. | Carlos Kamiya-Matsuoka |
| Kanbayashi Y, Ishikawa T, Tabuchi Y, et al. Predictive factors for the development of proteinuria in cancer patients treated with bevacizumab, ramucirumab, and aflibercept: a single-institution retrospective analysis. *Sci Rep.* 2020 Feb 6;10(1):2011. doi: 10.1038/s41598-020-58994-5. | Yuko Kanbayashi |
| Kaneda H, Kizaki M, Ochi M, et al. Ceritinib in Japanese patients with anaplastic lymphoma kinase (ALK)+ non-small cell lung cancer: interim analysis results of a post-marketing surveillance study. *Sci Rep*. 2020 Oct 8;10(1):16773. doi: 10.1038/s41598-020-72863-1. | Shigemi Akatsu |
| Kang I, Lee JG, Choi SH, et al. Impact of everolimus on survival after liver transplantation for hepatocellular carcinoma. *Clin Mol Hepatol.* 2021 Oct;27(4):589-602. doi: 10.3350/cmh.2021.0038. | Dong Jin Joo |
| Kanjanapan Y, Lok SW, Gibbs P, et al. Impact of prior (neo)adjuvant trastuzumab (NAT) exposure on the efficacy of HER2-targeted therapy for metastatic breast cancer. *Breast Cancer Res Treat.* 2020 Nov;184(1):87-95. doi: 10.1007/s10549-020-05825-w. | Yada Kanjanapan |
| Kapała A, Surwiłło-Snarska A, Jodkiewicz M, Kawecki A. Nutritional Care in Patients with Head and Neck Cancer during Chemoradiotherapy (CRT) and Bioradiotherapy (BRT) Provides Better Compliance with the Treatment Plan. *Cancers (Basel).* 2021 May 21;13(11):2532. doi: 10.3390/cancers13112532. | Aleksandra Kapała |
| Karacin C, Bilgetekin I, Basal FB, Oksuzoglu OB. Prognostic Importance of Metastatic Site in Intermediate-risk Group Metastatic Renal Cell Cancer Treated with Tyrosine Kinase Inhibitors. *J Coll Physicians Surg Pak*. 2020 Jun;30(6):590-594. doi: 10.29271/jcpsp.2020.06.590. | Cengiz Karacin |
| Karivedu V, Bonomi M, Issa M, et al. Treatment Outcomes of Head and Neck Cancer Patients in the Elderly Receiving Different Chemoradiation Combinations: A Single-Center Experience. *Oncol Res Treat.* 2021;44(10):521-529. doi: 10.1159/000518548. | Marcelo Bonomi |
| Kariya S, Shimizu Y, Hanai N, et al. Effectiveness of nivolumab affected by prior cetuximab use and neck dissection in Japanese patients with recurrent or metastatic head and neck cancer: results from a retrospective observational study in a real-world setting. *Int J Clin Oncol.* 2021 Jun;26(6):1049-1056. doi: 10.1007/s10147-021-01900-4. | Akihiro Homma |
| Kariyama K, Hiraoka A, Kumada T, et al. Chronological change in serum albumin as a prognostic factor in patients with hepatocellular carcinoma treated with lenvatinib: proposal of albumin simplified grading based on the modified albumin-bilirubin score (ALBS grade). *J Gastroenterol.* 2022 Aug;57(8):581-586. doi: 10.1007/s00535-022-01883-7. | Kazuhiro Nouso |
| Kartolo A, Deluce J, Hopman WM, et al. Real-World Evidence of Systemic Therapy Sequencing on Overall Survival for Patients with Metastatic BRAF-Mutated Cutaneous Melanoma. *Curr Oncol.* 2022 Mar 1;29(3):1501-1513. doi: 10.3390/curroncol29030126. | John G. Lenehan |
| Kato R, Hayashi H, Chiba Y, et al. Propensity score-weighted analysis of chemotherapy after PD-1 inhibitors versus chemotherapy alone in patients with non-small cell lung cancer (WJOG10217L). *J Immunother Cancer.* 2020 Feb;8(1):e000350. doi: 10.1136/jitc-2019-000350. | Hidetoshi Hayashi |
| Kaufman PA, Hurvitz SA, O'Shaughnessy J, et al. Baseline characteristics and first-line treatment patterns in patients with HER2-positive metastatic breast cancer in the SystHERs registry. *Breast Cancer Res Treat.* 2021 Jul;188(1):179-190. doi: 10.1007/s10549-021-06103-z. | Peter A. Kaufman |
| Kawachi H, Tamiya M, Matsumoto K, et al. Efficacy and safety of ramucirumab and docetaxel in previously treated patients with squamous cell lung cancer: a multicenter retrospective cohort study. *Invest New Drugs*. 2022 Jun;40(3):634-642. doi: 10.1007/s10637-022-01214-w. | Hayato Kawachi |
| Kawai M, Takada M, Nakayama T, et al. Patient characteristics, treatment patterns, and outcomes of hormone receptor-positive, human epidermal growth factor receptor 2-negative advanced breast cancer patients prescribed cyclin-dependent kinase 4 and 6 inhibitors: large-scale data analysis using a Japanese claims database. *Breast Cancer Res Treat*. 2023 Jan;197(2):435-447. doi: 10.1007/s10549-022-06816-9. | Yoshinori Tanizawa |
| Kawakami T, Masuishi T, Kawamoto Y, et al. The survival benefit of increasing the number of active drugs for metastatic colorectal cancer: A multicenter retrospective study. *Cancer Med.* 2022 Jun;11(11):2184-2192. doi: 10.1002/cam4.4599. | Takeshi Kawakami |
| Kazama K, Shiozawa M, Numata M, et al. Comparison of safety and efficacy of fluorouracil + oxaliplatin + irinotecan (FOLFOXIRI) and modified FOLFOXIRI with bevacizumab for metastatic colorectal cancer: data from clinical practice*. Int J Colorectal Dis.* 2022 Feb;37(2):337-348. doi: 10.1007/s00384-021-04064-9. | Keisuke Kazama |
| Kegasawa T, Sakamori R, Maesaka K, et al. Lower Serum Sodium Levels Are Associated with the Therapeutic Effect of Sorafenib on Hepatocellular Carcinoma. *Dig Dis Sci.* 2021 May;66(5):1720-1729. doi: 10.1007/s10620-020-06380-6. | Tetsuo Takehara |
| Kelkar SS, Prabhu VS, Zhang J, et al. Treatment patterns and real-world clinical outcomes in patients with advanced endometrial cancer that are non-microsatellite instability high (non-MSI-high) or mismatch repair proficient (pMMR) in the United States. *Gynecol Oncol Rep.* 2022 Jun 17;42:101026. doi: 10.1016/j.gore.2022.101026. | Vimalanand S. Prabhu |
| Kesby NL, Papachristos AJ, Gild M, et al. Outcomes of Advanced Medullary Thyroid Carcinoma in the Era of Targeted Therapy. *Ann Surg Oncol.* 2022 Jan;29(1):64-71. doi: 10.1245/s10434-021-10980-5. | Anthony R. Glover |
| Khan J, Ullah A, Waheed A, et al. Gastrointestinal Stromal Tumors (GIST): A Population-Based Study Using the SEER Database, including Management and Recent Advances in Targeted Therapy. *Cancers (Basel).* 2022 Jul 28;14(15):3689. doi: 10.3390/cancers14153689. | Hector Mesa |
| Kido K, Hatakeyama S, Numakura K, et al. Comparison of nivolumab plus ipilimumab with tyrosine kinase inhibitors as first-line therapies for metastatic renal-cell carcinoma: a multicenter retrospective study. *Int J Clin Oncol*. 2021 Jan;26(1):154-162. doi: 10.1007/s10147-020-01797-5. | Shingo Hatakeyama |
| Kieler M, Unseld M, Bianconi D, et al. Impact of New Chemotherapy Regimens on the Treatment Landscape and Survival of Locally Advanced and Metastatic Pancreatic Cancer Patients. *J Clin Med.* 2020 Feb 28;9(3):648. doi: 10.3390/jcm9030648. | Gerald W. Prager |
| Kim BH, Cho MJ, Kwon J. Potential intrinsic subtype dependence on the association between metformin use and survival in surgically resected breast cancer: a Korean national population-based study. *Int J Clin Oncol.* 2021 Nov;26(11):2004-2016. doi: 10.1007/s10147-021-02005-8. | Jeanny Kwon |
| Kim BJ, Jee HJ, Rha SY, et al. Ramucirumab plus paclitaxel as a second-line treatment in HER2-positive gastric cancer: subgroup analysis of a nationwide, real-world study in Korea (KCSG-ST19-16). *Gastric Cancer.* 2022 May;25(3):609-618. doi: 10.1007/s10120-021-01276-4. | Dae Young Zang |
| Kim BK, Cheon J, Kim H, et al. Atezolizumab/Bevacizumab vs. Lenvatinib as First-Line Therapy for Unresectable Hepatocellular Carcinoma: A Real-World, Multi-Center Study. *Cancers (Basel).* 2022 Mar 29;14(7):1747. doi: 10.3390/cancers14071747. | Hong Jae Chon |
| Kim HD, Bang Y, Lee MA, et al. Regorafenib in patients with advanced Child-Pugh B hepatocellular carcinoma: A multicentre retrospective study. *Liver Int.* 2020 Oct;40(10):2544-2552. doi: 10.1111/liv.14573. | Changhoon Yoo |
| Kim HS, Yoo TK, Park WC, Chae BJ. The prognostic value of HER2 status and efficacy of anti-HER2 therapy in patients with HR-positive mucinous breast cancer: a nationwide study from the Korean Breast Cancer Society. *Breast Cancer Res Treat.* 2020 Apr;180(2):461-470. doi: 10.1007/s10549-020-05550-4. | Byung Joo Chae |
| Kim JH, Yoon S, Lee DH, et al. Real-world utility of next-generation sequencing for targeted gene analysis and its application to treatment in lung adenocarcinoma. *Cancer Med*. 2021 May;10(10):3197-3204. doi: 10.1002/cam4.3874. | Sang-We Kim |
| Kim JY, Nam SJ, Lee JE, et al. Real World Evidence of Neoadjuvant Docetaxel/Carboplatin/Trastuzumab/Pertuzumab (TCHP) in Patients with HER2-Positive Early or Locally Advanced Breast Cancer: A Single-Institutional Clinical Experience. *Cancer Res Treat*. 2022 Oct;54(4):1091-1098. doi: 10.4143/crt.2021.901. | Yeon Hee Park |
| Kim M, Jin M, Jeon MJ, et al. Lenvatinib Compared with Sorafenib as a First-Line Treatment for Radioactive Iodine-Refractory, Progressive, Differentiated Thyroid Carcinoma: Real-World Outcomes in a Multicenter Retrospective Cohort Study. *Thyroid*. 2023 Jan;33(1):91-99. doi: 10.1089/thy.2022.0054. | Won Gu Kim |
| Kim MH, Choi CM, Lee SY, et al. First-line Afatinib in Patients With Non-small-cell Lung Cancer With Uncommon EGFR Mutations in South Korea. *Anticancer Res*. 2022 Mar;42(3):1615-1622. doi: 10.21873/anticanres.15636. | Tae Won Jang |
| Kim S, Kim KH, Kim BK, et al. Lenvatinib is independently associated with the reduced risk of progressive disease when compared with sorafenib in patients with advanced hepatocellular carcinoma. *J Gastroenterol Hepatol*. 2021 May;36(5):1317-1325. doi: 10.1111/jgh.15355. | Seung Up Kim |
| Kim SH, Lee DE, Joung JY, et al. Survival prognoses of Heng intermediate-risk patients with metastatic renal cell carcinoma treated with immunotherapy or targeted therapy: A real-world, single-center retrospective study. *Investig Clin Urol*. 2020 Mar;61(2):146-157. doi: 10.4111/icu.2020.61.2.146. | Jinsoo Chung |
| Kim SI, Lim H, Kim HS, et al. Effect of BRCA1/2 Mutational Status on Survival Outcomes According to Secondary Cytoreductive Surgery and Maintenance Therapy in Platinum-Sensitive Relapsed Ovarian Cancer: A Real-World Evidence Study. *Cancer Res Treat.* 2023 Jan;55(1):245-257. doi: 10.4143/crt.2022.232. | Maria Lee |
| Kim T, Jang TW, Choi CM, et al. Sequential treatment of afatinib and osimertinib or other regimens in patients with advanced non-small-cell lung cancer harboring EGFR mutations: Results from a real-world study in South Korea. *Cancer Med*. 2021 Sep;10(17):5809-5822. doi: 10.1002/cam4.4127. | Tae Won Jang |
| Kim T, Lee SJ, Jang TW. Application of several machine learning algorithms for the prediction of afatinib treatment outcome in advanced-stage EGFR-mutated non-small-cell lung cancer. *Thorac Cancer.* 2022 Dec;13(23):3353-3361. doi: 10.1111/1759-7714.14694. | Tae-Won Jang |
| Kim YJ, Oremus M, Chen HH, et al. Factors affecting treatment selection and overall survival for first-line EGFR-tyrosine kinase inhibitor therapy in non-small-cell lung cancer. *J Comp Eff Res*. 2021 Feb;10(3):193-206. doi: 10.2217/cer-2020-0173. | Yong-Jin Kim |
| Kimura M, Usami E, Teramachi H, Yoshimura T. Elucidation of optimal proteinuria management based on the risk of ramucirumab-induced proteinuria. *Oncol Lett.* 2022 Feb;23(2):45. doi: 10.3892/ol.2021.13163. | Michio Kimura |
| Kimura M, Usami E, Teramachi H, Yoshimura T. Identifying optimal magnesium replenishment points based on risk of severe hypomagnesemia in colorectal cancer patients treated with cetuximab or panitumumab. *Cancer Chemother Pharmacol.* 2020 Sep;86(3):383-391. doi: 10.1007/s00280-020-04126-9. | Michio Kimura |
| King BH, Baumgartner JM, Kelly KJ, et al. Preoperative bevacizumab does not increase complications following cytoreductive surgery and hyperthermic intraperitoneal chemotherapy. *PLoS One.* 2020 Dec 3;15(12):e0243252. doi: 10.1371/journal.pone.0243252 | Jula Veerapong |
| King G, Ittershagen S, He L, et al. Treatment Patterns in US Patients Receiving First-Line and Second-Line Therapy for Metastatic Pancreatic Ductal Adenocarcinoma in the Real World. *Adv Ther.* 2022 Dec;39(12):5433-5452. doi: 10.1007/s12325-022-02317-9. | Reginald Villacorta |
| Kinget L, Roussel E, Verbiest A, et al. MicroRNAs Targeting HIF-2α, VEGFR1 and/or VEGFR2 as Potential Predictive Biomarkers for VEGFR Tyrosine Kinase and HIF-2α Inhibitors in Metastatic Clear-Cell Renal Cell Carcinoma. *Cancers (Basel).* 2021 Jun 21;13(12):3099. doi: 10.3390/cancers13123099. | Benoit Beuselinck |
| Kish JK, Chatterjee D, Wan Y, et al. Lenvatinib and Subsequent Therapy for Radioactive Iodine-Refractory Differentiated Thyroid Cancer: A Real-World Study of Clinical Effectiveness in the United States. *Adv Ther*. 2020 Jun;37(6):2841-2852. doi: 10.1007/s12325-020-01362-6. | Jonathan K. Kish |
| Knispel S, Gassenmaier M, Menzies AM, et al. Outcome of melanoma patients with elevated LDH treated with first-line targeted therapy or PD-1-based immune checkpoint inhibition. *Eur J Cancer.* 2021 May;148:61-75. doi: 10.1016/j.ejca.2021.01.034. | Lisa Zimmer |
| Knudsen ES, Schultz E, Hamilton D, et al. Real-World Experience with CDK4/6 Inhibitors for Metastatic HR+/HER2- Breast Cancer at a Single Cancer Center. Oncologist. 2022 Aug 5;27(8):646-654. doi: 10.1093/oncolo/oyac089. | Agnieszka K. Witkiewicz |
| Koca S, Beşiroğlu M, Özçelik M, et al. Pazopanib for metastatic soft-tissue sarcoma: A multicenter retrospective study. *J Oncol Pharm Pract.* 2021 Apr;27(3):541-546. doi: 10.1177/1078155220924075. | Sinan Koca |
| Koch C, Göller M, Schott E, et al. Combination of Sorafenib and Transarterial Chemoembolization in Selected Patients with Advanced-Stage Hepatocellular Carcinoma: A Retrospective Cohort Study at Three German Liver Centers. *Cancers (Basel).* 2021 Apr 28;13(9):2121. doi: 10.3390/cancers13092121. | Jörg Trojan |
| Koehler VF, Berg E, Adam P, et al. Real-World Efficacy and Safety of Multi-Tyrosine Kinase Inhibitors in Radioiodine Refractory Thyroid Cancer. *Thyroid*. 2021 Oct;31(10):1531-1541. doi: 10.1089/thy.2021.0091. | Matthias Kroiss |
| Kokkali S, Saloustros E, Stefanou D, et al. Front-Line Bevacizumab plus Chemotherapy with or without Maintenance Therapy for Metastatic Breast Cancer: An Observational Study by the Hellenic Oncology Research Group. *Curr Oncol.* 2022 Feb 17;29(2):1237-1251. doi: 10.3390/curroncol29020105. | Vassilis Georgoulias |
| Koo DH, Ryu MH, Lee MY, et al. Trends in Chemotherapy Patterns and Survival of Patients with Advanced Gastric Cancer over a 16-Year Period: Impact of Anti-HER2-Targeted Agent in the Real-World Setting. *Cancer Res Treat*. 2021 Apr;53(2):436-444. doi: 10.4143/crt.2020.725. | Yoon-Koo Kang |
| Koo J, Roh TH, Lee SR, et al. Whole-Brain Radiotherapy vs. Localized Radiotherapy after Resection of Brain Metastases in the Era of Targeted Therapy: A Retrospective Study. *Cancers (Basel)*. 2021 Sep 20;13(18):4711. doi: 10.3390/cancers13184711. | Tae Hoon Roh |
| Korpics MC, Turchan WT, Koshy M, Spiotto MT. Decreased overall survival in patients with locally advanced head and neck cancer receiving definitive radiotherapy and concurrent cetuximab: National Cancer Database analysis. *Head Neck.* 2022 Jul;44(7):1528-1544. doi: 10.1002/hed.27050. | Michael T. Spiotto |
| Koshkin VS, Henderson N, James M, et al. Efficacy of enfortumab vedotin in advanced urothelial cancer: Analysis from the Urothelial Cancer Network to Investigate Therapeutic Experiences (UNITE) study. *Cancer.* 2022 Mar 15;128(6):1194-1205. doi: 10.1002/cncr.34057. | Vadim S. Koshkin |
| Krasniqi E, Pizzuti L, Barchiesi G, et al. Impact of BMI on HER2+ metastatic breast cancer patients treated with pertuzumab and/or trastuzumab emtansine. Real-world evidence. *J Cell Physiol*. 2020 Nov;235(11):7900-7910. doi: 10.1002/jcp.29445. | Maddalena Barba |
| Kraus AL, Yu-Kite M, Mardekian J, et al. Real-World Data of Palbociclib in Combination With Endocrine Therapy for the Treatment of Metastatic Breast Cancer in Men. *Clin Pharmacol Ther.* 2022 Jan;111(1):302-309. doi: 10.1002/cpt.2454. | Albert L. Kraus |
| Kreft S, Glutsch V, Zaremba A, et al. MAPKinase inhibition after failure of immune checkpoint blockade in patients with advanced melanoma - An evaluation of the multicenter prospective skin cancer registry ADOREG. *Eur J Cancer.* 2022 May;167:32-41. doi: 10.1016/j.ejca.2022.02.023. | Bastian Schilling |
| Kristensen KB, Thomsen IMN, Berg T, et al. Dose modifications of ribociclib and endocrine therapy for treatment of ER+ HER2- metastatic breast cancer. *Breast Cancer Res Treat.* 2021 Aug;188(3):799-809. doi: 10.1007/s10549-021-06215-6. | Kristoffer B. Kristensen |
| Kroese TE, Takahashi Y, Lordick F, et al. Liver oligometastatic disease in synchronous metastatic gastric cancer patients: a nationwide population-based cohort study. *Eur J Cancer.* 2023 Jan;179:65-75. doi: 10.1016/j.ejca.2022.11.011. | Hanneke W. M. van Laarhoven |
| Kroeze SGC, Fritz C, Schaule J, et al. Continued versus Interrupted Targeted Therapy during Metastasis-Directed Stereotactic Radiotherapy: A Retrospective Multi-Center Safety and Efficacy Analysis. *Cancers (Basel).* 2021 Sep 24;13(19):4780. doi: 10.3390/cancers13194780. | Stephanie G. C. Kroeze |
| Kroeze SGC, Schaule J, Fritz C, et al. Metastasis directed stereotactic radiotherapy in NSCLC patients progressing under targeted- or immunotherapy: efficacy and safety reporting from the 'TOaSTT' database. *Radiat Oncol.* 2021 Jan 6;16(1):4. doi: 10.1186/s13014-020-01730-0. | Stephanie G. C. Kroeze |
| Kruger DT, Jansen MPHM, Konings IRHM, et al. High ctDNA molecule numbers relate with poor outcome in advanced ER+, HER2- postmenopausal breast cancer patients treated with everolimus and exemestane. *Mol Oncol.* 2020 Mar;14(3):490-503. doi: 10.1002/1878-0261.12617. | Epie Boven |
| Kuang J, Wan D, Wan P, Wu D. Efficacy of sorafenib combined with transcatheter hepatic arterial chemoembolization in treating intermediate-advanced hepatocellular carcinoma. *J BUON*. 2021 May-Jun;26(3):868-874. | Dehua Wu |
| Kudo K, Nishii K, Makimoto G, et al. First and repeat rebiopsy for detecting EGFR T790M mutation in non-small-cell lung cancer: CS-Lung-003 prospective observational registry study. *J Cancer Res Clin Oncol*. 2022 Aug;148(8):1869-1877. doi: 10.1007/s00432-021-03893-z. | Katsuyuki Hotta |
| Kunimasa K, Kamada R, Oka T, et al. Cardiac Adverse Events in EGFR-Mutated Non-Small Cell Lung Cancer Treated With Osimertinib. *JACC CardioOncol*. 2020 Mar 17;2(1):1-10. doi: 10.1016/j.jaccao.2020.02.003. | Toru Oka |
| Kuo CS, Chiu TH, Tung PH, et al. Afatinib Treatment Alone or with Bevacizumab in a Real-World Cohort of Non-Small Cell Lung Cancer Patients with Epidermal Growth Factor Receptor Mutation. *Cancers (Basel).* 2022 Jan 9;14(2):316. doi: 10.3390/cancers14020316. | Chin-Chou Wang |
| Kuo YH, Huang TH, Wang JH, et al. Well-Controlled Viremia Predicts the Outcome of Hepatocellular Carcinoma in Chronic Viral Hepatitis Patients Treated with Sorafenib. *Cancers (Basel).* 2022 Aug 17;14(16):3971. doi: 10.3390/cancers14163971. | Chao-Hung Hung |
| Kuo YH, Lu SN, Chen YY, et al. Real-World Lenvatinib Versus Sorafenib in Patients With Advanced Hepatocellular Carcinoma: A Propensity Score Matching Analysis. *Front Oncol.* 2021 Oct 25;11:737767. doi: 10.3389/fonc.2021.737767. | Jing-Houng Wang |
| Kuwano A, Yada M, Nagasawa S, et al. Hepatitis C virus eradication ameliorates the prognosis of advanced hepatocellular carcinoma treated with sorafenib. *J Viral Hepat*. 2022 Jul;29(7):543-550. doi: 10.1111/jvh.13681. | Masayoshi Yada |
| Kwok WC, Cheong TF, Chiang KY, et al. Clinical efficacy and safety of pemetrexed with or without either Bevacizumab or Pembrolizumab in patients with metastatic nonsquamous non-small cell carcinoma. *Asia Pac J Clin Oncol.* 2023 Feb;19(1):87-95. doi: 10.1111/ajco.13658. | Terence Chi Chun Tam |
| Kwok WC, Ho JCM, Lam DCL, et al. When compared to plasma-based detection, osimertinib-treated non-small cell lung cancer (NSCLC) with tissue rebiopsy-confirmed acquired T790M mutation is associated with better survival. *Asia Pac J Clin Oncol.* 2021 Apr;17(2):e35-e39. doi: 10.1111/ajco.13287. | Terence Chi Chun Tam |
| Kwok WC, Ho JCM, Tam TCC, et al. Serum protein level as a predictor of therapeutic response and adverse effects associated with afatinib use. *J Thorac Dis.* 2022 Jun;14(6):1880-1889. doi: 10.21037/jtd-21-1649. | David Chi Leung Lam |
| Kwok WC, Ho JCM, Tam TCC, et al. Survival benefits from afatinib compared with gefitinib and erlotinib among patients with common EGFR mutation in first-line setting. *Thorac Cancer.* 2022 Jul;13(14):2057-2063. doi: 10.1111/1759-7714.14528. | David Chi Leung Lam |
| Kwok WC, Lam DCL, Ip MSM, et al. Association of genetic polymorphisms of CYP3A4 and CYP2D6 with gefitinib-induced toxicities. *Anticancer Drugs.* 2022 Nov 1;33(10):1139-1144. doi: 10.1097/CAD.0000000000001360. | James Chung Man Ho |
| Laakmann E, Witzel I, Neunhöffer T, et al. Characteristics of patients with brain metastases from human epidermal growth factor receptor 2-positive breast cancer: subanalysis of Brain Metastases in Breast Cancer Registry. *ESMO Open.* 2022 Jun;7(3):100495. doi: 10.1016/j.esmoop.2022.100495. | Volkmar Müller |
| Labeur TA, Berhane S, Edeline J, et al. Improved survival prediction and comparison of prognostic models for patients with hepatocellular carcinoma treated with sorafenib. *Liver Int.* 2020 Jan;40(1):215-228. doi: 10.1111/liv.14270. | Philip J. Johnson |
| Lai E, Puzzoni M, Ziranu P, et al. Long Term Survival With Regorafenib: REALITY (Real Life in Italy) Trial - A GISCAD Study. *Clin Colorectal Cancer.* 2021 Dec;20(4):e253-e262. doi: 10.1016/j.clcc.2021.07.008. | Mario Scartozzi |
| Lai X, Wang A. Effects of Fufang Banmao Capsule Associated with Sorafenib on Liver Function, Immune Status, Quality of Life Improvement, and Survival in Patients with Advanced Hepatocellular Carcinoma: A Retrospective Cohort Study. *Comput Intell Neurosci*. 2022 Aug 23;2022:6336107. doi: 10.1155/2022/6336107. | Anmei Wang |
| Lamba N, Ott PA, Iorgulescu JB. Use of First-Line Immune Checkpoint Inhibitors and Association With Overall Survival Among Patients With Metastatic Melanoma in the Anti-PD-1 Era. *JAMA Netw Open*. 2022 Aug 1;5(8):e2225459. doi: 10.1001/jamanetworkopen.2022.25459. | J. Bryan Iorgulescu |
| Lamy FX, Batech M, Boutmy E, et al. Comparative effectiveness of weekly versus every-2-weeks cetuximab in metastatic colorectal cancer in a US-insured population. *J Comp Eff Res*. 2020 Nov;9(16):1117-1129. doi: 10.2217/cer-2020-0132. | Francois-Xavier Lamy |
| Larson KL, Huang B, Chen Q, et al. EGFR testing and erlotinib use in non-small cell lung cancer patients in Kentucky. *PLoS One.* 2020 Aug 18;15(8):e0237790. doi: 10.1371/journal.pone.0237790. | Jill M. Kolesar |
| Laru L, Ronkainen H, Vaarala MH. Transition to Targeted Therapies Improved the Prognosis and Increased the Utilization of Medical Treatments among Patients with Synchronous Metastatic Renal Cell Cancer. *Int J Surg Oncol.* 2021 Aug 12;2021:5237695. doi: 10.1155/2021/5237695. | Markku H. Vaarala |
| Laufer-Perl M, Mor L, Milwidsky A, et al. Cancer Therapeutics-Related Cardiac Dysfunction among Patients with Active Breast Cancer: A Cardio-Oncology Registry. *Isr Med Assoc J*. 2020 Sep;22(9):564-568. | Michal Laufer-Perl |
| Lau-Min KS, Li Y, Eads JR, et al. Association between timely targeted treatment and outcomes in patients with metastatic HER2-overexpressing gastroesophageal adenocarcinoma. *Cancer.* 2022 May 1;128(9):1853-1862. doi: 10.1002/cncr.34117. | Kelsey S. Lau-Min |
| Lauppe R, Nilsson FOL, Fues Wahl H, et al. Use of ALK-tyrosine kinase inhibitors (ALK TKI) in clinical practice, overall survival, and treatment duration - a Swedish nationwide retrospective study. *Acta Oncol*. 2022 Nov;61(11):1354-1361. doi: 10.1080/0284186X.2022.2133972. | Rosa Lauppe |
| Lavacchi D, Fancelli S, Roviello G, et al. Mutations matter: An observational study of the prognostic and predictive value of KRAS mutations in metastatic colorectal cancer. *Front Oncol.* 2022 Nov 29;12:1055019. doi: 10.3389/fonc.2022.1055019. | Serena Pillozzi |
| Lavery L, DiSogra K, Lea J, et al. Risk factors associated with palbociclib-induced neutropenia in patients with metastatic breast cancer. *Support Care Cancer.* 2022 Dec;30(12):9803-9809. doi: 10.1007/s00520-022-07400-z. | Lesli Lavery |
| Law JW, Mitra D, Kaplan HG, et al. Real-World Treatment Patterns and Clinical Effectiveness of Palbociclib Plus an Aromatase Inhibitor as First-Line Therapy in Advanced/Metastatic Breast Cancer: Analysis from the US Syapse Learning Health Network. *Curr Oncol*. 2022 Feb 12;29(2):1047-1061. doi: 10.3390/curroncol29020089. | Jeanna W. Law |
| Le D, Vargo C, Collins S, et al. Impact of Dose Intensity on Pathologic Complete Response Rate in HER2-Positive Breast Cancer Patients Receiving Neoadjuvant Docetaxel, Carboplatin, Trastuzumab and Pertuzumab (TCHP). *Target Oncol.* 2022 Mar;17(2):167-175. doi: 10.1007/s11523-022-00874-1. | Dat Le |
| Le Roy C, Vernerey D, Evin C, et al. Efficacy and Tolerance of Carboplatin plus Cetuximab (Simplified EXTREME Regimen) in Patients with Recurrent and/or Metastatic Head and Neck Squamous Cell Carcinoma. *Clin Oncol (R Coll Radiol).* 2022 Dec;34(12):e473-e481. doi: 10.1016/j.clon.2022.09.046. | F. Huguet |
| Leal LF, Laus AC, Cavagna R, et al. EGF+61 A>G polymorphism does not predict response to first-generation EGFR tyrosine kinase inhibitors in lung cancer patients. *Thorac Cancer*. 2020 Oct;11(10):2987-2992. doi: 10.1111/1759-7714.13628. | Rui M. Reis |
| Leblanc O, Vacher S, Lecerf C, et al. Biomarkers of cetuximab resistance in patients with head and neck squamous cell carcinoma. *Cancer Biol Med*. 2020 Feb 15;17(1):208-217. doi: 10.20892/j.issn.2095-3941.2019.0153. | Maud Kamal |
| Lee A, Larck C, Moore DC. Impact of obesity on safety outcomes and treatment modifications with ado-trastuzumab emtansine in breast cancer patients. *J Oncol Pharm Pract*. 2022 Jan;28(1):49-54. doi: 10.1177/1078155220982648. | Donald C. Moore |
| Lee CH, Chung J, Kwak C, et al. Targeted therapy response in early versus late recurrence of renal cell carcinoma after surgical treatment: A propensity score-matched study using the Korean Renal Cancer Study Group database. *Int J Urol.* 2021 Apr;28(4):417-423. doi: 10.1111/iju.14485. | Hong Koo Ha |
| Lee CH, Lee YB, Kim MA, et al. Effectiveness of nivolumab versus regorafenib in hepatocellular carcinoma patients who failed sorafenib treatment. *Clin Mol Hepatol*. 2020 Jul;26(3):328-339. doi: 10.3350/cmh.2019.0049n. | Yoon Jun Kim |
| Lee CH, Shen MC, Tsai MJ, et al. Proton pump inhibitors reduce the survival of advanced lung cancer patients with therapy of gefitinib or erlotinib. *Sci Rep*. 2022 Apr 29;12(1):7002. doi: 10.1038/s41598-022-10938-x. | Kun-Pin Hsieh |
| Lee CS, Ahmed I, Miao E, et al. A real world analysis of first line treatment of advanced EGFR mutated non-small cell lung cancer: A multi-center, retrospective study. *J Oncol Pharm Pract.* 2022 Jul;28(5):1140-1151. doi: 10.1177/10781552211020798. | Chung-Shien Lee |
| Lee IC, Lee PC, Chao Y, et al. Application and Impact of Antiviral Therapy for Patients with HBV-Related Hepatocellular Carcinoma Receiving Sorafenib and Lenvatinib Treatment. *Viruses*. 2022 Oct 26;14(11):2355. doi: 10.3390/v14112355. | Yi-Hsiang Huang |
| Lee J, Choi Y, Han J, et al. Osimertinib Improves Overall Survival in Patients With EGFR-Mutated NSCLC With Leptomeningeal Metastases Regardless of T790M Mutational Status. *J Thorac Oncol*. 2020 Nov;15(11):1758-1766. doi: 10.1016/j.jtho.2020.06.018. | Myung-Ju Ahn |
| Lee J, Han JW, Sung PS, et al. Comparative Analysis of Lenvatinib and Hepatic Arterial Infusion Chemotherapy in Unresectable Hepatocellular Carcinoma: A Multi-Center, Propensity Score Study. *J Clin Med.* 2021 Sep 7;10(18):4045. doi: 10.3390/jcm10184045. | Pil-Soo Sung |
| Lee J, Park HS, Won HS, et al. Real-World Clinical Data of Palbociclib in Asian Metastatic Breast Cancer Patients: Experiences from Eight Institutions. *Cancer Res Treat*. 2021 Apr;53(2):409-423. doi: 10.4143/crt.2020.451. | Jae Ho Byun |
| Lee JH, Kim EY, Park CK, et al. Real-World Study of Osimertinib in Korean Patients with Epidermal Growth Factor Receptor T790M Mutation-Positive Non-Small Cell Lung Cancer. *Cancer Res Treat.* 2023 Jan;55(1):112-122. doi: 10.4143/crt.2022.381. | Jae Cheol Lee |
| Lee JS, Yost SE, Li SM, et al. Genomic Markers of CDK 4/6 Inhibitor Resistance in Hormone Receptor Positive Metastatic Breast Cancer. *Cancers (Basel).* 2022 Jun 28;14(13):3159. doi: 10.3390/cancers14133159. | Yuan Yuan |
| Lee K, Noh E, Moon SJ, et al. Statin use in patients with hormone receptor-positive metastatic breast cancer treated with everolimus and exemestane. *Cancer Med.* 2023 Mar;12(5):5461-5470. doi: 10.1002/cam4.5369. | In Hae Park |
| Lee MJ, Chang SW, Kim JH, et al. Real-world systemic sequential therapy with sorafenib and regorafenib for advanced hepatocellular carcinoma: a multicenter retrospective study in Korea. *Invest New Drugs*. 2021 Feb;39(1):260-268. doi: 10.1007/s10637-020-00977-4. | Ji Hoon Kim |
| Lee MY, Yoon SY, Kim KH, et al. Pulmonary toxicities of molecular targeted antineoplastic agents: a single-center 10-year experience. *Korean J Intern Med*. 2021 May;36(3):689-698. doi: 10.3904/kjim.2020.295. | Jong-Ho Won |
| Lee SY, Choi CM, Chang YS, et al. Real-world experience of afatinib as first-line therapy for advanced EGFR mutation-positive non-small cell lung cancer in Korea*. Transl Lung Cancer Res*. 2021 Dec;10(12):4353-4367. doi: 10.21037/tlcr-21-501. | Tae Won Jang |
| Lee YC, Wang JH, Chen CH, et al. Sorafenib use in hepatitis B virus- or hepatitis C virus-related hepatocellular carcinoma: A propensity score matching study. *Kaohsiung J Med Sci.* 2021 Oct;37(10):894-902. doi: 10.1002/kjm2.12413. | Yuan-Hung Kuo |
| Lee YP, Jung HA, Lee MS, et al. Bevacizumab plus irinotecan with or without gamma knife radiosurgery after failure of concurrent chemo-radiotherapy for high-grade glioma*. J Neurooncol*. 2022 Feb;156(3):541-549. doi: 10.1007/s11060-021-03930-4. | Se-Hoon Lee |
| Lee YP, Lee MS, Kim H, et al. Real-World Evidence of Trastuzumab, Pertuzumab, and Docetaxel Combination as a First-Line Treatment for Korean Patients with HER2-Positive Metastatic Breast Cancer. *Cancer Res Treat*. 2022 Oct;54(4):1130-1137. doi: 10.4143/crt.2021.1103. | Yeon Hee Park |
| Lemelin A, Maucort-Boulch D, Castel-Kremer E, et al. Elderly Patients with Metastatic Neuroendocrine Tumors Are Undertreated and Have Shorter Survival: The LyREMeNET Study. *Neuroendocrinology.* 2020;110(7-8):653-661. doi: 10.1159/000503901. | Thomas Walter |
| Lester J, Escriu C, Khan S, et al. Retrospective analysis of real-world treatment patterns and clinical outcomes in patients with advanced non-small cell lung cancer starting first-line systemic therapy in the United Kingdom. *BMC Cancer*. 2021 May 7;21(1):515. doi: 10.1186/s12885-021-08096-w. | Jason Lester |
| Leyh C, Ehmer U, Roessler D, et al. Sorafenib Versus Lenvatinib-Based Sequential Systemic Therapy for Advanced Hepatocellular Carcinoma: A Real-World Analysis. *Cancers (Basel).* 2022 Apr 13;14(8):1975. doi: 10.3390/cancers14081975. | Christian M. Lange |
| Li C, Bian X, Liu Z, et al. Effectiveness and safety of pyrotinib-based therapy in patients with HER2-positive metastatic breast cancer: A real-world retrospective study. *Cancer Med*. 2021 Dec;10(23):8352-8364. doi: 10.1002/cam4.4335. | Zhiyong Yu |
| Li C, Nie W, Guo J, et al. Osimertinib alone as second-line treatment for brain metastases (BM) control may be more limited than for non-BM in advanced NSCLC patients with an acquired EGFR T790M mutation. *Respir Res.* 2021 May 11;22(1):145. doi: 10.1186/s12931-021-01741-9. | Xueyan Zhang |
| Li F, Xu F, Li J, et al. Pyrotinib versus trastuzumab emtansine for HER2-positive metastatic breast cancer after previous trastuzumab and lapatinib treatment: a real-world study. *Ann Transl Med.* 2021 Jan;9(2):103. doi: 10.21037/atm-20-4054. | Zefei Jiang |
| Li H, Rong X, Hu W, et al. Bevacizumab Combined with Corticosteroids Does Not Improve the Clinical Outcome of Nasopharyngeal Carcinoma Patients With Radiation-Induced Brain Necrosis. *Front Oncol.* 2021 Sep 28;11:746941. doi: 10.3389/fonc.2021.746941. | Yamei Tang |
| Li HS, Wang SZ, Xu HY, et al. Afatinib and Dacomitinib Efficacy, Safety, Progression Patterns, and Resistance Mechanisms in Patients with Non-Small Cell Lung Cancer Carrying Uncommon EGFR Mutations: A Comparative Cohort Study in China (AFANDA Study). *Cancers (Basel).* 2022 Oct 28;14(21):5307. doi: 10.3390/cancers14215307. | Yan Wang |
| Li J, Huang W, Piao M, et al. Efficacy of bevacizumab combined with temozolomide dose-dense regimen on recurrent glioma. *J BUON*. 2021 Jan-Feb;26(1):145-151. | Xin Zhu |
| Li J, Yue H, Yu H, et al. Patients with low nicotinamide N-methyltransferase expression benefit significantly from bevacizumab treatment in ovarian cancer. *BMC Cancer.* 2021 Jan 14;21(1):67. doi: 10.1186/s12885-021-07785-w. | Xiaohong Xue |
| Li JR, Wu MJ, Wang T, et al. A prognostic score model for predicting the survival benefits of patients undergoing sorafenib plus transarterial chemoembolization for hepatocellular carcinoma with portal vein invasion*. Abdom Radiol (NY).* 2021 May;46(5):1967-1976. doi: 10.1007/s00261-020-02897-6. | Ying-Qiang Zhang |
| Li K, Liao N, Chen B, et al. Genetic mutation profile of Chinese HER2-positive breast cancers and genetic predictors of responses to Neoadjuvant anti-HER2 therapy. *Breast Cancer Res Treat.* 2020 Sep;183(2):321-332. doi: 10.1007/s10549-020-05778-0. | Ning Liao |
| Li L, Chen M, Zheng S, et al. Clinical and Genetic Predictive Models for the Prediction of Pathological Complete Response to Optimize the Effectiveness for Trastuzumab Based Chemotherapy. *Front Oncol.* 2021 Jul 15;11:592393. doi: 10.3389/fonc.2021.592393. | Jiong Wu |
| Li M, Hou X, Zhou C, et al. Prevalence and Clinical Impact of Concomitant Mutations in Anaplastic Lymphoma Kinase Rearrangement Advanced Non-small-Cell Lung Cancer (Guangdong Association of Thoracic Oncology Study 1055). *Front Oncol.* 2020 Aug 21;10:1216. doi: 10.3389/fonc.2020.01216. | Likun Chen |
| Li Q, He Z, Guo Y, et al. Assessing the Validity of a *a priori* Patient-Trial Generalizability Score using Real-world Data from a Large Clinical Data Research Network: A Colorectal Cancer Clinical Trial Case Study. *AMIA Annu Symp Proc.* 2020 Mar 4;2019:1101-1110. | Jiang Bian |
| Li Q, Song T. Association Between Adjuvant Sorafenib and the Prognosis of Patients With Hepatocellular Carcinoma at a High Risk of Recurrence After Radical Resection. *Front Oncol.* 2021 Sep 23;11:633033. doi: 10.3389/fonc.2021.633033. | Tianqiang Song |
| Li Q, Wang Y, Zhu M, et al. Clinical observation of neoadjuvant chemotherapy with pyrotinib plus trastuzumab in HER2-positive breast cancer: a cohort study. *Gland Surg.* 2021 Dec;10(12):3389-3402. doi: 10.21037/gs-21-794. | Yuanting Gu |
| Li RR, Yin XL, Zeng DY, et al. Efficacy and safety of anti-PD-1 antibody plus regorafenib in refractory microsatellite stable metastatic colorectal cancer: a retrospective single-arm cohort study. *Ann Transl Med.* 2022 Aug;10(16):880. doi: 10.21037/atm-22-3690. | Zheng-Yang Liu |
| Li S, Mei J, Wang Q, et al. Transarterial infusion chemotherapy with FOLFOX for advanced hepatocellular carcinoma: a multi-center propensity score matched analysis of real-world practice. *Hepatobiliary Surg Nutr.* 2021 Oct;10(5):631-645. doi: 10.21037/hbsn.2020.03.14. | Rongping Guo. |
| Li S, Wu J, Huang O, et al. HER2 positivity is not associated with adverse prognosis in high-risk estrogen receptor-positive early breast cancer patients treated with chemotherapy and trastuzumab. *Breast.* 2020 Dec;54:235-241. doi: 10.1016/j.breast.2020.10.002. | Kunwei Shen |
| Li X, Chen G, Hu Y, et al. Caution the arrhythmia association with antibody-drug conjugates: a pharmacovigilance study. *Anticancer Drugs.* 2022 Jan 1;33(1):e228-e234. doi: 10.1097/CAD.0000000000001191. | Bin Zhao |
| Li X, Cheng Y, Zhu B, et al. Implication of *VEGFR2* Polymorphism on the Prognosis of Anlotinib Monotherapy for Patients With Treatment-Refractory Advanced NSCLC: An Exploratory Study. *Technol Cancer Res Treat.* 2022 Jan-Dec;21:15330338221080993. doi: 10.1177/15330338221080993. | Mu Hu |
| Li X, Fu Z, Chen X, et al. Efficacy and Safety of Lenvatinib Combined With PD-1 Inhibitors Plus TACE for Unresectable Hepatocellular Carcinoma Patients in China Real-World. *Front Oncol.* 2022 Jul 4;12:950266. doi: 10.3389/fonc.2022.950266. | Jian Zhai |
| Li X, Huang J, Qiu Y, et al. Pemetrexed-Platinum With or Without Bevacizumab for Chinese Chemo-Naive Advanced Lung Adenocarcinoma Patients: A Real-World Study. *Front Pharmacol.* 2021 May 7;12:649222. doi: 10.3389/fphar.2021.649222. | Bing Xia |
| Li Y, Gong C, Lu Q, et al. Real-World Data of Triplet Combination of Trastuzumab, Lapatinib, and Chemotherapy in HER2-Positive Metastatic Breast Cancer: A Multicenter Retrospective Study. *Front Oncol.* 2020 Mar 3;10:271. doi: 10.3389/fonc.2020.00271. | Biyun Wang |
| Li Y, Xie Y, Gong C, et al. Comparative Treatment Patterns and Outcomes of Fulvestrant versus Everolimus Plus Exemestane for Postmenopausal Metastatic Breast Cancer Resistant to Aromatase Inhibitors in Real-World Experience. *Ther Clin Risk Manag.* 2020 Jun 30;16:607-615. doi: 10.2147/TCRM.S255365. | Biyun Wang |
| Li Y, Zhang F, Yuan P, et al. High MAF of EGFR mutations and high ratio of T790M sensitizing mutations in ctDNA predict better third-generation TKI outcomes. *Thorac Cancer*. 2020 Jun;11(6):1503-1511. doi: 10.1111/1759-7714.13418. | Ying Jianming |
| Liang RB, Zhao Y, He MK, et al. Hepatic Arterial Infusion Chemotherapy of Oxaliplatin, Fluorouracil, and Leucovorin With or Without Sorafenib as Initial Treatment for Advanced Hepatocellular Carcinoma. *Front Oncol.* 2021 May 12;11:619461. doi: 10.3389/fonc.2021.619461. | Ming Shi |
| Liang SK, Keng LT, Chang CH, et al. Treatment Options of First-Line Tyrosine Kinase Inhibitors and Subsequent Systemic Chemotherapy Agents for Advanced EGFR Mutant Lung Adenocarcinoma Patients: Implications From Taiwan Cancer Registry Cohort. *Front Oncol.* 2021 Jan 8;10:590356. doi: 10.3389/fonc.2020.590356. | Meng-Rui Lee |
| Liao J, Liu C, Long Q, et al. Direct Comparison Between the Addition of Pembrolizumab or Bevacizumab for Chemotherapy-Based First-Line Treatment of Advanced Non-Squamous Non-Small Cell Lung Cancer Lacking Driver Mutations. *Front Oncol*. 2021 Sep 29;11:752545. doi: 10.3389/fonc.2021.752545. | Jialei Wang |
| Lien MY, Wang TH, Hsieh CY, et al. Both combined or sequential use with immune checkpoint inhibitors on cetuximab-treated patients with recurrent or metastatic head and neck squamous cell carcinoma improve the overall survival. *Oral Oncol.* 2021 Aug;119:105380. doi: 10.1016/j.oraloncology.2021.105380. | Jason Chia-Hsun Hsieh |
| Ligorio F, Zambelli L, Bottiglieri A, et al. Hormone receptor status influences the impact of body mass index and hyperglycemia on the risk of tumor relapse in early-stage HER2-positive breast cancer patients. *Ther Adv Med Oncol*. 2021 Apr 16;13:17588359211006960. doi: 10.1177/17588359211006960. | Claudio Vernieri |
| Lim DH, Casadei-Gardini A, Lee MA, et al. Prognostic implication of serum AFP in patients with hepatocellular carcinoma treated with regorafenib. *Future Oncol.* 2022 Sep;18(27):3021-3030. doi: 10.2217/fon-2022-0524. | Changhoon Yoo |
| Lim J, Kim HI, Kim E, et al. Variceal bleeding is aggravated by portal venous invasion of hepatocellular carcinoma: a matched nested case-control study. *BMC Cancer.* 2021 Jan 5;21(1):11. doi: 10.1186/s12885-020-07708-1. | Ju Hyun Shim |
| Lin C, Shi X, Zhao J, et al. Tumor Mutation Burden Correlates With Efficacy of Chemotherapy/Targeted Therapy in Advanced Non-Small Cell Lung Cancer. *Front Oncol.* 2020 Apr 29;10:480. doi: 10.3389/fonc.2020.00480. | Ying Jin |
| Lin GS, Wang WW, Lin H, Lin RS. Bevacizumab Combined with Intensity-Modulated Radiation Therapy on Cognitive and Coagulation Function in Postoperative Glioma Patients*. J Healthc Eng.* 2022 Mar 12;2022:9367919. doi: 10.1155/2022/9367919. | Rui-Sheng Lin |
| Lin HM, Pan X, Hou P, et al. Real-world treatment duration in ALK-positive non-small-cell lung cancer patients receiving brigatinib through the early access program. *Future Oncol*. 2020 May;16(15):1031-1041. doi: 10.2217/fon-2019-0849. | Huamao M. Lin |
| Lin J, McRoy L, Fisher MD, et al. Treatment patterns and clinical outcomes of palbociclib-based therapy received in US community oncology practices. *Future Oncol*. 2021 Mar;17(9):1001-1011. doi: 10.2217/fon-2020-0744. | Junji Lin |
| Lin PT, Teng W, Jeng WJ, et al. Add-on sorafenib is beneficial for hepatocellular carcinoma patients with transarterial chemoembolization refractoriness: a real-world experience. *Eur J Gastroenterol Hepatol*. 2020 Sep;32(9):1192-1199. doi: 10.1097/MEG.0000000000001637. | Chun-Yen Lin |
| Lin Y, Lin M, Zhang J, et al. Real-World Data of Pyrotinib-Based Therapy in Metastatic HER2-Positive Breast Cancer: Promising Efficacy in Lapatinib-Treated Patients and in Brain Metastasis. *Cancer Res Treat.* 2020 Oct;52(4):1059-1066. doi: 10.4143/crt.2019.633. | Xichun Hu |
| Liu C, Li T, Tao Z, et al. Clinical Outcomes of 130 Patients with Hormone Receptor-Positive and Human Epidermal Growth Factor Receptor 2-Negative Metastatic Breast Cancer Treated with Palbociclib plus Endocrine Therapy and Subsequent Therapy: A Real-World Single-Center Retrospective Study in China. *Med Sci Monit.* 2020 Nov 30;26:e927187. doi: 10.12659/MSM.927187. | Xichun Hu |
| Liu C, Liu C, Liao J, et al. Genetic correlation of crizotinib efficacy and resistance in ALK- rearranged non-small-cell lung cancer. *Lung Cancer*. 2022 Sep;171:18-25. doi: 10.1016/j.lungcan.2022.07.011. | Jialei Wang |
| Liu CM, Huang BS, Yen YH, et al. Concurrent Sorafenib and Radiotherapy versus Radiotherapy Alone for Locally Advanced Hepatocellular Carcinoma: A Propensity-Matched Analysis. *J Hepatocell Carcinoma*. 2021 Aug 18;8:963-973. doi: 10.2147/JHC.S323302. | Jen-Yu Cheng |
| Liu JY, Zhu BR, Wang YD, Sun X. The efficacy and safety of Apatinib mesylate in the treatment of metastatic osteosarcoma patients who progressed after standard therapy and the VEGFR2 gene polymorphism analysis. *Int J Clin Oncol*. 2020 Jun;25(6):1195-1205. doi: 10.1007/s10147-020-01644-7. | Jia-Yong Liu |
| Liu K, Wang Y, Ma L, et al. Efficacy and safety of a treatment in patients with locoregionally advanced nasopharyngeal carcinoma (LANC) involving carotid artery invasion. *Eur Arch Otorhinolaryngol.* 2022 Dec;279(12):5791-5799. doi: 10.1007/s00405-022-07446-z. | Xinxin Zhang |
| Liu L, Zhang Q, Geng J, et al. Comparison of radiofrequency ablation combined with sorafenib or sorafenib alone in patients with ECOG performance score 1: identifying optimal candidates. *Ann Transl Med.* 2020 May;8(9):583. doi: 10.21037/atm.2020.03.71. | Lei Liu |
| Liu Q, You N, Li J, et al. Camrelizumab Plus Sorafenib Versus Sorafenib Monotherapy for Advanced Hepatocellular Carcinoma: A Retrospective Analysis. *Front Oncol.* 2021 Oct 19;11:694409. doi: 10.3389/fonc.2021.694409. | Lu Zheng |
| Liu S, Jiang C, Yang L, et al. First-line cetuximab improves the efficacy of subsequent bevacizumab for RAS wild-type left-sided metastatic colorectal cancer: an observational retrospective study. *Sci Rep*. 2020 Jul 23;10(1):12336. doi: 10.1038/s41598-020-69230-5. | Liangping Xia |
| Liu X, Hong L, Nilsson M, et al. Concurrent use of aspirin with osimertinib is associated with improved survival in advanced EGFR-mutant non-small cell lung cancer. *Lung Cancer.* 2020 Nov;149:33-40. doi: 10.1016/j.lungcan.2020.08.023. | Jianjun Zhang |
| Liu X, Tao L, Wang M, et al. ABSDELL Model: Development and Internal Validation of a Risk Prediction Model of LVEF Decline in Breast Cancer Patients Treated With Trastuzumab. *Clin Breast Cancer.* 2023 Jan;23(1):23-31. doi: 10.1016/j.clbc.2022.10.010. | Weixian Xu |
| Liu X, Zheng D, Wu Y, et al. Treatment patterns and outcomes in older women with early breast cancer: a population-based cohort study in China. *BMC Cancer.* 2021 Mar 5;21(1):226. doi: 10.1186/s12885-021-07947-w. | Hong Zheng |
| Liu Y, Wang H, Yang S, et al. *EGFR* mutation types and abundance were associated with the overall survival of advanced lung adenocarcinoma patients receiving first-line tyrosine kinase inhibitors. *J Thorac Dis.* 2022 Jun;14(6):2254-2267. doi: 10.21037/jtd-22-755. | Hong Ge |
| Liu Z, Guan Y, Yao Y, et al. Effectiveness and Safety of Zercepac and Reference Trastuzumab in the Neoadjuvant Setting for Early-Stage Breast Cancer: A Retrospective Cohort Study. *J Oncol.* 2022 Nov 3;2022:9998114. doi: 10.1155/2022/9998114. | Yongzhong Yao |
| Long Q, Feng Y, Liu C, et al. The optimal timing and courses of bevacizumab added to chemotherapy for non-squamous non-small cell lung cancer: revelations from the real-world experience in a single Chinese cancer center. *Ann Transl Med.* 2020 Oct;8(20):1311. doi: 10.21037/atm-20-6327. | Xinmin Zhao |
| Longo F, Jorge M, Yaya R, et al. Real-life use of ramucirumab in gastric cancer in Spain: the RAMIS study. *Future Oncol.* 2021 May;17(14):1777-1791. doi: 10.2217/fon-2020-1216. | Silvia Díaz-Cerezo |
| Loo Gan C, Huang J, Pan E, et al. Real-world Practice Patterns and Safety of Concurrent Radiotherapy and Cabozantinib in Metastatic Renal Cell Carcinoma: Results from the International Metastatic Renal Cell Carcinoma Database Consortium. *Eur Urol Oncol.* 2023 Apr;6(2):204-211. doi: 10.1016/j.euo.2022.10.004. | Rana R. McKay |
| Lorenzi M, Ferro A, Cecere F, et al. First-Line Osimertinib in Patients with EGFR-Mutant Advanced Non-Small Cell Lung Cancer: Outcome and Safety in the Real World: FLOWER Study. *Oncologist.* 2022 Mar 4;27(2):87-e115. doi: 10.1002/onco.13951. | Giulia Pasello |
| Lorusso D, Marchetti C, Conte C, et al. Bevacizumab as maintenance treatment in BRCA mutated patients with advanced ovarian cancer: A large, retrospective, multicenter case-control study. *Gynecol Oncol*. 2020 Oct;159(1):95-100. doi: 10.1016/j.ygyno.2020.07.022. | G. Scambia |
| Lu Y, Gehr AW, Anikpo I, et al. Cardiotoxicity among socioeconomically marginalized breast cancer patients. *Breast Cancer Res Treat.* 2022 Oct;195(3):401-411. doi: 10.1007/s10549-022-06695-0. | Rohit P. Ojha |
| Lübbers K, Pavlychenko M, Wald T, et al. Choosing the Right Treatment Option for the Right R/M HNSCC Patient: Should We Adhere to PFE for First-Line Therapy? *Front Oncol*. 2021 Jul 20;11:715297. doi: 10.3389/fonc.2021.715297. | Gunnar Wichmann |
| Luger AL, König S, Samp PF, et al. Molecular matched targeted therapies for primary brain tumors-a single center retrospective analysis. *J Neurooncol*. 2022 Sep;159(2):243-259. doi: 10.1007/s11060-022-04049-w. | Anna-Luisa Luger |
| Lugtenberg RT, Boers-Doets CB, Witteveen PO, et al. Prospective practice survey of management of cetuximab-related skin reactions. *Support Care Cancer.* 2021 Jul;29(7):3497-3506. doi: 10.1007/s00520-020-05862-7. | R. T. Lugtenberg |
| Luo YH, Liu H, Wampfler JA, et al. Real-world efficacy of osimertinib in previously EGFR-TKI treated NSCLC patients without identification of T790M mutation. *J Cancer Res Clin Oncol.* 2022 Aug;148(8):2099-2114. doi: 10.1007/s00432-021-03766-5. | Ping Yang |
| Lv C, Ma Y, Feng Q, et al. Does neoadjuvant targeted therapy provide an opportunity for resectable EGFR-mutant lung cancer: a real-world retrospective study. *J Thorac Dis.* 2020 Oct;12(10):5324-5335. doi: 10.21037/jtd-20-1265. | Yue Yang |
| Lv W, Cheng H, Shao D, et al. Treatment Patterns and Survival of Patients With Advanced Non-Small Cell Lung Cancer Guided by Comprehensive Genomic Profiling: Real-World Single-Institute Study in China. *Front Oncol.* 2021 Mar 10;11:630717. doi: 10.3389/fonc.2021.630717. | Xiaofeng Pei |
| M SG, Mohapatra PR, Bhuniya S, et al. Impact of Comorbidity Scores on the Overall Survival of Patients With Advanced Non-small Cell Lung Cancer: A Real-World Experience From Eastern India. *Cureus.* 2022 Oct 22;14(10):e30589. doi: 10.7759/cureus.30589. | Prasanta R. Mohapatra |
| Ma X, Yang S, Zhang K, et al. Efficacy of different sequential patterns after crizotinib progression in advanced anaplastic lymphoma kinase-positive non-small cell lung cancer. *Thorac Cancer.* 2022 Jun;13(12):1788-1794. doi: 10.1111/1759-7714.14455. | Xiaoqing Liu |
| Ma X, Zhang X, Zhou X, et al. Real-world study of trastuzumab and pertuzumab combined with chemotherapy in neoadjuvant treatment for patients with HER2-positive breast cancer. *Medicine (Baltimore).* 2022 Oct 7;101(40):e30892. doi: 10.1097/MD.0000000000030892. | Yunjiang Liu |
| Ma Y, Liu G, Yang J, et al. The Efficacy of Combined Therapy of Regorafenib with Detoxicating and Stasis Softening Chinese Herbal Spleen Tonics in Mid-/Late-Stage Hepatocellular Carcinoma. *Contrast Media Mol Imaging*. 2022 Jun 6;2022:9316873. doi: 10.1155/2022/9316873. | Baocheng Zhao |
| Ma Z, Pei J, Zhang Y, et al. Interstitial pneumonitis associated with EGFR/ ALK tyrosine kinase inhibitors used in non-small cell lung cancer: an observational, retrospective, pharmacovigilance study. *Expert Opin Drug Saf.* 2023 Mar;22(3):237-242. doi: 10.1080/14740338.2022.2110235. | Zhuoling An |
| Maahs L, Ghanem AI, Gutta R, et al. Cetuximab and anemia prevention in head and neck cancer patients undergoing radiotherapy. *BMC Cancer.* 2022 Jun 7;22(1):626. doi: 10.1186/s12885-022-09708-9. | Jawad Sheqwara |
| Maccali C, Chagas AL, Boin I, et al. Recurrence of hepatocellular carcinoma after liver transplantation: Prognostic and predictive factors of survival in a Latin American cohort. *Liver Int.* 2021 Apr;41(4):851-862. doi: 10.1111/liv.14736. | Frederico Piñero |
| Madison CJ, Melson RA, Conlin MJ, et al. Thromboembolic risk in patients with lung cancer receiving systemic therapy. *Br J Haematol*. 2021 Jul;194(1):179-190. doi: 10.1111/bjh.17476. | David C. Calverley |
| Magios N, Bozorgmehr F, Volckmar AL, et al. Real-world implementation of sequential targeted therapies for EGFR-mutated lung cancer. *Ther Adv Med Oncol*. 2021 Mar 24;13:1758835921996509. doi: 10.1177/1758835921996509. | Petros Christopoulos |
| Magnes T, Wagner SM, Melchardt T, et al. Postoperative chemoradiotherapy with cisplatin is superior to radioimmunotherapy with cetuximab and radiotherapy alone : Analysis of the Austrian head and neck cancer registry of the AGMT. *Wien Klin Wochenschr.* 2021 Nov;133(21-22):1131-1136. doi: 10.1007/s00508-021-01939-3. | Richard Greil |
| Mähringer-Kunz A, Steinle V, Kloeckner R, et al. The impact of portal vein tumor thrombosis on survival in patients with hepatocellular carcinoma treated with different therapies: A cohort study. *PLoS One.* 2021 May 7;16(5):e0249426. doi: 10.1371/journal.pone.0249426. | Aline Mähringer-Kunz |
| Maniakas A, Dadu R, Busaidy NL, et al. Evaluation of Overall Survival in Patients With Anaplastic Thyroid Carcinoma, 2000-2019. *JAMA Oncol*. 2020 Sep 1;6(9):1397-1404. doi: 10.1001/jamaoncol.2020.3362. | Maria E. Cabanillas |
| Manso L, Hernando C, Galán M, et al. Palbociclib combined with endocrine therapy in heavily pretreated HR+/HER2- advanced breast cancer patients: Results from the compassionate use program in Spain (PALBOCOMP). *Breast*. 2020 Dec;54:286-292. doi: 10.1016/j.breast.2020.11.005. | Fernando Moreno |
| Marasco G, Colecchia A, Bacchi Reggiani ML, et al. Comparison of prognostic models in advanced hepatocellular carcinoma patients undergoing Sorafenib: A multicenter study. *Dig Liver Dis*. 2021 Aug;53(8):1011-1019. doi: 10.1016/j.dld.2020.12.001. | Giovanni Marasco |
| Marasco G, Poggioli F, Colecchia A, et al. A Nomogram-Based Prognostic Model for Advanced Hepatocellular Carcinoma Patients Treated with Sorafenib: A Multicenter Study. *Cancers (Basel).* 2021 May 29;13(11):2677. doi: 10.3390/cancers13112677. | Giovanni Marasco |
| Marczyk VR, Rosa DD, Maia AL, Goemann IM. Overall Survival for HER2-Positive Breast Cancer Patients in the HER2-Targeted Era: Evidence From a Population-Based Study. *Clin Breast Cancer.* 2022 Jul;22(5):418-423. doi: 10.1016/j.clbc.2022.03.004. | Iuri Martin Goemann |
| Marqueen KE, Moshier E, Buckstein M, Ang C. Neoadjuvant therapy for gastrointestinal stromal tumors: A propensity score-weighted analysis. *Int J Cancer.* 2021 Jul 1;149(1):177-185. doi: 10.1002/ijc.33536. | Celina Ang |
| Marques RP, Godinho AR, Heudtlass P, et al. Cetuximab versus bevacizumab in metastatic colorectal cancer: a comparative effectiveness study. *J Cancer Res Clin Oncol*. 2020 May;146(5):1321-1334. doi: 10.1007/s00432-020-03167-0. | Rui Pedro Marques |
| Martin JM, Handorf EA, Montero AJ, Goldstein LJ. Systemic Therapies Following Progression on First-line CDK4/6-inhibitor Treatment: Analysis of Real-world Data. *Oncologist.* 2022 Jun 8;27(6):441-446. doi: 10.1093/oncolo/oyac075. | James M. Martin |
| Martín-Aguilar AE, Núñez-López H, Ramirez-Sandoval JC. Sorafenib as a second-line treatment in metastatic renal cell carcinoma in Mexico: a prospective cohort study. *BMC Cancer.* 2021 Jan 5;21(1):16. doi: 10.1186/s12885-020-07720-5. | Juan C. Ramirez-Sandoval |
| Martinelli E, Cremolini C, Mazard T, et al. Real-world first-line treatment of patients with BRAF^V600E^-mutant metastatic colorectal cancer: the CAPSTAN CRC study. *ESMO Open.* 2022 Dec;7(6):100603. doi: 10.1016/j.esmoop.2022.100603. | Erika Martinelli |
| Martins D, Rodrigues J, Redondo P, et al. Evaluating the Optimal Sequence of Treatment With EGFR Inhibitors and Bevacizumab in RAS Wild-Type Metastatic Colorectal Cancer. *Cureus.* 2022 Mar 27;14(3):e23543. doi: 10.7759/cureus.23543. | Diana Martins |
| Martins JO, Borges MM, Malta CE, et al. Risk factors for oral mucositis during chemotherapy treatment for solid tumors: a retrospective STROBE-guided study. *Med Oral Patol Oral Cir Bucal.* 2022 Jul 1;27(4):e319-e329. doi: 10.4317/medoral.25253. | Cassia Emanuella Malta |
| Maruta S, Ogasawara S, Ooka Y, et al. Potential of Lenvatinib for an Expanded Indication from the REFLECT Trial in Patients with Advanced Hepatocellular Carcinoma. *Liver Cancer.* 2020 Aug;9(4):382-396. doi: 10.1159/000507022. | Sadahisa Ogasawara |
| Maruzzo M, Pierantoni F, Bortolami A, et al. Real-World Treatment with Nivolumab or Cabozantinib for Metastatic Renal Cell Carcinoma (mRCC) in the Veneto Region of Italy: Results of AMOUR Study. *Target Oncol.* 2022 Jul;17(4):467-474. doi: 10.1007/s11523-022-00892-z. | Marco Maruzzo |
| Masaki C, Sugino K, Tanizawa Y, et al. Multikinase Inhibitor Treatment Patterns for Advanced Thyroid Cancer in Japan: An Administrative Claims Database Study. *Drugs Real World Outcomes.* 2023 Mar;10(1):145-158. doi: 10.1007/s40801-022-00346-y. | Chie Masaki |
| Mason R, Dearden HC, Nguyen B, et al. Combined ipilimumab and nivolumab first-line and after BRAF-targeted therapy in advanced melanoma. *Pigment Cell Melanoma Res*. 2020 Mar;33(2):358-365. doi: 10.1111/pcmr.12831. | Robert Mason |
| Masuishi T, Nagaoka S, Jin L, Yoshizawa K. A post-marketing safety study of ramucirumab with FOLFIRI in patients with metastatic colorectal cancer. *J Gastrointest Oncol*. 2022 Aug;13(4):1701-1710. doi: 10.21037/jgo-21-863. | Kenichi Yoshizawa |
| Mathew A, Joseph S, Boby J, et al. Clinical Benefit of Comprehensive Genomic Profiling for Advanced Cancers in India. *JCO Glob Oncol*. 2022 Mar;8:e2100421. doi: 10.1200/GO.21.00421. | Aju Mathew |
| Matsumiya H, Todo Y, Yamazaki H, et al. Bevacizumab-related gastrointestinal perforation in patients with three or more prior chemotherapy regimens: A real-world experience. *Taiwan J Obstet Gynecol*. 2020 May;59(3):377-380. doi: 10.1016/j.tjog.2020.03.007. | Yukiharu Todo |
| Matsumoto J, Iwata N, Watari S, et al. Adverse Events of Axitinib plus Pembrolizumab Versus Lenvatinib plus Pembrolizumab: A Pharmacovigilance Study in Food and Drug Administration Adverse Event Reporting System. *Eur Urol Focus.* 2023 Jan;9(1):141-144. doi: 10.1016/j.euf.2022.07.003. | Soichiro Ushio |
| Matsumoto K, Tamiya A, Inagaki Y, et al. Efficacy and safety of ramucirumab plus docetaxel in older patients with advanced non-small cell lung cancer: A multicenter retrospective cohort study. *J Geriatr Oncol.* 2022 Mar;13(2):207-213. doi: 10.1016/j.jgo.2021.09.004. | Kinnosuke Matsumoto |
| Matsumoto K, Tamiya A, Matsuda Y, et al. Impact of docetaxel plus ramucirumab on metastatic site in previously treated patients with non-small cell lung cancer: a multicenter retrospective study. *Transl Lung Cancer Res.* 2021 Apr;10(4):1642-1652. doi: 10.21037/tlcr-20-1263. | Kinnosuke Matsumoto |
| Matsumoto MM, Mouli S, Saxena P, et al. Comparing Real World, Personalized, Multidisciplinary Tumor Board Recommendations with BCLC Algorithm: 321-Patient Analysis. *Cardiovasc Intervent Radiol*. 2021 Jul;44(7):1070-1080. doi: 10.1007/s00270-021-02810-8. | Riad Salem |
| Matsuo M, Yasumatsu R, Masuda M, et al. Drug-induced interstitial lung disease in recurrent and/or metastatic head and neck cancer patients treated with cetuximab and/or nivolumab. *Oral Oncol.* 2021 Feb;113:105129. doi: 10.1016/j.oraloncology.2020.105129. | Ryuji Yasumatsu |
| Mauro C, de Jesus VHF, Barros M, et al. Opportunistic and Serious Infections in Patients with Neuroendocrine Tumors Treated with Everolimus: A Multicenter Study of Real-World Patients. *Neuroendocrinology.* 2021;111(7):631-638. doi: 10.1159/000508632. | Rachel P. Riechelmann |
| Mayr L, Steinmaurer T, Weseslindtner L, et al. Viral infections in pediatric brain tumor patients treated with targeted therapies. *Pediatr Blood Cancer*. 2023 Jan;70(1):e30065. doi: 10.1002/pbc.30065. | Andreas Peyrl |
| McAndrew NP, Dickson MA, Clark AS, et al. Early treatment-related neutropenia predicts response to palbociclib. *Br J Cancer.* 2020 Sep;123(6):912-918. doi: 10.1038/s41416-020-0967-7. | Angela DeMichele |
| Medina BD, Choi BH, Rodogiannis KG, et al. Metastasectomy for melanoma is associated with improved overall survival in responders to targeted molecular or immunotherapy. *J Surg Oncol.* 2020 Sep;122(3):555-561. doi: 10.1002/jso.25987. | Ann Y. Lee |
| Meert AP, Toffart AC, Picard M, et al. When targeted therapy for cancer leads to ICU admission. RETRO-TARGETICU multicentric study. *Bull Cancer.* 2022 Sep;109(9):916-924. doi: 10.1016/j.bulcan.2022.04.014. | Virginie Lemiale |
| Mehta A, Saifi M, Batra U, et al. Incidence of *ROS1*-Rearranged Non-Small-Cell Lung Carcinoma in India and Efficacy of Crizotinib in Lung Adenocarcinoma Patients. *Lung Cancer (Auckl).* 2020 Feb 24;11:19-25. doi: 10.2147/LCTT.S244366. | Anurag Mehta |
| Mei J, Li SH, Wang QX, et al. Resection vs. Sorafenib for Hepatocellular Carcinoma With Macroscopic Vascular Invasion: A Real World, Propensity Score Matched Analytic Study. *Front Oncol.* 2020 May 5;10:573. doi: 10.3389/fonc.2020.00573. | Wei Wei |
| Merchant SJ, Kong W, Gyawali B, et al. Effectiveness of Trastuzumab in Routine Clinical Practice: A Population-based Study of Patients with HER-2-positive Oesophageal, Gastroesophageal and Gastric Cancer. *Clin Oncol (R Coll Radiol).* 2021 Mar;33(3):202-207. doi: 10.1016/j.clon.2020.07.013. | S. J. Merchant |
| Merola D, Young J, Schrag D, et al. Effectiveness research in oncology with electronic health record data: A retrospective cohort study emulating the PALOMA-2 trial. *Pharmacoepidemiol Drug Saf.* 2023 Apr;32(4):426-434. doi: 10.1002/pds.5565. | David Merola |
| Merola D, Young J, Schrag D, et al. Oncology Drug Effectiveness from Electronic Health Record Data Calibrated Against RCT Evidence: The PARSIFAL Trial Emulation. *Clin Epidemiol.* 2022 Oct 10;14:1135-1144. doi: 10.2147/CLEP.S373291. | David Merola |
| Meyer N, Pérol D, Duval-Modeste AB, et al. Survival in adult patients with BRAFV600 mutation-positive advanced melanoma: a noninterventional ambispective study of patients with cobimetinib combined with vemurafenib during the French early access program: MELANIS study. *Melanoma Res.* 2022 Aug 1;32(4):269-277. doi: 10.1097/CMR.0000000000000833. | Nicolas Meyer |
| Miao Y, Chen J, Deng R, Liu Y. Clinical Efficacy of Pyrotinib Combined with Capecitabine in the Second-Line or Above Treatment for HER-2 Positive Advanced Breast Cancer and Its Association with Cell-Free DNA. *J Oncol.* 2022 Oct 7;2022:9449489. doi: 10.1155/2022/9449489. | Yufei Liu |
| Min ST, Roohullah A, Tognela A, et al. Patient demographics and management landscape of metastatic colorectal cancer in the third-line setting: Real-world data in an australian population. *Asia Pac J Clin Oncol*. 2022 Apr;18(2):e56-e63. doi: 10.1111/ajco.13553. | Sandy Tun Min |
| Mineur L, François E, Plassot C, et al. PREMIUM: A French prospective multicenter observational study of factors impacting on efficacy and compliance to cetuximab treatment in first-line KRAS wild-type metastatic colorectal cancer. *PLoS One.* 2020 Dec 21;15(12):e0243997. doi: 10.1371/journal.pone.0243997. | Lea Vazquez |
| Minoux K, Lassailly G, Ningarhari M, et al. Neo-Adjuvant Use of Sorafenib for Hepatocellular Carcinoma Awaiting Liver Transplantation. *Transpl Int.* 2022 Nov 9;35:10569. doi: 10.3389/ti.2022.10569. | Sebastien Dharancy |
| Miura S, Jung HA, Lee SY, et al. Sequential Afatinib and Osimertinib in Asian Patients with EGFR Mutation-Positive Non-Small Cell Lung Cancer and Acquired T790M: Combined Analysis of Two Global Non-Interventional Studies. *Onco Targets Ther*. 2022 Aug 22;15:873-882. doi: 10.2147/OTT.S362535. | Sanjay Popat |
| Miyamoto Y, Schirripa M, Suenaga M, et al. A polymorphism in the cachexia-associated gene INHBA predicts efficacy of regorafenib in patients with refractory metastatic colorectal cancer. *PLoS One*. 2020 Sep 24;15(9):e0239439. doi: 10.1371/journal.pone.0239439. | Heinz-Josef Lenz |
| Miyawaki T, Kenmotsu H, Kodama H, et al. Association between oligo-residual disease and patterns of failure during EGFR-TKI treatment in EGFR-mutated non-small cell lung cancer: a retrospective study. *BMC Cancer.* 2021 Nov 19;21(1):1247. doi: 10.1186/s12885-021-08983-2. | Hirotsugu Kenmotsu |
| Mizukami T, Takahashi M, Sunakawa Y, et al. Genomic Landscape of Primary Tumor Site and Clinical Outcome for Patients with Metastatic Colorectal Cancer Receiving Standard-of-Care Chemotherapy. *Target Oncol.* 2022 May;17(3):343-353. doi: 10.1007/s11523-022-00880-3. | Takako Eguchi Nakajima |
| Mizuno T, Horinouchi H, Watanabe S, et al. Number of metastatic organs negatively affects the treatment sequence in patients with EGFR-TKI failure. *Thorac Cancer.* 2020 Apr;11(4):1038-1044. doi: 10.1111/1759-7714.13360. | Hidehito Horinouchi |
| Mizuno T, Sakai T, Tanabe K, et al. Identification of target small molecule tyrosine kinase inhibitors that need monitoring and clinical application of protocol for early detection of cancer therapeutics-related cardiac dysfunction using signal detection: An investigation of real world data. *J Oncol Pharm Pract.* 2021 Jun;27(4):804-814. doi: 10.1177/1078155220930367. | Takahito Mizuno |
| Mo H, Ma F, Li Q, et al. Treatment patterns and clinical outcomes in patients with metastatic breast cancer treated with palbociclib-based therapies: real-world data in the Han population. *Chin Med J (Engl).* 2022 Jul 20;135(14):1734-1741. doi: 10.1097/CM9.0000000000002240. | Fei Ma |
| Mo H, Renna CE, Moore HCF, et al. Real-World Outcomes of Everolimus and Exemestane for the Treatment of Metastatic Hormone Receptor-Positive Breast Cancer in Patients Previously Treated With CDK4/6 Inhibitors. *Clin Breast Cancer.* 2022 Feb;22(2):143-148. doi: 10.1016/j.clbc.2021.10.002. | Hanjie Mo |
| Mohammadi M, Jansen-Werkhoven TM, Ijzerman NS, et al. Dutch Gastrointestinal Stromal Tumor (GIST) Registry Data Comparing Sunitinib with Imatinib Dose Escalation in Second-Line Advanced Non-KIT Exon 9 Mutated GIST Patients. *Target Oncol.* 2022 Nov;17(6):627-634. doi: 10.1007/s11523-022-00926-6. | Mahmoud Mohammadi |
| Moinard-Butot F, Saint-Martin C, Pflumio C, et al. Efficacy of trastuzumab emtansine (T-DM1) and lapatinib after dual HER2 inhibition with trastuzumab and pertuzumab in patient with metastatic breast cancer: Retrospective data from a French multicenter real-life cohort. *Breast*. 2022 Jun;63:54-60. doi: 10.1016/j.breast.2022.03.004. | Fabien Moinard-Butot |
| Moisuc DC, Marinca MV, Gafton B, et al. Antiangiogenic Drug-Induced Proteinuria as a Prognostic Factor in Metastatic Colorectal Cancer. *Curr Oncol.* 2022 May 31;29(6):3996-4011. doi: 10.3390/curroncol29060319. | Petru Cianga |
| Molife C, Cho JM, Lapthorn J, et al. Treatment Patterns, Clinical Outcomes and Health Care Resource Utilisation in Patients with EGFR-mutated Metastatic Non-Small Cell Lung Cancer: A Real-World Study in South Korea. *Drugs Real World Outcomes.* 2023 Mar;10(1):131-143. doi: 10.1007/s40801-022-00344-0. | Hye Ryun Kim |
| Mondragon-Soto M, Rodríguez-Hernández LA, Moreno Jiménez S, et al. Clinical, Therapeutic, and Prognostic Experience in Patients With Glioblastoma. *Cureus.* 2022 Oct 3;14(10):e29856. doi: 10.7759/cureus.29856. | Alberto Gonzalez-Aguilar |
| Monteiro MR, Nunes NCC, Crespo J, et al. Patient-centered Outcomes in Breast Cancer: Description of EQ-5D-5L and EORTC-QLQ-BR23 Measurements in Real-world Data and Their Association With Survival. *Clin Oncol (R Coll Radiol).* 2022 Sep;34(9):608-616. doi: 10.1016/j.clon.2022.05.015. | M. R. Monteiro |
| Moreau-Bachelard C, Letailleur V, Bompas E, et al. Effect of Concomitant Proton Pump Inhibitors with Pazopanib on Cancer Patients: A Retrospective Analysis. *Cancers (Basel).* 2022 Sep 28;14(19):4721. doi: 10.3390/cancers14194721. | Jean-Luc Raoul |
| Moriguchi M, Aramaki T, Sato R, et al. Intrahepatic Tumor Burden as a Novel Factor Influencing the Introduction of Second-line Chemotherapy for Hepatocellular Carcinoma. *Anticancer Res.* 2020 Jul;40(7):3953-3960. doi: 10.21873/anticanres.14387. | Michihisa Moriguchi |
| Morii Y, Fujimoto S, Nakahara R, et al. Effect of proton pump inhibitors on the development of hypomagnesemia induced by panitumumab. *Pharmazie.* 2022 Feb 1;77(2):81-84. doi: 10.1691/ph.2022.1988. | Shozo Nishida |
| Moriwaki T, Fukuoka S, Masuishi T, et al. Prognostic scores for evaluating the survival benefit of regorafenib or trifluridine/tipiracil in patients with metastatic colorectal cancer: an exploratory analysis of the REGOTAS study. *Int J Clin Oncol.* 2020 Apr;25(4):614-621. doi: 10.1007/s10147-019-01600-0. | Toshikazu Moriwaki |
| Morkramer L, Geitner M, Boeger D, et al. Systemic therapy for recurrent and/or metastatic head and neck cancer: a population-based healthcare research study in Thuringia, Germany. *J Cancer Res Clin Oncol.* 2021 Sep;147(9):2625-2635. doi: 10.1007/s00432-021-03535-4. | Orlando Guntinas-Lichius |
| Mosca A, De Giorgi U, Procopio G, et al. An Italian, multicenter, real-world, retrospective study of first-line pazopanib in unselected metastatic renal-cell carcinoma patients: the 'Pamerit' study. *Jpn J Clin Oncol*. 2021 Mar 3;51(3):484-491. doi: 10.1093/jjco/hyaa193. | Alessandra Mosca |
| Moser M, Radu IP, Dufour JF. Effects of Home Care on patients with hepatocellular carcinoma treated with sorafenib. *JGH Open*. 2021 Jul 6;5(8):864-870. doi: 10.1002/jgh3.12533. | Jean-François Dufour |
| Moskovitz M, Dudnik E, Shamai S, et al. ALK Inhibitors or Chemotherapy for Third Line in ALK-positive NSCLC? Real-world Data. *Oncologist.* 2022 Feb 3;27(1):e76-e84. doi: 10.1093/oncolo/oyab005. | Jair Bar |
| Mouabbi JA, Raghavendra AS, Bassett RL Jr, et al. Histology-based survival outcomes in hormone receptor-positive metastatic breast cancer treated with targeted therapies. *NPJ Breast Cancer.* 2022 Dec 20;8(1):131. doi: 10.1038/s41523-022-00499-7. | Jason A. Mouabbi |
| Moya-Alarcón C, Piera G, Callejo Á, Gascó A. Real-world treatment patterns and outcomes in platinum-sensitive recurrent high-grade serous ovarian cancer patients. *J Comp Eff Res.* 2022 Jan;11(1):13-27. doi: 10.2217/cer-2021-0135. | Carlota Moya-Alarcón |
| Müller V, Ruhnke M, Hoffmann O, et al. First-line bevacizumab-containing therapy for HER2-negative locally advanced/metastatic breast cancer: Real-world experience from >2000 patients treated in the multicentre AVANTI study. *Breast*. 2021 Dec;60:70-77. doi: 10.1016/j.breast.2021.08.014. | Volkmar Müller |
| Musicco F, Lasala R, Santoleri F, et al. A multicentre study with real-world data of the use of palbociclib in the treatment of breast cancer: Treatment duration correlates with dose reductions. *J Oncol Pharm Pract.* 2023 Dec;29(8):1806-1815. doi: 10.1177/10781552221117135. | Ruggero Lasala |
| Mycock K, Hanson KA, Taylor-Stokes G, et al. Real-world Treatment Patterns and Clinical Outcomes Associated With Palbociclib Combination Therapy: A Multinational, Pooled Analysis From the Ibrance Real World Insights Study. *Clin Ther.* 2022 Dec;44(12):1588-1601. doi: 10.1016/j.clinthera.2022.11.004. | Katie Mycock |
| Mycock K, Zhan L, Hart K, et al. Real-world treatment of patients with palbociclib for HR+/HER2-advanced/metastatic breast cancer: the Europe IRIS study. *Future Oncol*. 2022 Jan;18(3):349-362. doi: 10.2217/fon-2021-0716. | Katie Mycock |
| Mycock K, Zhan L, Hart K, et al. Real-world treatment patterns and clinical outcomes in patients receiving palbociclib combinations for HR+/HER2- advanced/metastatic breast cancer in Japan: Results from the IRIS study. *Cancer Treat Res Commun*. 2022;32:100573. doi: 10.1016/j.ctarc.2022.100573. | Lin Zhan |
| Mycock K, Zhan L, Taylor-Stokes G, et al. Real-World Palbociclib Use in HR+/HER2- Advanced Breast Cancer in Canada: The IRIS Study. *Curr Oncol*. 2021 Jan 24;28(1):678-688. doi: 10.3390/curroncol28010066. | Katie Mycock |
| Nack E, Koffer PP, Blumberg CS, et al. New Cardiac Abnormalities After Radiotherapy in Breast Cancer Patients Treated With Trastuzumab. *Clin Breast Cancer.* 2020 Jun;20(3):246-252. doi: 10.1016/j.clbc.2019.12.006. | Elana Nack |
| Nadler E, Arondekar B, Aguilar KM, et al. Treatment patterns and clinical outcomes in patients with advanced non-small cell lung cancer initiating first-line treatment in the US community oncology setting: a real-world retrospective observational study. *J Cancer Res Clin Oncol*. 2021 Mar;147(3):671-690. doi: 10.1007/s00432-020-03414-4. | Kathleen Marie Aguilar |
| Nadler E, Espirito JL, Pavilack M, et al. Real-world disease burden and outcomes of brain metastases in EGFR mutation-positive non-small-cell lung cancer. *Future Oncol*. 2020 Aug;16(22):1575-1584. doi: 10.2217/fon-2020-0280. | Eric Nadler |
| Nagai H, Shimada T, Takahashi Y, et al. Evaluation of factors affecting epidermal growth factor receptor tyrosine kinase inhibitor-induced hepatotoxicity in Japanese patients with non-small cell lung cancer: a two-center retrospective study. *J Pharm Health Care Sci.* 2022 Dec 1;8(1):28. doi: 10.1186/s40780-022-00258-7. | Hirofumi Nagai |
| Nahleh ZA, Elimimian EB, Elson LC, et al. Endocrine Therapy Plus Anti-HER2 Therapy as Adjuvant Systemic Therapy for Luminal HER2-Positive Breast Cancer: An Analysis of the National Cancer Database. *Breast Cancer (Auckl).* 2020 Aug 4;14:1178223420945694. doi: 10.1177/1178223420945694. | Zeina A. Nahleh |
| Naito S, Kato T, Numakura K, et al. Prognosis of Japanese metastatic renal cell carcinoma patients in the targeted therapy era. *Int J Clin Oncol*. 2021 Oct;26(10):1947-1954. doi: 10.1007/s10147-021-01979-9. | Sei Naito |
| Nakagawa M, Inoue M, Ogasawara S, et al. Clinical effects and emerging issues of atezolizumab plus bevacizumab in patients with advanced hepatocellular carcinoma from Japanese real-world practice. *Cancer.* 2023 Feb 15;129(4):590-599. doi: 10.1002/cncr.34559. | Sadahisa Ogasawara |
| Nakajima H, Fukuoka S, Masuishi T, et al. Clinical Impact of Primary Tumor Location in Metastatic Colorectal Cancer Patients Under Later-Line Regorafenib or Trifluridine/Tipiracil Treatment. *Front Oncol.* 2021 Jun 15;11:688709. doi: 10.3389/fonc.2021.688709. | Hiromichi Nakajima |
| Nakamoto S, Watanabe J, Ohtani S, et al. Bevacizumab as First-line Treatment for HER2-negative Advanced Breast Cancer: Paclitaxel plus Bevacizumab Versus Other Chemotherapy. *In Vivo.* 2020 May-Jun;34(3):1377-1386. doi: 10.21873/invivo.11917. | Shogo Nakamoto |
| Nakamoto S, Watanabe J, Ohtani S, et al. Shorter duration of first-line chemotherapy reflects poorer outcomes in patients with HER2-negative advanced breast cancer: a multicenter retrospective study. *Sci Rep.* 2021 Nov 2;11(1):21454. doi: 10.1038/s41598-021-00711-x. | Shogo Nakamoto |
| Nakashima M, Takeuchi M, Kawakami K. Effectiveness and Safety of Regorafenib vs. Trifluridine/Tipiracil in Unresectable Colorectal Cancer: A Retrospective Cohort Study. *Clin Colorectal Cancer.* 2020 Dec;19(4):e208-e225. doi: 10.1016/j.clcc.2020.05.003. | Koji Kawakami |
| Nakasya A, Hagiwara Y, Ikoma T, et al. Nanoparticle albumin-bound paclitaxel and ramucirumab versus paclitaxel and ramucirumab as second-line chemotherapy for unresectable advanced or recurrent gastric cancer: a multicenter, propensity score-matched analysis (CROSS SELL study). *Int J Clin* *Oncol*. 2022 Apr;27(4):684-694. doi: 10.1007/s10147-022-02114-y. | Akio Nakasya |
| Nakayama T, Yoshinami T, Yasojima H, et al. Real-world effectiveness of post-trastuzumab emtansine treatment in patients with HER2-positive, unresectable and/or metastatic breast cancer: a retrospective observational study (KBCSG-TR 1917). *BMC Cancer*. 2021 Jul 9;21(1):795. doi: 10.1186/s12885-021-08504-1. | Takahiro Nakayama |
| Nam Y, Kim HC, Kim YC, et al. Clinical impact of rebiopsy among patients with epidermal growth factor receptor-mutant lung adenocarcinoma in a real-world clinical setting. *Thorac Cancer.* 2021 Mar;12(6):890-898. doi: 10.1111/1759-7714.13857. | Chang-Min Choi |
| Nannini M, Rizzo A, Nigro MC, et al. Standard versus personalized schedule of regorafenib in metastatic gastrointestinal stromal tumors: a retrospective, multicenter, real-world study. *ESMO Open*. 2021 Aug;6(4):100222. doi: 10.1016/j.esmoop.2021.100222. | Margherita Nannini |
| Navani V, Wells JC, Boyne DJ, et al. CABOSEQ: The Effectiveness of Cabozantinib in Patients With Treatment Refractory Advanced Renal Cell Carcinoma: Results From the International Metastatic Renal Cell Carcinoma Database Consortium (IMDC). *Clin Genitourin Cancer.* 2023 Feb;21(1):106.e1-106.e8. doi: 10.1016/j.clgc.2022.07.008. | Vishal Navani |
| Nawa H, Niimura T, Yagi K, et al. Evaluation of potential complication of interstitial lung disease with abemaciclib and palbociclib treatments. *Cancer Rep (Hoboken).* 2022 Jan;5(1):e1402. doi: 10.1002/cnr2.1402. | Hideki Nawa |
| Nevala-Plagemann C, Iyengar S, Trunk AD, et al. Treatment Trends and Clinical Outcomes of Left-Sided RAS/RAF Wild-Type Metastatic Colorectal Cancer in the United States. *J Natl Compr Canc Netw.* 2022 Feb 4;20(3):268-275. doi: 10.6004/jnccn.2021.7079. | Ignacio Garrido-Laguna |
| Ng IK, Kumarakulasinghe NB, Syn NL, Soo RA. Development, internal validation and calibration of a risk score to predict survival in patients with *EGFR*-mutant non-small cell lung cancer. *J Clin Pathol.* 2021 Feb;74(2):116-122. doi: 10.1136/jclinpath-2020-206754. | Ross Andrew Soo |
| Ng WW, Lin CC, Cheng CY, et al. Real-world outcomes of first- and second-generation tyrosine kinase inhibitors first-line in patients with epidermal growth factor receptor mutation-positive non-small cell lung cancer: A retrospective observational cohort study. *PLoS One*. 2021 Jun 24;16(6):e0253335. doi: 10.1371/journal.pone.0253335. | Diana Yuwung Yeh |
| Ngo DTM, Williams T, Horder S, et al. Factors Associated with Adverse Cardiovascular Events in Cancer Patients Treated with Bevacizumab. *J Clin Med.* 2020 Aug 18;9(8):2664. doi: 10.3390/jcm9082664. | Aaron L. Sverdlov |
| Nguy S, Wu SP, Oh C, Gerber NK. Outcomes of HER2-positive non-metastatic breast cancer patients treated with anti-HER2 therapy without chemotherapy. *Breast Cancer Res Treat.* 2021 Jun;187(3):815-830. doi: 10.1007/s10549-021-06115-9. | Naamit K. Gerber |
| Nieva J, Reckamp KL, Potter D, et al. Retrospective Analysis of Real-World Management of EGFR-Mutated Advanced NSCLC, After First-Line EGFR-TKI Treatment: US Treatment Patterns, Attrition, and Survival Data. *Drugs Real World Outcomes.* 2022 Sep;9(3):333-345. doi: 10.1007/s40801-022-00302-w. | Jorge Nieva |
| Niizeki T, Tokunaga T, Takami Y, et al. Comparison of Efficacy and Safety of Atezolizumab Plus Bevacizumab and Lenvatinib as First-Line Therapy for Unresectable Hepatocellular Carcinoma: A Propensity Score Matching Analysis. *Target Oncol*. 2022 Nov;17(6):643-653. doi: 10.1007/s11523-022-00921-x. | Shigeo Shimose |
| Nikic P, Babovic N, Dzamic Z, et al. Real World Overall Survival of Patients With Metastatic Renal Cell Carcinoma Treated With Only Available Sunitinib and Pazopanib in First-Line Setting. *Front Oncol.* 2022 Jun 8;12:892156. doi: 10.3389/fonc.2022.892156. | Predrag Nikic |
| Nimgaonkar V, Hubbard RA, Carpenter EL, Mamtani R. Biomarker Testing, Treatment Uptake, and Survival Among Patients With Urothelial Cancer Receiving Gene-Targeted Therapy. *JAMA Oncol.* 2022 Jul 1;8(7):1070-1072. doi: 10.1001/jamaoncol.2022.1167. | Vivek Nimgaonkar, |
| Noteware L, Broadwater G, Dalal N, et al. Brain metastasis as the first and only metastatic relapse site portends worse survival in patients with advanced HER2 + breast cancer. *Breast Cancer Res Treat.* 2023 Jan;197(2):425-434. doi: 10.1007/s10549-022-06799-7. | Sarah Sammons |
| Novakova-Jiresova A, Kopeckova K, Boublikova L, et al. Regorafenib for Metastatic Colorectal Cancer: An Analysis of a Registry-Based Cohort of 555 Patients. *Cancer Manag Res.* 2020 Jul 3;12:5365-5372. doi: 10.2147/CMAR.S255332. | Tomas Buchler |
| Ochi M, Kamoshida T, Araki M, Ikegami T. Prolonged survival in patients with hand-foot skin reaction secondary to cooperative sorafenib treatment. *World J Gastroenterol.* 2021 Aug 28;27(32):5424-5437. doi: 10.3748/wjg.v27.i32.5424. | Masanori Ochi |
| Odan N, Kikawa Y, Matsumoto H, et al. Real-World Outcomes of Treating Advanced Breast Cancer Patients With Palbociclib: A Multicenter Retrospective Cohort Study in Japan-The KBCOG-14 Study. *Breast Cancer (Auckl).* 2020 Dec 28;14:1178223420983843. doi: 10.1177/1178223420983843. | Nina Odan |
| Ogata M, Kotaka M, Ogata T, et al. Regorafenib vs trifluridine/tipiracil for metastatic colorectal cancer refractory to standard chemotherapies: A multicenter retrospective comparison study in Japan. *PLoS One.* 2020 Jun 12;15(6):e0234314. doi: 10.1371/journal.pone.0234314. | Hironaga Satake |
| Ogushi K, Chuma M, Uojima H, et al. Safety and Efﬁcacy of Lenvatinib Treatment in Child-Pugh A and B Patients with Unresectable Hepatocellular Carcinoma in Clinical Practice: A Multicenter Analysis. *Clin Exp Gastroenterol*. 2020 Oct 1;13:385-396. doi: 10.2147/CEG.S256691. | Makoto Chuma |
| Oh CR, Hong JY, Kim JH, et al. Real-World Outcomes of Pazopanib Treatment in Korean Patients with Advanced Soft Tissue Sarcoma: A Multicenter Retrospective Cohort Study. *Target Oncol.* 2020 Aug;15(4):485-493. doi: 10.1007/s11523-020-00731-z. | Jeong Eun Kim |
| Ohe Y, Kato T, Sakai F, et al. Real-world use of osimertinib for epidermal growth factor receptor T790M-positive non-small cell lung cancer in Japan. *Jpn J Clin Oncol.* 2020 Aug 4;50(8):909-919. doi: 10.1093/jjco/hyaa067. | Yuichiro Ohe |
| Ohtsu H, Shimomura A, Miyazaki S, et al. Cardiotoxicity of adjuvant chemotherapy with trastuzumab: a Japanese claim-based data analysis. *Open Heart.* 2022 Aug;9(2):e002053. doi: 10.1136/openhrt-2022-002053. | Kazuhiro Sase |
| Ohyama K, Hirakawa K, Sasazaki K, et al. Time-to-onset of diabetes with everolimus use: analysis of a spontaneous reporting system database. *Pharmazie*. 2021 Oct 1;76(10):515-518. doi: 10.1691/ph.2021.1624. | Katsuhiro Ohyama |
| Ohyama K, Tanaka H, Hori Y. Effect of Concomitant Drug Use on the Onset and Exacerbation of Diabetes Mellitus in Everolimus-Treated Cancer. *J Pharm Pharm Sci.* 2022;25:245-252. doi: 10.18433/jpps32908. | Katsuhiro Ohyama |
| Okamoto M, Tajiri W, Ueo H, et al. Efficacy of Adjuvant Combination Therapy With Trastuzumab and Chemotherapy in HER2-positive Early Breast Cancer: A Single Institutional Cohort Study from Clinical Practice. *Anticancer Res.* 2020 Jun;40(6):3315-3323. doi: 10.21873/anticanres.14314. | Eriko Tokunaga |
| Okauchi S, Numata T, Nawa T, et al. Real Clinical Practice in ALK-rearranged NSCLC Patients: A Retrospective Observational Study. *Anticancer Res.* 2020 Feb;40(2):957-964. doi: 10.21873/anticanres.14029. | Hiroaki Satoh |
| Oki E, Kawahira M, Kusumoto T, et al. Multicenter Cohort Study to Assess the Association between Changes on Imaging and Outcome after Regorafenib Treatment (KSCC1603). *Oncology*. 2020;98(10):719-726. doi: 10.1159/000507814. | Eiji Oki |
| Okunaka M, Kotani D, Demachi K, et al. Retrospective cohort study of nanoparticle albumin-bound paclitaxel plus ramucirumab versus paclitaxel plus ramucirumab as second-line treatment in patients with advanced gastric cancer. *BMC Cancer*. 2020 Nov 16;20(1):1111. doi: 10.1186/s12885-020-07614-6. | Daisuke Kotani |
| Okuyama H, Kagawa Y, Masuishi T, et al. Infusion-related reaction to ramucirumab plus FOLFIRI in patients with advanced colorectal cancer. *Int J Clin Oncol*. 2021 Nov;26(11):2025-2028. doi: 10.1007/s10147-021-02004-9. | Akihito Tsuji |
| Olsen S, Liao J, Hayashi H. Real-World Clinical Outcomes after Genomic Profiling of Circulating Tumor DNA in Patients with Previously Treated Advanced Non-Small Cell Lung Cancer. *Curr Oncol.* 2022 Jul 8;29(7):4811-4826. doi: 10.3390/curroncol29070382. | Steven Olsen |
| Omar NE, Fahmy Soliman AI, Eshra M, et al. Postmarketing safety of anaplastic lymphoma kinase (ALK) inhibitors: an analysis of the FDA Adverse Event Reporting System (FAERS). *ESMO Open*. 2021 Dec;6(6):100315. doi: 10.1016/j.esmoop.2021.100315. | Nabil E. Omar |
| Ono T, Igawa S, Kurahayashi S, et al. Impact of neutrophil-to-lymphocyte ratio in patients with EGFR-mutant NSCLC treated with tyrosine kinase inhibitors. *Invest New Drugs*. 2020 Jun;38(3):885-893. doi: 10.1007/s10637-020-00919-0. | Satoshi Igawa |
| Oppelt KA, Kuiper JG, Ingrasciotta Y, et al. Characteristics and Absolute Survival of Metastatic Colorectal Cancer Patients Treated With Biologics: A Real-World Data Analysis From Three European Countries. *Front Oncol.* 2021 Mar 5;11:630456. doi: 10.3389/fonc.2021.630456. | Katja A. Oppelt |
| Oranratnachai S, Rattanasiri S, Sirachainan E, et al. Treatment outcomes of advanced hepatocellular carcinoma in real-life practice: Chemotherapy versus multikinase inhibitors. *Cancer Med.* 2023 Feb;12(3):3046-3053. doi: 10.1002/cam4.5224. | Sasivimol Rattanasiri |
| Osawa T, Kojima T, Hara T, et al. Oncological outcomes of a multicenter cohort treated with axitinib for metastatic renal cell carcinoma. *Cancer Sci.* 2020 Jul;111(7):2460-2471. doi: 10.1111/cas.14449. | Nobuo Shinohara |
| O'Shaughnessy J, Robert N, Annavarapu S, et al. Recurrence rates in patients with HER2+ breast cancer who achieved a pathological complete response after neoadjuvant pertuzumab plus trastuzumab followed by adjuvant trastuzumab: a real-world evidence study. *Breast Cancer Res Treat*. 2021 Jun;187(3):903-913. doi: 10.1007/s10549-021-06137-3. | Srinivas Annavarapu |
| O'Sullivan DE, Jarada TN, Yusuf A, et al. Prevalence, Treatment Patterns, and Outcomes of Individuals with *EGFR* Positive Metastatic Non-Small Cell Lung Cancer in a Canadian Real-World Setting: A Comparison of Exon 19 Deletion, L858R, and Exon 20 Insertion *EGFR* Mutation Carriers. *Curr Oncol.* 2022 Sep 30;29(10):7198-7208. doi: 10.3390/curroncol29100567. | Devon J. Boyne |
| Osumi H, Shinozaki E, Ooki A, et al. Early hypertension and neutropenia are predictors of treatment efficacy in metastatic colorectal cancer patients administered FOLFIRI and vascular endothelial growth factor inhibitors as second-line chemotherapy. *Cancer Med*. 2021 Jan;10(2):615-625. doi: 10.1002/cam4.3638. | Eiji Shinozaki |
| Ota Y, Kodaira T, Fujii H, et al. Real-world clinical outcomes in Japanese patients with locally advanced squamous cell carcinoma of the head and neck treated with radiotherapy plus cetuximab: a prospective observational study (JROSG12-2). *Int J Clin Oncol.* 2022 Nov;27(11):1675-1683. doi: 10.1007/s10147-022-02228-3. | Yosuke Ota |
| Ouyang T, Cao Y, Chen L, Zheng C. Comparison of the Efficacy Among Transcatheter Arterial Chemoembolization (TACE)-Radiofrequency Ablation Plus Apatinib, TACE Plus Apatinib, and TACE Alone for Hepatocellular Carcinoma: A Retrospective Study. *Cardiovasc Intervent Radiol*. 2022 Jun;45(6):780-790. doi: 10.1007/s00270-022-03141-y. | Chuansheng Zheng |
| Ouyang W, Yu J, Zhou Y, et al. Risk factors of metachronous brain metastasis in patients with EGFR-mutated advanced non-small cell lung cancer. *BMC Cancer.* 2020 Jul 28;20(1):699. doi: 10.1186/s12885-020-07202-8. | Conghua Xie |
| Oya M, Kaneko S, Imai T, et al. Effectiveness and safety of sorafenib for renal cell, hepatocellular and thyroid carcinoma: pooled analysis in patients with renal impairment. *Cancer Chemother Pharmacol.* 2022 Jun;89(6):761-772. doi: 10.1007/s00280-022-04428-0. | Yutaka Okayama |
| Ozeki N, Kadomatsu Y, Mizuno Y, et al. Risk Assessment for Loss-of-Exercise Capacity After Lung Cancer Surgery: Current Advances in Surgery and Systemic Treatment. *World J Surg*. 2022 Apr;46(4):933-941. doi: 10.1007/s00268-021-06427-3. | Naoki Ozeki |
| Palmieri LJ, Mineur L, Tougeron D, et al. Withholding the Introduction of Anti-Epidermal Growth Factor Receptor: Impact on Outcomes in RAS Wild-Type Metastatic Colorectal Tumors: A Multicenter AGEO Study (the WAIT or ACT Study). *Oncologist.* 2020 Feb;25(2):e266-e275. doi: 10.1634/theoncologist.2019-0328. | Romain Coriat |
| Palumbo R, Torrisi R, Sottotetti F, et al. Patterns of treatment and outcome of palbociclib plus endocrine therapy in hormone receptor-positive/HER2 receptor-negative metastatic breast cancer: a real-world multicentre Italian study. *Ther Adv Med Oncol.* 2021 Mar 10;13:1758835920987651. doi: 10.1177/1758835920987651. | Erica Quaquarini |
| Pan D, Rong X, Chen D, et al. Mortality of early treatment for radiation-induced brain necrosis in head and neck cancer survivors: A multicentre, retrospective, registry-based cohort study. *EClinicalMedicine.* 2022 Aug 12;52:101618. doi: 10.1016/j.eclinm.2022.101618. | Yamei Tang |
| Pang LL, Gan JD, Tan JR, et al. Efficacy and potential resistance mechanisms of afatinib in advanced non-small cell lung cancer patients with EGFR G719X/L861Q/S768I. *Cancer*. 2022 Nov 1;128(21):3804-3814. doi: 10.1002/cncr.34451. | Wen-Feng Fang |
| Parisi A, Cortellini A, Cannita K, et al. Evaluation of Second-line Anti-VEGF after First-line Anti-EGFR Based Therapy in RAS Wild-Type Metastatic Colorectal Cancer: The Multicenter "SLAVE" Study. *Cancers (Basel).* 2020 May 16;12(5):1259. doi: 10.3390/cancers12051259. | Alessandro Parisi |
| Parisi A, Cortellini A, Venditti O, et al. Post-Induction Management in Patients With Left-Sided *RAS* and *BRAF* Wild-Type Metastatic Colorectal Cancer Treated With First-Line Anti-EGFR-Based Doublet Regimens: A Multicentre Study. *Front Oncol.* 2021 Oct 27;11:712053. doi: 10.3389/fonc.2021.712053. | Alessandro Parisi |
| Parisi A, Porzio G, Cannita K, et al. Clinicians' Attitude to Doublet Plus Anti-EGFR Versus Triplet Plus Bevacizumab as First-line Treatment in Left-Sided RAS and BRAF Wild-Type Metastatic Colorectal Cancer Patients: A Multicenter, "Real-Life", Case-Control Study. *Clin Colorectal Cancer*. 2021 Dec;20(4):318-325. doi: 10.1016/j.clcc.2021.07.003. | Alessandro Parisi |
| Park J, Jung HA, Shim JH, et al. Multimodal treatments and outcomes for anaplastic thyroid cancer before and after tyrosine kinase inhibitor therapy: a real-world experience. *Eur J Endocrinol.* 2021 May 6;184(6):837-845. doi: 10.1530/EJE-20-1482. | Jae Hoon Chung |
| Park J, Kim SI, Jeong SY, et al. Second-line olaparib maintenance therapy is associated with poor response to subsequent chemotherapy in BRCA1/2-mutated epithelial ovarian cancer: A multicentre retrospective study. *Gynecol Oncol.* 2022 Apr;165(1):97-104. doi: 10.1016/j.ygyno.2022.02.002. | Jung-Yun Lee |
| Park JC, Durbeck J, Clark JR, Faden DL. Treatment sequence of cetuximab and immune checkpoint inhibitor in head and neck squamous cell carcinoma differentially affects outcomes. *Oral Oncol.* 2020 Dec;111:105024. doi: 10.1016/j.oraloncology.2020.105024. | Daniel L. Faden |
| Park JH, Yeo JH, Kim YS, et al. Efficacy and Safety of Trastuzumab Biosimilar (CT-P6) Compared With Reference Trastuzumab in Patients With HER2-positive Advanced Gastric Cancer: A Retrospective Analysis. *Am J Clin Oncol*. 2022 Feb 1;45(2):61-65. doi: 10.1097/COC.0000000000000887. | Sun Jin Sym |
| Park JH, Yeo JH, Kim YS, et al. Predictive Roles of HER2 Gene Amplification and Neutrophil-to-Lymphocyte Ratio on Survival in HER2-Positive Advanced Gastric Cancer Treated With Trastuzumab-Based Chemotherapy. *Am J Clin Oncol.* 2021 Jun 1;44(6):232-238. doi: 10.1097/COC.0000000000000810. | Sun Jin Sym |
| Park S, Lee SY, Kim D, et al. Comparison of epidermal growth factor receptor tyrosine kinase inhibitors for patients with lung adenocarcinoma harboring different epidermal growth factor receptor mutation types. *BMC Cancer*. 2021 Jan 11;21(1):52. doi: 10.1186/s12885-020-07765-6. | Jung Hyun Chang |
| Park SY, Suh KJ, Lee DW, et al. Prognostic role of tumor subtype and germline BRCA mutation in advanced breast cancer patients treated with palbociclib plus endocrine therapy. *Breast Cancer Res Treat.* 2022 Nov;196(1):121-128. doi: 10.1007/s10549-022-06566-8. | Seock-Ah Im |
| Patel A, Batra U, Prasad KT, et al. Real world experience of treatment and outcome in ALK-rearranged metastatic nonsmall cell lung cancer: A multicenter study from India. *Curr Probl Cancer.* 2020 Jun;44(3):100571. doi: 10.1016/j.currproblcancer.2020.100571. | Bivas Biswas |
| Patel AK, Abhyankar R, Brais LK, et al. Trifluridine/Tipiracil and Regorafenib in Patients with Metastatic Colorectal Cancer: A Retrospective Study at a Tertiary Oncology Center. *Oncologist.* 2021 Dec;26(12):e2161-e2169. doi: 10.1002/onco.13942. | Lynn Huynh |
| Patt D, Liu X, Li B, et al. Real-World Treatment Patterns and Outcomes of Palbociclib Plus an Aromatase Inhibitor for Metastatic Breast Cancer: Flatiron Database Analysis. *Clin Breast Cancer.* 2022 Aug;22(6):601-610. doi: 10.1016/j.clbc.2022.05.002. | Debra Patt |
| Patwala K, Prince DS, Celermajer Y, et al. Lenvatinib for the treatment of hepatocellular carcinoma-a real-world multicenter Australian cohort study. *Hepatol Int*. 2022 Oct;16(5):1170-1178. doi: 10.1007/s12072-022-10398-5. | Kurvi Patwala |
| Pavlick AC, Zhao R, Lee CH, et al. First-line immunotherapy versus targeted therapy in patients with *BRAF*-mutant advanced melanoma: a real-world analysis. *Future Oncol.* 2021 Feb;17(6):689-699. doi: 10.2217/fon-2020-0643. | Anna C. Pavlick |
| Pawloy K, Urquhart G, Brown D, et al. Not all small HER2 positive breast cancers have the same clinical outcome in the North-East of Scotland. *Cancer Treat Res Commun*. 2022;31:100549. doi: 10.1016/j.ctarc.2022.100549. | Karola Pawloy |
| Payen T, Trédaniel J, Moreau L, et al. Real world data of efficacy and safety of erlotinib as first-line TKI treatment in EGFR mutation-positive advanced non-small cell lung cancer: Results from the EGFR-2013-CPHG study. *Respir Med Res.* 2021 Nov;80:100795. doi: 10.1016/j.resmer.2020.100795. | D. Debieuvre |
| Pedersen S, Larsen KO, Christensen AH, et al. Cardiotoxicity in metastatic melanoma patients treated with BRAF and MEK inhibitors in a real-world setting. *Acta Oncol.* 2022 Jan;61(1):45-51. doi: 10.1080/0284186X.2021.1992010. | Eva Ellebaek |
| Peled N, Gillis R, Kilickap S, et al. GLASS: Global Lorlatinib for ALK(+) and ROS1(+) retrospective Study: real world data of 123 NSCLC patients. *Lung Cancer*. 2020 Oct;148:48-54. doi: 10.1016/j.lungcan.2020.07.022. | Nir Peled |
| Peng D, Shan D, Dai C, et al. Real-World Data on Osimertinib in Chinese Patients with Pretreated, EGFR T790M Mutation Positive, Advanced Non-Small Cell Lung Cancer: A Retrospective Study. *Cancer Manag Res.* 2021 Feb 26;13:2033-2039. doi: 10.2147/CMAR.S287466. | Xuezhen Ma |
| Peng TR, Wu TW, Wu CC, et al. Transarterial chemoembolization with or without sorafenib for hepatocellular carcinoma: A real-world propensity score-matched study. *Tzu Chi Med J.* 2021 Sep 10;34(2):219-225. doi: 10.4103/tcmj.tcmj_84_21. | Ching-Sheng Hsu |
| Peng W, Zhang F, Wang Z, et al. Large Scale, Multicenter, Prospective Study of Apatinib in Advanced Gastric Cancer: A Real-World Study from China. *Cancer Manag Res*. 2020 Aug 6;12:6977-6985. doi: 10.2147/CMAR.S249153. | Guoping Sun |
| Perdrizet K, Sutradhar R, Li Q, et al. Second and later-line erlotinib use in non-small cell lung cancer: real world outcomes and practice patterns overtime in Canada. *J Thorac Dis.* 2021 Sep;13(9):5419-5429. doi: 10.21037/jtd-21-804. | Kirstin Perdrizet |
| Persano M, Rimini M, Tada T, et al. Clinical outcomes with atezolizumab plus bevacizumab or lenvatinib in patients with hepatocellular carcinoma: a multicenter real-world study. *J Cancer Res Clin Oncol.* 2023 Aug;149(9):5591-5602. doi: 10.1007/s00432-022-04512-1. | Margherita Rimini |
| Petito LC, García-Albéniz X, Logan RW, et al. Estimates of Overall Survival in Patients With Cancer Receiving Different Treatment Regimens: Emulating Hypothetical Target Trials in the Surveillance, Epidemiology, and End Results (SEER)-Medicare Linked Database. *JAMA Netw Open*. 2020 Mar 2;3(3):e200452. doi: 10.1001/jamanetworkopen.2020.0452. | Lucia C. Petito |
| Petracci F, Abuin GG, Pini A, Chacón M. RENATA study-Latin American prospective experience: clinical outcome of patients treated with palbociclib in hormone receptor-positive metastatic breast cancer-real-world use. *Ecancermedicalscience*. 2020 Jun 17;14:1058. doi: 10.3332/ecancer.2020.1058. | Fernando Petracci |
| Pinto Á, Reig O, Iglesias C, et al. Clinical Factors Associated With Long-Term Benefit in Patients With Metastatic Renal Cell Carcinoma Treated With Axitinib: Real-World AXILONG Study. *Clin Genitourin Cancer*. 2022 Feb;20(1):25-34. doi: 10.1016/j.clgc.2021.09.006. | Álvaro Pinto |
| Pishvaian MJ, Blais EM, Brody JR, et al. Overall survival in patients with pancreatic cancer receiving matched therapies following molecular profiling: a retrospective analysis of the Know Your Tumor registry trial. *Lancet Oncol*. 2020 Apr;21(4):508-518. doi: 10.1016/S1470-2045(20)30074-7. | Michael J. Pishvaian |
| Pizzuti L, Krasniqi E, Barchiesi G, et al. Distinct HR expression patterns significantly affect the clinical behavior of metastatic HER2+ breast cancer and degree of benefit from novel anti-HER2 agents in the real world setting. *Int J Cancer*. 2020 Apr 1;146(7):1917-1929. doi: 10.1002/ijc.32583. | Eriseld Krasniqi |
| Pizzuti L, Krasniqi E, Sperduti I, et al. PANHER study: a 20-year treatment outcome analysis from a multicentre observational study of HER2-positive advanced breast cancer patients from the real-world setting. *Ther Adv Med Oncol*. 2021 Nov 29;13:17588359211059873. doi: 10.1177/17588359211059873. | Maddalena Barba |
| Pluzanski A, Krzakowski M, Kowalski D, Dziadziuszko R. Real-world clinical outcomes of first-generation and second-generation epidermal growth factor receptor tyrosine kinase inhibitors in a large cohort of European non-small-cell lung cancer patients. *ESMO Open.* 2020 Nov;5(6):e001011. doi: 10.1136/esmoopen-2020-001011. | Adam Pluzanski |
| Poizeau F, Kerbrat S, Happe A, et al. Patients with Metastatic Melanoma Receiving Anticancer Drugs: Changes in Overall Survival, 2010-2017. *J Invest Dermatol*. 2021 Apr;141(4):830-839.e3. doi: 10.1016/j.jid.2020.07.038. | Alain Dupuy |
| Polychronopoulou E, Giordano SH, Chou LN, et al. Signal Detection of Adverse Events Associated with Trastuzumab in a Cohort of Elderly Patients with Breast Cancer. *Oncologist.* 2022 Jun 8;27(6):434-440. doi: 10.1093/oncolo/oyac059. | Yong-Fang Kuo |
| Pontes F, Garcia AR, Domingues I, et al. Survival predictors and outcomes of patients with recurrent and/or metastatic head and neck cancer treated with chemotherapy plus cetuximab as first-line therapy: A real-world retrospective study. *Cancer Treat Res Commun*. 2021;27:100375. doi: 10.1016/j.ctarc.2021.100375. | Filipa Pontes |
| Poorvu PD, Hu J, Zheng Y, et al. Treatment-related amenorrhea in a modern, prospective cohort study of young women with breast cancer. *NPJ Breast Cancer.* 2021 Jul 27;7(1):99. doi: 10.1038/s41523-021-00307-8. | Ann H. Partridge |
| Popat S, Hsia TC, Hung JY, et al. Tyrosine Kinase Inhibitor Activity in Patients with NSCLC Harboring Uncommon EGFR Mutations: A Retrospective International Cohort Study (UpSwinG). *Oncologist.* 2022 Apr 5;27(4):255-265. doi: 10.1093/oncolo/oyac022. | Sanjay Popat |
| Popat S, Jung HA, Lee SY, et al. Sequential afatinib and osimertinib in patients with EGFR mutation-positive NSCLC and acquired T790M: A global non-interventional study (UpSwinG). *Lung Cancer*. 2021 Dec;162:9-15. doi: 10.1016/j.lungcan.2021.09.009. | Sanjay Popat |
| Poprach A, Holanek M, Chloupkova R, et al. Cytoreductive Nephrectomy and Overall Survival of Patients with Metastatic Renal Cell Carcinoma Treated with Targeted Therapy-Data from the National Renis Registry. *Cancers (Basel).* 2020 Oct 10;12(10):2911. doi: 10.3390/cancers12102911. | Alexandr Poprach |
| Porte B, Carton M, Lerebours F, et al. Real life efficacy of palbociclib and endocrine therapy in HR positive, HER2 negative advanced breast cancer. *Breast.* 2020 Dec;54:303-310. doi: 10.1016/j.breast.2020.11.008. | P. Cottu |
| Price GL, Sudharshan L, Ryan P, et al. Real world incidence and management of adverse events in patients with HR+, HER2- metastatic breast cancer receiving CDK4 and 6 inhibitors in a United States community setting. *Curr Med Res Opin.* 2022 Aug;38(8):1319-1331. doi: 10.1080/03007995.2022.2073122. | Yu-Jing Huang |
| Provencio M, Serna-Blasco R, Franco F, et al. Analysis of circulating tumour DNA to identify patients with epidermal growth factor receptor-positive non-small cell lung cancer who might benefit from sequential tyrosine kinase inhibitor treatment. *Eur J Cancer*. 2021 May;149:61-72. doi: 10.1016/j.ejca.2021.02.031. | Atocha Romero |
| Provencio M, Terrasa J, Garrido P, et al. Osimertinib in advanced EGFR-T790M mutation-positive non-small cell lung cancer patients treated within the Special Use Medication Program in Spain: OSIREX-Spanish Lung Cancer Group. *BMC Cancer*. 2021 Mar 6;21(1):230. doi: 10.1186/s12885-021-07922-5. | Virginia Calvo |
| Ptashnikov D, Zaborovskii N, Kostrickii S, et al. Metastasectomy and Targeted Therapy for Patients With Spinal Metastases of Renal Cell Carcinoma. *Int J Spine Surg.* 2020 Dec;14(6):982-988. doi: 10.14444/7147. | Nikita Zaborovskii |
| Pusceddu S, Prinzi N, Tafuto S, et al. Association of Upfront Peptide Receptor Radionuclide Therapy With Progression-Free Survival Among Patients With Enteropancreatic Neuroendocrine Tumors. *JAMA Netw Open.* 2022 Feb 1;5(2):e220290. doi: 10.1001/jamanetworkopen.2022.0290. | Claudio Ricci |
| Qian J, Zhang X, Zhang B, et al. Tyrosine Kinase Inhibitor-Related Hepatotoxicity in Patients with Advanced Lung Adenocarcinoma: A Real-World Retrospective Study. *Cancer Manag Res.* 2020 May 11;12:3293-3299. doi: 10.2147/CMAR.S237968. | Baohui Han |
| Qin S, Ji J, Xu RH, et al. Treatment Patterns and Outcomes in Chinese Patients with Gastric Cancer by HER2 Status: A Noninterventional Registry Study (EVIDENCE). *Oncologist.* 2021 Sep;26(9):e1567-e1580. doi: 10.1002/onco.13826. | Lin Shen |
| Qiu X, Li M, Wu L, et al. Severe Fatigue is an Important Factor in the Prognosis of Patients with Advanced Hepatocellular Carcinoma Treated with Sorafenib. *Cancer Manag Res.* 2020 Sep 4;12:7983-7992. doi: 10.2147/CMAR.S233448. | Liqun Wu |
| Qiu Z, Shen L, Jiang Y, et al. Transarterial chemoembolization (TACE) combined with apatinib versus TACE combined with sorafenib in advanced hepatocellular carcinoma patients: a multicenter retrospective study. *Ann Transl Med.* 2021 Feb;9(4):283. doi: 10.21037/atm-20-5360. | Yunfei Yuan |
| Qu Y, Lin Z, Qi Y, et al. PAK1 expression determines poor prognosis and immune evasion in metastatic renal cell carcinoma patients. *Urol Oncol.* 2020 Apr;38(4):293-304. doi: 10.1016/j.urolonc.2019.10.010. | Jiejie Xu |
| Qureshi S, Boily G, Boulanger J, et al. Advanced Lung Cancer Patients' Use of EGFR Tyrosine Kinase Inhibitors and Overall Survival: Real-World Evidence from Quebec, Canada. *Curr Oncol.* 2022 Oct 26;29(11):8043-8073. doi: 10.3390/curroncol29110636. | Samia Qureshi |
| Rahardja S, Tan RYC, Sultana R, et al. Efficacy, patterns of use and cost of Pertuzumab in the treatment of HER2+ metastatic breast cancer in Singapore: The National Cancer Centre Singapore experience. *World J Clin Oncol.* 2020 Mar 24;11(3):143-151. doi: 10.5306/wjco.v11.i3.143. | Sylwan Rahardja |
| Rakha EA, Miligy IM, Quinn CM, et al. Retrospective observational study of HER2 immunohistochemistry in borderline breast cancer patients undergoing neoadjuvant therapy, with an emphasis on Group 2 (HER2/CEP17 ratio ≥2.0, HER2 copy number <4.0 signals/cell) cases. *Br J Cancer.* 2021 May;124(11):1836-1842. doi: 10.1038/s41416-021-01351-8. | Emad A. Rakha |
| Rala de Paula BH, Costa METF, de Sousa CAM, Bines J. Is there a window of opportunity to optimize trastuzumab cardiac monitoring? *World J Cardiol.* 2022 Jul 26;14(7):403-410. doi: 10.4330/wjc.v14.i7.403. | Bruno Henrique Rala de Paula |
| Ramagopalan S, Leahy TP, Ray J, et al. The value of innovation: association between improvements in survival of advanced and metastatic non-small cell lung cancer and targeted and immunotherapy. *BMC Med.* 2021 Sep 15;19(1):209. doi: 10.1186/s12916-021-02070-w. | Sreeram Ramagopalan |
| Ramagopalan SV, Pisoni R, Rathore LS, et al. Association of Pertuzumab, Trastuzumab, and Docetaxel Combination Therapy With Overall Survival in Patients With Metastatic Breast Cancer. *JAMA Netw Open*. 2021 Jan 4;4(1):e2027764. doi: 10.1001/jamanetworkopen.2020.27764. | Sreeram V. Ramagopalan |
| Ramagopalan SV, Pisoni R, Zenin A, et al. Comparative effectiveness of trastuzumab emtansine versus lapatinib plus chemotherapy for HER2+ metastatic breast cancer. *J Comp Eff Res.* 2021 May;10(7):595-602. doi: 10.2217/cer-2020-0201. | Sreeram V. Ramagopalan |
| Raphael A, Dudnik E, Hershkovitz D, et al. *FGFR* Fusions as an Acquired Resistance Mechanism Following Treatment with Epidermal Growth Factor Receptor Tyrosine Kinase Inhibitors (EGFR TKIs) and a Suggested Novel Target in Advanced Non-Small Cell Lung Cancer (aNSCLC). *J Clin Med.* 2022 Apr 28;11(9):2475. doi: 10.3390/jcm11092475. | Elizabeth Dudnik |
| Rapposelli IG, Tada T, Shimose S, et al. Adverse events as potential predictive factors of activity in patients with advanced hepatocellular carcinoma treated with lenvatinib. *Liver Int.* 2021 Dec;41(12):2997-3008. doi: 10.1111/liv.15014. | Andrea Casadei-Gardini |
| Raschi E, Fusaroli M, Ardizzoni A, et al. Cyclin-dependent kinase 4/6 inhibitors and interstitial lung disease in the FDA adverse event reporting system: a pharmacovigilance assessment. *Breast Cancer Res Treat*. 2021 Feb;186(1):219-227. doi: 10.1007/s10549-020-06001-w. | Emanuel Raschi |
| Rath S, Elamarthi P, Parab P, et al. Efficacy and safety of palbociclib and ribociclib in patients with estrogen and/or progesterone receptor positive, HER2 receptor negative metastatic breast cancer in routine clinical practice. *PLoS One.* 2021 Jul 22;16(7):e0253722. doi: 10.1371/journal.pone.0253722. | Sudeep Gupta |
| Rebuzzi SE, Cerbone L, Signori A, et al. Application of the Meet-URO score to metastatic renal cell carcinoma patients treated with second- and third-line cabozantinib. *Ther Adv Med Oncol.* 2022 Feb 26;14:17588359221079580. doi: 10.1177/17588359221079580. | Sara Elena Rebuzzi |
| Reinders MTM, van Meer S, Burgmans MC, et al. Trends in incidence, diagnosis, treatment and survival of hepatocellular carcinoma in a low-incidence country: Data from the Netherlands in the period 2009-2016. *Eur J Cancer.* 2020 Sep;137:214-223. doi: 10.1016/j.ejca.2020.07.008. | Margot T. M. Reinders |
| Ribeiro ARG, Salvadori MM, de Brot L, et al. Retrospective analysis of the role of cyclin E1 overexpression as a predictive marker for the efficacy of bevacizumab in platinum-sensitive recurrent ovarian cancer. *Ecancermedicalscience.* 2021 Jul 5;15:1262. doi: 10.3332/ecancer.2021.1262. | Alexandre André Balieiro Anastácio da Costa |
| Rigakos G, Razis E, Koliou GA, et al. Evaluation of the Role of p95 HER2 Isoform in Trastuzumab Efficacy in Metastatic Breast Cancer. *Anticancer Res.* 2021 Apr;41(4):1793-1802. doi: 10.21873/anticanres.14945. | Georgios Rigakos |
| Rigo R, Doherty J, Koczka K, et al. Real World Outcomes in Patients with Advanced Melanoma Treated in Alberta, Canada: A Time-Era Based Analysis. *Curr Oncol.* 2021 Oct 5;28(5):3978-3986. doi: 10.3390/curroncol28050338. | Jose G. Monzon |
| Rimini M, Kudo M, Tada T, et al. Nonalcoholic steatohepatitis in hepatocarcinoma: new insights about its prognostic role in patients treated with lenvatinib. *ESMO Open*. 2021 Dec;6(6):100330. doi: 10.1016/j.esmoop.2021.100330. | Andrea Casadei-Gardini |
| Rimini M, Rimassa L, Ueshima K, et al. Atezolizumab plus bevacizumab versus lenvatinib or sorafenib in non-viral unresectable hepatocellular carcinoma: an international propensity score matching analysis. *ESMO Open*. 2022 Dec;7(6):100591. doi: 10.1016/j.esmoop.2022.100591. | Andrea Casadei-Gardin |
| Rimini M, Shimose S, Lonardi S, et al. Lenvatinib versus Sorafenib as first-line treatment in hepatocellular carcinoma: A multi-institutional matched case-control study. *Hepatol Res.* 2021 Dec;51(12):1229-1241. doi: 10.1111/hepr.13718. | Margherita Rimini |
| Rimini M, Yoo C, Lonardi S, et al. Identification of Regorafenib Prognostic Index (REP Index) via Recursive Partitioning Analysis in Patients with Advanced Hepatocellular Carcinoma Receiving Systemic Treatment: A Real-World Multi-Institutional Experience. *Target Oncol.* 2021 Sep;16(5):653-661. doi: 10.1007/s11523-021-00834-1. | Margherita Rimini |
| Ring A, Battisti NML, Reed MWR, et al. Bridging The Age Gap: observational cohort study of effects of chemotherapy and trastuzumab on recurrence, survival and quality of life in older women with early breast cancer. *Br J Cancer*. 2021 Jul;125(2):209-219. doi: 10.1038/s41416-021-01388-9. | Lynda Wyld |
| Roger S, Edeline J, Campillo-Gimenez B, et al. Adverse events of targeted therapies reported by patients with cancer treated in primary care. *Eur J Gen Pract*. 2020 Dec;26(1):202-209. doi: 10.1080/13814788.2020.1846713. | Samuel Roger |
| Roman D, Saftescu S, Timar B, et al. Diabetes Mellitus and Other Predictors for the Successful Treatment of Metastatic Colorectal Cancer: A Retrospective Study. *Medicina (Kaunas).* 2022 Jun 29;58(7):872. doi: 10.3390/medicina58070872. | Sorin Saftescu |
| Romeo M, Gil-Martín M, Gaba L, et al. Multicenter Real-World Data of Subsequent Chemotherapy after Progression to PARP Inhibitors in a Maintenance Relapse Setting. *Cancers (Basel).* 2022 Sep 11;14(18):4414. doi: 10.3390/cancers14184414. | Margarita Romeo |
| Rosati G, Corsi D, Avallone A, et al. Reduced-dose of doublet chemotherapy combined with anti-EGFR antibodies in vulnerable older patients with metastatic colorectal cancer: Data from the REVOLT study. *J Geriatr Oncol*. 2022 Apr;13(3):302-307. doi: 10.1016/j.jgo.2021.10.007. | Gerardo Rosati |
| Rosier L, Wang Y, Lee JH, Daily K. Does definitive local therapy have a role in select HER2+ de novo metastatic breast cancer patients treated with dual anti-HER2 blockade? *Breast Cancer Res Treat.* 2022 Jan;191(2):375-383. doi: 10.1007/s10549-021-06440-z. | Karen Daily |
| Rostom Y, Abdelmoneim SE, Shaker M, Mahmoud N. Presentation and management of female breast cancer in Egypt. *East Mediterr Health J*. 2022 Oct 30;28(10):725-732. doi: 10.26719/emhj.22.076. | Yousri Rostom |
| Rouyer M, François E, Sa Cunha A, et al. Effectiveness of first-line cetuximab in wild-type RAS metastatic colorectal cancer according to tumour BRAF mutation status from the EREBUS cohort. *Br J Clin Pharmacol*. 2021 Mar;87(3):1120-1128. doi: 10.1111/bcp.14472. | Denis Smith |
| Rozenblit M, Mun S, Soulos P, et al. Patterns of treatment with everolimus exemestane in hormone receptor-positive HER2-negative metastatic breast cancer in the era of targeted therapy. *Breast Cancer Res.* 2021 Jan 29;23(1):14. doi: 10.1186/s13058-021-01394-y. | Sarah Mougalian |
| Rubinstein MM, Dickinson S, Narayan P, et al. Bevacizumab in advanced endometrial cancer. *Gynecol Oncol*. 2021 Jun;161(3):720-726. doi: 10.1016/j.ygyno.2021.04.016. | Maria M. Rubinstein |
| Rugo HS, Brufsky A, Liu X, et al. Real-world study of overall survival with palbociclib plus aromatase inhibitor in HR+/HER2- metastatic breast cancer. *NPJ Breast Cancer*. 2022 Oct 11;8(1):114. doi: 10.1038/s41523-022-00479-x. | Hope S. Rugo |
| Rugo HS, Raskina K, Schrock AB, et al. Biology and Targetability of the Extended Spectrum of PIK3CA Mutations Detected in Breast Carcinoma. *Clin Cancer Res.* 2023 Mar 14;29(6):1056-1067. doi: 10.1158/1078-0432.CCR-22-2115. | Hanna Tukachinsky |
| Rushton M, Kappel C, Lima I, et al. Cardiac Monitoring and Heart Failure in Advanced Breast Cancer Patients Treated With Trastuzumab in Ontario, Canada. *Front Cardiovasc Med.* 2022 May 19;9:850674. doi: 10.3389/fcvm.2022.850674. | Susan Dent |
| Rushton M, Lima I, Tuna M, et al. Impact of Stopping Trastuzumab in Early Breast Cancer: A Population-Based Study in Ontario, Canada. *J Natl Cancer Inst.* 2020 Dec 14;112(12):1222-1230. doi: 10.1093/jnci/djaa054. | Susan Dent |
| Rutkowski P, Indini A, De Luca M, et al. Body mass index (BMI) and outcome of metastatic melanoma patients receiving targeted therapy and immunotherapy: a multicenter international retrospective study. *J Immunother Cancer.* 2020 Nov;8(2):e001117. doi: 10.1136/jitc-2020-001117. | Mario Mandala |
| Sadetsky N, Hernandez A, Wallick CJ, et al. Survival outcomes in an older US population with advanced melanoma and central nervous system metastases: SEER-Medicare analysis. *Cancer Med*. 2020 Sep;9(17):6216-6224. doi: 10.1002/cam4.3256. | Natalia Sadetsky |
| Sagawa T, Sato Y, Hirakawa M, et al. Clinical impact of primary tumour location, early tumour shrinkage, and depth of response in the treatment of metastatic colorectal cancer with first-line chemotherapy plus cetuximab or bevacizumab. *Sci Rep*. 2020 Nov 13;10(1):19815. doi: 10.1038/s41598-020-76756-1. | Yasushi Sato |
| Sait Bakir M, Birge O, Karadag C, et al. Bevacizumab in recurrent ovarian cancer. *J BUON.* 2021 Jul-Aug;26(4):1271-1278. | Mehmet Sait Bakir |
| Saito Y, Takekuma Y, Komatsu Y, Sugawara M. Risk factor analysis for regorafenib-induced severe hypertension in metastatic colorectal cancer treatment. *Support Care Cancer*. 2022 Dec;30(12):10203-10211. doi: 10.1007/s00520-022-07381-z. | Mitsuru Sugawara |
| Saito Y, Tamaki S, Hasegawa H, et al. Safety Evaluation of Initial CT-P6 Administration for 30 min during the Switch from Reference Trastuzumab in Maintenance Infusion: A Multicenter Observational Study. *Biol Pharm Bull*. 2021;44(4):474-477. doi: 10.1248/bpb.b20-00984. | Mitsuru Sugawara |
| Sakaguchi T, Furuya N, Ito K, et al. The efficacy and safety of ramucirumab plus docetaxel in older patients with advanced non-small cell lung cancer. *Thorac Cancer.* 2020 Jun;11(6):1559-1565. doi: 10.1111/1759-7714.13429. | Tadashi Sakaguchi |
| Sakamoto H, Yanagitani N, Manabe R, et al. Characteristics of central nervous system progression in non-small cell lung cancer treated with crizotinib or alectinib. *Cancer Rep (Hoboken).* 2021 Dec;4(6):e1414. doi: 10.1002/cnr2.1414. | Makoto Nishio |
| Sakata Y, Sakata S, Oya Y, et al. Osimertinib as first-line treatment for advanced epidermal growth factor receptor mutation-positive non-small-cell lung cancer in a real-world setting (OSI-FACT). *Eur J Cancer.* 2021 Dec;159:144-153. doi: 10.1016/j.ejca.2021.09.041. | Yoshihiko Sakata |
| Salgia R, Mambetsariev I, Pharaon R, et al. Evaluation of Omics-Based Strategies for the Management of Advanced Lung Cancer. *JCO Oncol Pract*. 2021 Feb;17(2):e257-e265. doi: 10.1200/OP.20.00117. | Ravi Salgia |
| Sangaré L, Divita A, Rehn M, et al. Navigating metastatic colorectal treatment options in the USA: a survey of patient acceptance of skin toxicities associated with Vectibix. *Support Care Cancer.* 2021 Nov;29(11):6731-6740. doi: 10.1007/s00520-021-06134-8. | Kimberly A. Lowe |
| Sanglier T, Fabi A, Flores C, et al. T-DM1 after Pertuzumab plus Trastuzumab: Treatment Sequence-Induced Selection Bias in HER2-Positive Metastatic Breast Cancer. *Cancers (Basel).* 2022 May 17;14(10):2468. doi: 10.3390/cancers14102468. | Thibaut Sanglier |
| Sanglier T, Ross R, Shi T, et al. Trastuzumab-based regimens beyond progression: A crucial treatment option for HER2+ advanced/metastatic breast cancer. *Breast.* 2022 Dec;66:262-271. doi: 10.1016/j.breast.2022.10.008. | Thibaut Sanglier |
| Sansone V, Tovoli F, Casadei-Gardini A, et al. Comparison of Prognostic Scores in Patients With Hepatocellular Carcinoma Treated With Sorafenib. *Clin Transl Gastroenterol*. 2021 Jan 14;12(1):e00286. doi: 10.14309/ctg.0000000000000286. | Vito Sansone |
| Santoni M, Aurilio G, Massari F, et al. Nivolumab VERSUS Cabozantinib as Second-Line Therapy in Patients With Advanced Renal Cell Carcinoma: A Real-World Comparison. *Clin Genitourin Cancer*. 2022 Jun;20(3):285-295. doi: 10.1016/j.clgc.2022.02.003. | Matteo Santoni |
| Santoni M, Massari F, Bracarda S, et al. Body Mass Index in Patients Treated with Cabozantinib for Advanced Renal Cell Carcinoma: A New Prognostic Factor? *Diagnostics (Basel).* 2021 Jan 18;11(1):138. doi: 10.3390/diagnostics11010138. | Matteo Santoni |
| Santoni M, Massari F, Bracarda S, et al. Cabozantinib in Patients with Advanced Renal Cell Carcinoma Primary Refractory to First-line Immunocombinations or Tyrosine Kinase Inhibitors. *Eur Urol Focus*. 2022 Nov;8(6):1696-1702. doi: 10.1016/j.euf.2022.02.004. | Francesco Massari |
| Sasaki A, Kawazoe A, Eto T, et al. Improved efficacy of taxanes and ramucirumab combination chemotherapy after exposure to anti-PD-1 therapy in advanced gastric cancer. *ESMO Open*. 2020 Jul;4(Suppl 2):e000775. doi: 10.1136/esmoopen-2020-000775. | Kohei Shitara |
| Sasaki M, Ishikawa T, Ishiguro M, et al. The effectiveness of plasma miR-33a-5p as a predictive biomarker for the efficacy of colorectal cancer chemotherapy. *Oncol Lett*. 2021 Jun;21(6):489. doi: 10.3892/ol.2021.12749. | Toshiaki Ishikawa |
| Sasaki R, Fukushima M, Haraguchi M, et al. Liver Function in Older Patients With Unresectable Hepatocellular Carcinoma After Administration of Lenvatinib. *Anticancer Res*. 2021 Apr;41(4):2025-2032. doi: 10.21873/anticanres.14970. | Ryu Sasaki |
| Satake H, Kagawa Y, Shinozaki E, et al. Real-World Data Analysis of Second-Line Antiangiogenic Targeted Treatments Following Anti-Epidermal Growth Factor Receptor Monoclonal Antibodies and First-Line FOLFOX for Patients with Metastatic Colorectal Cancer. *Adv Ther.* 2022 Jun;39(6):2596-2613. doi: 10.1007/s12325-022-02122-4. | Yoshinori Tanizawa |
| Sato H, Nagashima H, Akiyama M, et al. Analysis of bevacizumab treatments and metastatic sites of lung cancer. *Cancer Treat Res Commun*. 2021;26:100290. doi: 10.1016/j.ctarc.2020.100290. | Makoto Maemondo |
| Sato J, Uchida M, Wakabayashi H, Shimizu T. Evaluation of Lung Toxicity Related to the Treatment With Alectinib Using a Pharmacovigilance Database. *Anticancer Res.* 2022 Jun;42(6):3109-3116. doi: 10.21873/anticanres.15799. | Junya Sato |
| Sato MT, Ida A, Kanda Y, et al. Prognostic model for overall survival that includes the combination of platelet count and neutrophil-lymphocyte ratio within the first six weeks of sunitinib treatment for metastatic renal cell carcinoma. *BMC Cancer*. 2022 Nov 24;22(1):1214. doi: 10.1186/s12885-022-10316-w. | Miki Takenaka Sato |
| Sato R, Moriguchi M, Iwai K, et al. Real-world outcomes of molecular targeted agents for patients with hepatocellular carcinoma over 80 years old. *Hepatol Res.* 2022 Oct;52(10):859-871. doi: 10.1111/hepr.13818. | Rui Sato |
| Sato Y, Sumikawa H, Shibaki R, et al. Drug-Related Pneumonitis Induced by Osimertinib as First-Line Treatment for Epidermal Growth Factor Receptor Mutation-Positive Non-Small Cell Lung Cancer: A Real-World Setting. *Chest.* 2022 Nov;162(5):1188-1198. doi: 10.1016/j.chest.2022.05.035. | Yuki Sato |
| Savard MF, Wells JC, Graham J, et al. Real-World Assessment of Clinical Outcomes Among First-Line Sunitinib Patients with Clear Cell Metastatic Renal Cell Carcinoma (mRCC) by the International mRCC Database Consortium Risk Group. *Oncologist.* 2020 May;25(5):422-430. doi: 10.1634/theoncologist.2019-0605. | Daniel Y. C. Heng |
| Sawaki M, Taira N, Uemura Y, et al. Adjuvant trastuzumab without chemotherapy for treating early HER2-positive breast cancer in older patients: A propensity score-adjusted analysis of a prospective cohort study. *Breast.* 2022 Dec;66:245-254. doi: 10.1016/j.breast.2022.10.017. | Masataka Sawaki |
| Schaule J, Kroeze SGC, Blanck O, et al. Predicting survival in melanoma patients treated with concurrent targeted- or immunotherapy and stereotactic radiotherapy : Melanoma brain metastases prognostic score. *Radiat Oncol*. 2020 Jun 1;15(1):135. doi: 10.1186/s13014-020-01558-8. | Jana Schaule |
| Scheiner B, Pomej K, Kirstein MM, et al. Prognosis of patients with hepatocellular carcinoma treated with immunotherapy - development and validation of the CRAFITY score. *J Hepatol.* 2022 Feb;76(2):353-363. doi: 10.1016/j.jhep.2021.09.035. | Matthias Pinter |
| Schmid S, Cheng S, Chotai S, et al. Real-World Treatment Sequencing, Toxicities, Health Utilities, and Survival Outcomes in Patients with Advanced ALK-Rearranged Non-Small-Cell Lung Cancer. *Clin Lung Cancer*. 2023 Jan;24(1):40-50. doi: 10.1016/j.cllc.2022.09.007. | Geoffrey Liu |
| Schmidinger M, Porta C, Oudard S, et al. Real-world Experience With Sunitinib Treatment in Patients With Metastatic Renal Cell Carcinoma: Clinical Outcome According to Risk Score. *Clin Genitourin Cancer*. 2020 Oct;18(5):e588-e597. doi: 10.1016/j.clgc.2020.02.013. | Manuela Schmidinger |
| Schmidt M, KÜmmel S, Ruf-Doerdelmann A, et al. Neo-adjuvant and/or Adjuvant Subcutaneous Trastuzumab (Herceptin®) in Patients With Early HER2-positive Breast Cancer: Real World Data from a German Observational Study - (NIS HerSCin). *Anticancer Res*. 2021 Jan;41(1):485-496. doi: 10.21873/anticanres.14799. | Marcus Schmidt |
| Schneeweiss A, Ettl J, Lüftner D, et al. Initial experience with CDK4/6 inhibitor-based therapies compared to antihormone monotherapies in routine clinical use in patients with hormone receptor positive, HER2 negative breast cancer - Data from the PRAEGNANT research network for the first 2 years of drug availability in Germany. *Breast.* 2020 Dec;54:88-95. doi: 10.1016/j.breast.2020.08.011. | Peter A. Fasching |
| Schreier A, Munoz-Arcos L, Alvarez A, et al. Racial disparities in neutrophil counts among patients with metastatic breast cancer during treatment with CDK4/6 inhibitors. *Breast Cancer Res Treat.* 2022 Jul;194(2):337-351. doi: 10.1007/s10549-022-06574-8. | Jesus D. Anampa |
| Schulz MS, Wolf S, Struck V, et al. Anti-EGFR Reintroduction and Rechallenge in Metastatic Colorectal Cancer (mCRC): A Real-World Analysis. *Cancers (Basel).* 2022 Mar 24;14(7):1641. doi: 10.3390/cancers14071641. | Oliver Waidmann |
| Schumann K, Mauch C, Klespe KC, et al. Real-world outcomes using PD-1 antibodies and BRAF + MEK inhibitors for adjuvant melanoma treatment from 39 skin cancer centers in Germany, Austria and Switzerland. *J Eur Acad Dermatol Venereol.* 2023 May;37(5):894-906. doi: 10.1111/jdv.18779. | Christian Posch |
| Seethapathy H, Lee MD, Strohbehn IA, et al. Clinical features of acute kidney injury in patients receiving dabrafenib and trametinib. *Nephrol Dial Transplant*. 2022 Feb 25;37(3):507-514. doi: 10.1093/ndt/gfaa372. | Meghan E. Sise |
| Sehouli J, Mustea A, Oskay-Özcelik G, et al. Bevacizumab Combined with Platinum-Taxane Chemotherapy as First-Line Treatment for Advanced Ovarian Cancer: Results of the NOGGO Non-Interventional Study (OTILIA) in 824 Patients. *Cancers (Basel).* 2021 Sep 22;13(19):4739. doi: 10.3390/cancers13194739. | Jalid Sehouli |
| Sekiguchi S, Tsuchiya K, Yasui Y, et al. Clinical usefulness of geriatric assessment in elderly patients with unresectable hepatocellular carcinoma receiving sorafenib or lenvatinib therapy. *Cancer Rep (Hoboken).* 2022 Nov;5(11):e1613. doi: 10.1002/cnr2.1613. | Namiki Izumi |
| Sekine M, Enomoto T, Watanabe Y, et al. The efficacy and safety profile of 2-weekly dosing of bevacizumab-containing chemotherapy for platinum-resistant recurrent ovarian cancer. *Int J Clin Oncol*. 2021 Nov;26(11):2123-2129. doi: 10.1007/s10147-021-01996-8. | Masayuki Sekine |
| Sertesen E, Yekedüz E, Köksoy EB, et al. The effect of primary tumour resection on patients with synchronous metastatic colorectal cancer treated with cetuximab-containing regimens. *ANZ J Surg.* 2023 Apr;93(4):945-950. doi: 10.1111/ans.18117. | Emre Yekedüz |
| Seung SJ, Hurry M, Walton RN, Evans WK. Real-world treatment patterns and survival in stage IV non-small-cell lung cancer in Canada. *Curr Oncol.* 2020 Aug;27(4):e361-e367. doi: 10.3747/co.27.6049. | S. J. Seung |
| Seystahl K, Hentschel B, Loew S, et al. Bevacizumab versus alkylating chemotherapy in recurrent glioblastoma. *J Cancer Res Clin Oncol.* 2020 Mar;146(3):659-670. doi: 10.1007/s00432-019-03086-9. | Katharina Seystahl |
| Shah KP, Song H, Ye F, Johnson DB. Prognostic Clinical and Radiographic Biomarkers for BRAF-Targeted Therapy in Advanced Melanoma. *Oncologist.* 2021 Feb;26(2):e333-e335. doi: 10.1002/onco.13562. | Douglas B. Johnson |
| Sharma R, Pillai A, Marron TU, et al. Patterns and outcomes of subsequent therapy after immune checkpoint inhibitor discontinuation in HCC. *Hepatol Commun*. 2022 Jul;6(7):1776-1785. doi: 10.1002/hep4.1927. | Rohini Sharma |
| Shayeb AM, McManus HD, Urman D, et al. Cabozantinib Safety With Different Anticoagulants in Patients With Renal Cell Carcinoma. *Clin Genitourin Cancer.* 2023 Feb;21(1):55-62. doi: 10.1016/j.clgc.2022.10.013. | Rana R. McKay |
| Shen B, Jiang H, Wang L, et al. Effectiveness and Safety of Apatinib in Patients with Advanced or Metastatic Adenocarcinoma of Stomach or Gastroesophageal Junction: A Prospective Observation Study. *Onco Targets Ther*. 2020 May 20;13:4457-4464. doi: 10.2147/OTT.S232287. | Jifeng Feng |
| Shen HB, Li J, Yao YS, et al. Impact of Somatic Mutations in Non-Small-Cell Lung Cancer: A Retrospective Study of a Chinese Cohort. *Cancer Manag Res.* 2020 Aug 19;12:7427-7437. doi: 10.2147/CMAR.S254139. | Tian-Jun Hu |
| Shen L, Zhou J, Chen Y, et al. Treatment patterns, effectiveness, and patient-reported outcomes of palbociclib therapy in Chinese patients with advanced breast cancer: A multicenter ambispective real-world study. *Cancer Med*. 2022 Nov;11(22):4157-4168. doi: 10.1002/cam4.4767. | Chao Ni |
| Shenolikar R, Liu S, Shah A, et al. Real-world treatment patterns of metastatic non-small cell lung cancer patients receiving epidermal growth factor receptor tyrosine kinase inhibitors. *Cancer Med*. 2023 Jan;12(1):159-169. doi: 10.1002/cam4.4918. | Aimee Near |
| Shi Y, Zhang X, Wu G, et al. Treatment strategy, overall survival and associated risk factors among patients with unresectable stage IIIB/IV non-small cell lung cancer in China (2015-2017): A multicentre prospective study. *Lancet Reg Health West Pac.* 2022 Apr 11;23:100452. doi: 10.1016/j.lanwpc.2022.100452. | Yuankai Shi |
| Shibaki R, Ozawa Y, Noguchi S, et al. Impact of pre-existing interstitial lung abnormal shadow on lung injury development and severity in patients of non-small cell lung cancer treated with osimertinib. *Cancer Med*. 2022 Oct;11(20):3743-3750. doi: 10.1002/cam4.4750. | Yuichi Ozawa |
| Shields CL, Dalvin LA, Chang M, et al. Visual Outcome at 4 Years Following Plaque Radiotherapy and Prophylactic Intravitreal Bevacizumab (Every 4 Months for 2 Years) for Uveal Melanoma: Comparison With Nonrandomized Historical Control Individuals. *JAMA Ophthalmol.* 2020 Feb 1;138(2):136-146. doi: 10.1001/jamaophthalmol.2019.5132. | Carol L. Shields |
| Shimose S, Hiraoka A, Nakano M, et al. First-line sorafenib sequential therapy and liver disease etiology for unresectable hepatocellular carcinoma using inverse probability weighting: A multicenter retrospective study. *Cancer Med.* 2021 Dec;10(23):8530-8541. doi: 10.1002/cam4.4367. | Shigeo Shimose |
| Shimose S, Iwamoto H, Niizeki T, et al. Clinical Significance of Adverse Events for Patients with Unresectable Hepatocellular Carcinoma Treated with Lenvatinib: A Multicenter Retrospective Study. *Cancers (Basel).* 2020 Jul 11;12(7):1867. doi: 10.3390/cancers12071867. | Shigeo Shimose |
| Shimose S, Iwamoto H, Tanaka M, et al. Alternating Lenvatinib and Trans-Arterial Therapy Prolongs Overall Survival in Patients with Inter-Mediate Stage HepatoCellular Carcinoma: A Propensity Score Matching Study. *Cancers (Basel).* 2021 Jan 5;13(1):160. doi: 10.3390/cancers13010160. | Shigeo Shimose |
| Shimose S, Iwamoto H, Tanaka M, et al. Association between Adverse Events and Prognosis in Patients with Hepatocellular Carcinoma Treated with Atezolizumab Plus Bevacizumab: A Multicenter Retrospective Study. *Cancers (Basel).* 2022 Sep 1;14(17):4284. doi: 10.3390/cancers14174284. | Shigeo Shimose |
| Shimose S, Kawaguchi T, Iwamoto H, et al. Controlling Nutritional Status (CONUT) Score is Associated with Overall Survival in Patients with Unresectable Hepatocellular Carcinoma Treated with Lenvatinib: A Multicenter Cohort Study. *Nutrients.* 2020 Apr 13;12(4):1076. doi: 10.3390/nu12041076. | Takuji Torimura |
| Shimose S, Kawaguchi T, Tanaka M, et al. Lenvatinib prolongs the progression-free survival time of patients with intermediate-stage hepatocellular carcinoma refractory to transarterial chemoembolization: A multicenter cohort study using data mining analysis. *Oncol Lett.* 2020 Sep;20(3):2257-2265. doi: 10.3892/ol.2020.11758. | Shigeo Shimose |
| Shimozato N, Namisaki T, Okano A, et al. Efficacy and Safety of Lenvatinib for Patients With Advanced Hepatocellular Carcinoma: A Retrospective, Real-world Study Conducted in Japan. *Anticancer Res*. 2022 Jan;42(1):173-183. doi: 10.21873/anticanres.15471. | Tadashi Namisaki |
| Shin SJ, Lee JL, Kwon TG, et al. Real-World Study Evaluating Safety and Effectiveness of Axitinib in Korean Patients with Renal Cell Carcinoma after Failure of One Prior Systemic Therapy. *Cancer Res Treat.* 2023 Apr;55(2):643-651. doi: 10.4143/crt.2022.883. | Se Hoon Park |
| Shinano H, Miyazaki S, Miura K, et al. Risk Profiling of Cancer Treatment-Related Cardiovascular Disorders in Breast Cancer Patients Who Received Adjuvant Chemotherapy With Trastuzumab. *Circ Rep.* 2020 Mar 24;2(4):235-242. doi: 10.1253/circrep.CR-19-0119. | Kazuhiro Sase |
| Shindoh J, Kawamura Y, Kobayashi M, et al. Prognostic Advantages of Individual Additional Interventions After Lenvatinib Therapy in Patients with Advanced Hepatocellular Carcinoma. *J Gastrointest Surg*. 2022 Aug;26(8):1637-1646. doi: 10.1007/s11605-022-05388-9. | Junichi Shindoh |
| Shinozaki E, Makiyama A, Kagawa Y, et al. Treatment sequences of patients with advanced colorectal cancer and use of second-line FOLFIRI with antiangiogenic drugs in Japan: A retrospective observational study using an administrative database. *PLoS One.* 2021 Feb 8;16(2):e0246160. doi: 10.1371/journal.pone.0246160. | Yoshinori Tanizawa |
| Shiozawa T, Numata T, Tamura T, et al. Prognostic Implication of PD-L1 Expression on Osimertinib Treatment for EGFR-mutated Non-small Cell Lung Cancer. *Anticancer Res.* 2022 May;42(5):2583-2590. doi: 10.21873/anticanres.15736. | Toshihiro Shiozawa |
| Sho T, Suda G, Ogawa K, et al. Lenvatinib in patients with unresectable hepatocellular carcinoma who do not meet the REFLECT trial eligibility criteria. *Hepatol Res.* 2020 Aug;50(8):966-977. doi: 10.1111/hepr.13511. | Goki Suda |
| Shoji T, Takatori E, Nagasawa T, et al. Comparison of treatment outcomes between first-line chemotherapy with or without bevacizumab for advanced ovarian, fallopian tube, and primary peritoneal cancer (Tohoku gynecologic cancer unit: TGCU-RS001 study). *Int J Clin Oncol.* 2022 Dec;27(12):1874-1880. doi: 10.1007/s10147-022-02246-1. | Tadahiro Shoji |
| Shokoohi A, Al-Hashami Z, Moore S, et al. Effect of targeted therapy and immunotherapy on advanced nonsmall-cell lung cancer outcomes in the real world. *Cancer Med.* 2022 Jan;11(1):86-93. doi: 10.1002/cam4.4427. | Cheryl Ho |
| Shu Y, Ding Y, Dai B, Zhang Q. A real-world pharmacovigilance study of axitinib: data mining of the public version of FDA adverse event reporting system. *Expert Opin Drug Saf*. 2022 Apr;21(4):563-572. doi: 10.1080/14740338.2022.2016696. | Qilin Zhang |
| Shu Y, He X, Liu Y, et al. A Real-World Disproportionality Analysis of Olaparib: Data Mining of the Public Version of FDA Adverse Event Reporting System*. Clin Epidemiol*. 2022 Jun 28;14:789-802. doi: 10.2147/CLEP.S365513. | Qilin Zhang |
| Shuanggang C, Shen L, Qiu Z, et al. Transarterial chemoembolization combined with microwave ablation and apatinib in patients with Barcelona clinic liver cancer Stage C hepatocellular carcinoma: A propensity score matching analysis. *J Cancer Res Ther*. 2020;16(2):250-257. doi: 10.4103/jcrt.JCRT_345_19. | Weijun Fan |
| Singal AG, Nagar SP, Hitchens A, et al. Real-world effectiveness of lenvatinib monotherapy in previously treated unresectable hepatocellular carcinoma in US clinical practice. *Cancer Rep (Hoboken).* 2023 Jan;6(1):e1679. doi: 10.1002/cnr2.1679. | Amit G. Singal |
| Singal AG, Nagar SP, Hitchens A, et al. Real-world effectiveness of lenvatinib monotherapy among unresectable hepatocellular carcinoma patients in the USA. *Future Oncol.* 2021 Jul;17(21):2759-2768. doi: 10.2217/fon-2021-0242. | Amit G. Singal |
| Sinn DH, Lee HW, Paik YH, et al. Patterns and Outcomes in Hepatocellular Carcinoma Patients with Portal Vein Invasion: A Multicenter Prospective Cohort Study. *Dig Dis Sci.* 2021 Jan;66(1):315-324. doi: 10.1007/s10620-020-06134-4. | Yong-Han Paik |
| Slootbeek PHJ, Kloots ISH, Smits M, et al. Impact of molecular tumour board discussion on targeted therapy allocation in advanced prostate cancer. *Br J Cancer.* 2022 Apr;126(6):907-916. doi: 10.1038/s41416-021-01663-9. | Niven Mehra |
| Smith D, Lepage C, Vicaut E, et al. Observational Study in a Real-World Setting of Targeted Therapy in the Systemic Treatment of Progressive Unresectable or Metastatic Well-Differentiated Pancreatic Neuroendocrine Tumors (pNETs) in France: OPALINE Study. *Adv Ther*. 2022 Jun;39(6):2731-2748. doi: 10.1007/s12325-022-02103-7. | Denis Smith |
| Smyth EN, Beyrer J, Saverno KR, et al. Real-World Patient Characteristics, Utilization Patterns, and Outcomes of US Patients with HR+, HER2- Metastatic Breast Cancer Treated with Abemaciclib. *Drugs Real World Outcomes*. 2022 Dec;9(4):681-693. doi: 10.1007/s40801-022-00327-1. | Emily Nash Smyth |
| Sobu R, Numakura K, Naito S, et al. Clinical impact of early response to first-line VEGFR-TKI in patients with metastatic renal cell carcinoma on survival: A multi-institutional retrospective study. *Cancer Med.* 2023 Feb;12(4):4100-4109. doi: 10.1002/cam4.5268. | Kazuyuki Numakura |
| Song X, Lu J, Wang L, et al. Efficacy of everolimus combined with endocrine therapy in HR-positive/HER-2-negative advanced breast cancer. *J BUON.* 2020 Sep-Oct;25(5):2228-2236. | Haixia Shan |
| Song ZZ, Zhao LF, Zuo J, et al. Clinical Outcomes and Safety of Apatinib Mesylate in the Treatment of Advanced Non-Squamous Non-Small Cell Lung Cancer in Patients Who Progressed After Standard Therapy and Analysis of the KDR Gene Polymorphism. *Onco Targets Ther.* 2020 Jan 21;13:603-613. doi: 10.2147/OTT.S222985. | Yu-Dong Wang |
| Soria A, Calvo M, Casas M, et al. Survival and adverse events of elderly patients treated with sorafenib for hepatocellular carcinoma. *Front Oncol.* 2022 Aug 2;12:829483. doi: 10.3389/fonc.2022.829483. | Mercedes Vergara |
| Sousa LG, Wang K, Torman D, et al. Treatment patterns and outcomes of palliative systemic therapy in patients with salivary duct carcinoma and adenocarcinoma, not otherwise specified. *Cancer.* 2022 Feb 1;128(3):509-518. doi: 10.1002/cncr.33968. | Renata Ferrarotto |
| Stanbouly D, Philipone E, Morlandt AB, et al. Adverse events secondary to cetuximab therapy in head & neck cancer therapy and risk factors for serious outcomes. *Oral Oncol*. 2022 Aug;131:105952. doi: 10.1016/j.oraloncology.2022.105952. | Dani Stanbouly |
| Stares M, Swan A, Cumming K, et al. Hypoalbuminaemia as a Prognostic Biomarker of First-Line Treatment Resistance in Metastatic Non-small Cell Lung Cancer. *Front Nutr.* 2021 Oct 1;8:734735. doi: 10.3389/fnut.2021.734735. | Iain Phillips |
| Stefanini B, Bucci L, Santi V, et al. Potential feasibility of atezolizumab-bevacizumab therapy in patients with hepatocellular carcinoma treated with tyrosine-kinase inhibitors. *Dig Liver Dis.* 2022 Nov;54(11):1563-1572. doi: 10.1016/j.dld.2022.07.003. | Franco Trevisani |
| Steger GG, Egle D, Bartsch R, et al. Efficacy and safety of everolimus plus exemestane in patients with HR+, HER2- advanced breast cancer progressing on/after prior endocrine therapy in routine clinical practice: Primary results from the non-interventional study, STEPAUT. *Breast*. 2020 Apr;50:64-70. doi: 10.1016/j.breast.2020.01.035. | Guenther G. Steger |
| Stein SM, Snider J, Ali SM, et al. Real-world association of HER2/ERBB2 concordance with trastuzumab clinical benefit in advanced esophagogastric cancer. *Future Oncol*. 2021 Nov;17(31):4101-4114. doi: 10.2217/fon-2021-0203. | Jeremy Snider |
| Stift J, Graf A, Neudert B, et al. Immune checkpoints and liver resection after neoadjuvant chemotherapy including bevacizumab in patients with microsatellite-stable colorectal liver metastases. *HPB (Oxford).* 2022 Jan;24(1):40-46. doi: 10.1016/j.hpb.2021.05.015. | Stefan Stremitzer |
| Straś W, Gotlib J, Małkowski P, et al. Overall Survival in Patients with Hepatocellular Carcinoma Treated with Sorafenib: A Polish Experience. *Med Sci Monit.* 2021 Aug 31;27:e931856. doi: 10.12659/MSM.931856. | Piotr Małkowski |
| Su C, Zhou J, Qiang H, et al. Special issue "The advance of solid tumor research in China": Real-world clinical outcomes of alectinib for advanced nonsmall-cell lung cancer patients with ALK fusion in China. *Int J Cancer*. 2023 Jan 1;152(1):15-23. doi: 10.1002/ijc.34123. | Tianqing Chu |
| Su VY, Yang KY, Huang TY, et al. The efficacy of first-line tyrosine kinase inhibitors combined with co-medications in Asian patients with EGFR mutation non-small cell lung cancer. *Sci Rep.* 2020 Sep 11;10(1):14965. doi: 10.1038/s41598-020-71583-w. | Yuh-Lih Chang |
| Su WL, Chuang SC, Wang YC, et al. Expression of FOXM1 and Aurora-A predicts prognosis and sorafenib efficacy in patients with hepatocellular carcinoma. *Cancer Biomark*. 2020;28(3):341-350. doi: 10.3233/CBM-190507. | Kung-Kai Kuo |
| Su YC, Wu CC, Su CC, et al. Comparative Effectiveness of Bevacizumab versus Cetuximab in Metastatic Colorectal Cancer Patients without Primary Tumor Resection. *Cancers (Basel).* 2022 Apr 24;14(9):2118. doi: 10.3390/cancers14092118. | Yea-Huei Kao Yang |
| Su YL, Tsai KL, Chiu TJ, et al. Development and Validation of a Novel Serum Prognostic Marker for Patients with Metastatic Colorectal Cancer on Regorafenib Treatment. *Cancers (Basel).* 2021 Oct 11;13(20):5080. doi: 10.3390/cancers13205080. | Hung-Chih Hsu |
| Sueoka-Aragane N, Nakashima C, Yoshida H, et al. The role of comprehensive analysis with circulating tumor DNA in advanced non-small cell lung cancer patients considered for osimertinib treatment. *Cancer Med*. 2021 Jun;10(12):3873-3885. doi: 10.1002/cam4.3929. | Naoko Sueoka-Aragane |
| Sugimoto S, Ishida T, Kawada K, et al. Central Nervous System Ischemia Associated with Bevacizumab: An Analysis of the Japanese Adverse Drug Event Report Database. *Biol Pharm Bull.* 2022;45(12):1805-1811. doi: 10.1248/bpb.b22-00496. | Shohei Sugimoto |
| Sugiyama S, Sato K, Shibasaki Y, et al. Real-world use of temsirolimus in Japanese patients with unresectable or metastatic renal cell carcinoma: recent consideration based on the results of a post-marketing, all-case surveillance study. *Jpn J Clin Oncol.* 2020 Aug 4;50(8):940-947. doi: 10.1093/jjco/hyaa062. | Kazuo Sato |
| Sugiyama T, Katsumata N, Toita T, et al. Incidence of fistula occurrence in patients with cervical cancer treated with bevacizumab: data from real-world clinical practice*. Int J Clin Oncol.* 2022 Sep;27(9):1517-1528. doi: 10.1007/s10147-022-02196-8. | Toru Sugiyama |
| Suh HJ, Flórez Á, Sacristán V, et al. Cutaneous adverse events in patients receiving anticancer therapy in a tertiary hospital setting: the old and the new. *Int J Dermatol*. 2021 Feb;60(2):208-216. doi: 10.1111/ijd.15081. | Hae-Jin Suh |
| Sukrithan V, Barbaro A, Chergui A, et al. Differential Efficacy of Anti-VEGF Antibodies Based on Sex and Race in a Diverse Cohort of Advanced Nonsquamous Non-Small Cell Lung Cancer. *Am J Clin Oncol.* 2020 Jan;43(1):64-68. doi: 10.1097/COC.0000000000000628. | Sanjay Goel |
| Sun J, Zhong X, Ma J, et al. Real-world benefit of combination palbociclib and endocrine therapy for metastatic breast cancer and correlation with neutropenia. *Cancer Med.* 2021 Nov;10(21):7665-7672. doi: 10.1002/cam4.4295. | Hung T. Khong |
| Sun L, Candelieri-Surette D, Anglin-Foote T, et al. Cetuximab-Based vs Carboplatin-Based Chemoradiotherapy for Patients With Head and Neck Cancer. *JAMA Otolaryngol Head Neck Surg.* 2022 Nov 1;148(11):1022-1028. doi: 10.1001/jamaoto.2022.2791. | Lova Sun |
| Sun L, Dai J, Chen Y, et al. Pulmonary Sarcomatoid Carcinoma: Experience From SEER Database and Shanghai Pulmonary Hospital. *Ann Thorac Surg.* 2020 Aug;110(2):406-413. doi: 10.1016/j.athoracsur.2020.02.071. | Peng Zhang |
| Suntheralingam S, Fan CS, Calvillo-Argüelles O, et al. Evaluation of Risk Prediction Models to Identify Cancer Therapeutics Related Cardiac Dysfunction in Women with HER2+ Breast Cancer. *J Clin Med.* 2022 Feb 5;11(3):847. doi: 10.3390/jcm11030847. | Paaladinesh Thavendiranathan |
| Suo J, Sun Y, Fu Y, et al. A Retrospective Analysis of the Effect of Anlotinib in Patients With Lung Cancer With or Without Previous Antiangiogenic Therapy. *Front Oncol*. 2021 Dec 23;11:788837. doi: 10.3389/fonc.2021.788837. | Jiang Zhu |
| Svaton M, Blazek J, Krakorova G, et al. Laboratory Parameters are Possible Prognostic Markers in Patients with Advanced-stage NSCLC Treated with Bevacizumab plus Chemotherapy. *J Cancer*. 2021 Jul 30;12(19):5753-5759. doi: 10.7150/jca.58851. | Martin Svaton |
| Svaton M, Blazek J, Krakorova G, et al. Prognostic Role for CYFRA 21-1 in Patients With Advanced-stage NSCLC Treated With Bevacizumab Plus Chemotherapy. *Anticancer Res.* 2021 Apr;41(4):2053-2058. doi: 10.21873/anticanres.14974. | Martin Svaton |
| Svaton M, Bratova M, Fischer O, et al. Real-life Effectiveness of Afatinib Versus Gefitinib in Patients With Non-small-cell Lung Cancer: A Czech Multicentre Study. *Anticancer Res.* 2021 Apr;41(4):2059-2065. doi: 10.21873/anticanres.14975. | Martin Svaton |
| Svaton M, Fiala O, Krakorova G, et al. Thyroid transcription factor 1 and p63 expression is associated with survival outcome in patients with non-small cell lung cancer treated with erlotinib. *Oncol Lett.* 2020 Aug;20(2):1376-1382. doi: 10.3892/ol.2020.11663. | Martin Svaton |
| Taboada RG, Riechelmann RP, Mauro C, et al. Everolimus-Induced Pneumonitis in Patients with Neuroendocrine Neoplasms: Real-World Study on Risk Factors and Outcomes. *Oncologist*. 2022 Mar 4;27(2):97-103. doi: 10.1093/oncolo/oyab024. | Juan W. Valle |
| Tada T, Kumada T, Hiraoka A, et al. C-reactive protein to albumin ratio predicts survival in patients with unresectable hepatocellular carcinoma treated with lenvatinib. *Sci Rep.* 2022 May 19;12(1):8421. doi: 10.1038/s41598-022-12058-y. | Toshifumi Tada |
| Tada T, Kumada T, Hiraoka A, et al. Impact of Early Lenvatinib Administration on Survival in Patients with Intermediate-Stage Hepatocellular Carcinoma: A Multicenter, Inverse Probability Weighting Analysis. *Oncology*. 2021;99(8):518-527. doi: 10.1159/000515896. | Toshifumi Tada |
| Tai CC, Chen WS, Jiang JK, et al. Comparing Late-line Treatment Sequence of Regorafenib and Reduced-intensity FOLFOXIRI for Refractory Metastatic Colorectal Cancer. *Am J Clin Oncol.* 2020 Jan;43(1):28-34. doi: 10.1097/COC.0000000000000637. | Hao-Wei Teng |
| Tak KY, Nam HC, Choi JY, et al. Effectiveness of sorafenib dose modifications on treatment outcome of hepatocellular carcinoma: Analysis in real-life settings. *Int J Cancer*. 2020 Oct 1;147(7):1970-1978. doi: 10.1002/ijc.32964. | Jeong Won Jang |
| Takahara K, Ando R, Kanao K, et al. Prognostic Stratification of the IMDC Intermediate Risk Group After Treatment With First-line Molecular-targeted Therapy for Metastatic Renal Cell Carcinoma. *Anticancer Res*. 2020 Aug;40(8):4395-4400. doi: 10.21873/anticanres.14443. | Kiyoshi Takahara |
| Takahashi A, Moriguchi M, Seko Y, et al. Early Tumor Shrinkage as a Predictive Factor for Outcomes in Hepatocellular Carcinoma Patients Treated with Lenvatinib: A Multicenter Analysis. *Cancers (Basel).* 2020 Mar 23;12(3):754. doi: 10.3390/cancers12030754. | Michihisa Moriguchi |
| Takahashi S, Tahara M, Ito K, et al. Safety and Effectiveness of Lenvatinib in 594 Patients with Unresectable Thyroid Cancer in an All-Case Post-Marketing Observational Study in Japan. *Adv Ther*. 2020 Sep;37(9):3850-3862. doi: 10.1007/s12325-020-01433-8. | Shunji Takahashi |
| Tamura K, Ando R, Takahara K, et al. Development of novel ACN (albumin, C-reactive protein and neutrophil-to-lymphocyte ratio) prognostication model for patients with metastatic renal cell carcinoma receiving first-line molecular-targeted therapy. *Urol Oncol.* 2021 Jan;39(1):78.e1-78.e8. doi: 10.1016/j.urolonc.2020.08.029. | Hideaki Miyake |
| Tamura K, Osawa T, Takeuchi A, et al. External validation of the albumin, C-reactive protein and lactate dehydrogenase model in patients with metastatic renal cell carcinoma receiving second-line axitinib therapy in a Japanese multi-center cohort. *Jpn J Clin Oncol*. 2021 Apr 30;51(5):810-818. doi: 10.1093/jjco/hyaa264. | Keita Tamura |
| Tan AD, Willemsma K, MacNeill A, et al. Tyrosine kinase inhibitors significantly improved survival outcomes in patients with metastatic gastrointestinal stromal tumour: a multi-institutional cohort study. *Curr Oncol.* 2020 Jun;27(3):e276-e282. doi: 10.3747/co.27.5869. | Christine E. Simmons |
| Tanabe T, Shida D, Boku N, et al. Primary Tumor-Related Complications Among Patients With Unresectable Stage IV Colorectal Cancer in the Era of Targeted Therapy: A Competing Risk Regression Analysis. *Dis Colon Rectum*. 2021 Sep 1;64(9):1074-1082. doi: 10.1097/DCR.0000000000002010. | Dai Shida |
| Tanaka I, Morise M, Miyazawa A, et al. Potential Benefits of Bevacizumab Combined With Platinum-Based Chemotherapy in Advanced Non-Small-Cell Lung Cancer Patients With EGFR Mutation. *Clin Lung Cancer.* 2020 May;21(3):273-280.e4. doi: 10.1016/j.cllc.2020.01.011. | Ichidai Tanaka |
| Tanaka R, Ishikawa H, Sato J, et al. Prevention of Acne-Like Eruption Caused by Panitumumab Treatment through Oral Administration of Non-steroidal Anti-inflammatory Drugs. *Biol Pharm Bull.* 2022;45(10):1531-1536. doi: 10.1248/bpb.b22-00404. | Rei Tanaka |
| Tanaka T, Hiraoka A, Tada T, et al. Therapeutic efficacy of atezolizumab plus bevacizumab treatment for unresectable hepatocellular carcinoma in patients with Child-Pugh class A or B liver function in real-world clinical practice. *Hepatol Res.* 2022 Sep;52(9):773-783. doi: 10.1111/hepr.13797. | Atusushi Hiraoka |
| Tanaka T, Kuzuya T, Ishigami M, et al. Efficacy and Safety of Sorafenib in Unresectable Hepatocellular Carcinoma with Bile Duct Invasion. *Oncology.* 2020;98(9):621-629. doi: 10.1159/000507051. | Teiji Kuzuya |
| Tang B, Mo J, Yan X, et al. Real-world efficacy and safety of axitinib in combination with anti-programmed cell death-1 antibody for advanced mucosal melanoma. *Eur J Cancer*. 2021 Oct;156:83-92. doi: 10.1016/j.ejca.2021.07.018. | Xinan Sheng |
| Tang M, Schaffer AL, Kiely BE, et al. Cardiac assessment in Australian patients receiving (neo)adjuvant trastuzumab for HER2-positive early breast cancer: a population-based study. *Breast Cancer Res Treat.* 2021 Jun;187(3):893-902. doi: 10.1007/s10549-021-06135-5. | Monica Tang |
| Tang M, Song C, Zhang Y, et al. Levels of pretreatment blood lipids are prognostic factors in advanced NSCLC patients treated with anlotinib. *Lipids Health Dis*. 2021 Nov 20;20(1):165. doi: 10.1186/s12944-021-01596-5. | Tian Chen |
| Tang T, Abu-Sbeih H, Ma W, et al. Gastrointestinal Injury Related to Antiangiogenesis Cancer Therapy. *Clin Colorectal Cancer.* 2020 Sep;19(3):e117-e123. doi: 10.1016/j.clcc.2020.03.002. | Yinghong Wang |
| Tang X, Li Y, Qian WL, et al. A comprehensive prognostic analysis of osimertinib treatment in advanced non-small cell lung cancer patients with acquired EGFR-T790M mutation: a real-world study. *J Cancer Res Clin Oncol.* 2022 Sep;148(9):2475-2486. doi: 10.1007/s00432-021-03797-y. | Zhi-Gang Yang |
| Tang Y, Xia B, Xie R, et al. Timing in combination with radiotherapy and patterns of disease progression in non-small cell lung cancer treated with EGFR-TKI. *Lung Cancer.* 2020 Feb;140:65-70. doi: 10.1016/j.lungcan.2019.12.009. | Shenglin Ma |
| Taniyama Y, Oze I, Koyanagi YN, et al. Changes in survival of patients with non-small cell lung cancer in Japan: An interrupted time series study. *Cancer Sci*. 2023 Mar;114(3):1154-1164. doi: 10.1111/cas.15646. | Hidemi Ito |
| Tashiro R, Kawazoe H, Mamishin K, et al. Patient-associated risk factors for severe anemia in patients with advanced ovarian or breast cancer receiving olaparib monotherapy: A multicenter retrospective study. *Front Oncol.* 2022 Oct 4;12:898150. doi: 10.3389/fonc.2022.898150. | Hitoshi Kawazoe |
| Taza F, Holler AE, Fu W, et al. Differential Activity of PARP Inhibitors in BRCA1- Versus BRCA2-Altered Metastatic Castration-Resistant Prostate Cancer. *JCO Precis Oncol*. 2021 Jul 22;5:PO.21.00070. doi: 10.1200/PO.21.00070. | Emmanuel S. Antonarakis |
| Temraz S, Nasr F, Kattan J, et al. A Non-Interventional Multicenter Study of First-Line Bevacizumab in Combination with Chemotherapy in Patients with Metastatic Colorectal Cancer in Lebanon. *Biologics.* 2022 Feb 21;16:7-15. doi: 10.2147/BTT.S340525. | Ali Shamseddine |
| Ten Berge DMHJ, Aarts MJ, Groen HJM, et al. A population-based study describing characteristics, survival and the effect of TKI treatment on patients with EGFR mutated stage IV NSCLC in the Netherlands. *Eur J Cancer.* 2022 Apr;165:195-204. doi: 10.1016/j.ejca.2022.01.038. | Deirdre M. H. J. Ten Berge |
| Teshima Y, Kizaki M, Kurihara R, et al. Interim analysis for post-marketing surveillance of dabrafenib and trametinib combination therapy in Japanese patients with unresectable and metastatic melanoma with BRAF V600 mutation. *Int J Clin Oncol.* 2020 Oct;25(10):1870-1878. doi: 10.1007/s10147-020-01737-3. | Yasutomo Teshima |
| Teshima Y, Nomura S, Fukasawa N. Postmarketing observational study of pazopanib in patients with metastatic soft tissue sarcoma in Japan. *Jpn J Clin Oncol*. 2021 Apr 1;51(4):612-621. doi: 10.1093/jjco/hyaa208. | Yasutomo Teshima |
| Teyateeti A, Mahvash A, Long J, et al. Disease control and failure patterns of unresectable hepatocellular carcinoma following transarterial radioembolization with yttrium-90 microspheres and with/without sorafenib. *World J Gastroenterol.* 2021 Dec 21;27(47):8166-8181. doi: 10.3748/wjg.v27.i47.8166. | Srinivas Cheenu Kappadath |
| Thielmann CM, Matull J, Zaremba A, et al. TERT promoter mutations are associated with longer progression-free and overall survival in patients with BRAF-mutant melanoma receiving BRAF and MEK inhibitor therapy. *Eur J Cancer.* 2022 Jan;161:99-107. doi: 10.1016/j.ejca.2021.11.009. | Klaus G. Griewank |
| Thiem A, Mashhadiakbar P, Cussigh C, et al. Immune checkpoint inhibition and targeted therapy for melanoma: A patient-oriented cross-sectional comparative multicentre study. *J Eur Acad Dermatol Venereol*. 2023 May;37(5):884-893. doi: 10.1111/jdv.18778. | Alexander Thiem |
| Thill M, Wimberger P, Grafe A, et al. Dual HER2 blockade with pertuzumab (P) and trastuzumab (T) in patients with HER2-positive metastatic breast cancer (mBC) relapsing after adjuvant treatment with T: results from a German non-interventional study (NIS) HELENA (NCT01777958). *Breast Cancer Res Treat.* 2022 Nov;196(2):311-321. doi: 10.1007/s10549-022-06710-4. | Marc Thill |
| Tiainen S, Rilla K, Hämäläinen K, et al. The prognostic and predictive role of the neutrophil-to-lymphocyte ratio and the monocyte-to-lymphocyte ratio in early breast cancer, especially in the HER2+ subtype. *Breast Cancer Res Treat*. 2021 Jan;185(1):63-72. doi: 10.1007/s10549-020-05925-7. | Satu Tiainen |
| Tian G, Zhao X, Nie J, et al. The overall survival benefit in Chinese ALK+ NSCLC patients received targeted therapies. *J Thorac Dis*. 2022 Jun;14(6):2201-2212. doi: 10.21037/jtd-22-622. | Jian Fang |
| Tian X, Chen L, Gai D, et al. Adverse Event Profiles of PARP Inhibitors: Analysis of Spontaneous Reports Submitted to FAERS. *Front Pharmacol*. 2022 Mar 25;13:851246. doi: 10.3389/fphar.2022.851246. | Ni Zhang |
| Tinsley N, Zhou C, Nahm S, et al. Antibiotic use reduces efficacy of tyrosine kinase inhibitors in patients with advanced melanoma and non-small-cell lung cancer. *ESMO Open.* 2022 Jun;7(3):100430. doi: 10.1016/j.esmoop.2022.100430. | Natalie Cook |
| Tomczak A, Springfeld C, Dill MT, et al. Precision oncology for intrahepatic cholangiocarcinoma in clinical practice. *Br J Cancer.* 2022 Nov;127(9):1701-1708. doi: 10.1038/s41416-022-01932-1. | Thomas Longerich |
| Tomonari T, Sato Y, Tani J, et al. Comparison of therapeutic outcomes of sorafenib and lenvatinib as primary treatments for hepatocellular carcinoma with a focus on molecular-targeted agent sequential therapy: A propensity score-matched analysis. *Hepatol Res.* 2021 Apr;51(4):472-481. doi: 10.1111/hepr.13597. | Yasushi Sato |
| Tran HT, Lam VK, Elamin YY, et al. Clinical Outcomes in Non-Small-Cell Lung Cancer Patients Treated With EGFR-Tyrosine Kinase Inhibitors and Other Targeted Therapies Based on Tumor Versus Plasma Genomic Profiling. *JCO Precis Oncol*. 2021 Aug 5;5:PO.20.00532. doi: 10.1200/PO.20.00532. | John V. Heymach |
| Tredan O, Laurent M, Gilberg M, et al. Innovative Approach for a Typology of Treatment Sequences in Early Stage HER2 Positive Breast Cancer Patients Treated With Trastuzumab in the French National Hospital Database. *Cancer Inform.* 2022 Nov 9;21:11769351221135134. doi: 10.1177/11769351221135134. | Marie Laurent |
| Trunk A, Braithwaite M, Nevala-Plagemann C, et al. Real-World Outcomes of Patients With BRAF-Mutated Metastatic Colorectal Cancer Treated in the United States*. J Natl Compr Canc Netw.* 2022 Feb;20(2):144-150. doi: 10.6004/jnccn.2021.7059. | Ignacio Garrido-Laguna |
| Tsai HY, Chang HP, Chen CJ, et al. Effects of direct-acting antiviral therapy for patients with advanced hepatocellular carcinoma and concomitant hepatitis C-A population-based cohort study. *Eur Rev Med Pharmacol Sci*. 2021 Dec;25(23):7543-7552. doi: 10.26355/eurrev_202112_27454. | Po-Chang Lee |
| Tsai JS, Su PL, Yang SC, et al. EGFR-TKI plus bevacizumab versus EGFR-TKI monotherapy for patients with EGFR mutation-positive advanced non-small cell lung cancer-A propensity score matching analysis. *J Formos Med Assoc*. 2021 Sep;120(9):1729-1739. doi: 10.1016/j.jfma.2021.03.023. | Chien-Chung Lin |
| Tseng LC, Chen KH, Wang CL, Weng LC. Effects of tyrosine kinase inhibitor therapy on skin toxicity and skin-related quality of life in patients with lung cancer: An observational study. *Medicine (Baltimore*). 2020 Jun 5;99(23):e20510. doi: 10.1097/MD.0000000000020510. | Li-Chueh Weng |
| Tsuchiya K, Kurosaki M, Sakamoto A, et al. The Real-World Data in Japanese Patients with Unresectable Hepatocellular Carcinoma Treated with Lenvatinib from a Nationwide Multicenter Study. *Cancers (Basel).* 2021 May 26;13(11):2608. doi: 10.3390/cancers13112608. | Namiki Izumi |
| Tu X, Yang J, Zheng Y, et al. Immunotherapy combination with regorafenib for refractory hepatocellular carcinoma: A real-world study. *Int Immunopharmacol*. 2022 Dec;113(Pt B):109401. doi: 10.1016/j.intimp.2022.109401. | Yun Zheng |
| Turner S, Chia S, Kanakamedala H, et al. Effectiveness of Alpelisib + Fulvestrant Compared with Real-World Standard Treatment Among Patients with HR+, HER2-, PIK3CA-Mutated Breast Cancer. *Oncologist.* 2021 Jul;26(7):e1133-e1142. doi: 10.1002/onco.13804. | Hope S. Rugo |
| Turnsek N, Devjak R, Edelbaher N, et al. Real-world outcomes, treatment patterns and T790M testing rates in non-small cell lung cancer patients treated with first-line first- or second-generation epidermal growth factor receptor tyrosine kinase inhibitors from the Slovenian cohort of the REFLECT study. *Radiol Oncol*. 2022 Aug 14;56(3):371-379. doi: 10.2478/raon-2022-0025. | Nina Turnsek |
| Uchida M, Nakano K, Fujiwara M, et al. Comprehensive analysis of everolimus-induced adverse events using the Japanese real-world database. *J Clin Pharm Ther*. 2022 Aug;47(8):1173-1180. doi: 10.1111/jcpt.13648. | Tadashi Shimizu |
| Udagawa C, Kuah S, Shimoi T, et al. Replication Study for the Association of Five SNPs Identified by GWAS and Trastuzumab-Induced Cardiotoxicity in Japanese and Singaporean Cohorts. *Biol Pharm Bull.* 2022;45(8):1198-1202. doi: 10.1248/bpb.b22-00136. | Hitoshi Zembutsu |
| Ueda A, Watari H, Mandai M, et al. Incidence of gastrointestinal perforation associated with bevacizumab in combination with neoadjuvant chemotherapy as first-line treatment of advanced ovarian, fallopian tube, or peritoneal cancer: analysis of a Japanese healthcare claims database. *J Gynecol Oncol*. 2022 Nov;33(6):e78. doi: 10.3802/jgo.2022.33.e78. | Akihiko Ueda |
| Ueda K, Ogasawara N, Yonekura S, et al. The Prognostic Value of Systemic Inflammatory Markers in Advanced Renal Cell Carcinoma Patients Treated With Molecular Targeted Therapies. *Anticancer Res*. 2020 Mar;40(3):1739-1745. doi: 10.21873/anticanres.14127. | Kosuke Ueda |
| Ueda K, Suekane S, Kurose H, et al. Improved Survival of Real-world Japanese Patients With Advanced Renal Cell Carcinoma Treated With Immuno-oncology Combination Therapy. *Anticancer Res.* 2022 Sep;42(9):4573-4580. doi: 10.21873/anticanres.15960. | Kosuke Ueda |
| Ueshima K, Ogasawara S, Ikeda M, et al. Hepatic Arterial Infusion Chemotherapy versus Sorafenib in Patients with Advanced Hepatocellular Carcinoma. *Liver Cancer.* 2020 Sep;9(5):583-595. doi: 10.1159/000508724. | Sadahisa Ogasawara |
| Uojima H, Chuma M, Tanaka Y, et al. Skeletal Muscle Mass Influences Tolerability and Prognosis in Hepatocellular Carcinoma Patients Treated with Lenvatinib. *Liver Cancer.* 2020 Apr;9(2):193-206. doi: 10.1159/000504604. | Haruki Uojima |
| Upadhyay VA, Johnson BE, Landman AB, Hassett MJ. Real-World Analysis of Off-Label Use of Molecularly Targeted Therapy in a Large Academic Medical Center Cohort. *JCO Precis Oncol.* 2022 Jan;6:e2100232. doi: 10.1200/PO.21.00232. | Michael J. Hassett |
| Upshaw JN, Finkelman B, Hubbard RA, et al. Comprehensive Assessment of Changes in Left Ventricular Diastolic Function With Contemporary Breast Cancer Therapy. *JACC Cardiovasc Imaging.* 2020 Jan;13(1 Pt 2):198-210. doi: 10.1016/j.jcmg.2019.07.018. | Bonnie Ky |
| Valcarcel S, Gallego J, Jimenez-Fonseca P, et al. Does HER2 status influence in the benefit of ramucirumab and paclitaxel as second line treatment of advanced gastro-esophageal adenocarcinoma? Data from the AGAMENON-SEOM registry. *J Cancer Res Clin Oncol.* 2023 Jul;149(7):4077-4089. doi: 10.1007/s00432-022-04294-6. | Sena Valcarcel |
| Vallerio P, Orenti A, Tosi F, et al. Major adverse cardiovascular events associated with VEGF-targeted anticancer tyrosine kinase inhibitors: a real-life study and proposed algorithm for proactive management. *ESMO Open.* 2022 Feb;7(1):100338. doi: 10.1016/j.esmoop.2021.100338. | Patrizia Boracchi |
| van Breeschoten J, Ismail RK, Wouters MWJM, et al. End-of-Life Use of Systemic Therapy in Patients With Advanced Melanoma: A Nationwide Cohort Study. *JCO Oncol Pract.* 2022 Oct;18(10):e1611-e1620. doi: 10.1200/OP.22.00061. | Jesper van Breeschoten |
| van der Kooij MK, Dekkers OM, Aarts MJB, et al. Sex-Based Differences in Treatment with Immune Checkpoint Inhibition and Targeted Therapy for Advanced Melanoma: A Nationwide Cohort Study. *Cancers (Basel).* 2021 Sep 16;13(18):4639. doi: 10.3390/cancers13184639. | Ellen Kapiteijn |
| van der Kooij MK, Wetzels MJAL, Aarts MJB, et al. Age Does Matter in Adolescents and Young Adults versus Older Adults with Advanced Melanoma; A National Cohort Study Comparing Tumor Characteristics, Treatment Pattern, Toxicity and Response. *Cancers (Basel).* 2020 Jul 27;12(8):2072. doi: 10.3390/cancers12082072. | Ellen Kapiteijn |
| van Laar SA, Gombert-Handoko KB, Groenwold RHH, et al. Real-World Metastatic Renal Cell Carcinoma Treatment Patterns and Clinical Outcomes in The Netherlands. *Front Pharmacol.* 2022 Mar 23;13:803935. doi: 10.3389/fphar.2022.803935. | J. Zwaveling |
| Van Luan P, Tien ND, Hai NM, et al. Real-world analysis of the effect of gefitinib as a first-line therapy in patients with advanced non-small cell lung cancer with *EGFR* mutations. *Ther Adv Med Oncol.* 2021 Feb 20;13:1758835921992977. doi: 10.1177/1758835921992977. | Pham Van Luan |
| van Zeijl MCT, de Wreede LC, van den Eertwegh AJM, et al. Survival outcomes of patients with advanced melanoma from 2013 to 2017: Results of a nationwide population-based registry. *Eur J Cancer.* 2021 Feb;144:242-251. doi: 10.1016/j.ejca.2020.11.028. | J. B. A. G. Haanen |
| Vano YA, Phan L, Gravis G, et al. Cabozantinib-nivolumab sequence in metastatic renal cell carcinoma: The CABIR study. *Int J Cancer.* 2022 Oct 15;151(8):1335-1344. doi: 10.1002/ijc.34126. | Yann-Alexandre Vano |
| Vasista A, Ryan L, Naher S, et al. Survival and cardiac toxicity in patients with HER2-positive, metastatic breast cancer treated with trastuzumab in routine clinical practice. *Asia Pac J Clin Oncol.* 2020 Feb;16(1):34-38. doi: 10.1111/ajco.13280. | Anuradha Vasista |
| Vasseur A, Carton M, Guiu S, et al. Efficacy of taxanes rechallenge in first-line treatment of early metastatic relapse of patients with HER2-negative breast cancer previously treated with a (neo)adjuvant taxanes regimen: A multicentre retrospective observational study. *Breast.* 2022 Oct;65:136-144. doi: 10.1016/j.breast.2022.07.014. | Luc Cabel |
| Vazdar L, Gabrić ID, Kruljac I, et al. Influence of Ile655Val polymorphism on trastuzumab-induced cardiotoxicity in early-stage HER2 positive breast cancer. *Sci Rep.* 2021 Jul 13;11(1):14395. doi: 10.1038/s41598-021-93634-6. | Lora Stanka Kirigin Biloš |
| Veitch Z, Ribnikar D, Tilley D, et al. No evidence of disease versus residual disease in long-term responders to first-line HER2-targeted therapy for metastatic breast cancer. *Br J Cancer.* 2022 Apr;126(6):881-888. doi: 10.1038/s41416-021-01676-4. | Zachary Veitch |
| Venugopal B, Pillai M, Powles T, et al. Early Clinical Experience with Cabozantinib for Advanced Renal Cell Carcinoma in the UK: Real-World Treatment Pathways and Clinical Outcomes. *Clin Genitourin Cancer*. 2022 Feb;20(1):94-94.e10. doi: 10.1016/j.clgc.2021.09.005. | Balaji Venugopal |
| Vera R, Mata E, González E, et al. Is aflibercept an optimal treatment for wt RAS mCRC patients after progression to first line containing anti-EGFR? *Int J Colorectal Dis.* 2020 Apr;35(4):739-746. doi: 10.1007/s00384-020-03509-x. | Ruth Vera |
| Verdaguer H, Saurí T, Acosta DA, et al. ESMO Scale for Clinical Actionability of Molecular Targets Driving Targeted Treatment in Patients with Cholangiocarcinoma. *Clin Cancer Res*. 2022 Apr 14;28(8):1662-1671. doi: 10.1158/1078-0432.CCR-21-2384. | Teresa Macarulla |
| Vernieri C, Nichetti F, Lalli L, et al. Impact of Baseline and On-Treatment Glycemia on Everolimus-Exemestane Efficacy in Patients with Hormone Receptor-Positive Advanced Breast Cancer (EVERMET). *Clin Cancer Res.* 2021 Jun 15;27(12):3443-3455. doi: 10.1158/1078-0432.CCR-20-4928. | Claudio Vernieri |
| Vitale P, Zanaletti N, Famiglietti V, et al. Retrospective Study of Regorafenib Versus TAS-102 Efficacy and Safety in Chemorefractory Metastatic Colorectal Cancer (mCRC) Patients: A Multi-institution Real Life Clinical Data. *Clin Colorectal Cancer.* 2021 Sep;20(3):227-235. doi: 10.1016/j.clcc.2021.06.002. | Stefania Napolitano |
| Voog E, Campillo-Gimenez B, Elkouri C, et al. Long survival of patients with metastatic clear cell renal cell carcinoma. Results of real life study of 344 patients. *Int J Cancer*. 2020 Mar 15;146(6):1643-1651. doi: 10.1002/ijc.32578. | Eric Voog |
| Wadd N, Peedell C, Polwart C. Real-World Assessment of Cancer Drugs Using Local Data Uploaded to the Systemic Anti-Cancer Therapy Dataset in England. *Clin Oncol (R Coll Radiol).* 2022 Aug;34(8):497-507. doi: 10.1016/j.clon.2022.04.012. | C. Polwart |
| Wagener-Ryczek S, Heydt C, Süptitz J, et al. Mutational spectrum of acquired resistance to reversible versus irreversible EGFR tyrosine kinase inhibitors. *BMC Cancer*. 2020 May 12;20(1):408. doi: 10.1186/s12885-020-06920-3. | Svenja Wagener-Ryczek |
| Waliany S, Zhu H, Wakelee H, et al. Pharmacovigilance Analysis of Cardiac Toxicities Associated With Targeted Therapies for Metastatic NSCLC. *J Thorac Oncol*. 2021 Dec;16(12):2029-2039. doi: 10.1016/j.jtho.2021.07.030. | Joel W. Neal |
| Wallrabenstein T, Del Rio J, Templeton AJ, Buess M. Much has changed in the last decade except overall survival: A Swiss single center analysis of treatment and survival in patients with stage IV non-small cell lung cancer. *PLoS One*. 2020 May 29;15(5):e0233768. doi: 10.1371/journal.pone.0233768. | Martin Buess |
| Wan YN, Chen HM, Liu XF, et al. Elevated pretreatment neutrophil-to-lymphocyte ratio indicate low survival rate in apatinib-treated patients with non-small cell lung cancer: A STROBE-compliant article. *Medicine (Baltimore)*. 2022 Nov 25;101(47):e32043. doi: 10.1097/MD.0000000000032043. | Yi-Yu Lu |
| Wang C, Sandhu J, Fakih M. Mucinous Histology Is Associated with Resistance to Anti-EGFR Therapy in Patients with Left-Sided RAS/BRAF Wild-Type Metastatic Colorectal Cancer. *Oncologist.* 2022 Mar 4;27(2):104-109. doi: 10.1093/oncolo/oyab028. | Marwan Fakih |
| Wang DG, Barrios DM, Blinder VS, et al. Dermatologic adverse events related to the PI3Kα inhibitor alpelisib (BYL719) in patients with breast cancer. *Breast Cancer Res Treat.* 2020 Aug;183(1):227-237. doi: 10.1007/s10549-020-05726-y. | Mario E. Lacouture |
| Wang F, Jin F, Cheng B, et al. The real-world efficacy and safety of anlotinib in advanced non-small cell lung cancer. *J Cancer Res Clin Oncol*. 2022 Jul;148(7):1721-1735. doi: 10.1007/s00432-021-03752-x. | Fen Wang |
| Wang H, Liu D, Wang C, et al. Transarterial chemoembolization (TACE) plus apatinib-combined therapy versus TACE alone in the treatment of intermediate to advanced hepatocellular carcinoma patients: A real-world study. *Clin Res Hepatol Gastroenterol.* 2022 Jun-Jul;46(6):101869. doi: 10.1016/j.clinre.2022.101869. | Hui Wang |
| Wang H, Wang Z, Hou Z, et al. The Neutrophil-to-Lymphocyte Ratio (NLR) Predicts the Prognosis of Unresectable Intermediate and Advanced Hepatocellular Carcinoma Treated with Apatinib. *Cancer Manag Res*. 2021 Sep 7;13:6989-6998. doi: 10.2147/CMAR.S311526. | Ti Zhang |
| Wang HC, Liu PL, Lo PC, et al. Consistent administration of cetuximab is associated with favorable outcomes in recurrent/metastatic head and neck squamous cell carcinoma in an endemic carcinogen exposure area: a retrospective observational study. *PeerJ.* 2020 Sep 10;8:e9862. doi: 10.7717/peerj.9862. | Shih-Feng Cho |
| Wang L, Wang X, Huang M, et al. High-risk-pattern lung adenocarcinoma with epidermal growth factor receptor mutation is associated with distant metastasis risk and may benefit from adjuvant targeted therapy. *Interact Cardiovasc Thorac Surg.* 2021 Aug 18;33(3):395-401. doi: 10.1093/icvts/ivab099. | Yue Yang |
| Wang N, Yu X. Comparison between talazoparib and conventional chemotherapy in the treatment of HER2-positive breast cancer patients: A retrospective study. *Front Immunol.* 2022 Aug 15;13:901636. doi: 10.3389/fimmu.2022.901636. | Xiaopeng Yu |
| Wang S, Chen M, Zhang X, et al. Aneurysm and Artery Dissection Following the Use of Vascular Endothelial Growth Factor Inhibitor: A Real-World Analysis Using a Spontaneous Reporting System. *J Am Heart Assoc.* 2021 Dec 7;10(23):e020844. doi: 10.1161/JAHA.121.020844. | Jian Gong |
| Wang W, Gu X, Si J, et al. Treatment outcomes and prognosis of patients with primary and acquired BRAF-mutated non-small cell lung cancer: A multicenter retrospective study. *Genes Chromosomes Cancer.* 2022 Sep;61(9):530-541. doi: 10.1002/gcc.23043. | Zhengbo Song |
| Wang X, Cai J, Zeng Z, Liu A. Efficacy of osimertinib for preventing leptomeningeal metastasis derived from advanced EGFR-mutated non-small cell lung cancer: a propensity-matched retrospective study. *BMC Cancer*. 2021 Jul 30;21(1):873. doi: 10.1186/s12885-021-08581-2. | Anwen Liu |
| Wang X, Chen D, Qiu J, et al. The relationship between the degree of brain edema regression and changes in cognitive function in patients with recurrent glioma treated with bevacizumab and temozolomide. *Quant Imaging Med Surg.* 2021 Nov;11(11):4556-4568. doi: 10.21037/qims-20-1084. | Xianglian Wang |
| Wang X, Haaland B, Hu-Lieskovan S, et al. First line immunotherapy extends brain metastasis free survival, improves overall survival, and reduces the incidence of brain metastasis in patients with advanced melanoma. *Cancer Rep (Hoboken).* 2021 Dec;4(6):e1419. doi: 10.1002/cnr2.1419. | Sheri L. Holmen |
| Wang X, Wang L, Yu Q, et al. The Effectiveness of Lapatinib in HER2-Positive Metastatic Breast Cancer Patients Pretreated With Multiline Anti-HER2 Treatment: A Retrospective Study in China. *Technol Cancer Res Treat*. 2021 Jan-Dec;20:15330338211037812. doi: 10.1177/15330338211037812. | Zhiyong Yu |
| Wang X, Yu J, Yang M, et al. Safety and effectiveness of apatinib in patients with previously treated metastatic gastric cancer: a sub-analysis from the real-world study of apatinib for gastric cancer treatment (AHEAD-G202). *Am J Cancer Res.* 2020 Mar 1;10(3):987-996. | Chunmei Bai |
| Wang X, Zhang R, Du N, et al. An open label, multicenter, noninterventional study of apatinib in advanced gastric cancer patients (AHEAD-G202). *Ther Adv Med Oncol.* 2020 Mar 19;12:1758835920905424. doi: 10.1177/1758835920905424. | Chunmei Bai |
| Wang X, Zhong D, Zhang J, et al. Safety and effectiveness of apatinib in elderly patients with metastatic gastric cancer: a sub-analysis from the large-scale, prospective observational study of apatinib for gastric cancer treatment in a real-world clinical setting (AHEAD-G202). *J Gastrointest Oncol.* 2022 Aug;13(4):1679-1689. doi: 10.21037/jgo-22-727. | Mei Guan |
| Wang Y, Shen S, Hu P, et al. Alectinib versus crizotinib in ALK-positive advanced non-small cell lung cancer and comparison of next-generation TKIs after crizotinib failure: Real-world evidence. *Cancer Med*. 2022 Dec;11(23):4491-4500. doi: 10.1002/cam4.4834. | Xingya Li |
| Wang Y, Singh K, Dizon D, et al. Immunohistochemical HER2 score correlates with response to neoadjuvant chemotherapy in HER2-positive primary breast cancer. *Breast Cancer Res Treat.* 2021 Apr;186(3):667-676. doi: 10.1007/s10549-021-06124-8. | Evgeny Yakirevich |
| Wang Z, Wang E, Bai W, et al. Exploratory Analysis to Identify Candidates Benefitting from Combination Therapy of Transarterial Chemoembolization and Sorafenib for First-Line Treatment of Unresectable Hepatocellular Carcinoma: A Multicenter Retrospective Observational Study. *Liver Cancer.* 2020 Jun;9(3):308-325. doi: 10.1159/000505692. | Guohong Han |
| Waterhouse DM, Espirito JL, Chioda MD, et al. Retrospective Observational Study of ALK-Inhibitor Therapy Sequencing and Outcomes in Patients with ALK-Positive Non-small Cell Lung Cancer. *Drugs Real World Outcomes.* 2020 Dec;7(4):261-269. doi: 10.1007/s40801-020-00207-6. | David M. Waterhouse |
| Watson NW, Wander SA, Shatzel JJ, Al-Samkari H. Venous and arterial thrombosis associated with abemaciclib therapy for metastatic breast cancer. *Cancer.* 2022 Sep 1;128(17):3224-3232. doi: 10.1002/cncr.34367. | Hanny Al-Samkari |
| Wei J, Xiang J, Hao Y, et al. Baseline anemia predicts a poor prognosis in patients with non-small cell lung cancer with epidermal growth factor receptor mutations: a retrospective study. *BMC Pulm Med.* 2022 Oct 17;22(1):381. doi: 10.1186/s12890-022-02158-w. | Zhengbo Song |
| Wei S, Han Y, Zeng H, et al. Radiomics diagnosed histopathological growth pattern in prediction of response and 1-year progression free survival for colorectal liver metastases patients treated with bevacizumab containing chemotherapy. *Eur J Radiol.* 2021 Sep;142:109863. doi: 10.1016/j.ejrad.2021.109863. | Yi Wang |
| Welland S, Leyh C, Finkelmeier F, et al. Real-World Data for Lenvatinib in Hepatocellular Carcinoma (ELEVATOR): A Retrospective Multicenter Study. *Liver Cancer*. 2022 Jan 14;11(3):219-232. doi: 10.1159/000521746. | Arndt Vogel |
| Wells JC, Dudani S, Gan CL, et al. Clinical Effectiveness of Second-line Sunitinib Following Immuno-oncology Therapy in Patients with Metastatic Renal Cell Carcinoma: A Real-world Study. *Clin Genitourin Cancer*. 2021 Aug;19(4):354-361. doi: 10.1016/j.clgc.2021.03.006. | Daniel Y. C. Heng |
| Wells JC, Graham J, Beuselinck B, et al. Clinical Outcomes of First-line Sunitinib Followed by Immuno-oncology Checkpoint Inhibitors in Patients With Metastatic Renal Cell Carcinoma. *Clin Genitourin Cancer*. 2020 Aug;18(4):e350-e359. doi: 10.1016/j.clgc.2019.12.007. | Daniel Y. C. Heng |
| West MT, Smith CE, Kaempf A, et al. CDK 4/6 inhibitors are associated with a high incidence of thrombotic events in women with breast cancer in real-world practice*. Eur J Haematol.* 2021 May;106(5):634-642. doi: 10.1111/ejh.13590. | Malinda T. West |
| Westphalen CB, Kukiolka T, Garlipp B, et al. Correlation of skin rash and overall survival in patients with pancreatic cancer treated with gemcitabine and erlotinib - results from a non-interventional multi-center study. *BMC Cancer*. 2020 Feb 24;20(1):155. doi: 10.1186/s12885-020-6636-7. | C. Benedikt Westphalen |
| Wetzel CL, Sutton TL, Gardiner S, et al. Loss of HER2-positivity following neoadjuvant targeted therapy for breast cancer is not associated with inferior oncologic outcomes. *J Surg Oncol*. 2021 Dec;124(8):1224-1234. doi: 10.1002/jso.26646. | Jennifer R. Garreau |
| White MN, Piper-Vallillo AJ, Gardner RM, et al. Chemotherapy Plus Immunotherapy Versus Chemotherapy Plus Bevacizumab Versus Chemotherapy Alone in EGFR-Mutant NSCLC After Progression on Osimertinib. *Clin Lung Cancer*. 2022 May;23(3):e210-e221. doi: 10.1016/j.cllc.2021.11.001. | Heather A. Wakelee |
| Whitworth PW, Beitsch PD, Murray MK, et al. Genomic Classification of HER2-Positive Patients With 80-Gene and 70-Gene Signatures Identifies Diversity in Clinical Outcomes With HER2-Targeted Neoadjuvant Therapy. *JCO Precis Oncol.* 2022 Sep;6:e2200197. doi: 10.1200/PO.22.00197. | William Audeh |
| Winfree KB, Sheffield KM, Cui ZL, et al. Study of patient characteristics, treatment patterns, EGFR testing patterns and outcomes in real-world patients with EGFRm+ non-small cell lung cancer. *Curr Med Res Opin*. 2022 Jan;38(1):91-99. doi: 10.1080/03007995.2021.1983530. | Katherine B. Winfree |
| Wise J, Tiwari R, O'Halloran S, et al. Time trends for drug specific adverse events in patients on sunitinib; implications for remote monitoring. *Can J Urol*. 2022 Jun;29(3):11136-11141. | Raj Tiwari |
| Wong V, de Boer R, Baron-Hay S, et al. Real-World Outcomes of Ribociclib and Aromatase Inhibitor Use in First Line Hormone Receptor Positive, HER2-Negative Metastatic Breast Cancer. *Clin Breast Cancer*. 2022 Dec;22(8):792-800. doi: 10.1016/j.clbc.2022.08.011. | Vanessa Wong |
| Wong V, Lee M, Wong R, et al. BRAFV600E Mutations Arising from a Left-Side Primary in Metastatic Colorectal Cancer: Are They a Distinct Subset? *Target Oncol.* 2021 Mar;16(2):227-236. doi: 10.1007/s11523-021-00793-7. | Vanessa Wong |
| Wu CE, Chang CF, Huang CY, et al. Comparison of Different Tyrosine Kinase Inhibitors for Treatment of Poor Performance Status Patients with EGFR-Mutated Lung Adenocarcinoma. *Cancers (Basel).* 2022 Jan 28;14(3):674. doi: 10.3390/cancers14030674. | John Wen-Cheng Chan |
| Wu CH, Liang PC, Hsu CH, et al. Total skeletal, psoas and rectus abdominis muscle mass as prognostic factors for patients with advanced hepatocellular carcinoma. *J Formos Med Assoc.* 2021 Jan;120(1 Pt 2):559-566. doi: 10.1016/j.jfma.2020.07.005. | Yu-Yun Shao |
| Wu J, Wang Z, Jin C, et al. Effect of cetuximab combined with chemotherapy in treating metastatic colorectal cancer and its prognostic analysis. *J BUON.* 2021 Jan-Feb;26(1):101-108. | Yanping Hu |
| Wu M, Gong J, Yu W, et al. Effect of bevacizumab combined with docetaxel in the treatment of HER-2-negative recurrent metastatic breast cancer. *J BUON.* 2020 Jul-Aug;25(4):1814-1820. | Xiangdong Kong |
| Wu PY, Cheng YM, Shen MR, et al. Real-World Study of Adding Bevacizumab to Chemotherapy for Ovarian, Tubal, and Peritoneal Cancer as Front-Line or Relapse Therapy (ROBOT): 8-Year Experience. *Front Oncol.* 2020 Jul 14;10:1095. doi: 10.3389/fonc.2020.01095. | Yu-Fang Huang |
| Wu SG, Chiang CL, Liu CY, et al. An Observational Study of Acquired *EGFR* T790M-Dependent Resistance to EGFR-TKI Treatment in Lung Adenocarcinoma Patients in Taiwan. *Front Oncol.* 2020 Sep 4;10:1481. doi: 10.3389/fonc.2020.01481. | Jin-Yuan Shih |
| Wu YL, Fulgenzi CAM, D'Alessio A, et al. Neutrophil-to-Lymphocyte and Platelet-to-Lymphocyte Ratios as Prognostic Biomarkers in Unresectable Hepatocellular Carcinoma Treated with Atezolizumab plus Bevacizumab. *Cancers (Basel).* 2022 Nov 26;14(23):5834. doi: 10.3390/cancers14235834. | Celina Ang |
| Xia D, Bai W, Wang E, et al. Lenvatinib with or without Concurrent Drug-Eluting Beads Transarterial Chemoembolization in Patients with Unresectable, Advanced Hepatocellular Carcinoma: A Real-World, Multicenter, Retrospective Study. *Liver Cancer*. 2022 Mar 9;11(4):368-382. doi: 10.1159/000523849. | Guohong Han |
| Xia WL, Zhao XH, Guo Y, et al. Transarterial chemoembolization combined with apatinib with or without PD-1 inhibitors in BCLC stage C hepatocellular carcinoma: A multicenter retrospective study. *Front Oncol.* 2022 Sep 30;12:961394. doi: 10.3389/fonc.2022.961394. | Hai-Liang Li |
| Xiang YJ, Wang K, Yu HM, et al. Transarterial chemoembolization plus a PD-1 inhibitor with or without lenvatinib for intermediate-stage hepatocellular carcinoma. *Hepatol Res.* 2022 Aug;52(8):721-729. doi: 10.1111/hepr.13773. | Shu-Qun Cheng |
| Xiao J, Liang J, Zhang W, Li Y. Clinical observation of apatinib-related hypothyroidism in patients with advanced malignancies. *Exp Ther Med.* 2020 Sep;20(3):1961-1966. doi: 10.3892/etm.2020.8937. | Yan Li |
| Xiao Y, Ding J, Ma D, et al. Predicting Pathological Complete Response in Neoadjuvant Dual Blockade With Trastuzumab and Pertuzumab in HER2 Gene Amplified Breast Cancer. *Front Immunol*. 2022 May 19;13:877825. doi: 10.3389/fimmu.2022.877825. | Keda Yu |
| Xie QY, Huang LP, Gao FW, et al. Efficacy of lenvatinib combined with sequential transarterial chemoembolization for primary hepatocellular carcinoma and the effects on serum basic fibroblast growth factor and vascular endothelial growth factor. *Front Pharmacol.* 2022 Oct 21;13:965770. doi: 10.3389/fphar.2022.965770. | Feng-Wei Gao |
| Xie Y, Ge R, Sang D, et al. Real-world data of lapatinib and treatment after lapatinib in patients with previously treated HER2-positive metastatic breast cancer: A multicenter, retrospective study. *Cancer Med*. 2020 May;9(9):2981-2988. doi: 10.1002/cam4.2943. | Peng Yuan |
| Xie Y, Li Y, Ting L, et al. Pyrotinib Plus Vinorelbine Versus Lapatinib Plus Capecitabine in Patients With Previously Treated HER2-Positive Metastatic Breast Cancer: A Multicenter, Retrospective Study. *Front Oncol*. 2021 Aug 5;11:699333. doi: 10.3389/fonc.2021.699333. | Biyun Wang |
| Xie Y, Wu S, Zhang Y, et al. Optimal Duration of Neoadjuvant Taxane and Carboplatin Combined With Anti-HER2 Targeted Therapy for HER2-Positive Breast Cancer. *Front Oncol.* 2021 Jun 8;11:686591. doi: 10.3389/fonc.2021.686591. | Guangyu Liu |
| Xin S, Zhao Y, Wang C, et al. Polymorphisms of NF-κB pathway genes influence adverse drug reactions of gefitinib in NSCLC patients. *Pharmacogenomics J.* 2020 Apr;20(2):285-293. doi: 10.1038/s41397-019-0115-z. | Li Zhang |
| Xu D, Liu Y, Tang W, et al. Regorafenib in Refractory Metastatic Colorectal Cancer: A Multi-Center Retrospective Study. *Front Oncol.* 2022 Mar 30;12:838870. doi: 10.3389/fonc.2022.838870. | Jianmin Xu |
| Xu D, Zhang Z, Zhang S, et al. Efficacy of trastuzumab combined with SOX or IP chemotherapy regimen in the treatment of advanced gastric cancer. *J BUON*. 2021 May-Jun;26(3):932-939. | Zhimei Zhang |
| Xu H, Zhang Q, Liang L, et al. Crizotinib vs platinum-based chemotherapy as first-line treatment for advanced non-small cell lung cancer with different ROS1 fusion variants. *Cancer Med.* 2020 May;9(10):3328-3336. doi: 10.1002/cam4.2984. | Yan Wang |
| Xu L, Liu Y, Fan Z, et al. Assessment of CPS + EG, Neo-Bioscore and Modified Neo-Bioscore in Breast Cancer Patients Treated With Preoperative Systemic Therapy: A Multicenter Cohort Study. *Front Oncol.* 2021 Mar 16;11:606477. doi: 10.3389/fonc.2021.606477. | Xuening Duan |
| Xu YB, Zhang Y, Song Z, et al. Treatment and Prognosis of Solid and Cystic Brain Metastases in Patients with Non-Small-Cell Lung Cancer. *Cancer Manag Res.* 2021 Aug 10;13:6309-6317. doi: 10.2147/CMAR.S314060. | Lan Shao |
| Xu YJ, Lai ZC, He MK, et al. Toripalimab Combined With Hepatic Arterial Infusion Chemotherapy Versus Lenvatinib for Advanced Hepatocellular Carcinoma. *Technol Cancer Res Treat.* 2021 Jan-Dec;20:15330338211063848. doi: 10.1177/15330338211063848. | Qi-Jiong Li |
| Xue M, Wu Y, Zhu B, et al. Advanced hepatocellular carcinoma treated by transcatheter arterial chemoembolization with drug-eluting beads plus lenvatinib versus sorafenib, a propensity score matching retrospective study. *Am J Cancer Res.* 2021 Dec 15;11(12):6107-6118. | Wenzhe Fan |
| Yamaguchi S, Ishi Y, Motegi H, et al. The prognostic improvement of add-on bevacizumab for progressive disease during concomitant temozolomide and radiation therapy in patients with glioblastoma and anaplastic astrocytoma. *J Neurosurg Sci.* 2020 Dec;64(6):502-508. doi: 10.23736/S0390-5616.18.04463-6. | Hiroyuki Kobayashi |
| Yamaguchi S, Motegi H, Ishi Y, et al. Clinical Outcome of Cytoreductive Surgery Prior to Bevacizumab for Patients with Recurrent Glioblastoma: A Single-center Retrospective Analysis. *Neurol Med Chir (Tokyo).* 2021 Apr 15;61(4):245-252. doi: 10.2176/nmc.oa.2020-0308. | Shigeru Yamaguchi |
| Yamamoto G, Asahina H, Honjo O, et al. First-line osimertinib in elderly patients with epidermal growth factor receptor-mutated advanced non-small cell lung cancer: a retrospective multicenter study (HOT2002). *Sci Rep.* 2021 Nov 30;11(1):23140. doi: 10.1038/s41598-021-02561-z. | Hajime Asahina |
| Yamamoto N, Mera T, Märten A, Hochmair MJ. Observational Study of Sequential Afatinib and Osimertinib in EGFR Mutation-Positive NSCLC: Patients Treated with a 40-mg Starting Dose of Afatinib. *Adv Ther.* 2020 Feb;37(2):759-769. doi: 10.1007/s12325-019-01187-y. | Nobuyuki Yamamoto |
| Yamamoto Y, Yamashiro H, Schneeweiss A, et al. Factors affecting prognosis in patients treated with bevacizumab plus paclitaxel as first-line chemotherapy for HER2-negative metastatic breast cancer: an international pooled analysis of individual patient data from four prospective observational studies. *Breast Cancer.* 2023 Jan;30(1):88-100. doi: 10.1007/s12282-022-01399-1. | Yutaka Yamamoto |
| Yamamoto Y, Yamashiro H, Toh U, et al. Prospective observational study of bevacizumab combined with paclitaxel as first- or second-line chemotherapy for locally advanced or metastatic breast cancer: the JBCRG-C05 (B-SHARE) study. *Breast Cancer.* 2021 Jan;28(1):145-160. doi: 10.1007/s12282-020-01138-4. | Yutaka Yamamoto |
| Yamaoka K, Fujiwara M, Uchida M, et al. Comprehensive Analysis of Adverse Events Induced by PARP Inhibitors Using JADER and Time to Onset. *Life (Basel).* 2022 Aug 31;12(9):1355. doi: 10.3390/life12091355. | Tadashi Shimizu |
| Yamashiro H, Iwata H, Masuda N, et al. Outcomes of trastuzumab therapy in HER2-positive early breast cancer patients: extended follow-up of JBCRG-cohort study 01. *Breast Cancer.* 2020 Jul;27(4):631-641. doi: 10.1007/s12282-020-01057-4. | Hiroyasu Yamashiro |
| Yamazaki K, Yuki S, Oki E, et al. Real-World Evidence on Second-Line Treatment of Metastatic Colorectal Cancer Using Fluoropyrimidine, Irinotecan, and Angiogenesis Inhibitor. *Clin Colorectal Cancer.* 2021 Sep;20(3):e173-e184. doi: 10.1016/j.clcc.2021.03.001. | Kentaro Yamazaki |
| Yan Z, Gu YY, Hu XD, et al. Clinical outcomes and safety of apatinib monotherapy in the treatment of patients with advanced epithelial ovarian carcinoma who progressed after standard regimens and the analysis of the VEGFR2 polymorphism. *Oncol Lett.* 2020 Sep;20(3):3035-3045. doi: 10.3892/ol.2020.11857. | Wei Duan |
| Yang C, Chen W, Gong G, et al. Application of CT radiomics features to predict the EGFR mutation status and therapeutic sensitivity to TKIs of advanced lung adenocarcinoma. *Transl Cancer Res.* 2020 Nov;9(11):6683-6690. doi: 10.21037/tcr-20-1216. | Yong Yin |
| Yang F, Huang X, Sun C, et al. Lapatinib in combination with capecitabine versus continued use of trastuzumab in breast cancer patients with trastuzumab-resistance: a retrospective study of a Chinese population. *BMC Cancer*. 2020 Mar 29;20(1):255. doi: 10.1186/s12885-020-6639-4. | Zefei Jiang |
| Yang G, Sun H, Zhou C, et al. PD-1 inhibitor monotherapy versus combination therapy: A real-world study of patients with recurrent or metastatic advanced esophageal squamous cell carcinoma after first-line chemotherapy. *J Cancer Res Ther*. 2022 Apr;18(2):545-552. doi: 10.4103/jcrt.jcrt_125_22. | Baosheng Li |
| Yang G, Xu H, Hu J, et al. Specific HER2 Exon 20 Gly776 Deletion-Insertions in Non-Small Cell Lung Cancer: Structural Analysis and Sensitivity to HER2-Targeted Tyrosine Kinase Inhibitors. *Front Pharmacol.* 2022 Mar 7;13:806737. doi: 10.3389/fphar.2022.806737. | Yan Wang |
| Yang G, Yang Y, Liu R, et al. First-line immunotherapy or angiogenesis inhibitor combined with chemotherapy for advanced non-small cell lung cancer with EGFR exon 20 insertions: Real-world evidence from China. *Cancer Med.* 2023 Jan;12(1):335-344. doi: 10.1002/cam4.4852. | Yan Wang |
| Yang H, Wang W. Comparison of pyrotinib or lapatinib with chemotherapy for patients with HER2 positive breast cancer after first-line treatment failure: a retrospective study. *Am J Transl Res.* 2021 Sep 15;13(9):10863-10870. | Wei Wang |
| Yang H, Zhou Z, Lin L, et al. Characterization of MET exon 14 alteration and association with clinical outcomes of crizotinib in Chinese lung cancers. *Lung Cancer.* 2020 Oct;148:113-121. doi: 10.1016/j.lungcan.2020.08.009. | Nong Yang |
| Yang JC, Schuler M, Popat S, et al. Afatinib for the Treatment of Non-Small Cell Lung Cancer Harboring Uncommon EGFR Mutations: An Updated Database of 1023 Cases Brief Report. *Front Oncol.* 2022 Apr 28;12:834704. doi: 10.3389/fonc.2022.834704. | James Chih-Hsin Yang |
| Yang S, Mao S, Li X, et al. Uncommon EGFR mutations associate with lower incidence of T790M mutation after EGFR-TKI treatment in patients with advanced NSCLC. *Lung Cancer.* 2020 Jan;139:133-139. doi: 10.1016/j.lungcan.2019.11.018. | Shengxiang Ren |
| Yang S, Xiao J, Liu Q, et al. The Sequence of Intracranial Radiotherapy and Systemic Treatment With Tyrosine Kinase Inhibitors for Gene-Driven Non-Small Cell Lung Cancer Brain Metastases in the Targeted Treatment Era: A 10-Year Single-Center Experience. *Front Oncol.* 2021 Oct 14;11:732883. doi: 10.3389/fonc.2021.732883. | Jianping Xiao |
| Yang S, Zhang W, Chen Q, Guo Q. Clinical Investigation of the Efficacy and Safety of Anlotinib with Immunotherapy in Advanced Non-Small Cell Lung Cancer as Third-Line Therapy: A Retrospective Study. *Cancer Manag Res.* 2020 Oct 19;12:10333-10340. doi: 10.2147/CMAR.S280096. | Qisen Guo |
| Yang SC, Lai WW, Hsu JC, et al. Comparative effectiveness and cost-effectiveness of three first-line EGFR-tyrosine kinase inhibitors: Analysis of real-world data in a tertiary hospital in Taiwan. *PLoS One*. 2020 Apr 8;15(4):e0231413. doi: 10.1371/journal.pone.0231413. | Jung-Der Wang |
| Yang X, Hou Z, Zhu K, et al. Drug-Related Hypertension Associated with the Efficacy of Apatinib on Hepatocellular Carcinoma. *Cancer Manag Res.* 2020 May 6;12:3163-3173. doi: 10.2147/CMAR.S240394. | Ti Zhang |
| Yang X, Qu CX. Adjuvant therapy for HER2 positive pT1a-b pN0 breast cancer: A single center cohort study. *Medicine (Baltimore).* 2022 Jun 24;101(25):e29371. doi: 10.1097/MD.0000000000029371. | Xuan Yang |
| Yang Y, Liu Q, Cao L, et al. Osimertinib versus afatinib in patients with T790M-positive, non-small-cell lung cancer and multiple central nervous system metastases after failure of initial EGFR-TKI treatment. *BMC Pulm Med.* 2021 May 19;21(1):172. doi: 10.1186/s12890-021-01539-x. | Guixing Xu |
| Yang Y, Xu H, Yang G, et al. The value of blood biomarkers of progression and prognosis in ALK-positive patients with non-small cell lung cancer treated with crizotinib. *Asia Pac J Clin Oncol.* 2020 Feb;16(1):63-69. doi: 10.1111/ajco.13284. | Yan Wang |
| Yano Y, Yamamoto A, Minami A, et al. Significance of post-progression therapy after tyrosine kinase inhibitors for advanced hepatocellular carcinoma. *JGH Open*. 2022 May 25;6(6):427-433. doi: 10.1002/jgh3.12772. | Yoshihiko Yano |
| Yao J, Xi W, Chen X, et al. Mast cell density in metastatic renal cell carcinoma: Association with prognosis and tumour-infiltrating lymphocytes. *Scand J Immunol.* 2021 Apr;93(4):e13006. doi: 10.1111/sji.13006. | Jiaxi Yao |
| Yao RJR, Gibson J, Simmons C, Davis MK. Management strategies and clinical outcomes in breast cancer patients who develop left ventricular dysfunction during trastuzumab therapy. *Cardiooncology.* 2021 Mar 26;7(1):12. doi: 10.1186/s40959-021-00099-7. | Margot K. Davis |
| Yao W, Xue M, Lu M, et al. Diffuse Recurrence of Hepatocellular Carcinoma After Liver Resection: Transarterial Chemoembolization (TACE) Combined With Sorafenib Versus TACE Monotherapy. *Front Oncol.* 2020 Dec 17;10:574668. doi: 10.3389/fonc.2020.574668. | Wenzhe Fan |
| Yazici O, Ucar G, Sütcüoglu O, et al. Metastatic colorectal cancer in both sides of Aegean sea: practice patterns and outcome. *Curr Med Res Opin*. 2022 Apr;38(4):579-586. doi: 10.1080/03007995.2022.2037848. | Osman Sütcüoglu |
| Ye L, Mesbah Ardakani N, Thomas C, et al. Detection of Low-level EGFR c.2369 C > T (p.Thr790Met) Resistance Mutation in Pre-treatment Non-small Cell Lung Carcinomas Harboring Activating EGFR Mutations and Correlation with Clinical Outcomes. *Pathol Oncol Res.* 2020 Oct;26(4):2371-2379. doi: 10.1007/s12253-020-00833-z. | Nima Mesbah Ardakani |
| Ye X, Luo X, Du Q, et al. Efficacy and safety of lapatinib in Chinese breast cancer patients: a real-world study. *Ann Transl Med.* 2020 Mar;8(5):240. doi: 10.21037/atm.2020.03.21. | Qing Zhai |
| Ye Z, Deng Z, Jiang S, et al. Radiologic Response Combined with Dermatologic Toxicities is the Most Robust Predictor of Survival Benefits in Patients with Inoperable Hepatocellular Carcinoma After Transarterial Chemoembolization Plus Sorafenib Therapy. *Cardiovasc Intervent Radiol*. 2021 Sep;44(9):1394-1402. doi: 10.1007/s00270-021-02846-w. | Yingqiang Zhang |
| Yeh KH, Yang TS, Hsu TC, et al. Real-world evidence of the safety and effectiveness of regorafenib in Taiwanese patients with metastatic colorectal cancer: CORRELATE Taiwan. *J Formos Med Assoc*. 2021 Nov;120(11):2023-2031. doi: 10.1016/j.jfma.2020.12.015. | Jaw-Yuan Wang |
| Yeh ML, Kuo HT, Huang CI, et al. Eradication of hepatitis C virus preserve liver function and prolong survival in advanced hepatocellular carcinoma patients with limited life expectancy. *Kaohsiung J Med Sci.* 2021 Feb;37(2):145-153. doi: 10.1002/kjm2.12303. | Ming-Lung Yu |
| Yeom SS, Lee SY, Kwak HD, et al. The outcome of primary tumor resection in the unresectable stage IV colorectal cancer patients who received the bevacizumab-containing chemotherapy. *Medicine (Baltimore).* 2020 Feb;99(7):e19258. doi: 10.1097/MD.0000000000019258. | Hyeong Rok Kim |
| Yi Y, Sun BY, Weng JL, et al. Lenvatinib plus anti-PD-1 therapy represents a feasible conversion resection strategy for patients with initially unresectable hepatocellular carcinoma: A retrospective study. *Front Oncol*. 2022 Nov 24;12:1046584. doi: 10.3389/fonc.2022.1046584. | Shuang-Jian Qiu |
| Yi Z, Ma F, Rong G, et al. Clinical spectrum and prognostic value of TP53 mutations in circulating tumor DNA from breast cancer patients in China. *Cancer Commun (Lond).* 2020 Jun;40(6):260-269. doi: 10.1002/cac2.12032. | Binghe Xu |
| Yin S, Chi Y, Du Y, et al. Efficacy and safety of pyrotinib-containing regimen in the patients with HER2-positive metastatic breast cancer: A multicenter real-world study. *Cancer Med.* 2023 Feb;12(3):2333-2344. doi: 10.1002/cam4.5056. | Huihui Li |
| Yin Y, Shu Y, Zhu J, et al. A real-world pharmacovigilance study of FDA Adverse Event Reporting System (FAERS) events for osimertinib. *Sci Rep*. 2022 Nov 15;12(1):19555. doi: 10.1038/s41598-022-23834-1. | Juan Li |
| Yokoe T, Kurozumi S, Nozawa K, et al. Clinical benefit of treatment after trastuzumab emtansine for HER2-positive metastatic breast cancer: a real-world multi-centre cohort study in Japan (WJOG12519B). *Breast Cancer*. 2021 May;28(3):581-591. doi: 10.1007/s12282-020-01192-y. | Toshimi Takano |
| Yokota T, Ota Y, Fujii H, et al. Real-world clinical outcomes and prognostic factors in Japanese patients with recurrent or metastatic squamous cell carcinoma of head and neck treated with chemotherapy plus cetuximab: a prospective observation study (JROSG12-2). *Int J Clin Oncol.* 2021 Feb;26(2):316-325. doi: 10.1007/s10147-020-01817-4. | Tomoya Yokota |
| Yokoyama D, Hisamori S, Deguchi Y, et al. PTEN is a predictive biomarker of trastuzumab resistance and prognostic factor in HER2-overexpressing gastroesophageal adenocarcinoma. *Sci Rep.* 2021 Apr 27;11(1):9013. doi: 10.1038/s41598-021-88331-3. | Shigeo Hisamori |
| Yoo C, Byeon S, Bang Y, et al. Regorafenib in previously treated advanced hepatocellular carcinoma: Impact of prior immunotherapy and adverse events. *Liver Int.* 2020 Sep;40(9):2263-2271. doi: 10.1111/liv.14496. | Baek-Yeol Ryoo |
| Yoodee J, Sookprasert A, Sanguanboonyaphong P, et al. An Exploration of Heart Failure Risk in Breast Cancer Patients Receiving Anthracyclines with or without Trastuzumab in Thailand: A Retrospective Study. *Clin Pract.* 2021 Aug 2;11(3):484-493. doi: 10.3390/clinpract11030064. | Suphat Subongkot |
| Yoshida Y, Sasaoka S, Tanaka M, et al. Analysis of drug-induced hand-foot syndrome using a spontaneous reporting system database. *Ther Adv Drug Saf*. 2022 May 24;13:20420986221101963. doi: 10.1177/20420986221101963. | Mitsuhiro Nakamura |
| Yu AF, Moskowitz CS, Chuy KL, et al. Cardiotoxicity Surveillance and Risk of Heart Failure During HER2 Targeted Therapy. *JACC CardioOncol.* 2020 Jun;2(2):166-175. doi: 10.1016/j.jaccao.2020.03.002. | Anthony F. Yu |
| Yu F, Ni J, Zeng W, et al. Clinical Value of Upfront Cranial Radiation Therapy in Osimertinib-Treated Epidermal Growth Factor Receptor-Mutant Non-Small Cell Lung Cancer With Brain Metastases. *Int J Radiat Oncol Biol Phys.* 2021 Nov 1;111(3):804-815. doi: 10.1016/j.ijrobp.2021.05.125. | Zhengfei Zhu |
| Yu SY, Mckavanagh D, McPherson I, et al. Survival of advanced melanoma patients treated with immunotherapy and targeted therapy: A real-world study. *Pharmacoepidemiol Drug Saf.* 2021 Oct;30(10):1371-1379. doi: 10.1002/pds.5248. | Su-Yeon Yu |
| Yu X, Sheng J, Pan G, Fan Y. Real-world utilization of EGFR TKIs and prognostic factors for survival in EGFR-mutated non-small cell lung cancer patients with brain metastases. *Int J Cancer.* 2021 Sep 1;149(5):1121-1128. doi: 10.1002/ijc.33677. | Yun Fan |
| Yu Z, Dee EC, Bach DQ, et al. Evaluation of a Comprehensive Skin Toxicity Program for Patients Treated With Epidermal Growth Factor Receptor Inhibitors at a Cancer Treatment Center. *JAMA Dermatol*. 2020 Oct 1;156(10):1079-1085. doi: 10.1001/jamadermatol.2020.1795. | Nicole R. LeBoeuf |
| Yuan G, Xie F, Song Y, et al. Hepatic Tumor Stiffness Measured by Shear Wave Elastography Is Prognostic for HCC Progression Following Treatment With Anti-PD-1 Antibodies Plus Lenvatinib: A Retrospective Analysis of Two Independent Cohorts. *Front Immunol*. 2022 Jun 9;13:868809. doi: 10.3389/fimmu.2022.868809. | Jinzhang Chen |
| Yuan J, Cheng F, Xiao G, et al. Efficacy and Safety of Anlotinib in the Treatment of Small Cell Lung Cancer: A Real-World Observation Study. *Front Oncol.* 2022 Jun 20;12:917089. doi: 10.3389/fonc.2022.917089. | Huijie Fan |
| Yuan M, Wang Z, Zhao Y, et al. Cetuximab Can Be an Effective and Low-Toxicity Maintenance Treatment Drug in Patients With Metastatic Colorectal Cancer: A Real-World Study of Zhejiang Cancer Hospital. *Front Pharmacol.* 2021 May 28;12:632076. doi: 10.3389/fphar.2021.632076. | Haijun Zhong |
| Yücel KB, Yekedüz E, Karakaya S, et al. The relationship between systemic immune inflammation index and survival in patients with metastatic renal cell carcinomatreated withtyrosine kinase inhibitors. *Sci Rep.* 2022 Oct 3;12(1):16559. doi: 10.1038/s41598-022-20056-3. | Yüksel Ürün |
| Yucel S, Bilgin B. The prognostic values of systemic immune-inflammation index and derived neutrophil-lymphocyte ratio in EGFR-mutant advanced non-small cell lung cancer. *J Oncol Pharm Pract.* 2021 Jan;27(1):71-77. doi: 10.1177/1078155220913106. | Burak Bilgin |
| Zahler D, Arnold JH, Bar-On T, et al. Valvular Heart Disease following Anthracycline Therapy-Is It Time to Look beyond Ejection Fraction? *Life (Basel).* 2022 Aug 20;12(8):1275. doi: 10.3390/life12081275. | Michal Laufer-Perl |
| Zakharia Y, Thomaidou D, Li B, et al. Real-World Therapy Management and Outcomes of First-Line Axitinib Plus Pembrolizumab in Patients With Advanced Renal Cell Carcinoma in the United States. *Front Oncol.* 2022 May 19;12:861189. doi: 10.3389/fonc.2022.861189. | Yousef Zakharia |
| Zaremba A, Philip M, Hassel JC, et al. Clinical characteristics and therapy response in unresectable melanoma patients stage IIIB-IIID with in-transit and satellite metastases. *Eur J Cancer.* 2021 Jul;152:139-154. doi: 10.1016/j.ejca.2021.04.032. | Anne Zaremba |
| Zarrabi KK, Handorf E, Miron B, et al. Comparative Effectiveness of Front-Line Ipilimumab and Nivolumab or Axitinib and Pembrolizumab in Metastatic Clear Cell Renal Cell Carcinoma. *Oncologist.* 2023 Feb 8;28(2):157-164. doi: 10.1093/oncolo/oyac195. | Daniel M. Geynisman |
| Zeng L, Xiao L, Jiang W, et al. Investigation of efficacy and acquired resistance for EGFR-TKI plus bevacizumab as first-line treatment in patients with EGFR sensitive mutant non-small cell lung cancer in a Real world population. *Lung Cancer.* 2020 Mar;141:82-88. doi: 10.1016/j.lungcan.2020.01.009. | Nong Yang |
| Zeng T, Sun C, Liang Y, et al. A Real-World Multicentre Retrospective Study of Low-Dose Apatinib for Human Epidermal Growth Factor Receptor 2-Negative Metastatic Breast Cancer. *Cancers (Basel).* 2022 Aug 23;14(17):4084. doi: 10.3390/cancers14174084. | Yongmei Yin |
| Zeng Y, Guo T, Zhou Y, et al. Clinical outcomes of advanced non-small cell lung cancer patients harboring distinct subtypes of EGFR mutations and receiving first-line tyrosine kinase inhibitors: brain metastasis and de novo T790M matters. *BMC Cancer.* 2022 Feb 21;22(1):198. doi: 10.1186/s12885-022-09245-5. | Zhengfei Zhu |
| Zengarini C, Mussi M, Veronesi G, et al. BRAF V600K vs. BRAF V600E: a comparison of clinical and dermoscopic characteristics and response to immunotherapies and targeted therapies. *Clin Exp Dermatol.* 2022 Jun;47(6):1131-1136. doi: 10.1111/ced.15113. | Martina Mussi |
| Zhang D, Li S, Zhang X, et al. What predicts the clinical benefits of PARP inhibitors in platinum-sensitive recurrent ovarian cancer: A real-world single-center retrospective cohort study from China. *Front Oncol*. 2022 Aug 18;12:955124. doi: 10.3389/fonc.2022.955124. | Shiqian Zhang |
| Zhang F, Yin Y, Ni T, et al. Treatment effect of apatinib combined chemotherapy as second-line or above therapy in patients with advanced gastric cancer or adenocarcinoma of the gastroesophageal junction. *Pharmazie.* 2020 Aug 1;75(8):389-394. doi: 10.1691/ph.2020.0403. | Ping Li |
| Zhang G, Cheng R, Niu Y, et al. Efficacy Differences of First-line EGFR-TKIs Alone vs in Combination with Chemotherapy in Advanced Lung Adenocarcinoma Patients with Sensitive EGFR Mutation and Concomitant Non-EGFR Genetic Alterations. *Zhongguo Fei Ai Za Zhi.* 2022 Sep 20;25(9):651-657. doi: 10.3779/j.issn.1009-3419.2022.102.34. | Guowei Zhang |
| Zhang HQ, Zhou JM, Zhang SH, et al. Efficacy and safety of low-dose everolimus combined with endocrine drugs for patients with hormone receptor-positive, human epidermal growth factor receptor 2-negative metastatic breast cancer. *Ann Transl Med.* 2021 Oct;9(19):1493. doi: 10.21037/atm-21-4273. | Tao Wang |
| Zhang JX, Chen YX, Zhou CG, et al. Transarterial chemoembolization combined with lenvatinib versus transarterial chemoembolization combined with sorafenib for unresectable hepatocellular carcinoma: A comparative retrospective study. *Hepatol Res.* 2022 Sep;52(9):794-803. doi: 10.1111/hepr.13801. | Qing-Quan Zu |
| Zhang L, Hamdani O, Gjoerup O, et al. *ERBB2* Copy Number as a Quantitative Biomarker for Real-World Outcomes to Anti-Human Epidermal Growth Factor Receptor 2 Therapy in Advanced Gastroesophageal Adenocarcinoma. JCO Precis Oncol. 2022 Jan;6:e2100330. doi: 10.1200/PO.21.00330. | Alexa B. Schrock |
| Zhang L, Song G, Shao B, et al. The efficacy and safety of palbociclib combined with endocrine therapy in patients with hormone receptor-positive HER2-negative advanced breast cancer: a multi-center retrospective analysis. *Anticancer Drugs.* 2022 Jan 1;33(1):e635-e643. doi: 10.1097/CAD.0000000000001210. | Huiping Li |
| Zhang L, Sun JH, Hou ZH, et al. Prognosis Nomogram for Hepatocellular Carcinoma Patients with Portal Vein Invasion Undergoing Transarterial Chemoembolization Plus Sorafenib Treatment: A Retrospective Multicentre Study. *Cardiovasc Intervent Radiol.* 2021 Jan;44(1):63-72. doi: 10.1007/s00270-020-02579-2. | Cai-Fang Ni |
| Zhang L, Wu X, Zhou J, et al. Pyrotinib in the Treatment of Women With HER2-Positive Advanced Breast Cancer: A Multicenter, Prospective, Real-World Study. *Front Oncol.* 2021 Jul 16;11:699323. doi: 10.3389/fonc.2021.699323. | Jifeng Feng |
| Zhang L, Xu T, Li Y, et al. Serum hsa_circ_0000615 is a prognostic biomarker of sorafenib resistance in hepatocellular carcinoma. *J Clin Lab Anal.* 2022 Nov;36(11):e24741. doi: 10.1002/jcla.24741. | Xiaolin Ding |
| Zhang L, Yan ZP, Hou ZH, et al. Neutrophil-to-Lymphocyte and Platelet-to-Lymphocyte Ratios as Predictors of Outcomes in Patients With Unresectable Hepatocellular Carcinoma Undergoing Transarterial Chemoembolization Plus Sorafenib. *Front Mol Biosci.* 2021 May 28;8:624366. doi: 10.3389/fmolb.2021.624366. | Qiang Li |
| Zhang M, Li L, Zhang S, et al. Efficacy of Neoadjuvant Chemotherapy with Epirubicin and Cyclophosphamide and Weekly Paclitaxel and Trastuzumab in Human Epidermal Growth Factor Receptor 2-Positive Breast Carcinoma: A Real-World Study. *Biomed Res Int.* 2020 May 2;2020:3208391. doi: 10.1155/2020/3208391. | Haisong Yang |
| Zhang Q, Chen M, Wang Z, et al. Efficacy and Safety Comparison of Regorafenib and Fruquintinib in Metastatic Colorectal Cancer-An Observational Cohort Study in the Real World. *Clin Colorectal Cancer*. 2022 Sep;21(3):e152-e161. doi: 10.1016/j.clcc.2022.01.007. | Jian Li |
| Zhang RS, Liu J, Deng YT, et al. The real-world clinical outcomes and treatment patterns of patients with unresectable locally advanced or metastatic soft tissue sarcoma treated with anlotinib in the post-ALTER0203 trial era. *Cancer Med*. 2022 Jun;11(11):2271-2283. doi: 10.1002/cam4.4613. | Yu Jiang |
| Zhang W, Wu L, Chen L, et al. The efficacy of drug-eluting bead or conventional transarterial chemoembolization plus apatinib for hepatocellular carcinoma with portal vein tumor thrombus. *Sci Rep.* 2022 Apr 6;12(1):5725. doi: 10.1038/s41598-022-09609-8. | Chuansheng Zheng |
| Zhang W, Zhang Y, Zhao Q, et al. Long-term safety of icotinib in patients with non-small cell lung cancer: a retrospective, real-world study. *J Thorac Dis.* 2020 Mar;12(3):639-650. doi: 10.21037/jtd.2019.12.115. | Fenlai Tan |
| Zhang W, Zhang Z, Lou S, et al. Efficacy, safety and predictors of combined fruquintinib with programmed death-1 inhibitors for advanced microsatellite-stable colorectal cancer: A retrospective study. *Front Oncol.* 2022 Aug 31;12:929342. doi: 10.3389/fonc.2022.929342. | Weijie Zhang |
| Zhang X, Li Z, Han L, et al. Efficacy and Safety of Pyrotinib in Human Epidermal Growth Factor Receptor 2-Positive Advanced Breast Cancer: A Multicenter, Retrospective, Real-World Study. *Onco Targets Ther.* 2022 Sep 28;15:1067-1078. doi: 10.2147/OTT.S379591. | Man Li |
| Zhang X, Wang F, Gu G, Wu Q. High HBV Load Weakens Predictive Effect of Serum miR-122 on Response to Sorafenib in Hepatocellular Carcinoma Patients. *J Oncol.* 2021 Jun 11;2021:9938207. doi: 10.1155/2021/9938207. | Xiaomin Zhang |
| Zhang X, Zeng L, Li Y, et al. Anlotinib combined with PD-1 blockade for the treatment of lung cancer: a real-world retrospective study in China. *Cancer Immunol Immunother*. 2021 Sep;70(9):2517-2528. doi: 10.1007/s00262-021-02869-9. | Nong Yang |
| Zhang Y, Miao H, Xie W, et al. The PPRD score stratifies patients with hepatocellular carcinoma and portal vein tumor thrombus treated with sorafenib plus transarterial chemoembolization. *Eur Radiol*. 2021 Jan;31(1):232-243. doi: 10.1007/s00330-020-07078-z. | Yong Chen |
| Zhang Y, Wu JL, Li LQ. Efficacy comparison of optimal treatments for hepatocellular carcinoma patients with portal vein tumor thrombus. *Ann Hepatol.* 2022 Jan-Feb;27(1):100552. doi: 10.1016/j.aohep.2021.100552. | Le-Qun Li |
| Zhang Y, Zhang X, Zhang R, et al. Clinical and molecular factors that impact the efficacy of first-line crizotinib in ROS1-rearranged non-small-cell lung cancer: a large multicenter retrospective study. *BMC Med*. 2021 Sep 13;19(1):206. doi: 10.1186/s12916-021-02082-6. | Yongchang Zhang |
| Zhang Z, Zhang L, Yu G, et al. Exosomal miR-1246 and miR-155 as predictive and prognostic biomarkers for trastuzumab-based therapy resistance in HER2-positive breast cancer. *Cancer Chemother Pharmacol.* 2020 Dec;86(6):761-772. doi: 10.1007/s00280-020-04168-z. | Chi Zhang |
| Zhao L, Chang N, Shi L, et al. Lenvatinib plus sintilimab versus lenvatinib monotherapy as first-line treatment for advanced HBV-related hepatocellular carcinoma: A retrospective, real-world study. *Heliyon.* 2022 May 25;8(6):e09538. doi: 10.1016/j.heliyon.2022.e09538. | Fusheng Wang |
| Zhao S, Dou W, Fan Q, et al. Identifying optimal candidates of transarterial chemoembolization (TACE) vs. sorafenib in patients with unresectable hepatocellular carcinoma. *Ann Transl Med*. 2020 May;8(9):587. doi: 10.21037/atm.2020.02.123. | Lei Liu |
| Zhao W, Bian L, Wang T, et al. Effectiveness of second-line anti-HER2 treatment in HER2-positive metastatic breast cancer patients previously treated with trastuzumab: A real-world study. *Chin J Cancer Res.* 2020 Jun;32(3):361-369. doi: 10.21147/j.issn.1000-9604.2020.03.07. | Zefei Jiang |
| Zhao Y, Li S, Yang X, et al. Overall survival benefit of osimertinib and clinical value of upfront cranial local therapy in untreated EGFR-mutant nonsmall cell lung cancer with brain metastasis. *Int J Cancer*. 2022 Apr 15;150(8):1318-1328. doi: 10.1002/ijc.33904. | Zhengfei Zhu |
| Zhao Y, Xie N, Li W, et al. Real-world effectiveness of eribulin in heavily pretreated patients with metastatic breast cancer in China: a multicenter retrospective study. *Ther Adv Med Oncol*. 2021 Jul 9;13:17588359211030210. doi: 10.1177/17588359211030210. | Biyun Wang |
| Zheng H, Gao Y, Guo H, et al. Real-world Experience of Olaparib Treatment in Patients with Ovarian Cancer: A Chinese Multicenter Study. *Mol Cancer Ther*. 2021 Sep;20(9):1735-1742. doi: 10.1158/1535-7163.MCT-20-1064. | Yunong Gao |
| Zheng H, Pan Q, Zhu W, et al. Novel Nutrition-Based Nomograms to Assess the Outcomes of Lung Cancer Patients Treated With Anlotinib or Apatinib. *Front Oncol.* 2021 Mar 8;11:628693. doi: 10.3389/fonc.2021.628693. | Hongming Pan |
| Zheng HR, Jiang AM, Gao H, et al. The Efficacy and Safety of Anlotinib in Extensive-Stage Small Cell Lung Cancer: A Multicenter Real-World Study. *Cancer Manag Res.* 2022 Aug 2;14:2273-2287. doi: 10.2147/CMAR.S364125. | Yu Yao |
| Zheng L, Gu X, Zheng G, et al. Prediction of early recurrence and response to adjuvant Sorafenib for hepatocellular carcinoma after resection. *PeerJ.* 2021 Nov 26;9:e12554. doi: 10.7717/peerj.12554. | Longgen Liu |
| Zheng Z, Liu Z, Zhang H, et al. Efficacy and Safety of Apatinib in Advanced Hepatocellular Carcinoma: A Multicenter Real World Retrospective Study. *Front Pharmacol.* 2022 May 17;13:894016. doi: 10.3389/fphar.2022.894016. | Ying Xin |
| Zhong BY, Yan ZP, Sun JH, et al. Prognostic Performance of Albumin-Bilirubin Grade With Artificial Intelligence for Hepatocellular Carcinoma Treated With Transarterial Chemoembolization Combined With Sorafenib. *Front Oncol.* 2020 Dec 18;10:525461. doi: 10.3389/fonc.2020.525461. | Cai-Fang Ni |
| Zhong BY, Yan ZP, Sun JH, et al. Random Survival Forests to Predict Disease Control for Hepatocellular Carcinoma Treated With Transarterial Chemoembolization Combined With Sorafenib. *Front Mol Biosci.* 2021 May 20;8:618050. doi: 10.3389/fmolb.2021.618050. | Cai-Fang Ni |
| Zhong Q, Liu Z. Efficacy and Safety of Anlotinib in Patients with Advanced Non-Small Cell Lung Cancer: A Real-World Study. *Cancer Manag Res*. 2021 May 20;13:4115-4128. doi: 10.2147/CMAR.S304838. | Zhihua Liu |
| Zhong Y, Wei Q, Lu Y, et al. Efficacy and safety of anlotinib in patients with advanced non-small cell lung cancer. *J Thorac Dis.* 2020 Oct;12(10):6016-6022. doi: 10.21037/jtd-20-2855. | Lingxiang Chen |
| Zhou L, Li H, Yang S. Age does matter in adolescents and young adults vs. older adults with lung adenocarcinoma: A retrospective analysis comparing clinical characteristics and outcomes in response to systematic treatments. *Oncol Lett.* 2022 Aug 31;24(4):362. doi: 10.3892/ol.2022.13482. | Shuhui Yang |
| Zhou LN, Feng CX, Zhang Y, et al. The bevacizumab plus oxaliplatin-based chemotherapy regimen is more suitable for metastatic colorectal cancer patients with a history of schistosomiasis: a clinical retrospective analysis. *J Gastrointest Oncol.* 2022 Jun;13(3):1086-1096. doi: 10.21037/jgo-22-207. | Jun Jin |
| Zhou Q, Wang X, Li R, et al. Sorafenib as adjuvant therapy following radiofrequency ablation for recurrent hepatocellular carcinoma within Milan criteria: a multicenter analysis. *J Gastroenterol*. 2022 Sep;57(9):684-694. doi: 10.1007/s00535-022-01895-3. | Qunfang Zhou |
| Zhou Y, Song L, Xu Q, et al. Investigation on the survival implications of PD-L1 expression status in ALK- rearranged advanced non-small cell lung cancer treated with first-line crizotinib. *Lung Cancer*. 2022 May;167:58-64. doi: 10.1016/j.lungcan.2022.04.002. | Yongchang Zhang |
| Zhou YW, Long YX, Chen Y, et al. First-line therapy of bevacizumab plus chemotherapy versus cetuximab plus chemotherapy for metastatic colorectal cancer patients with mucinous adenocarcinoma or mucinous component. *Cancer Med.* 2021 May;10(10):3388-3402. doi: 10.1002/cam4.3876. | Meng Qiu |
| Zhou YW, Long YX, Liu X, et al. Tumor calcification is associated with better survival in metastatic colorectal cancer patients treated with bevacizumab plus chemotherapy. *Future Oncol.* 2022 Jul;18(22):2453-2464. doi: 10.2217/fon-2021-1422. | Meng Qiu |
| Zhou YW, Wang JL, Li QF, et al. Efficacy and safety of raltitrexed plus S-1 versus regorafenib in patients with refractory metastatic colorectal cancer: a real-world propensity score matching study. *Therap Adv Gastroenterol*. 2022 May 18;15:17562848221098246. doi: 10.1177/17562848221098246. | Meng Qiu |
| Zhou Z, Wang C, Ying L, et al. Anaplastic lymphoma kinase tyrosine kinase inhibitor-induced hepatic failure in lung cancer patients: A study of signal mining and analysis of the FDA adverse event reporting system database. *J Clin Pharm Ther*. 2021 Aug;46(4):1148-1154. doi: 10.1111/jcpt.13404. | Dawei Shi |
| Zhu Y, Sun P, Wang K, et al. Efficacy and safety of lenvatinib monotreatment and lenvatinib-based combination therapy for patients with unresectable hepatocellular carcinoma: a retrospective, real-world study in China. *Cancer Cell Int.* 2021 Sep 18;21(1):503. doi: 10.1186/s12935-021-02200-7. | Yang Cheng |
